# Multi-omics analysis of the cervical epithelial integrity of women using depot medroxyprogesterone acetate

**DOI:** 10.1371/journal.ppat.1010494

**Published:** 2022-05-09

**Authors:** Frideborg Bradley, Mathias Franzén Boger, Vilde Kaldhusdal, Alexandra Åhlberg, Gabriella Edfeldt, Julie Lajoie, Sofia Bergström, Kenneth Omollo, Anastasios Damdimopoulos, Paulo Czarnewski, Anna Månberg, Julius Oyugi, Joshua Kimani, Peter Nilsson, Keith Fowke, Annelie Tjernlund, Kristina Broliden

**Affiliations:** 1 Department of Medicine Solna, Division of Infectious Diseases, Karolinska Institutet, Department of Infectious Diseases, Karolinska University Hospital, Center for Molecular Medicine, Stockholm, Sweden; 2 Department of Medical Microbiology and Infectious Diseases, University of Manitoba, Winnipeg, Canada; 3 Department of Medical Microbiology, University of Nairobi, Nairobi, Kenya; 4 Division of Affinity Proteomics, Department of Protein Science, SciLifeLab, KTH Royal Institute of Technology, Stockholm, Sweden; 5 Bioinformatics and Expression Analysis core facility, Department of Biosciences and Nutrition, Karolinska Institutet, Huddinge, Sweden; 6 Department of Biochemistry and Biophysics, National Bioinformatics Infrastructure Sweden, SciLifeLab, Stockholm University, Solna, Sweden; 7 Partners for Health and Development in Africa, Nairobi, Kenya; 8 Department of Community Health Sciences, University of Manitoba, Winnipeg, Canada; Burnet Institute, AUSTRALIA

## Abstract

Depot medroxyprogesterone acetate (DMPA) is an injectable hormonal contraceptive used by millions of women worldwide. However, experimental studies have associated DMPA use with genital epithelial barrier disruption and mucosal influx of human immunodeficiency virus (HIV) target cells. We explored the underlying molecular mechanisms of these findings. Ectocervical biopsies and cervicovaginal lavage (CVL) specimens were collected from HIV-seronegative Kenyan sex workers using DMPA (*n* = 32) or regularly cycling controls (*n* = 64). Tissue samples were assessed by RNA-sequencing and quantitative imaging analysis, whereas protein levels were measured in CVL samples. The results suggested a DMPA-associated upregulation of genes involved in immune regulation, including genes associated with cytokine-mediated signaling and neutrophil-mediated immunity. A transcription factor analysis further revealed DMPA-associated upregulation of RELA and NFKB1 which are involved in several immune activation pathways. Several genes significantly downregulated in the DMPA versus the control group were involved in epithelial structure and function, including genes encoding keratins, small proline-rich proteins, and cell-cell adhesion proteins. Pathway analyses indicated DMPA use was associated with immune activation and suppression of epithelium development, including keratinization and cornification processes. The cervicovaginal microbiome composition (*Lactobacillus* dominant and non-*Lactobacillus* dominant) had no overall interactional impact on the DMPA associated tissue gene expression. Imaging analysis verified that DMPA use was associated with an impaired epithelial layer as illustrated by staining for the selected epithelial junction proteins E-cadherin, desmoglein-1 and claudin-1. Additional staining for CD4^+^ cells revealed a more superficial location of these cells in the ectocervical epithelium of DMPA users versus controls. Altered protein levels of SERPINB1 and ITIH2 were further observed in the DMPA group. Identification of specific impaired epithelial barrier structures at the gene expression level, which were verified at the functional level by tissue imaging analysis, illustrates mechanisms by which DMPA adversely may affect the integrity of the genital mucosa.

## Author summary

Sexual transmission accounts for the majority of all new HIV infections in women, and alterations to the mucosal environment of the female genital tract have been associated with an increase in the risk of acquiring HIV. Observational epidemiological studies have implied that the use of the injectable hormonal contraceptive depot medroxyprogesterone acetate (DMPA) may be associated with increased HIV-acquisition. However, a prospective clinical study has not confirmed this association and the controversial findings are currently evaluated in the context of international reproductive health policies. Several studies using various model systems indicate that DMPA affects the integrity of the genital epithelial barrier as well as the mucosal immune system, but the exact mechanisms remain largely unknown. To characterize the effect of DMPA on the genital mucosal environment, we used a multi-omics approach to assess paired genital secretions and cervical tissue samples from long-term regular DMPA users living in Kenya. This unique cohort represents a population at risk of HIV infection in which DMPA is one of the most commonly used hormonal contraceptives. We identified impaired cervical epithelial barrier structures, including DMPA-associated reduction in the expression of cell-cell adhesion molecules, keratins, small proline-rich proteins and a thinner upper epithelial layer with more superficially located CD4^+^ cells. Gene set enrichment pathway analyses indicated DMPA use was associated with immune activation and suppression of epithelium development including keratinization and cornification pathways. Protein analysis identified altered levels of selected anti-proteases. Our findings illustrate mechanisms by which DMPA adversely may affect the integrity of the genital mucosa.

## Introduction

Sub-Saharan Africa bears the greatest burden of human immunodeficiency virus type 1 (HIV) infection, with approximately 21 million people living with HIV in Eastern and Southern Africa [1]. In women, female sex hormones affect the susceptibility to HIV infection by altering both the structure of the genital mucosa and the immune repertoire, as seen during the menstrual cycle and with the use of hormonal contraception [2,3]. The genital microbiota also significantly affects the risk of HIV infection in women [4], as does genital inflammation, with elevated concentrations of HIV target cell-recruiting chemokines serving as the strongest predictor of increased susceptibility [5].

Depot medroxyprogesterone acetate (DMPA) is a highly effective, long-acting, injectable hormonal contraceptive used by millions of women worldwide [6]. However, meta-analyses of high-quality observational studies have suggested that DMPA use is associated with a 40–50% increase in the risk of acquiring HIV relative to no use of hormonal contraceptives [7,8]. A large randomized, multicenter study further compared DMPA with two other types of long-lasting contraceptives—levonorgestrel implant and copper-intrauterine device [9]. Here, DMPA usage was not found to be associated with an increased risk for HIV acquisition, although the study design, including appropriate comparator groups, has been questioned and a clear epidemiological link between HIV infection risk and DMPA use remains to be established [10].

Several studies in humans and animal models have nevertheless convincingly demonstrated that DMPA significantly affects the genital mucosa at the cellular and molecular levels [2,11–20]. These effects include impaired mucosal integrity, altered expression of phenotypic and activation markers on immune cells, and reduced concentrations of genital growth factors.

In multi-layered epithelia such as the ectocervical mucosa, penetration by HIV is blocked by epithelial junctions that form an adherent barrier in the intermediate and basal layers [21]. Characterizing patterns of gene expression in mucosal tissues could therefore enhance understanding of the molecular mechanisms underlying altered susceptibility to infection with viruses such as HIV, as well as elucidate the role of hormones in viral infection risk [15,16,20,22,23].

In the present study, we expanded upon previous research by investigating a large set of ectocervical tissue samples obtained from women who were long-term users of DMPA living in an HIV-endemic geographical region. Our multi-omics analyses revealed DMPA-associated impairments of the ectocervical epithelial integrity. In theory, this may increase permeability to encountered pathogens, including HIV.

## Results

### Sociodemographic data and clinical characterization of study participants

Samples were obtained from 96 female sex workers from Nairobi, Kenya. Thirty-two of the participants had used DMPA for at least 6 months, whereas 64 participants (“controls”) did not use any hormonal contraceptives and had regular menstrual cycles (Table 1). Participants consented to refrain from vaginal intercourse throughout the four-week study period, with study sampling two weeks into this period, as well as to regular testing for presence of prostate-specific antigen (PSA) as a follow-up of these instructions. This study design aimed to reduce the effect of possible differences in sexual behaviors between the study groups, as well as a safety precaution for the mucosal sampling. No significant differences were observed between the study groups with regard to PSA positivity, having a regular partner, cervicovaginal microbiome composition, or bacterial vaginosis (BV). One woman in the DMPA group tested positive for *Neisseria gonorrhoeae* at the study visit (although negative at the enrollment visit). Women in the DMPA group were significantly younger than those of the control group (median age 30 *vs*. 34 years; *P* = 0.01) and reported being engaged in sex work for a significantly shorter time (median 24 months *vs*. 36 months; *P*<0.001). These two parameters were therefore included as confounders in the statistical analyses in all experimental analyses. All women remained HIV seronegative for 3–6 months after completion of the study.

**Table 1:** Sociodemographic data and clinical characteristics of study subjects.

	DMPA group (n = 32)	Control group (n = 64)	P-value
	Number or median (range or %)	Number or median (range or %)	
**Age (years)**	30 (22, 41)	34 (21, 50)	**0.01** ^**1**^
**Months in sex work**	24 (3, 36)	36 (4, 372)	**< 0.001** ^**1**^
**Positive PSA test** *	1 (3%)	5 (8%)	0.66^**2**^
**Self-reported days since onset of last menses** **	N/A	9 (3, 44)	
- Not available		3	
**Plasma hormone levels**			
*Estradiol (pg/ml)*	22 (22, 124)	93 (22, 405)	**< 0.001** ^**1**^
- Below LLD (22 pg/ml)***	17 (54%)	6 (9%)	**< 0.001** ^**3**^
- Not available	1 (3%)	0	
*Progesterone (ng/ml)*	0.05 (0.05, 0.09)	0.05 (0.05, 19)	**< 0.001** ^**1**^
- Below LLD (0.05 ng/ml)***	30 (97%)	34 (53%)	**< 0.001** ^**3**^
- Not available	1 (3%)	0	
**Having a regular partner**			0.87^**3**^
- Yes	19 (59%)	36 (56%)	
- No	12 (38%)	27 (42%)	
- Not available	1 (3%)	1 (2%)	
**Cervicovaginal microbiome composition**			0.50^2^
- L1 (*L*.*crispatus/jensenii*)	3 (9%)	6 (9%)	
- L2 (*L*. *iners*)	11 (34%)	16 (25%)	
- L3 (*Gardnerella*)	6 (19%)	12 (19%)	
- L4 (High diverse)	8 (25%)	26 (41%)	
- L5 (Other)	4 (13%)	4 (6%)	
**Bacterial Vaginosis (BV; based on Nugent Score)**			0.21^**3**^
- BV	7 (22%)	23 (36%)	
- Intermediate	5 (16%)	16 (25%)	
- Normal	17 (53%)	25 (39%)	
- Not available	3 (9%)	0	

*Prostate-specific antigen (PSA) test: a positive value represents >1 ng/mL.

**Only applicable for samples in control group

*** Percentage based on total number of samples with values (i.e. excluding not available samples) in denominator, n = 31 in DMPA group and n = 64 in control group

LLD: Lower limit of detection. N/A: not applicable.

^1^ Mann-Whitney *U* test.

^2^ Fischer’s exact test.

^**3**^ Pearson’s Chi-squared test. Significant p-values in bold

### Plasma levels of estradiol and progesterone correlate with DMPA use

Plasma estradiol (E2) and progesterone (P4) levels were significantly lower in the DMPA group than control group (E2: median 22 pg/mL *vs*. 93 pg/mL; *P*<0.001; P4: median 0.05 ng/mL *vs*. 0.05 ng/mL; *P*<0.001), consistent with previous data demonstrating suppression of ovarian steroidogenesis in DMPA users (Tables 1 and S1 and S1A and S1B Fig) [24]. In the DMPA group, 55% (17 of 31) and 97% (30 of 31) of subjects had E2 and P4 values, respectively, that fell below the lower limit of detection (LLD), as compared with only 9% (6 of 64) and 53% (34 of 64) of controls. Samples from three subjects in the DMPA group exhibited E2 levels >90 pg/mL, while the P4 concentration was below the LLD for all three samples. The sampling in the control group was aimed for the follicular phase of the menstrual cycle, and the median number of self-reported days since onset of the last menses was 9 days (range 3–44 days) (Table 1 and S1C Fig). Of the 64 subjects in the control group, 46 (72%) had a progesterone level <0.3 ng/mL, a value considered to define the follicular phase [12].

### DMPA use is associated with altered expression of genes regulating immune function and the epithelial barrier

**Fig 1:**
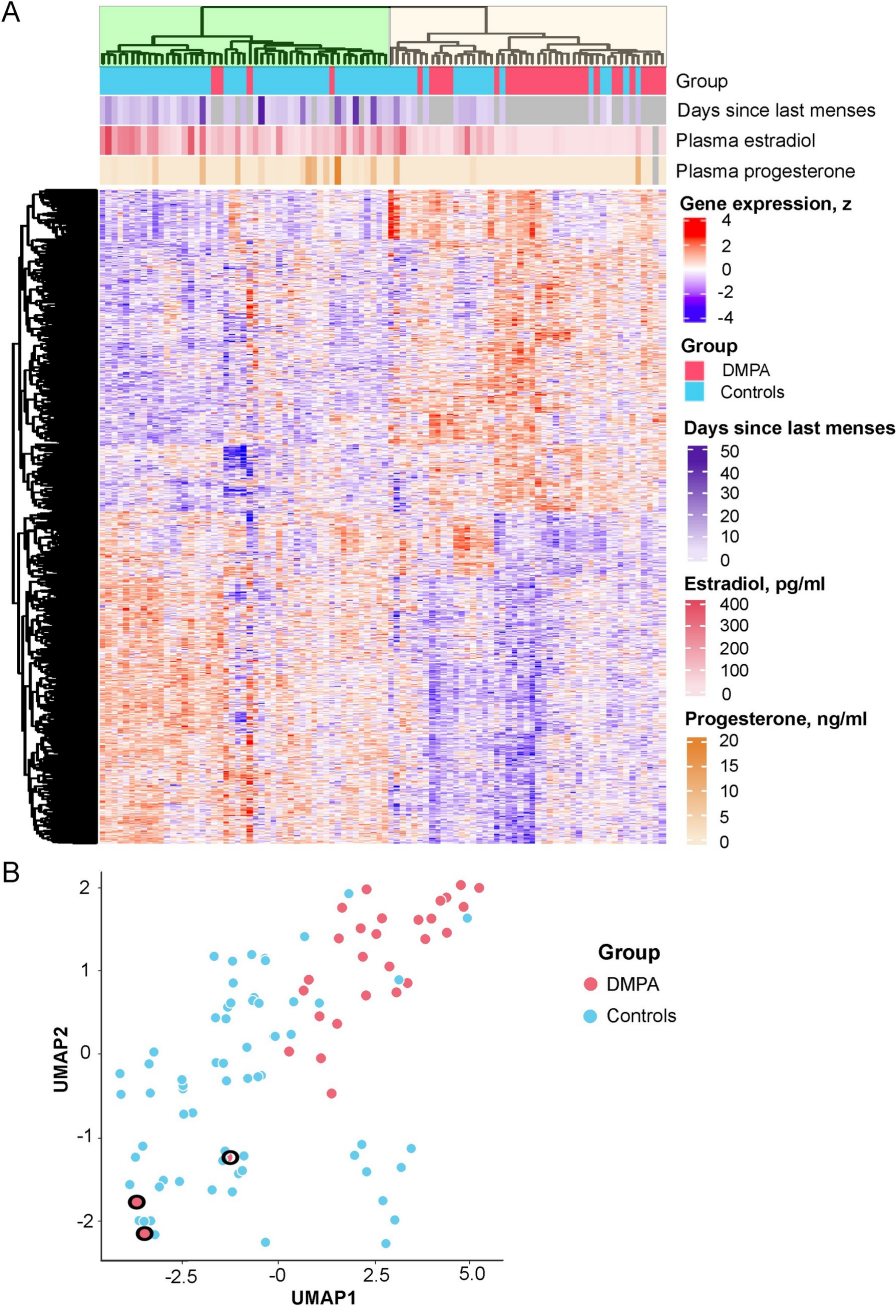
Hierarchical clustering of differentially expressed genes shows separation of DMPA vs. control samples. A) Heatmap of all 2,317 DEGs (FDR-adjusted *P* <0.05) identified between the DMPA and the control group. Each sample is represented by a vertical column, and each gene by a horizontal row. The expression of each gene/row is standardized (z) to a mean of 0 and standard deviation of 1. The blue color indicates below average expression while red indicates above average expression. Samples representing the DMPA group are shown in pink and samples from the control group in turquoise. The horizontal bars below groups show days since onset of last menses, plasma estradiol (pg/ml) and plasma progesterone (ng/ml) levels. Samples with missing data for that parameter are shown in grey. The samples clustered into two major groups marked in green and yellow. B) Dimensionality reduction plot (UMAP) of samples based on all DEGs, displaying partially overlapping clusters of DMPA and control samples. Three subjects in the DMPA group exhibited E2 levels >90 pg/mL and are marked with circles in the figure. DEGs: differentially expressed genes. FDR: false discovery rate. UMAP: Uniform manifold approximation and projection.

To characterize in an unbiased manner the effect of DMPA use on gene expression in the epithelium, we performed RNA-sequencing (RNA-seq) analysis of ectocervical tissue biopsies. Of 15,326 genes detected in the RNA-seq dataset, 2,317 (15%) were found to be differentially expressed (false discovery rate [FDR]-adjusted *P*<0.05) between the DMPA and control groups after adjusting for the potential confounders age and time engaged in sex work (S2 Table). To assess clustering of samples based on expression data, hierarchical clustering and uniform manifold approximation and projection (UMAP) unsupervised dimensionality reduction analysis of differentially expressed genes (DEGs) were performed. These analyses revealed two partially overlapping clusters, one composed primarily of samples from the DMPA group, and the other composed of control samples (Fig 1A and 1B). The sample from the participant with *N*. *gonorrhoeae* clustered with the other samples from the DMPA group in the DMPA cluster. Interestingly, the three samples from the DMPA group in which the E2 level was >90 pg/mL clustered with the control samples.

**Fig 2:**
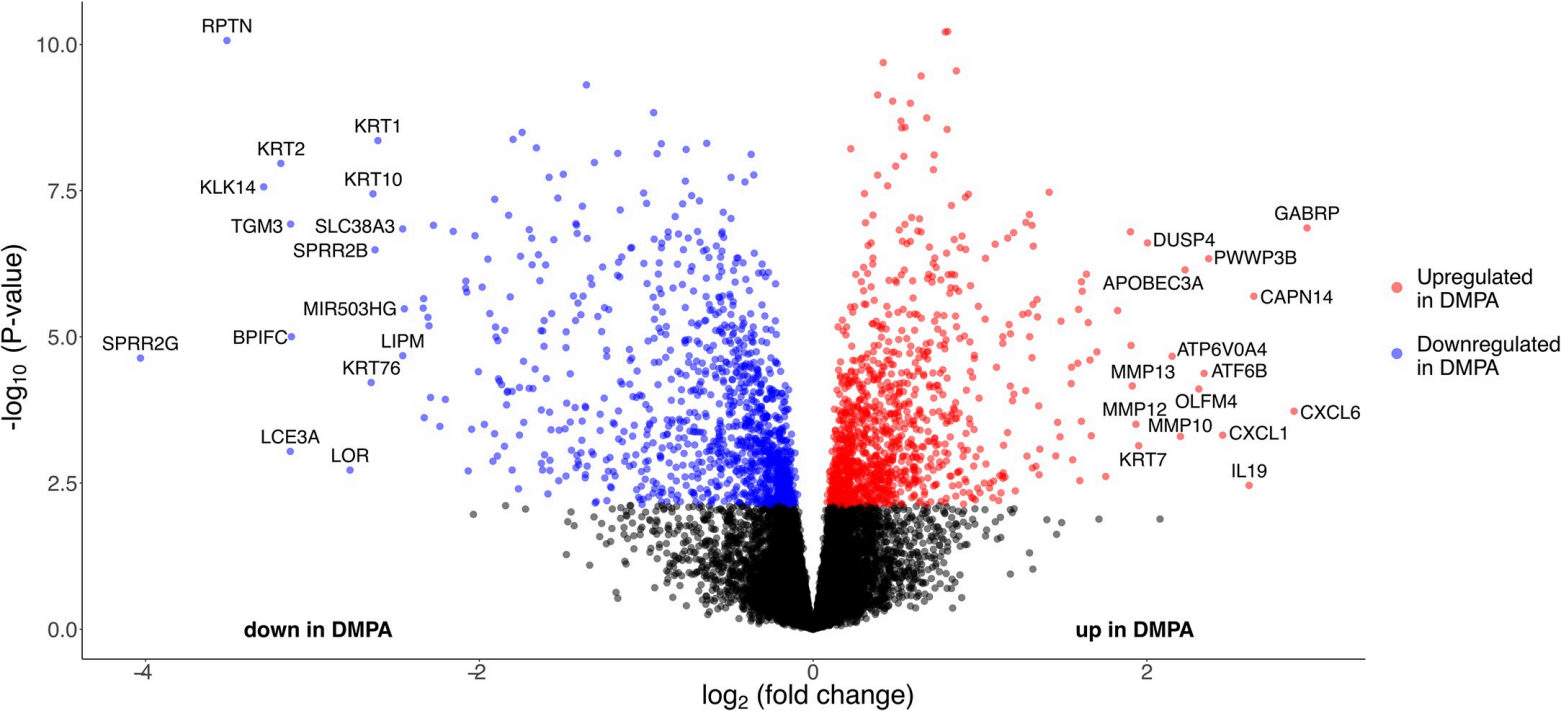
DMPA use associated with significant changes in genes involved in immune regulation and epithelial barrier structure and function in ectocervical tissue samples. Volcano plot of all genes in the data set, showing log_2_FC on the x-axis and -log_10_ (p-value) on the y-axis. Each gene is represented by a dot. DEGs (FDR-adjusted *P* <0.05) that are upregulated in the DMPA group are shown in red, and DEGs downregulated in the DMPA group are shown in blue. DEGs: differentially expressed genes. Log_2_FC: log_2_ fold change. FDR: false discovery rate.

To examine the molecular mechanism underlying the effects of DMPA use in more detail, we categorized the genes exhibiting a difference in expression profile between the study groups. The top-15 upregulated DEGs based on log_2_-fold change (FC) (Fig 2 and Table 2) represented a wide range of functions, including mucosal barrier disruption by the protease *CAPN14* [25], cytokines/chemokines (*CXCL1*, *CXCL6*, *IL19*), and members of the matrix metalloproteinase family (*MMP10*, *MMP12*, *MMP13*).

**Table 2:** The top 15 most differentially expressed genes according to log_2_ fold change between the DMPA and the control group.

HGNC ID	Gene name	Uniprot ID	Log_2_FC	FDR adj. P-value	EB*
*Genes upregulated in DMPA group*					
*GABRP*	gamma-aminoburyric acid type A receptor pi subunit	O00591	2.96	2.8E-05	
*CXCL6*	C-X-C motif chemokine 6	P80162	2.88	3.7E-03	
*CAPN14*	calpain 14	A8MX76	2.64	1.5E-04	X
*IL19*	Interleukin 19	Q9UHD0	2.61	2.9E-02	X
*CXCL1*	C-X-C motif chemokine ligand 1	P09341	2.45	7.3E-03	X
*PWWP3B*	PWWP domain containing 3B	Q5H9M0	2.37	5.3E-05	
*ATF6B*	activating transcription factor 6 beta	Q99941	2.34	1.3E-03	
*OLFM4*	olfactomedin 4	Q6UX06	2.31	1.9E-03	
*APOBEC3A*	apolipoprotein B mRNA editing enzyme catalytic subunit 3A	P31941	2.23	7.2E-05	
*MMP10*	matrix metallopeptidase 10	P09238	2.20	7.5E-03	X
*ATP6V0A4*	ATPase H+ transporting V0 subunit a4	Q9HBG4	2.15	8.0E-04	
*DUSP4*	dual specificity phosphatase 4	Q13115	2.00	3.6E-05	
*KRT7*	keratin 7	P08729	1.95	9.7E-03	X
*MMP12*	matrix metallopeptidase 12	P39900	1.93	5.3E-03	X
*MMP13*	matrix metallopeptidase 13	P45452	1.91	1.8E-03	X
*Genes downregulated in DMPA group*					
*SPRR2G*	small proline-rich protein 2G	Q9BYE4	-4.03	8.4E-04	X
*RPTN*	repetin	Q6XPR3	-3.51	4.4E-07	X
*KLK14*	kallikrein related peptidase 14	Q9P0G3	-3.29	9.9E-06	X
*KRT2*	keratin 2	P35908	-3.19	5.3E-06	X
*LCE3A*	late cornified envelope 3A	Q5TA76	-3.13	1.1E-02	X
*TGM3*	transglutaminase 3	Q08188	-3.13	2.6E-05	X
*BPIFC*	BPI fold containing family C	Q8NFQ6	-3.13	4.5E-04	
*LOR*	loricrin	P23490	-2.77	1.9E-02	X
*KRT76*	keratin 76	Q01546	-2.65	1.6E-03	X
*KRT10*	keratin 10	P13645	-2.64	1.2E-05	X
*SPRR2B*	small proline-rich protein 2B	P35325	-2.63	4.2E-05	X
*KRT1*	keratin 1	P04264	-2.61	3.5E-06	X
*SLC38A3*	solute carrier family 38 member 3	Q99624	-2.46	2.9E-05	
*LIPM*	lipase family member M	Q5VYY2	-2.46	7.8E-04	
*MIR503HG*	long intergenic non-coding RNAs	-	-2.45	2.1E-04	

HGNC: HUGO Gene Nomenclature Committee. Log_2_FC: Log_2_ fold change. FDR: false discovery rate. Adj.: adjusted. EB: Epithelial barrier *Involved in epithelial barrier function according to published literature.

Of the top-15 downregulated DEGs, several encode proteins involved in epithelial barrier function (Table 2), including the genes *SPRR2G*, *RPTN*, *LCE3A*, *TGM3*, *LOR*, *SPRR2B* as well as keratin genes *KRT1*, *KRT2*, *KRT10* and *KRT76*. Notably, several genes associated with desmosome function, such as *DSG1*, *DSC1*, *DSC2*, *PKP1*, *PKP3*, *JUP* and *DSP* exhibited decreased expression among DMPA users (S2 Table), although they were not among the top-15 DEGs. However, unlike other desmosomal cadherin genes, *DSG2* was upregulated in the DMPA group. Overall, these data indicate that the transcriptional profile of DMPA users is characterized by upregulation of immunoregulatory genes and downregulation of genes associated with the epithelial barrier integrity.

### DMPA use correlates with immune regulation and epithelial barrier structure and function

**Fig 3:**
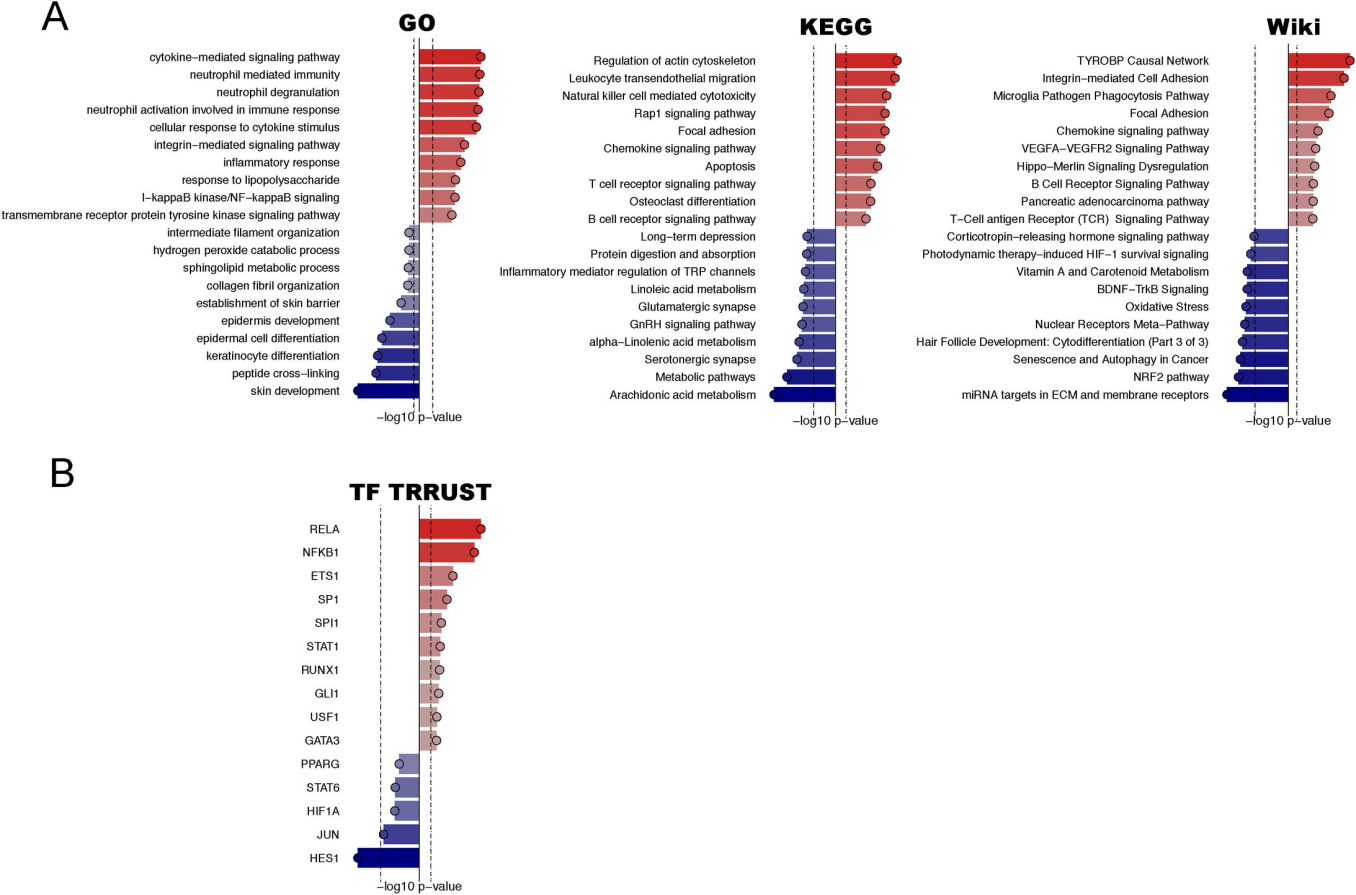
Gene set enrichment analysis identifies altered pathways involved in immune regulation and epithelial barrier in DMPA users. A) The DEGs (FDR adj. p <0.05) identified between DMPA users and controls were analyzed for their enrichment in molecular pathways by testing against the databases GO, KEGG and Wiki. The most significant upregulated (red) and downregulated (blue) pathways identified are sorted by p-value. The -log_10_ (p-value) is shown on the x-axis, the dotted vertical line represents p = 0.01. B) Transcription factors upregulated (red) and downregulated (blue) based on DEGs were identified using the TRRUST database. DEGs: differentially expressed genes. FDR: false discovery rate.

To more formally identify the biological pathways most strongly associated with DMPA use, enrichment of the identified DEGs in molecular pathways was analyzed in reference to the Gene Ontology (GO), Kyoto Encyclopedia of Genes and Genomes (KEGG), and WikiPathways databases. Most significantly, the results of these combined analyses confirmed a DMPA-associated upregulation of genes involved in immune regulation, including genes associated with cytokine-mediated signaling, neutrophil-mediated immunity, and T- and B-cell receptor signaling pathways (Fig 3A and S3–S5 Tables). Pathways that were significantly downregulated in DMPA users included those related to the epithelial barrier, such as skin development and keratinocyte/epidermal cell differentiation. Overall, these data indicate that DMPA use correlates with pathways involved in immune regulation and epithelial barrier structure and function.

### Transcription factor analysis reveals DMPA-associated effects on immune regulation and epithelial cell differentiation

To enhance understanding of the regulatory networks affecting the expression of the DEGs, a transcription factor analysis was performed using Transcriptional Regulatory Relationships Unraveled by Science-based Text mining (TRRUST) database. This analysis revealed DMPA-associated upregulation of the transcription factors RELA (*P* = 2.9E−11), also known as p65, and NFKB1 (*P* = 3.2E−10) (Fig 3B and S6 Table), which are members of the NFκB protein complex and involved in several of the immune activation and inflammation-related pathways (reviewed in [26]) as described in the above paragraph. Other upregulated transcription factors in the DMPA group included SP1 (*P* = 1.9E−05), involved in cell differentiation and immune responses [27], and GLI1 (*P* = 4.9E−04), involved in epidermal cell differentiation [28]. In the transcription factor analysis, expression of the transcriptional repressor HES1 (*P* = 6.9E−04) was downregulated in the DMPA group. We also observed a large degree of overlap in expression of the genes associated with the top-5 enriched transcription factors (S2A Fig). Overall, these data showed that DMPA use associated with NFκB-mediated transcriptional regulation, as well as with the expression of other transcription factors involved in immune regulation and epithelial cell differentiation.

### The cervicovaginal microbiome (*Lactobacillus* vs non-*Lactobacillus* dominant) has no interactional impact on the DMPA associated gene expression

**Fig 4:**
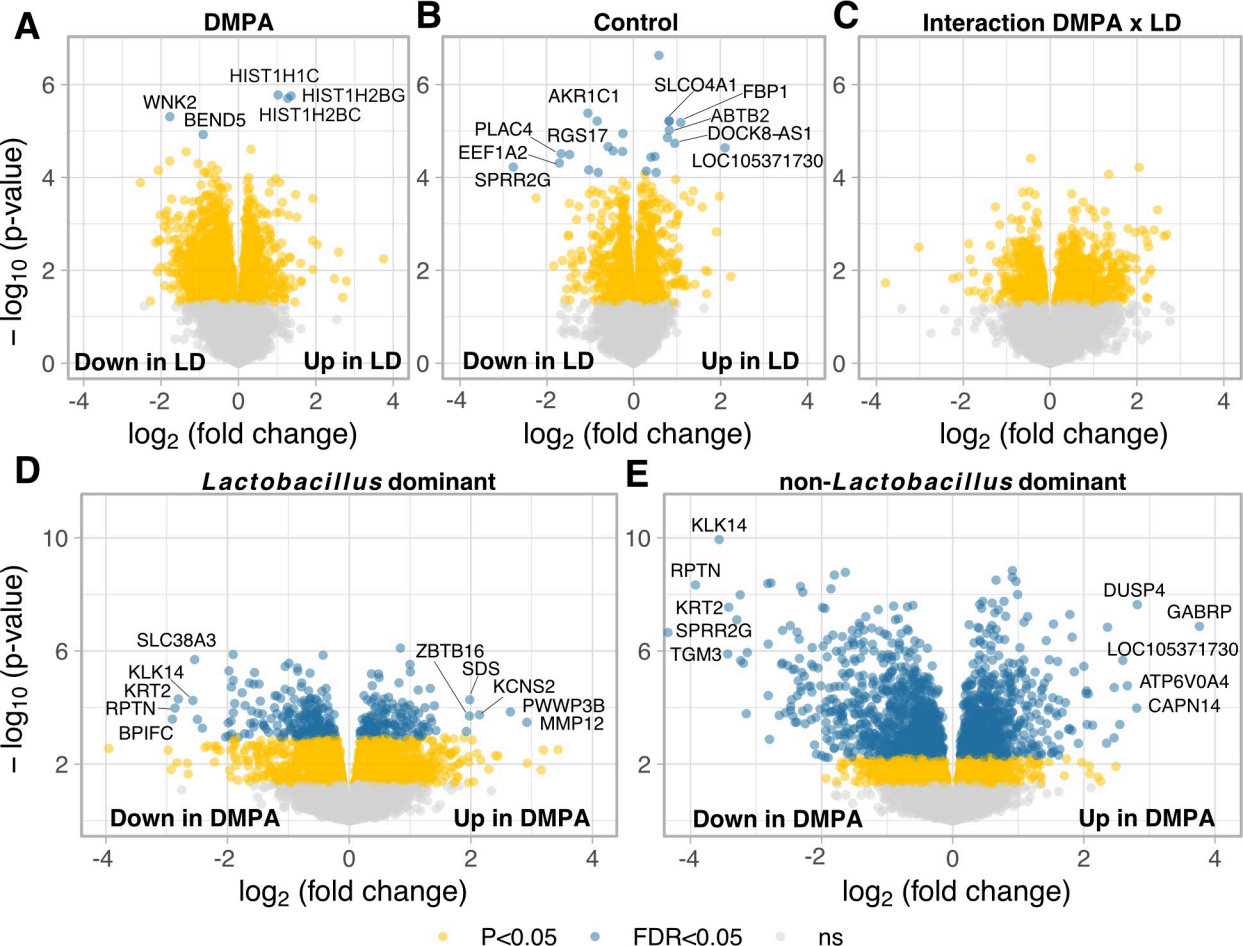
The cervicovaginal microbiome has no interactional impact on the DMPA associated gene expression. The impact of DMPA usage on gene expression profiles, based on a two-factor interaction model for DMPA and the microbiome (*Lactobacillus* dominant (LD) and non-*Lactobacillus* dominant (non-LD). A) Comparison of LD and non-LD groups, in the DMPA samples (18 non-LD vs 14 LD). B) Comparison of LD and non-LD groups, in the control samples (42 non-LD vs 22 LD). C) Testing for an interaction between DMPA and microbiome composition. D-E) Impact of DMPA use in LD (22 controls vs 14 DMPA) and non-LD samples (42 controls vs 18 DMPA), respectively. FDR-adjusted P-value <0.05 is considered significant (blue dots), non-adjusted P-value <0.05 shown in yellow. FDR: false discovery rate.

To investigate the effects of the cervicovaginal microbiota composition on the gene expression landscape upon DMPA usage, we investigated differential expression taking into consideration a two-factor interaction model for the microbiome and DMPA, in addition to the confounders age and time in sex work. This design is useful for detecting genes that respond differently to DMPA, given either a *Lactobacillus* dominated (LD) or a non-*Lactobacillus* dominated (non-LD) microbiome. The comparison of LD and non-LD samples showed a total of only 5 and 24 differentially expressed genes, for DMPA and control samples respectively (Fig 4A and 4B). The interaction term showed no significant differentially expressed genes (Fig 4C). Comparison of the LD and non-LD groups showed a much larger effect comprising 349 and 1800 significantly differentially expressed genes for the LD and non-LD samples respectively (Fig 4D and 4E). Of note, *RPTN*, *KRT2*, *KLK14*, *BPIFC* and *PWWP3B* remained among the top 15 DEGs sorted according to highest log fold change in both comparisons. Gene set enrichment analysis using the GO data base for samples dominated by a non-*Lactobacillus* microbiome revealed upregulation of pathways associated with neutrophil activation, and downregulation of pathways associated with epithelial development. Samples with a *Lactobacillus* dominated microbiome showed upregulated GO pathways associated with cytokine-mediated signaling, and downregulated pathways comparable to those observed for the non-*Lactobacillus* dominated samples (S7 Table). This demonstrates that DMPA usage is associated with a stronger effect on gene expression compared with the LD and non-LD groups, and that these groups showed no interactional effect on the host tissue transcriptome upon DMPA usage.

### DMPA use is associated with a thinner superficial layer of the ectocervical epithelium and a more apical distribution of CD4^+^ cells

**Fig 5:**
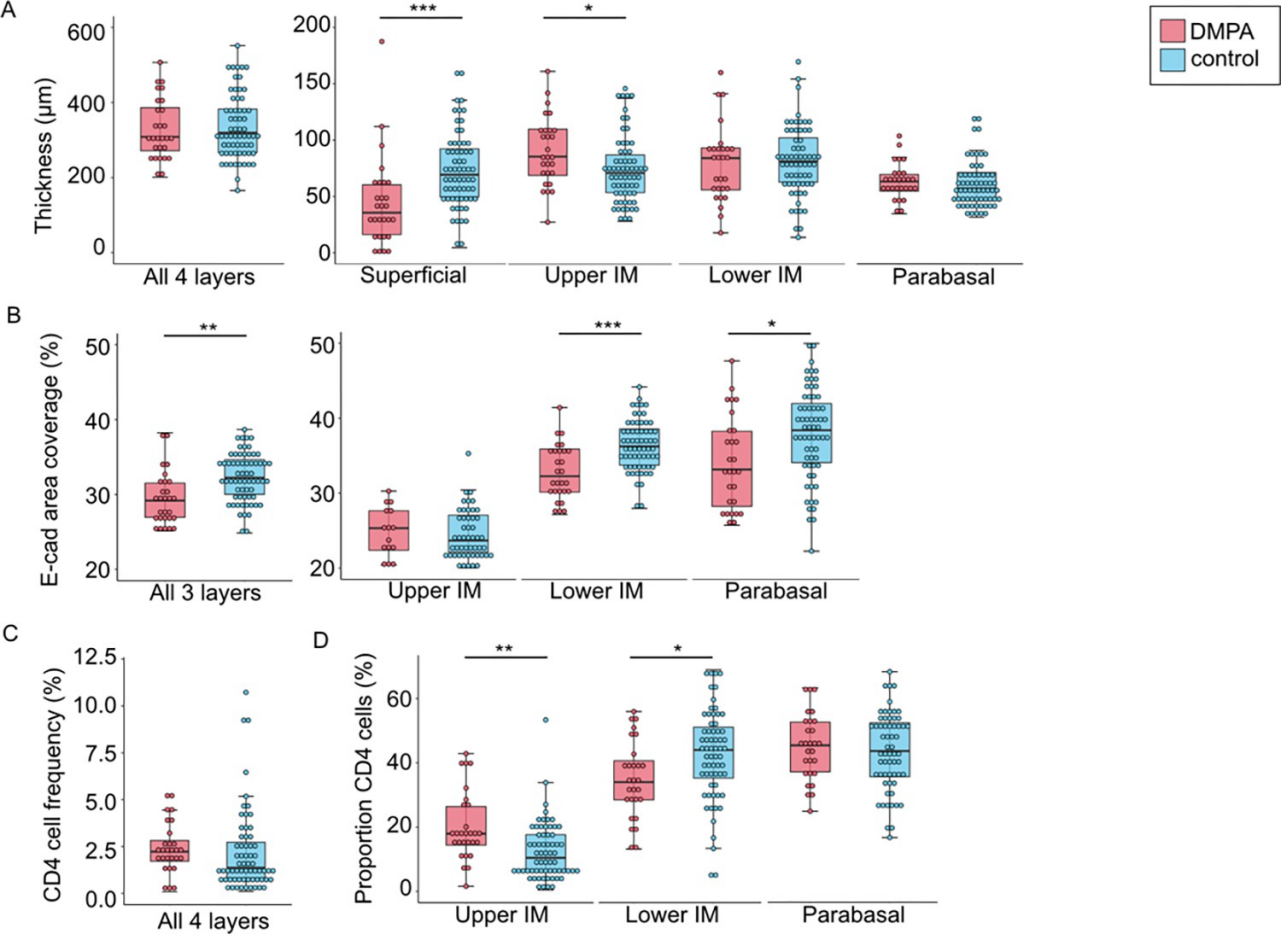
DMPA use is associated with a thinner superficial layer and a more apical distribution of CD4^+^-cells. The study groups were compared for markers of epithelial cell integrity and CD4^+^ cell distribution. A) Thickness in μm of the total epithelium and of each of the four individual layers (superficial, upper IM, lower IM, and parabasal) of the ectocervical epithelium. B) The percentage of E-cadherin area coverage (% of E-cadherin net area out of the total epithelial area that express E-cadherin); the upper- and lower IM, as well as the parabasal layer. C) The percentage of CD4^+^ cell frequency (% of positively stained CD4^+^ area out of total epithelial tissue area. D) The distribution of all CD4^+^ cells in the different layers in the epithelium is shown as the proportion of % CD4^+^ cells present in each of the individual layer; upper IM, lower IM and parabasal layers. No CD4^+^ cells were seen in the superficial layer. Samples from the DMPA group are shown in pink, and from the control group in light blue. Boxplots represent median, IQR, range within 1.5 x IQR (whiskers), outliers represented as dots. Graphs show Mann Whitney U comparisons, p-values were FDR-adjusted by Benjamini-Hochberg test. * FDR adj p<0.05; ** FDR adj p<0.01, *** FDR adj p<0.001. IM: intermediate. IQR: interquartile range. FDR: false discovery rate.

To complement our transcriptional profiling data and better understand the effect of DMPA at the protein and cellular level, we performed *in situ* staining combined with digital image analysis. Staining for the epithelial junction protein E-cadherin, which was not differentially expressed between the DMPA and control groups at the gene level (FDR adj. p-value > 0.05), formed the basis for identifying four different epithelial layers: The superficial layer (E-cadherin negative), the upper intermediate layer (irregular E-cadherin staining), the lower intermediate layer (intact net-like E-cadherin staining), and the parabasal layer (high intensity nuclear staining). We have previously analyzed these parameters in a sub-set (n = 60) of these samples [13]. For the present study we had access to 32 additional control samples (total number of samples: DMPA group n = 28, control group n = 64). While there was no significant difference in total epithelial thickness between the groups, women using DMPA had a significantly thinner superficial layer (median: 36 μm *vs*. 69 μm; FDR-adj. p = 0.001) and a significantly thicker upper IM layer (corresponding to a thicker layer of irregular E-cadherin staining) than controls (median: 86 μm *vs*. 71 μm; FDR-adj. p = 0.04) (Fig 5A and S8 Table). Tissue from DMPA users also displayed a significantly reduced E-cadherin area coverage than controls (median: 29% *vs*. 32%; FDR-adj. p = 0.002), as measured in the three lower epithelial layers (Fig 5B). No significant difference in the mean fluorescence intensity (MFI) of the E-cadherin staining was seen in the epithelium.

We further found that DMPA users displayed a significantly higher proportion of CD4^+^ cells in the upper IM layer relative to controls (median: 18% *vs*. 10%, respectively; FDR-adj. p = 0.004), and a significantly lower proportion in the lower IM layer than controls (median: 34% *vs*. 44%, respectively; FDR-adj. p = 0.03) (Fig 5C). The average distance between the most apical layer of the epithelium and CD4^+^ cells was however similar in the two groups. Our data thus indicate that DMPA users, compared with controls, have a less intact ectocervical epithelium as defined by a thinner superficial layer, larger areas with disrupted E-cadherin structures and more superficially located CD4^+^ cells.

### DMPA use is associated with decreased desmoglein-1 and claudin-1 expression

In addition to E-cadherin, members of desmosomal and claudin protein families are widely expressed in the human cervical epithelium and contribute to epithelial integrity. Our RNA-seq data revealed that several genes associated with cell-cell adhesion, including desmosome function (*DSG1*, *DSC1*, *DSC2*, *PKP1*, *PKP3*, *JUP*, *DSP*), were downregulated in the DMPA group (S2 Table). Among these, suppressed expression of *DSG1* has been associated with DMPA use also in other human and animal models [15,16,22,29,30]. The *DSG1*-encoded protein desmoglein-1 (*DSG1*: DMPA vs controls, top-15 downregulated genes, Table 2) and the *CLDN1*-encoded protein claudin-1 (*CLDN1*: DMPA vs controls, no significant differential expression at the gene level, FDR adj. *P*>0.05) were selected for visualization in ectocervical tissue samples. Furthermore, quantitative expression of these proteins in genital tissues of DMPA users has not previously been assessed in such a large human cohort. Due to limitations in sample availability (n = 3 missing from DMPA group) and quality (n = 2 from DMPA group, n = 9 from control group), imaging analysis was performed on 27 samples from the DMPA group (all overlapping with full cohort used for RNA-sequencing) and 56 samples from the control group (55 overlapping and one additional sample) (S9 Table).

**Fig 6:**
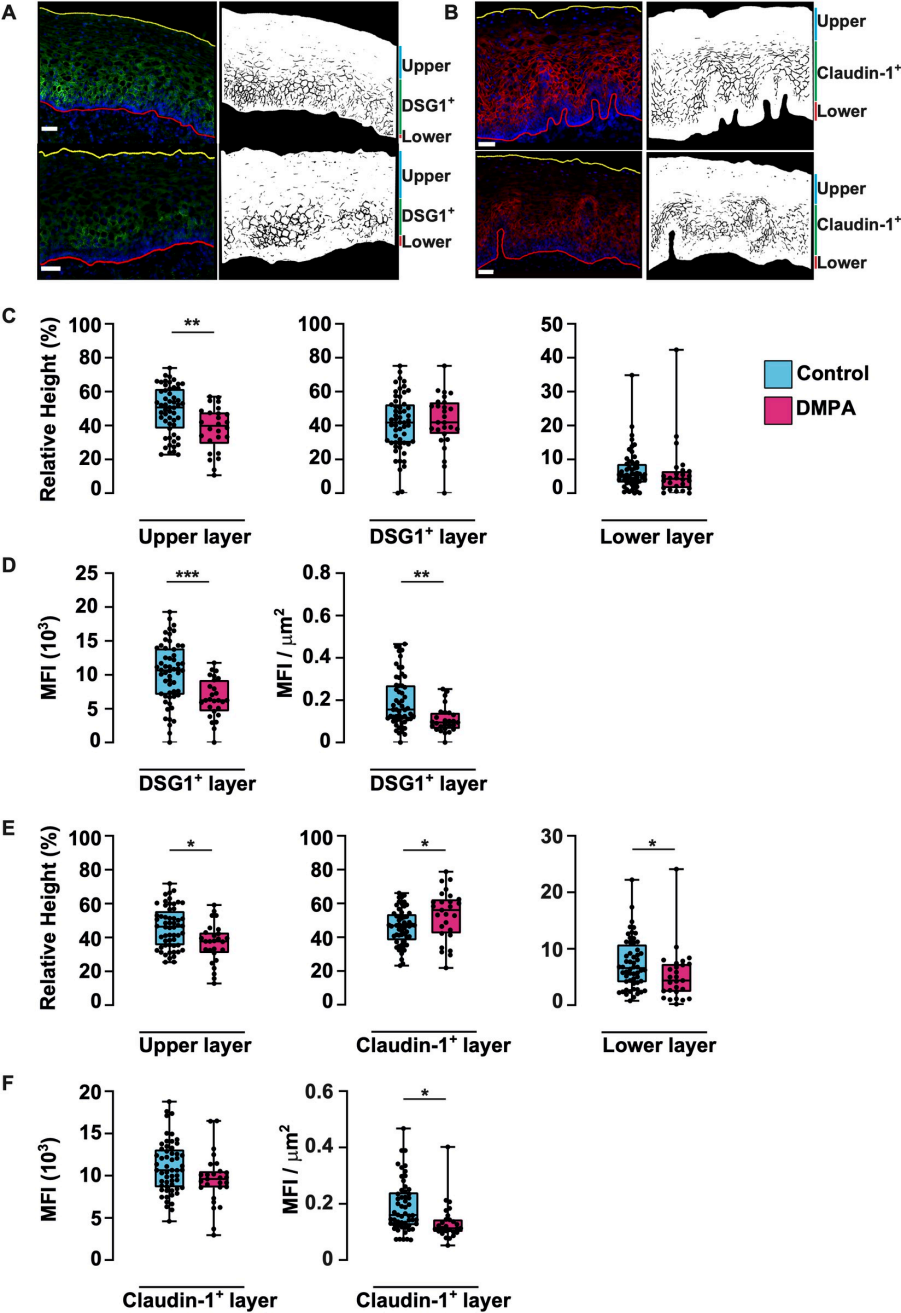
*In situ* image analysis shows decreased protein expression of desmoglein-1 and claudin-1 in the DMPA group. Immunofluorescence images of ectocervical tissue sections, and their corresponding digitalized images stained for desmoglein-1 in green **(A)** and claudin-1 in red **(B)**. All nuclei are stained with DAPI (blue). The immunofluorescence images are selected to represent those study subjects that displayed median values from the corresponding desmoglein-1 and claudin-1 MFI analysis in their respective study group. Images representing the control groups are shown in the upper row and DMPA in the lower row. The apical border (towards the vaginal lumen) is depicted in yellow and the basal line in red. In the digitalized images, the full epithelial area is shown in white and the desmoglein-1^+^ or the claudin-1^+^ staining in black. Box plots showing the relative height (%) for the three individual layers based on the desmoglein-1 staining **(C),** the MFI of the desmoglein-1^+^ layer including the MFI/ μm^2^ **(D)** as well as the relative height (%) based on claudin-1 staining **(E)** and the MFI of the claudin-1^+^ layer including the MFI/μm^2^ **(F)**. Control group (n = 56); turquoise, DMPA group (n = 27); pink. One individual from each study group was excluded due to technical issues. Box plots indicate medians and IQR and whiskers show full range. *p<0.05, ** p< 0.01, *** p <0.001, by Mann-Whitney *U* comparison, p-values FDR-adjusted by Benjamini-Hochberg test. MFI: mean fluorescence intensity. IQR: interquartile range. FDR: False Discovery Rate.

The ectocervical epithelium was first segmented into three distinct layers based on the spatial localization of the desmoglein-1^+^ or claudin-1^+^ staining (Fig 6A and 6B). The layer containing the staining of desmoglein-1 or claudin-1 was located between the apical and the lower layer of the epithelium. The two latter layers were segmented based on a lack of desmoglein-1 or claudin-1 expression. The apical layer is located towards the vaginal lumen and the lower layer is located above the basal membrane that separates the epithelium from the underlying submucosal/stromal compartment.

The total tissue area analyzed was similar between the DMPA and control groups (median: 166 x10^3^ μm^2^ vs. 176 x10^3^ μm^2^) (S3B Fig and S10 Table). The total height of the ectocervical epithelium was also similar in the DMPA and control groups (median: 300 μm vs. 297 μm), and no significant differences were seen in the height of the individual layers for desmoglein-1 (S3A and S3C Fig). However, analysis of the relative height of the three individual layers for desmoglein-1 revealed a significantly thinner upper layer in the DMPA group compared with the control group (median: 40% vs. 51%, p = 0.007), while no differences could be observed in the desmoglein-1^+^ layer (median: 42% vs. 42%) or the lower layer (median: 4% vs. 5%) (Fig 6C). The DMPA group had a significantly lower MFI in the desmoglein-1^+^ layer compared to the control group (median: 6x10^3^ vs. 10x10^3^, p = 0.001). This was also seen when adjusting the MFI for area of the segmented desmoglein-1^+^ layer, i.e. assessing MFI/μm^2^ (median: 0.1 vs., 0.16, p = 0.002). (Fig 6D).

Next, the claudin-1 staining was assessed. No significant differences in the height of the upper or claudin-1^+^ layers were seen, while a significantly thinner lower layer in the DMPA group compared with controls was detected (S3D Fig). Comparison of the relative height of the three individual layers revealed that the DMPA group, compared with the control group, had a significantly thinner upper layer (median: 38% vs. 46%, p = 0.02), a significantly thicker claudin-1^+^ layer (median: 56% vs. 47%, p = 0.04) and significantly thinner lower layer (median: 4% vs. 7%, p = 0.04) (Fig 6E). Moreover, the DMPA group compared with the control group trended towards significantly lower MFI of claudin-1 (median: 9.5x10^3^ vs. 10.5x10^3^, p = 0.09) and when adjusting the MFI for area of the segmented claudin-1 layer, the DMPA group had a significantly lower MFI/μm^2^ compared to the control group (median: 0.11 vs. 0.16, p = 0.01) (Fig 6F).

To explore the effect of the potential confounders age and time engaged in sex work on desmoglein-1 and claudin-1 expression, correlation analysis was performed, showing that neither of these factors affected the MFI of desmoglein-1 and claudin-1 within the respective study group (P>0.05). The high variability seen for some of the measurements in the control group could possibly be attributable to the variability in estradiol levels. This was tested for MFI levels of desmoglein-1 and claudin-1 in both study groups and thereby a significant correlation between the MFI of desmoglein-1 in the control group was revealed (P = 0.006) (S4 Fig). Collectively, these data indicate that the DMPA users displayed a thinner superficial epithelial layer and lower expression of both desmoglein-1 and claudin-1 than the control group, indicative of less robust epithelial integrity.

### Protein profiling reveals changes in levels of enzymes involved in protease inhibition

**Fig 7:**
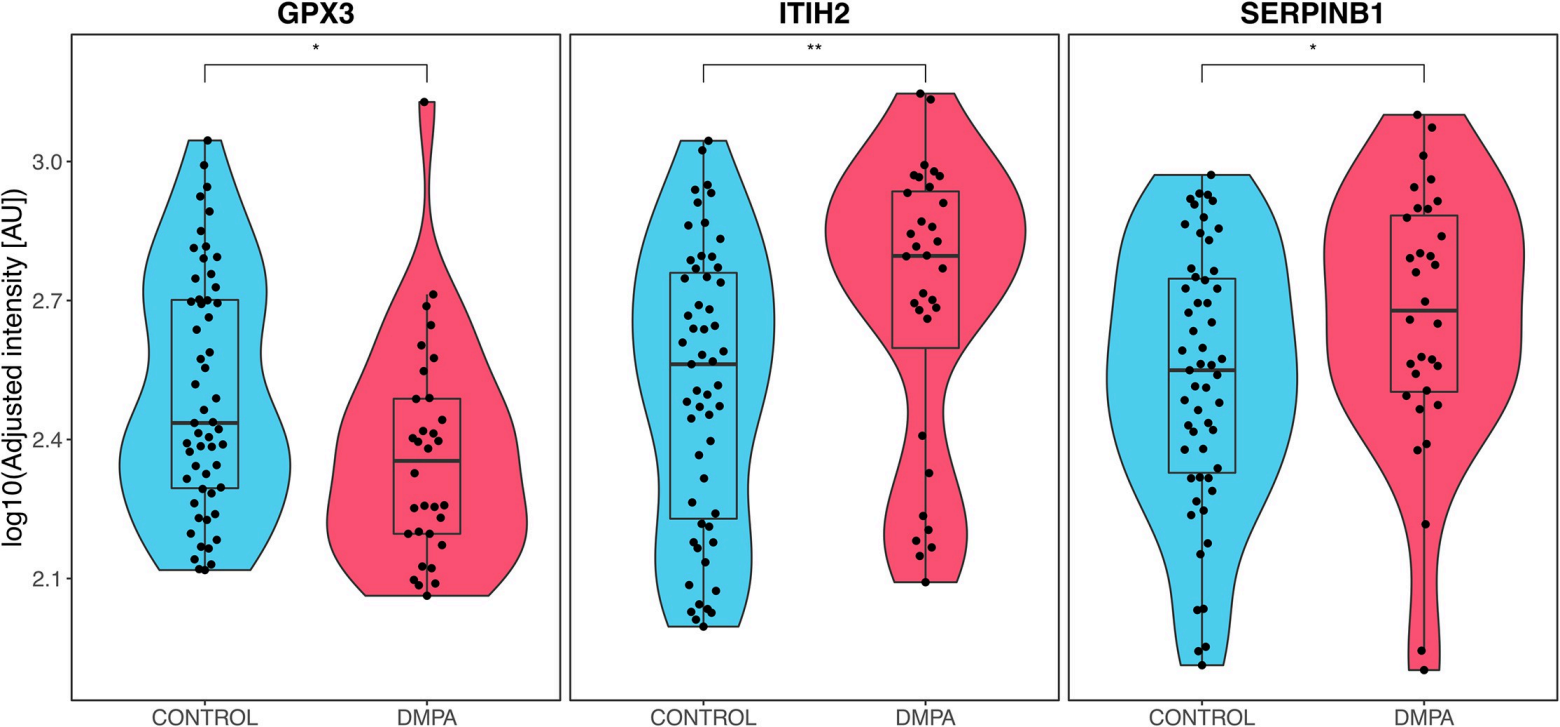
Proteins with altered profiles in CVL as compared between the DMPA vs. control groups. Based on analysis of protein levels in CVL from DMPA users (n = 32) and controls (n = 55), three proteins (GPX3, ITIH2 and SERPINB1) were identified as significantly different between these groups. Boxes indicate medians and IQR and whiskers and violin show full range. * p< 0.05, ** p < 0.01, by Mann-Whitney U test, not adjusted for potential confounders. CVL: cervicovaginal lavage. AU: arbitrary units. IQR: Interquartile range.

In order to complement the gene expression and imaging analyses with data regarding protein expression in genital secretions, the levels of 74 proteins in matching CVL samples were analyzed using an antibody-based suspension bead array (S11 and S12 Tables). Thirty-two samples from the DMPA group (all overlapping from full cohort used for RNA-sequencing) and 55 samples (53 overlapping samples overlapping and 2 additional) from the control group were analyzed (S13 Table). The levels of three proteins differed significantly (p<0.05) between the DMPA and control groups: the extracellular antioxidant enzyme glutathione peroxidase 3 (GPX3), inter-alpha-trypsin inhibitor heavy chain 2 (ITIH2), and serpin family B member 1 (SERPINB1), a serine protease inhibitor (Figs 7 and S5 and Table 3). We assessed the effect of age and duration of sex work on protein levels by utilizing generalized linear models. ITIH2 and SERPINB1 remained significant between controls and DMPA users when controlling for age and time in sex work, but GPX3 did not (S14 Table). Overall, these data show that DMPA use correlates with the expression of selected proteins involved in protease-inhibition in genital fluids.

**Table 3:** Proteins present at altered levels in women using DMPA contraceptives compared to controls.

HGNC ID	Protein name	Uniprot ID	Antibody	p-value
GPX3	glutathione peroxidase 3	P22352	HPA062579	0.016
ITIH2	inter-alpha-trypsin inhibitor heavy chain 2	P19823	HPA062964	0.003
SERPINB1	serpin family B member 1	P30740	HPA018871	0.037

P-values calculated using Mann-Whitney *U* test. HGNC: HUGO Gene Nomenclature Committee.

Among the three proteins (ITIH2, SERPIN1B, GPX3) that showed significantly altered levels between the DMPA and control groups, only GPX3 corresponded to a significantly different gene expression (Log2 FC -0.56; FDR-adjusted p-value 1.12E-03) (S2 Table). Furthermore, we evaluated whether differences in protein levels between the DMPA and control groups were influenced by the cervicovaginal microbiome composition. Only the ITIH2 protein levels differed significantly between the DMPA and control groups in samples with non-*Lactobacillus* dominance (adj. p < 0.01), whereas none of the proteins differed significantly between the study groups in the *Lactobacillus* dominant samples (adj. p > 0.05) (S15 Table). There was no interactional effect on the protein expression between these microbiome groups and DMPA use.

## Discussion

Here, we present the results of a comprehensive characterization of the effects of long-term DMPA use on the epithelial barrier of the ectocervical mucosa in women living in an area of high HIV prevalence. Our findings reveal an association between DMPA use and significant disruption of the epithelial barrier structure, as indicated by a downregulation of genes involved in maintaining epithelial barrier integrity. We also observed enhanced immune regulation and NFκB-mediated signaling associated with DMPA use. Quantitative analysis of digitalized images of tissue samples confirmed decreased expression of selected epithelial junction proteins, including E-cadherin, desmoglein-1 and claudin-1, as well as thinning of the upper epithelial layer in DMPA users. Based on these data, we propose that these DMPA-associated impairments increase the permeability of the epithelial barrier to incoming pathogens, including HIV.

Notably, we observed substantial downregulation of multiple genes important for maintaining epithelial barrier function in DMPA users, including *RPTN*, *LOR*, *SPRR2G/2B*, *TGM3* [31,32], and various keratin genes (*KRT1/2/10/76*) that encode intermediate filament proteins expressed in the stratified squamous epithelium [33]. Further highlighting the association with the epithelium in our dataset, gene set enrichment analyses indicated a downregulation of genes associated with development of the epithelium in the DMPA group. Of note, although DMPA use was correlated with several downregulated genes and pathways corresponding to epithelial barrier integrity, this was a selective process as other genes with similar function could be upregulated. Interestingly, the transcription factor GLI1, which was upregulated in the DMPA group, affects epidermal cell differentiation via its effect on keratinocytes [28], thus potentially affecting epithelial barrier function. Overall, the functions of the above-mentioned genes in the human skin epidermis have been well characterized [31–33], but their role in maintaining the mucosal epithelium of the female genital tract remains largely unknown. The skin epidermis and ectocervical/vaginal epithelium are structurally similar except for a keratinized layer that is not present in the cervicovaginal epithelium [34]. However, our data suggest that the above-mentioned genes/proteins, which play well-defined roles in the skin epidermis, also play roles in the female genital mucosa.

The results of our gene expression analyses using tissue samples from sexually active long-term DMPA living in an area of high HIV prevalence are strikingly similar to those from a longitudinal North American study of women at lower risk of acquiring HIV, in which microarray-based whole-genome transcriptome profiling was performed on ectocervical biopsies prior to initiation and after 6 weeks of DMPA use [16]. Despite substantial differences in study design compared with our present study, those authors also observed DMPA-associated downregulation of genes and pathways involved in epithelial barrier formation, including *RPTN*, *LOR*, *TMG3*, and several genes within the *SPRR* and *KRT* families. Taken together, these studies representing women with different sociodemographic and clinical characteristics suggest that DMPA is associated with an impaired epithelial barrier in the genital mucosa.

Resistance against pathogens, including HIV, during vaginal sexual intercourse is dependent on several factors such as antimicrobial compounds in genital secretions, the mucus layer and the thickness and integrity of the multi-stratified epithelial layer. Weakening of the multi-stratified epithelial barrier, including thinning of the epithelial layer, has been associated with increased susceptibility to HIV/simian immunodeficiency virus (SIV) infection in humanized mice [35] and non-human primates [36] following intravaginal exposure. We have previously demonstrated a thinner superficial/apical epithelial layer in DMPA users based on immunostaining for E-cadherin [13], as well as in users of progestin-based intrauterine devices [23]. These studies suggest that epithelial HIV target cells are more accessible to virus particles at the vaginal lumen [13]. While many studies have shown that DMPA does not significantly affect cervicovaginal epithelial thickness [13,37–39], our imaging technique allowed specific measurements of individual epithelial layers in the samples. This revealed thinning of the upper epithelial layer by staining for epithelial junction proteins. The epithelial integrity was further proven to be more generally impaired in DMPA users by identifying reduced expression of the selected epithelial junction proteins E-cadherin, desmoglein-1 and claudin-1. In addition to the epithelial robustness, the distribution of CD4^+^ cells as a proxy for HIV-target cells was also measured. We found that although total numbers of CD4^+^ cells did not differ in the epithelium from DMPA users and controls, these cells were more superficially located in the DMPA group and thus are more accessible to virus particles localized at the border of the vaginal lumen.

Interestingly, transcriptional profiling revealed decreased gene expression of several desmosomal cadherins and overexpression of the desmoglein-1-degrading protease *CAPN14* [25] in tissues from DMPA users, indicating a possible mechanism for impairment of the desmosome function. Desmosomes structurally contribute to cell-cell adhesions between keratinocytes in the epithelium, and in concert with other epithelial junction proteins, contribute to epithelial barrier function [40]. Desmosomal cadherins (desmogleins and desmocollins) are important desmosome-associated transmembrane proteins that, by binding to JUP (plakoglobin), DSP (desmoplakin) and PKP (plakophilin), connect to intermediate intracellular filament proteins such as keratins [40]. It was therefore intriguing to observe downregulation of the genes encoding both desmosomal cadherins, JUP, DSP and PKPs, as well as genes encoding several keratins in our sample set representing DMPA users. Among the top-15 most downregulated genes between the DMPA and control groups, four were keratins (*KRT1*,*2*,*10* and *76*). In addition, smaller studies assessing genital tissues from mice [15,22,29], humanized mice [41], non-human primates [30], and humans [15,16] have reported decreased DSG1 gene or protein expression with DMPA treatment. This decreased DSG1 expression was associated with increased permeability of the genital tissues [15,22,29,30,35,41] and enhanced susceptibility to intravaginal infection with herpes simplex virus–2 [15,22,29] and HIV [29,35]. Combined with the results derived from our imaging analysis, these gene expression data significantly extend those of previous studies in humans and animal models by demonstrating DMPA associated downregulation of several key factors involved in mediating epithelial cell-cell adhesion function at both gene and protein levels.

Gene set enrichment analysis suggested that DMPA users have increased immune regulation relative to non–DMPA users, as defined by changes in the expression of genes associated with the biological processes cytokine-mediated signaling and T- and B-cell receptor signaling. The observed immune regulation changes in DMPA users were further supported by transcription factor analyses, which revealed upregulation of members of the NFκB-protein complex and SP1 (involved in immune regulation) [26,27] and downregulation of the inflammatory suppressor HES1 [42]. Specifically, two of the most overexpressed genes in the DMPA group, the pro-inflammatory cytokines *CXCL6* and *CXCL1*, exhibit neutrophil chemotactic activity [43,44] which could indicate an inflamed genital mucosa in this group. Several genes and pathways related to neutrophil activation were upregulated in the DMPA users. Unfortunately, we could not enumerate neutrophils in the corresponding tissue sections due to lack of material.

Genital inflammation, characterized by upregulation of several pro-inflammatory cytokines, is a well-known risk factor for HIV infection [45]. Previous studies have reported that DMPA/MPA (MPA, the metabolically active progestin in DMPA) is associated with immune regulation and inflammation in the female genital tract (reviewed in [2] and [14]), including both general immunosuppression and a more pro-inflammatory state. Immune activation and inflammation can be affected by exogenous and endogenous factors including vaginal microbiome composition, which has been associated with increased HIV acquisition [4]. Interestingly, Noël-Romas *et al*. recently demonstrated that higher MPA levels were associated with enhanced genital inflammation in women with a *Lactobacillus*-dominant vaginal microbiome composition, whereas exacerbated genital inflammation was not observed in women with a non–*Lactobacillus*-dominant vaginal microbiome [46]. These results are indicative of an important interplay between the vaginal microbiome, DMPA, and genital inflammation and suggest that the effect of DMPA on the mucosa is context dependent [17,18,46]. However, no significant differences in the cervicovaginal microbiome composition were observed between the DMPA and control groups in our study. Furthermore, we here showed that DMPA usage associated to a significantly stronger effect than the cervicovaginal microbiome composition (as defined by *Lactobacillus* versus non-*Lactobacillus* dominated groups) on the cervical tissue gene expression. In addition, there was no overall interactional effect on gene or protein expression levels of these two microbiome groups and DMPA usage. Collectively, our data indicate enhanced immune regulation in DMPA users; however, the net effect of such regulation—and how it affects HIV susceptibility—remains to be determined.

The active progestin component of DMPA can bind to both progesterone and glucocorticoid intracellular steroid receptors [47] resulting in both direct and indirect regulation of transcriptional activation of hundreds of genes involved in a broad range of immune functions. Other progestins used for contraception that more specifically bind to the progesterone receptor can also cause immune activation and decrease epithelial integrity in animal models [2]. Another important aspect of DMPA’s direct or indirect effect on gene regulation is suppression of natural systemic E2 levels [24] which was confirmed in our data set. Thus, in addition to the progestin effect per se, the DMPA-induced hypoestrogenic state could possibly affect gene expression profiles in DMPA users. Indeed, such hypoestrogenism may contribute to increased HIV susceptibility in DMPA users (reviewed in [48]). Furthermore, treatment with estrogen is associated with protection against infection by SIV [49] and cell-associated HIV [41] after intravaginal challenge in non-human primates [49] and humanized mice [41]. Several transcription factors identified in our data set, such as the NFκB family and SP1, interact with E2 [50,51]. Interestingly, a few samples in the DMPA group exhibited high E2 levels, and the transcriptional profile of those samples was similar to that of control samples (with higher E2 levels), again highlighting the importance of E2 in transcriptional regulation. Collectively, these results indicate that the direct effect of MPA and hypoestrogenism in DMPA users could possibly affect transcriptional regulation and genital barrier structure and function. However, the relative effect of hypoestrogenism in DMPA users is difficult to interpret and thus warrants further research due to the complex actions of sex hormones and sex hormone receptors, including the cell- and gene-specific nature of steroid receptors, cross-talk between signaling pathways, and the effects of co-repressors and co-activators on transcription.

To complement the tissue transcriptomics and *in situ* imaging data set, a pre-selected protein panel was analyzed using matching genital secretions from the participants. Interestingly, levels of SERPINB1, a protease inhibitor that protects against neutrophil serine protease–mediated inflammatory damage at mucosal surfaces [52] were altered in the DMPA group as compared to the control group. Furthermore, increased genital expression of SERPINB1 and other serpin family members is reportedly associated with relative resistance to HIV [53], highlighting its importance at the genital mucosal barrier. Levels of ITIH2, which contributes to extracellular matrix construction and stabilization [54], were also altered in the DMPA group. Collectively, these results support our observation that DMPA use correlates with factors important in the mucosal immune system and mucosal barrier function.

Our study has several limitations, including inability to confirm self-reported DMPA use with MPA concentration measurements. However, the suppression of plasma E2 and P4 levels was consistent with previous studies of long-term DMPA users [24]. Another limitation of our study is that the control group targeted participants who were in the follicular phase of the menstrual cycle based on self-reported days since onset of last menses; however, for logistical reasons, this could not be fully achieved. A concern is also that the DMPA users may be different in behaviors and other factors as they, unlike the control group, do not need to use condoms for contraception. A major confounder of observational DMPA studies could thus be the influence of unprotected sex on the genital epithelium with significant impact on mucosal immune responses [55]. This influence was here reduced as all women consented to refrain from vaginal intercourse two weeks prior to sampling. They also consented to be repeatedly tested for presence of PSA as a measure of adherence to the study protocol. As a possible consequence of these measures, there was no statistically significant difference between presence of PSA in the two study groups. To further reduce possible confounding effects on the data, the parameters ‘age’ and ‘time in sex work’ were adjusted for in the statistical analyses of the experimental data.

In conclusion, our transcriptional profiling and imaging data reveal that women using DMPA had decreased epithelial barrier integrity and increased immune regulation in the ectocervical mucosa compared to non-hormonal contraceptive users. Our results also suggest that the downregulation of genes essential for cell-cell adhesion, including several desmosomes, and keratinization processes, combined with the thinning of the upper epithelial layer could contribute to the diminishment of ectocervical tissue integrity. Such epithelial barrier impairments could increase permeability to incoming pathogens, including HIV.

## Material and methods

### Ethics statement

The study was approved by the ethical review boards at the University of Manitoba, the Kenyatta National Hospital/University of Nairobi, and the Regional Ethical Review Board in Stockholm. Each participant provided written informed consent.

### Study population

Samples for this analysis were selected from samples of a larger longitudinal study performed within the Pumwani Sex Worker Cohort, Nairobi, Kenya, as previously described [56]. Briefly, inclusion criteria at enrollment included the following: age 18–50 years; actively engaging in sex work (self-reported); not pregnant/breastfeeding; not menopausal; no prior hysterectomy; and negative for HIV, *Chlamydia trachomatis*, *Neisseria gonorrhoeae*, and syphilis infection. HIV serology was performed at enrollment, approximately 6 weeks after the study visit, and at 3–6 months after study completion using an HIV rapid test (Determine, Inverness Medical, Shinjuku, Japan). Urine was collected at enrollment and at the study visit (2–4 weeks after enrollment) for PCR screening for *N*. *gonorrhoeae* and *C*. *trachomatis* using a Roche AMPLICOR kit (Pleasanton, NJ, USA). BV was diagnosed using the Nugent score, with scores of 0–3, 4–6, and 7–10 indicating BV-negative, BV-intermediate, and BV-positive, respectively.

For the present study, we selected HIV-seronegative women who had used DMPA for at least 6 months (DMPA group) and regularly cycling non-hormonal contraceptive users (control group). For DMPA users, sampling was aimed at 4–8 weeks after the last DMPA injection. For the control group, sampling was targeted to the follicular phase of the menstrual cycle, based on self-reported days since last menses. Differences in continuous variables between groups (DMPA group vs. control group) were assessed using the Mann-Whitney *U* test, and differences in categorical variables were analyzed using the Pearson’s chi-squared and Fischer’s exact test using R software [57]. P<0.05 were considered significant. The RNA-seq data from the control biopsies (n = 64), but not from the DMPA group (n = 32), has been published previously in a different context [58].

### Sample collection and plasma hormone analysis

CVL samples were collected using a sterile transfer pipette to wash the endocervix with 2 mL of sterile phosphate-buffered saline and then aspirating the lavage from the posterior fornix, as previously described [56]. CVL fluid was centrifuged, and the supernatant was aliquoted; the pellet containing cellular debris, hereafter referred to as the “CVL pellet,” was resuspended in RNAlater. CVL and CVL pellets were immediately frozen at −80°C until time of analysis. Two 3-mm^2^ ectocervical biopsies from the superior part of the ectocervix were collected by a trained gynecologist. One biopsy sample was immediately snap-frozen in liquid nitrogen and stored at −80°C; the other biopsy sample was immediately placed in RNAlater and stored at −80°C. The women were counseled not to have sex for 2 weeks to enable healing. Measures such as text messages, on-site PSA detection and monetary compensation for loss of income have previously been evaluated and encouraged participants to adhere to the study protocol [56]. Presence of PSA was tested by loading a portion of unprocessed CVL to the immunochromatographic test strip cassette. The result was read according to the manufacturer’s instructions (Seratec PSA Semiquant, Göttingen, Germany) [56]. All women were examined at the clinic 3–5 days post-biopsy to ensure healing and compliance with abstinence instructions. Blood samples were collected by venipuncture using heparin as an anticoagulant, and plasma was isolated using a Ficoll density gradient. Plasma E2 and P4 levels were measured using electrochemiluminescence immunoassays (Roche Diagnostics) at the accredited Karolinska University Laboratory. The LLDs for E2 and P4 were 22 pg/mL and 0.05 ng/mL, respectively. Values <22 pg/mL (E2) and <0.05 ng/mL (P4) were reported as “below the LLD,” but for visual and statistical purposes, these values were assigned a value of 22 pg/mL and 0.05 ng/mL for E2 and P4, respectively.

### Sequencing of 16S ribosomal RNA (rRNA) gene to determine the cervicovaginal microbiome composition

Nucleic acids were extracted from CVL pellets, and the V4 region of the 16S rRNA gene was sequenced. The samples were then divided into five groups based on bacterial composition (L1: *L*.*crispatus/jensenii*, L2: *L*.*iners*, L3: *Gardnerella*, L4: High Diverse, L5: Other). Additional details are provided in the S1 Text.

### Preparation of tissues for library construction and RNA-seq analysis

Details regarding preparation of ectocervical tissues for RNA-seq analysis are provided in the S1 Text. Briefly, frozen ectocervical biopsy specimens stored in RNAlater were thawed, placed in RLT Plus Lysis Buffer (QIAGEN, Hilden, Germany), and homogenized using a TissueLyzer II machine (QIAGEN). RNA was isolated and purified using an AllPrep DNA/RNA Mini Kit (QIAGEN) and RNA isolated from all 96 ectocervical biopsy specimens exhibited good quality (RNA integrity number >8). TruSeq mRNA-Seq library prep kit (Illumina, San Diego, CA, USA) was used to purify mRNA and subsequently converted into cDNA libraries by using reverse transcriptase, random primers and DNA Polymerase I. Barcoded cDNA libraries were pooled and loaded onto reagent cartridges (Illumina) in order for sequencing in a NextSeq 550 (Illumina).

### Analysis of RNA-seq data and statistics

Base calling and demultiplexing of the Bcl files were performed using the Illumina bcl2fastq program. STAR was then used to index the human reference genome (hg38/GRCh38) and align the resulting fastq files. All samples generated >10 million reads and uniquely mapped reads were counted in annotated exons using featureCounts. Entrez gene annotations and reference genomes were obtained from University of California, Santa Cruz. The count table from featureCounts was then imported into R [57] Bioconductor for further analyses. In brief, read counts were imported into a DGEList object and normalized using the trimmed mean of M-values in Bioconductor with the EdgeR package. Genes with more than one count per million in more than three samples were retained for further analysis.

Analysis of differential gene expression between the two study groups (DMPA vs. controls) was performed with voomWithQualityWeights [59], followed by linear modeling using the limma package. Due to differences between the two study groups in terms of age and time engaged in sex work, these two parameters were corrected for in the differential gene expression analysis using an additive linear model including age and sex work duration as factors. Genes with FDR-adjusted *P*-values <0.05 were considered significant and classified as DEGs. Hierarchical clustering was performed on all DEGs, and the results were plotted as a heatmap using the ComplexHeatmap package. UMAP unsupervised dimensionality reduction analysis was performed using the normalized count per million of the DEGs from all subjects with the uwot package.

Details regarding gene set enrichment analysis are provided in the S1 Text. Briefly, the DEGs were subjected to gene set enrichment analysis against the GO [60], KEGG [61], and WikiPathways [62] databases. Similarly, transcription factor analysis was performed by searching the TRRUST database [63]. A transcription factor/protein interaction network was constructed and visualized with Igraph [64].

### Interaction analysis of the impact of the microbiome composition on the DMPA associated gene expression

A two-factor interaction model was explored to assess possible interactional effects of the microbiome composition and DMPA usage on host gene expression. Samples with microbiome compositions defined as groups L1 and L2 (Table 1) were combined into a *Lactobacillus* dominated group, whereas samples defined as groups L3, L4 and L5 were combined into a non-*Lactobacillus* dominated group. The factorial model also included the two confounders age and time in sex work and was performed with voomWithQualityWeights [59], followed by linear modeling using the limma package.

### *In situ* immunofluorescence staining and tissue imaging analysis of E-cadherin and CD4

*In situ* staining was performed to assess the protein expression of E-cadherin and CD4 in cryopreserved ectocervical biopsies (see S1 Text). Tissue imaging analysis was performed as previously described [13]. Briefly, using a Pannoramic MIDI II slide scanner (20× objective, 3DHISTECH Kft., Budapest, Hungary), each immunostained tissue section was scanned into digital images. Customized computerized image-based analysis workflows were used to assess the expression of these selected proteins. The E-cadherin staining formed a net-like structure around epithelial cells and was used to define four epithelial layers: The superficial layer lacking expression of E-cadherin, the upper IM layer defined by a broken net structure, the lower IM layer defined by an intact net structure and the parabasal layer with its high nucleus density. Three metrics were used to investigate epithelial barrier and integrity: 1) Total epithelial thickness, as well as the thickness of each of the four individual epithelial layers. 2) The percentage of E-cadherin area coverage was calculated by assessing the E-cadherin net area relative to the total epithelial area in the upper and lower IM layers and in the parabasal layer, as well as these three layers combined. 3) The MFI of the total E-cadherin positive staining in the respective layer.

A second digital image analysis workflow was previously developed by us [13] to assess the frequency and spatial localization of CD4^+^ cells in the ectocervical epithelium. Briefly, the percentage of positively stained area per total tissue area was used as proxy for the percentage of positive cells, and the following three measurements were assessed: 1) Frequency of CD4^+^cells per total tissue area in the total epithelium, as well as in each of the four individual layers; 2) Proportion of CD4^+^cells in each of the four individual layers; and 3) Average distance from CD4^+^cells to the apical border.

For the 18 measurements acquired by image analysis for the E-cadherin and CD4 stainings, a Mann-Whitney U test, followed by adjustment with FDR (Benjamini-Hochberg), was used for comparisons between groups. FDR-adjusted *P*-values <0.05 were considered significant. Statistical analyses were performed using R.

### *In situ* immunofluorescense staining and tissue imaging analysis of desmoglein-1 and claudin-1

*In situ* staining was performed to assess the protein expression of desmoglein-1 and claudin-1 in cryopreserved ectocervical biopsies (see S1 Text). All tissue sections were scanned into digital images using an ×20 objective on a Pannoramic 250 Flash Slide Scanner (3DHistech Ltd, Budapest, Hungary). Customized computerized image-based analysis workflows were used to assess the expression of these selected proteins. Briefly, the spatial distribution of the proteins was used to segment the ectocervical epithelium into three layers. The apical (upper layer) and the basal (lower layer) which contained no expression of the stained proteins, and a middle layer, where desmoglein-1 and claudin-1 were expressed. Three measurements were calculated: a) The total area and height of the epithelium, b) The total height of the three segmented layers, c) The relative height of the three segmented layers. The mean fluorescence intensity (MFI) including the MFI/μm^2^ of the desmoglein-1 and claudin-1 layers was also calculated. The differences in epithelial height and MFI between the study groups were assessed by Mann-Whitney *U* test and followed by a FDR (Benjamini-Hochberg) correction. Correlation analysis between MFI and the potential confounders age and time in sex work were assessed by Spearman’s correlation test. Statistical analyses were performed using GraphPad Prism version 6.0 and 9.1.0 (GraphPad Software, San Diego, CA, USA).

### Protein profiling on suspension bead arrays

To evaluate genital protein expression, antibody-based protein profiling was performed on CVL samples. Protein targets (n = 74) were selected from among a larger panel of proteins with verified presence in genital fluids and with known associations to HIV resistance and inflammation [65] and supplemented with protein targets identified in studies on the effect of sex hormones and inflammation on the FGT [11,16,66–69]. This panel was previously used in analyses of another sample set [58]. Details of the suspension bead array and data analysis can be found in the S1 Text, but briefly, antibodies from the Human Protein Atlas (www.proteinatlas.org) were immobilized onto color-coded beads and mixed with biotinylated CVL samples. Detection was enabled using a streptavidin-conjugated fluorophore and read out was performed in a Flexmap 3D instrument (Luminex Corp., Austin, TX, USA) where binding events were reported as fluorescence intensity (arbitrary units). Data were log10 transformed and normalized to diminish the effects of time delay during read out and reduce differences between plates. The Mann-Whitney *U* test was used to evaluate differences in protein levels, and generalized linear models were used to investigate the effects of potential confounders. Data processing and statistical analyses were performed using R, and p-values <0.05 were considered significant.

Potential differences in protein levels between the study groups were further evaluated taking the microbiome composition into account by doing sub-group analysis of the *Lactobacillus* dominant and non-*Lactobacillus* dominant groups (L1/L2 vs L3/L4/L5). Additionally, the interaction term between the microbiome groups and the effect of DMPA usage was tested. Linear models were fitted for each protein with the Limma package version 3.42 [70]. Age and time in sex work were included as blocking variables in the model. Differentially expressed proteins were considered significant at adjusted p-value < 0.05.

## Supporting information

S1 Fig Plasma hormone levels confirm suppressed estradiol and progesterone levels in DMPA users. A and B) Plasma levels of estradiol (E2) and progesterone (P4) in the control group (n = 64) and the DMPA group (n = 31). LLD was 22 pg/mL and 0.05 ng/mL for E2 and P4, respectively. Values <22 pg/mL (E2) and <0.05 ng/mL (P4) were reported as “below LLD”, but for visual and statistical purposes, these values were assigned a value of 22 pg/ml and 0.05 ng/ml for E2 and P4, respectively, shown here by the dotted line. C. Self-reported days since onset of last menstrual period, this parameter was only applicable for the control group. Data not available for 3 samples, resulting in a total of 61 controls. The longer horizontal bar is median; whiskers indicating full range. P-values calculated by Mann-Whitney U test. LLD: lower limit of detection.
(TIF)

S2 Fig Network of DEGs and associated transcription factors that are up and downregulated in DMPA users. A and B) Each node represents a DEG that is upregulated (A) and downregulated (B), respectively, in the DMPA group. The lines connect the gene to the transcription factor(s) (in boxes) that have been reported to regulate the expression of that particular gene. Transcription factors were identified by the TRRUST database. DEG: differentially expressed gene. TRRUST: Transcriptional Regulatory Relationships Unraveled by Science-based Text mining.
(TIF)

S3 Fig Expression of desmoglein-1 and claudin-1 in the ectocervical epithelium. Boxplots showing the height (A) and area (B) of the ectocervical epithelium as well as the height of the three individual layers based on desmoglein-1 staining (C), and based on claudin-1 staining (D). Control group (n = 56); turquoise, DMPA (n = 27); pink. One individual from each study group had non-detectable desmogelin-1 staining and could thus not be included in the measurements for the three individual epithelial layers. Boxplots indicate medians and IQR and whiskers show full range. *p<0.05 by Mann-Whitney *U* test. MFI: mean fluorescence intensity. IQR: interquartile range.
(TIF)

S4 Fig Correlations of the mean fluorescence intensity of desmoglein-1 and claudin-1 versus estradiol levels in the DMPA and control groups. Spearman’s correlations of the MFI of (A-B) desmoglein-1 and (C-D) claudin-1 expression as assessed for potential influence of plasma estradiol levels in the control and DMPA groups, respectively. P-values <0.05 considered significant. MFI: mean fluorescence intensity. DSG-1: desmoglein-1
(TIFF)

S5 Fig Comparison of levels of all proteins identified in CVL by the protein profiling assay in the DMPA vs. control groups. Boxes indicate medians and IQR and whiskers show full range. P-values calculated by Mann-Whitney *U* test, not adjusted for potential confounders. P-values <0.05 considered significant and marked in red. CVL: cervicovaginal lavage. AU: arbitrary units. IQR: Interquartile range.
(PDF)

S1 Table Plasma levels of estradiol and progesterone at study visit. (XLSX)

S2 Table All differentially expressed genes (FDR adjusted p<0.05) between the DMPA and control group. (XLSX)

S3 Table Gene set enrichment by GO (gene ontology) up and downregulated in the DMPA group as compared to controls. (XLSX)

S4 Table Gene pathways identified by Kyoto Encyclopedia of Genes and Genomes (KEGG) database to be up and downregulated in the DMPA group as compared to controls. (XLSX)

S5 Table Gene sets up and downregulated in DMPA users as compared to controls identified by WikiPathways. (XLSX)

S6 Table Transcription factors identified by the Transcriptional Regulatory Relationships Unraveled by Science-based Text mining (TRRUST) database to be up or downregulated in the DMPA group as compared to controls. (XLSX)

S7 Table Gene set enrichment analysis by GO (gene ontology) for DMPA vs. control groups divided into *Lactobacillus* and non-*Lactobacillus* dominated microbiomes, respectively. (XLSX)

S8 Table *In situ* imaging of protein expression of E-cadherin and CD4 in ectocervical tissue samples. (XLSX)

S9 Table Sociodemographic data and clinical characteristics of study subjects included in the *in situ* imaging analysis for desmoglein-1 and claudin-1 stainings. (PDF)

S10 Table *In situ* imaging of protein expression of desmoglein-1 and claudin-1 in ectocervical tissue samples. (XLSX)

S11 Table Proteins included in the protein-profiling assay. (XLSX)

S12 Table Protein levels in cervicovaginal lavage samples. (XLSX)

S13 Table Sociodemographic data and clinical characteristics of study subjects included in the protein profiling assay. (PDF)

S14 Table Adjustment of protein levels for age and time in sex work using generalized linear models. (PDF)

S15 Table Interaction analysis of the impact of the microbiome composition on the DMPA associated protein expression. (XLSX)

S1 Text Supplemental methods. (PDF)
