# Supplementary figures and images for: Multi-omics analysis of the cervical epithelial integrity of women using depot medroxyprogesterone acetate
Source: PLoS Pathog. 2022 May 9;18(5):e1010494. doi: 10.1371/journal.ppat.1010494 (PMC9119532; doi:10.1371/journal.ppat.1010494)

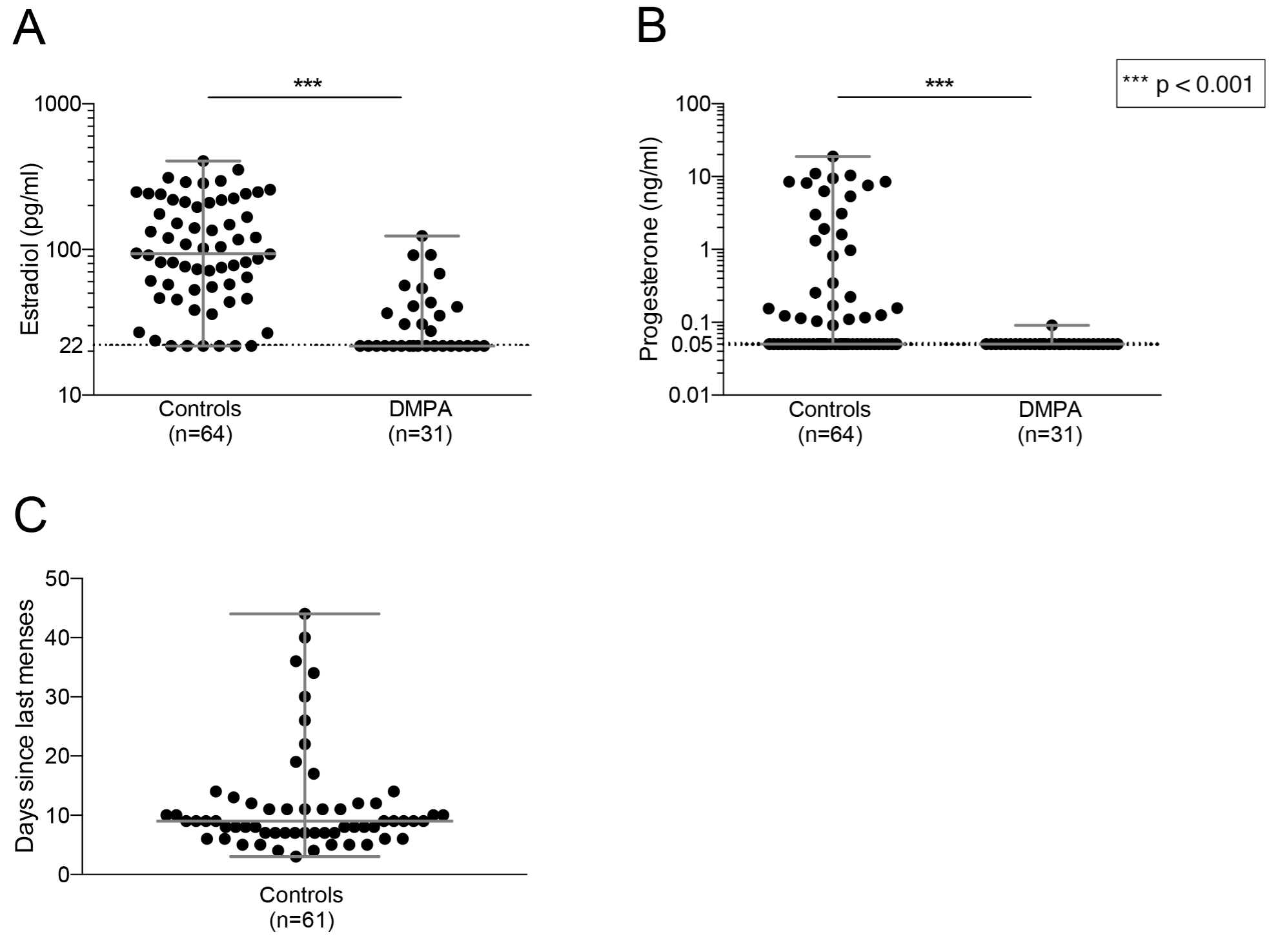

Supplement: S1 Fig — A and B) Plasma levels of estradiol (E2) and progesterone (P4) in the control group (n = 64) and the DMPA group (n = 31). LLD was 22 pg/mL and 0.05 ng/mL for E2 and P4, respectively. Values <22 pg/mL (E2) and <0.05 ng/mL (P4) were reported as “below LLD”, but for visual and statistical purposes, these values were assigned a value of 22 pg/ml and 0.05 ng/ml for E2 and P4, respectively, shown here by the dotted line. C. Self-reported days since onset of last menstrual period, this parameter was only applicable for the control group. Data not available for 3 samples, resulting in a total of 61 controls. The longer horizontal bar is median; whiskers indicating full range. P-values calculated by Mann-Whitney U test. LLD: lower limit of detection. (TIF) [file ppat.1010494.s001.tif]

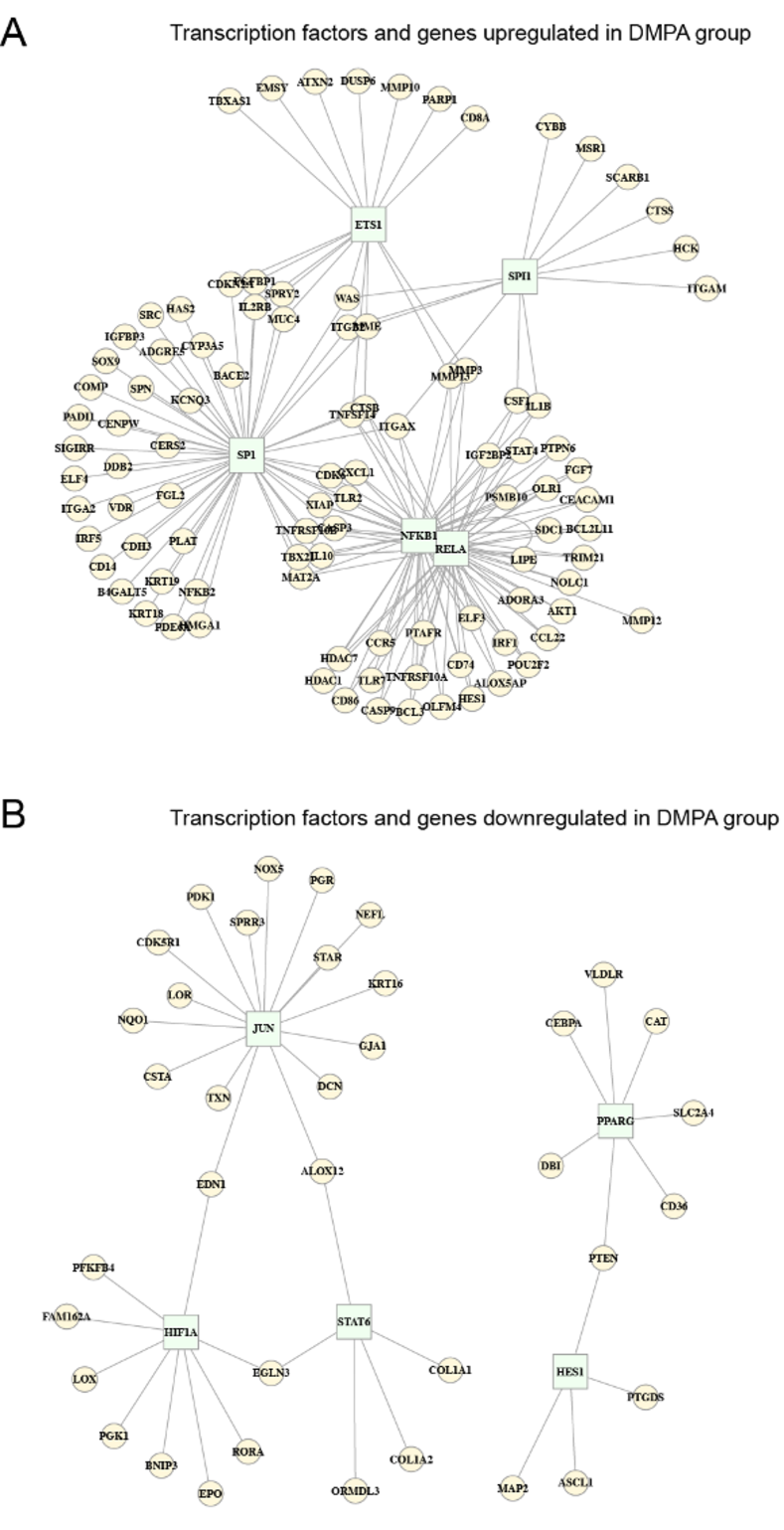

Supplement: S2 Fig — A and B) Each node represents a DEG that is upregulated (A) and downregulated (B), respectively, in the DMPA group. The lines connect the gene to the transcription factor(s) (in boxes) that have been reported to regulate the expression of that particular gene. Transcription factors were identified by the TRRUST database. DEG: differentially expressed gene. TRRUST: Transcriptional Regulatory Relationships Unraveled by Science-based Text mining. (TIF) [file ppat.1010494.s002.tif]

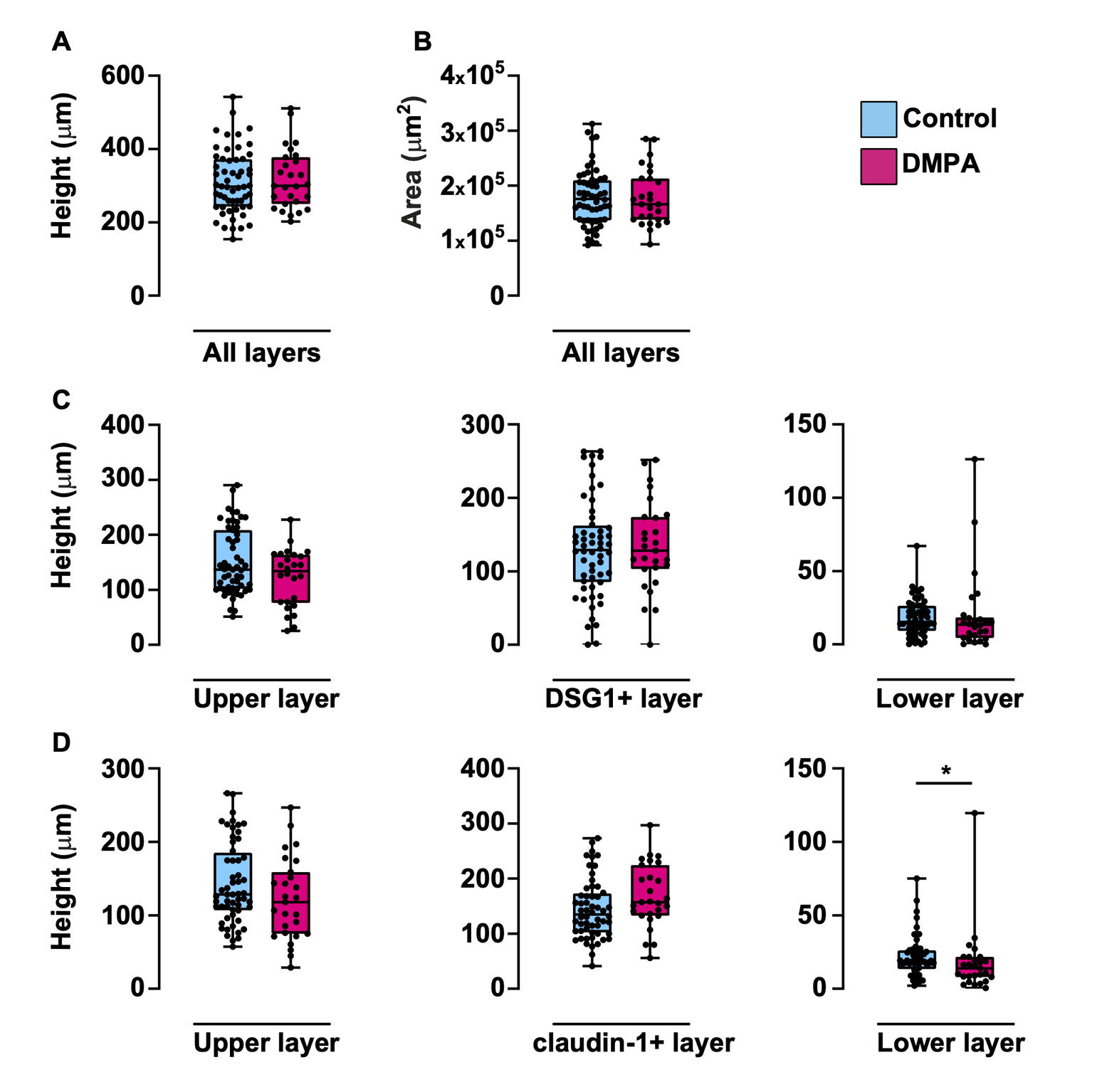

Supplement: S3 Fig — Boxplots showing the height (A) and area (B) of the ectocervical epithelium as well as the height of the three individual layers based on desmoglein-1 staining (C), and based on claudin-1 staining (D). Control group (n = 56); turquoise, DMPA (n = 27); pink. One individual from each study group had non-detectable desmogelin-1 staining and could thus not be included in the measurements for the three individual epithelial layers. Boxplots indicate medians and IQR and whiskers show full range. *p<0.05 by Mann-Whitney U test. MFI: mean fluorescence intensity. IQR: interquartile range. (TIF) [file ppat.1010494.s003.tif]

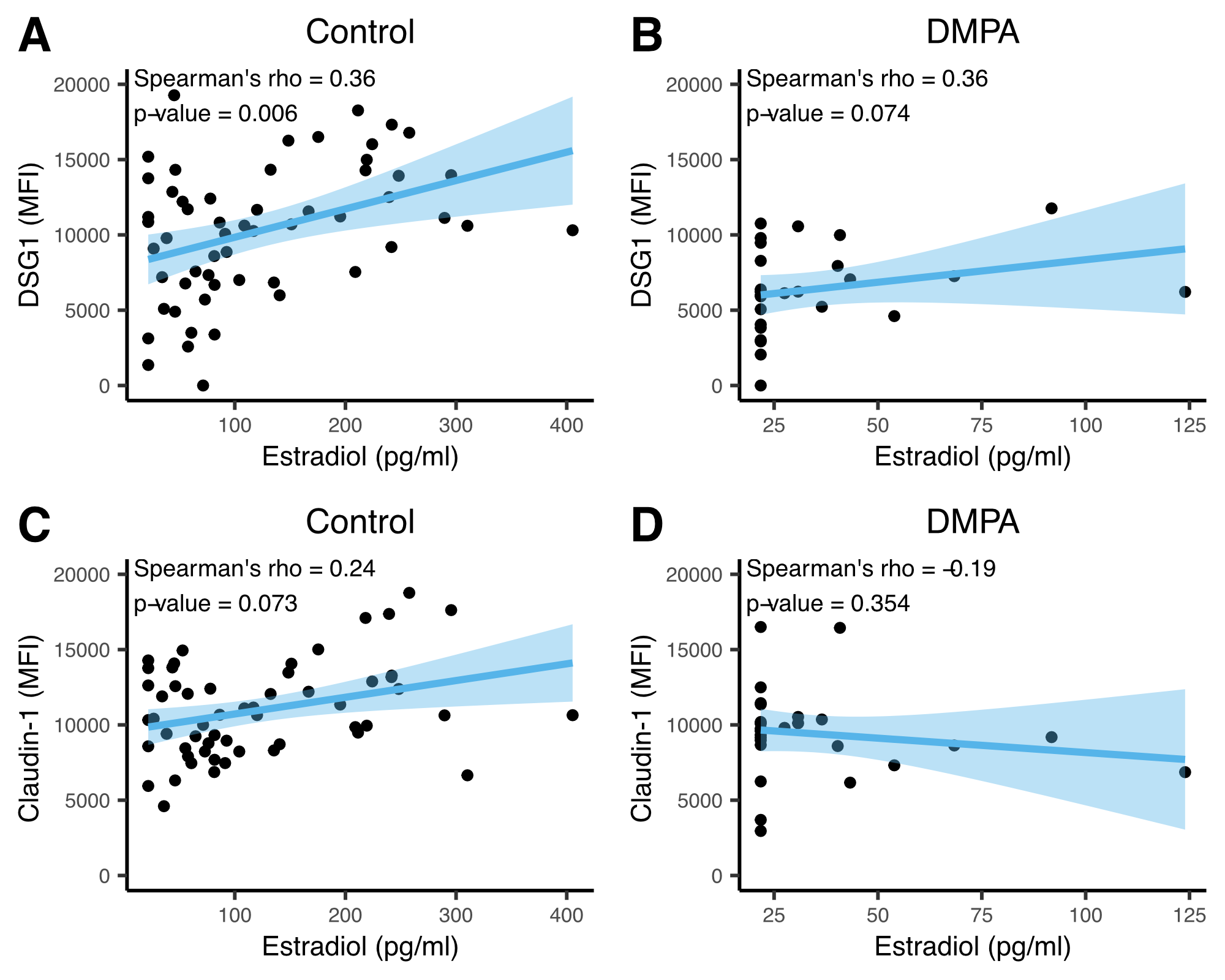

Supplement: S4 Fig — Spearman’s correlations of the MFI of (A-B) desmoglein-1 and (C-D) claudin-1 expression as assessed for potential influence of plasma estradiol levels in the control and DMPA groups, respectively. P-values <0.05 considered significant. MFI: mean fluorescence intensity. DSG-1: desmoglein-1 (TIFF) [file ppat.1010494.s004.tiff]
