# Supplementary material for: Multi-omics analysis of the cervical epithelial integrity of women using depot medroxyprogesterone acetate
Source: PLoS Pathog. 2022 May 9;18(5):e1010494. doi: 10.1371/journal.ppat.1010494 (PMC9119532; doi:10.1371/journal.ppat.1010494)
Supplement: S5 Fig — Boxes indicate medians and IQR and whiskers show full range. P-values calculated by Mann-Whitney U test, not adjusted for potential confounders. P-values <0.05 considered significant and marked in red. CVL: cervicovaginal lavage. AU: arbitrary units. IQR: Interquartile range. (PDF) [file ppat.1010494.s005.pdf]

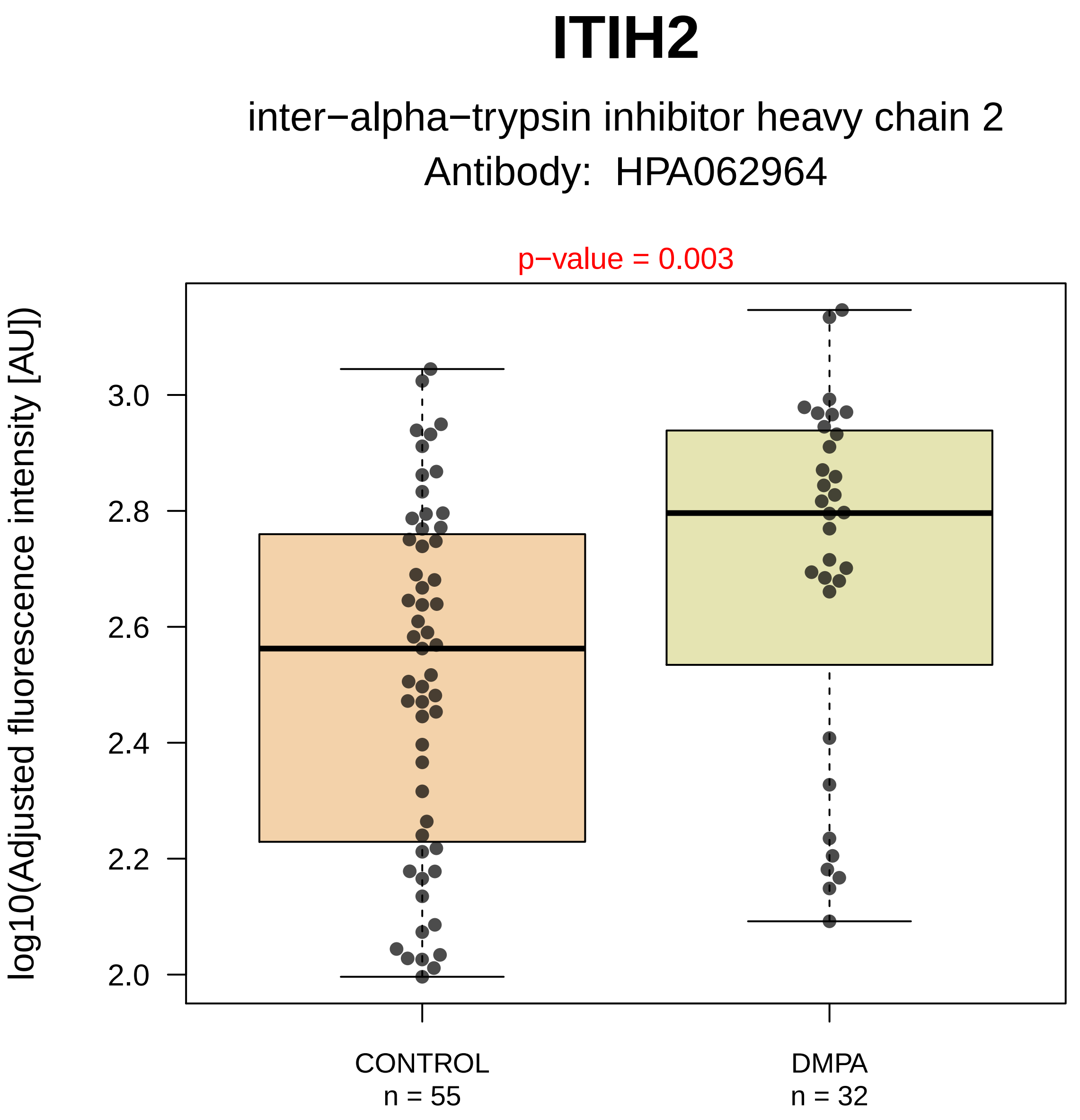
ITIH2
inter−alpha−trypsin inhibitor heavy chain 2
Antibody: HPA062964
p−value = 0.003
log10(Adjusted fluorescence intensity [AU])
3.0
2.8
2.6
2.4
2.2
2.0
CONTROL
n = 55
DMPA
n = 32

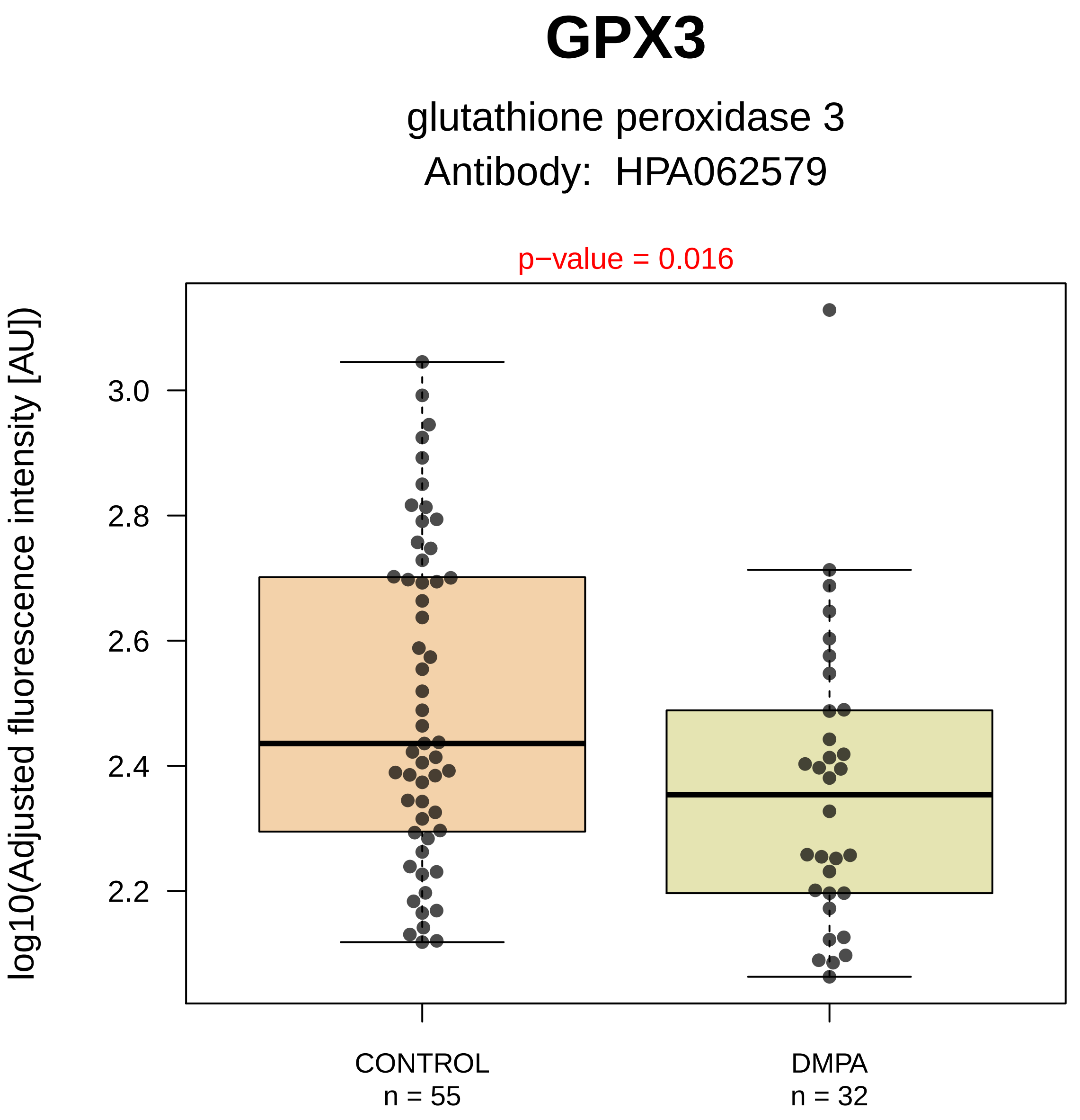

GPX3
glutathione peroxidase 3
Antibody: HPA062579
p−value = 0.016
log10(Adjusted fluorescence intensity [AU])
3.0
2.8
2.6
2.4
2.2
CONTROL
n = 55
DMPA
n = 32

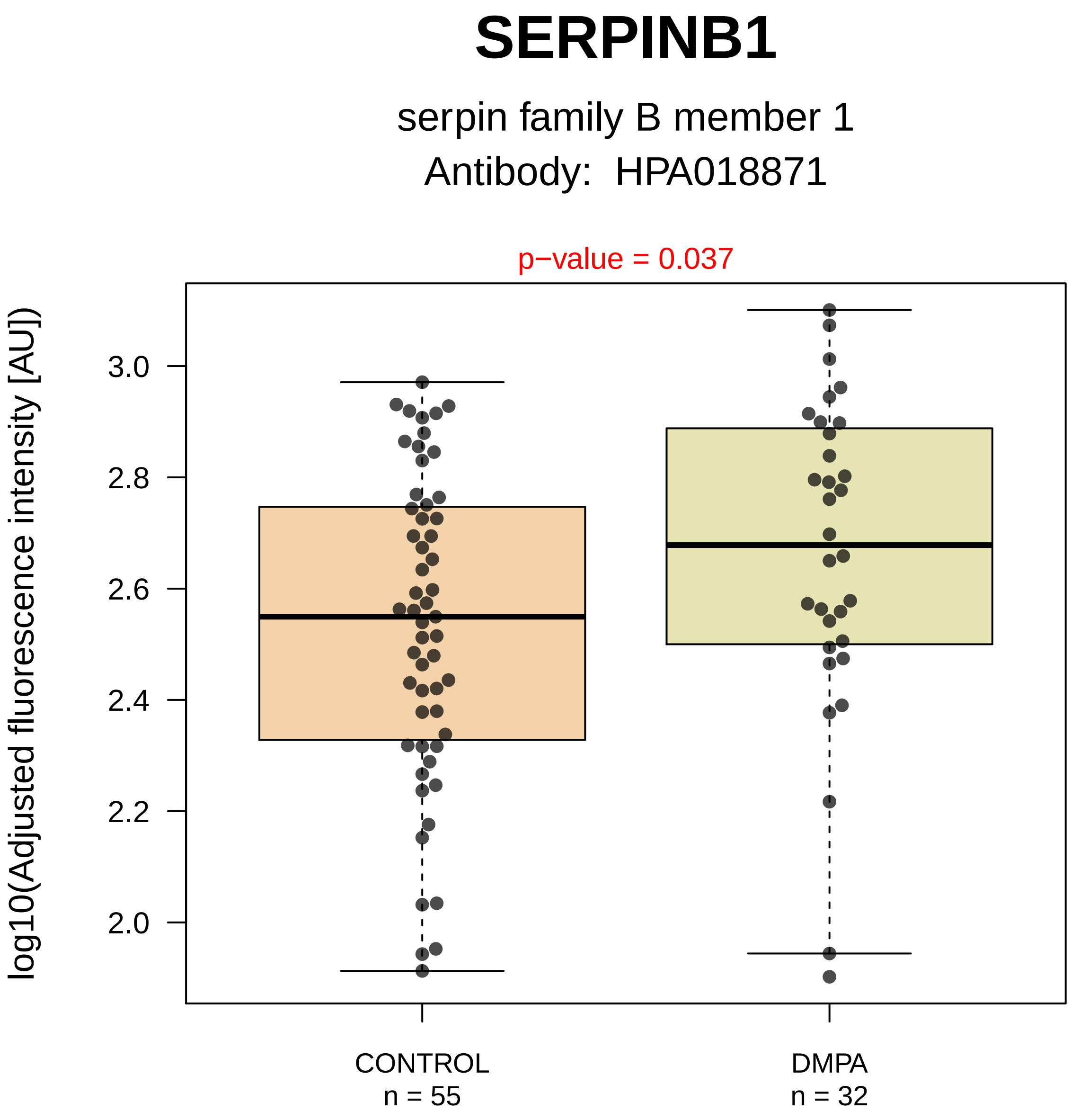

SERPINB1
serpin family B member 1
Antibody: HPA018871
p−value = 0.037
log10(Adjusted fluorescence intensity [AU])
3.0
2.8
2.6
2.4
2.2
2.0
CONTROL
n = 55
DMPA
n = 32

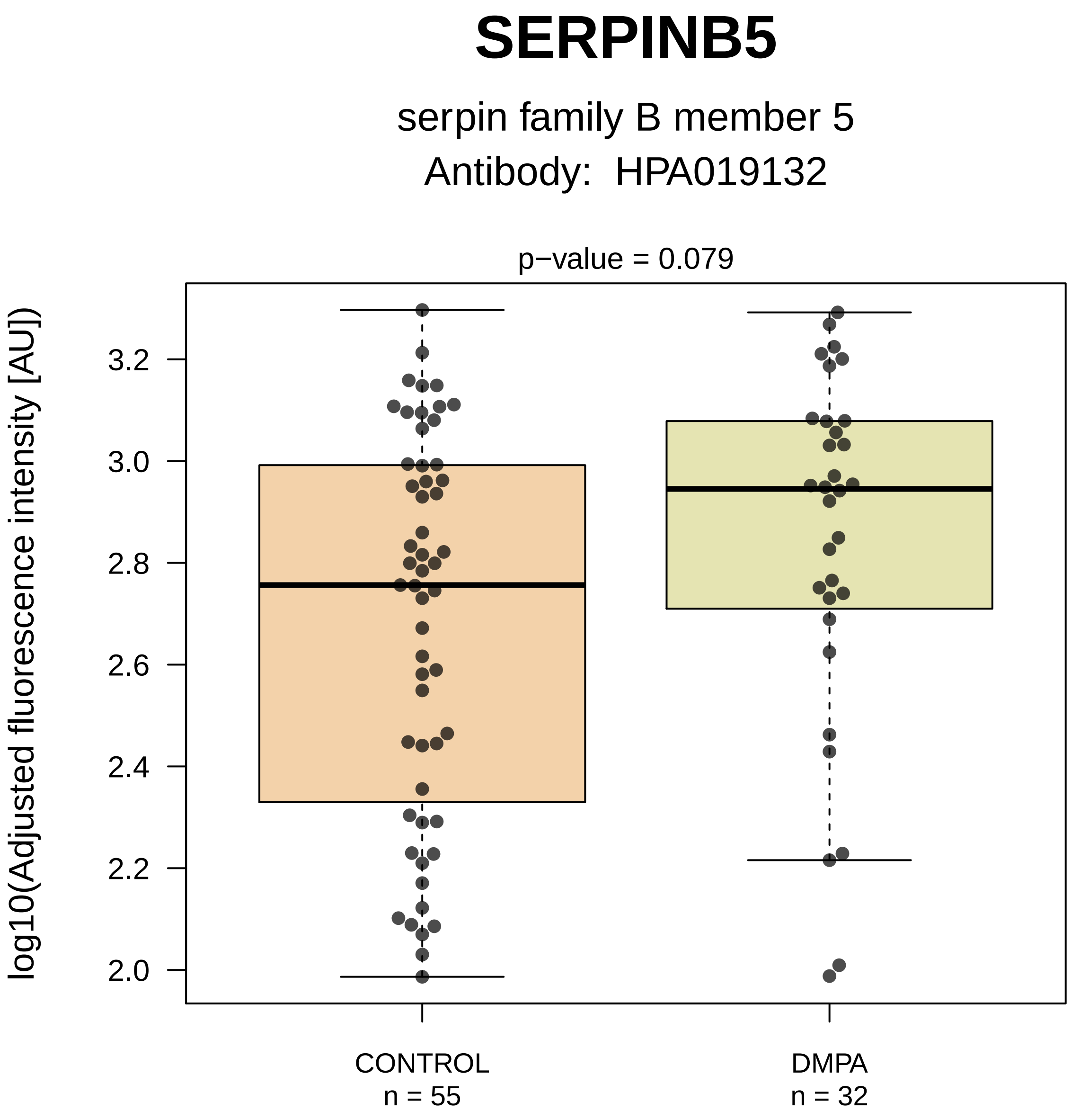

SERPINB5
serpin family B member 5
Antibody: HPA019132
p−value = 0.079
log10(Adjusted fluorescence intensity [AU])
3.2
3.0
2.8
2.6
2.4
2.2
2.0
CONTROL
n = 55
DMPA
n = 32

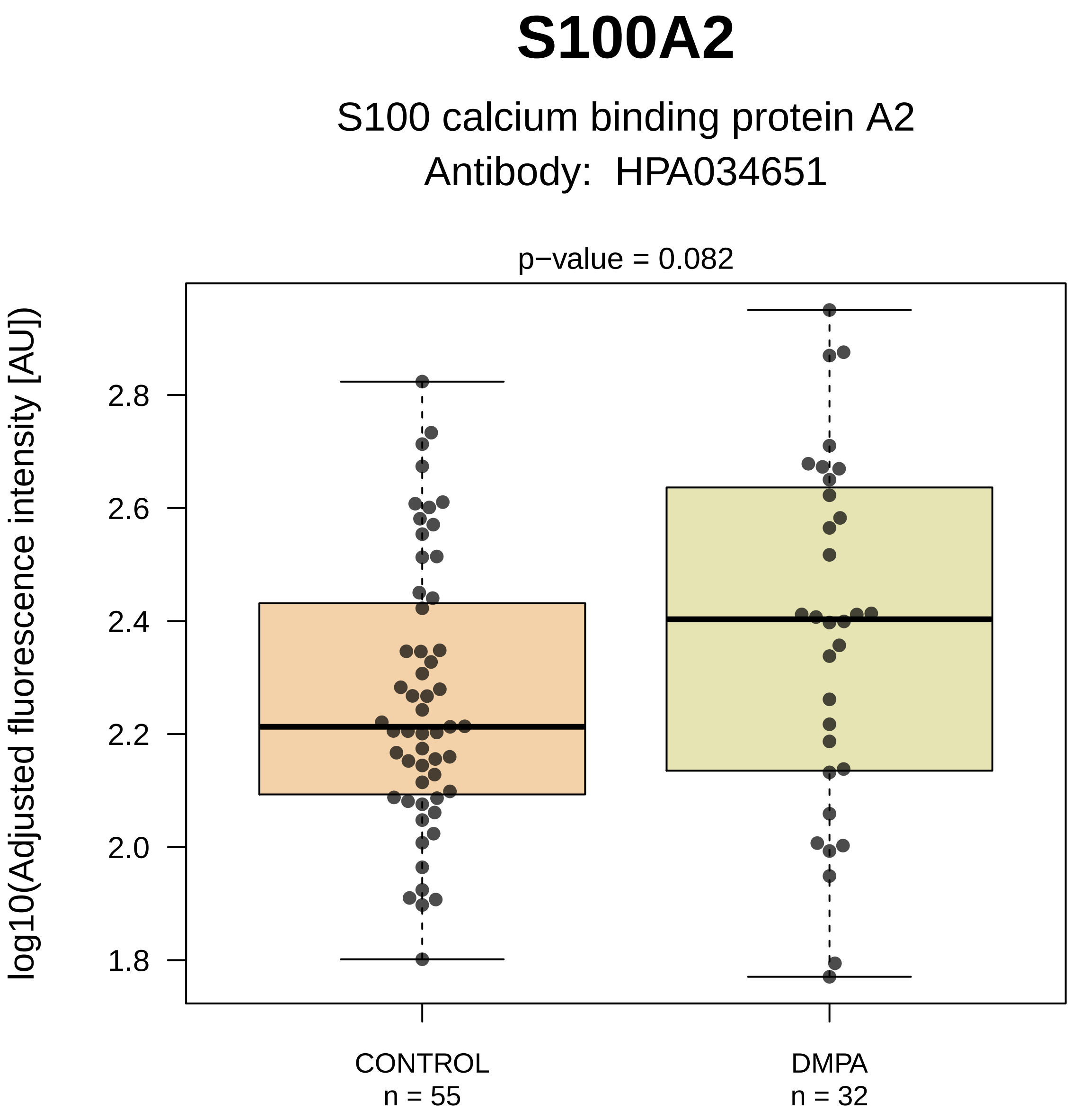
S100A2
S100 calcium binding protein A2
Antibody: HPA034651
p−value = 0.082
log10(Adjusted fluorescence intensity [AU])
2.8
2.6
2.4
2.2
2.0
1.8
CONTROL
n = 55
DMPA
n = 32

ANXA1

annexin A1

Antibody: HPA011918

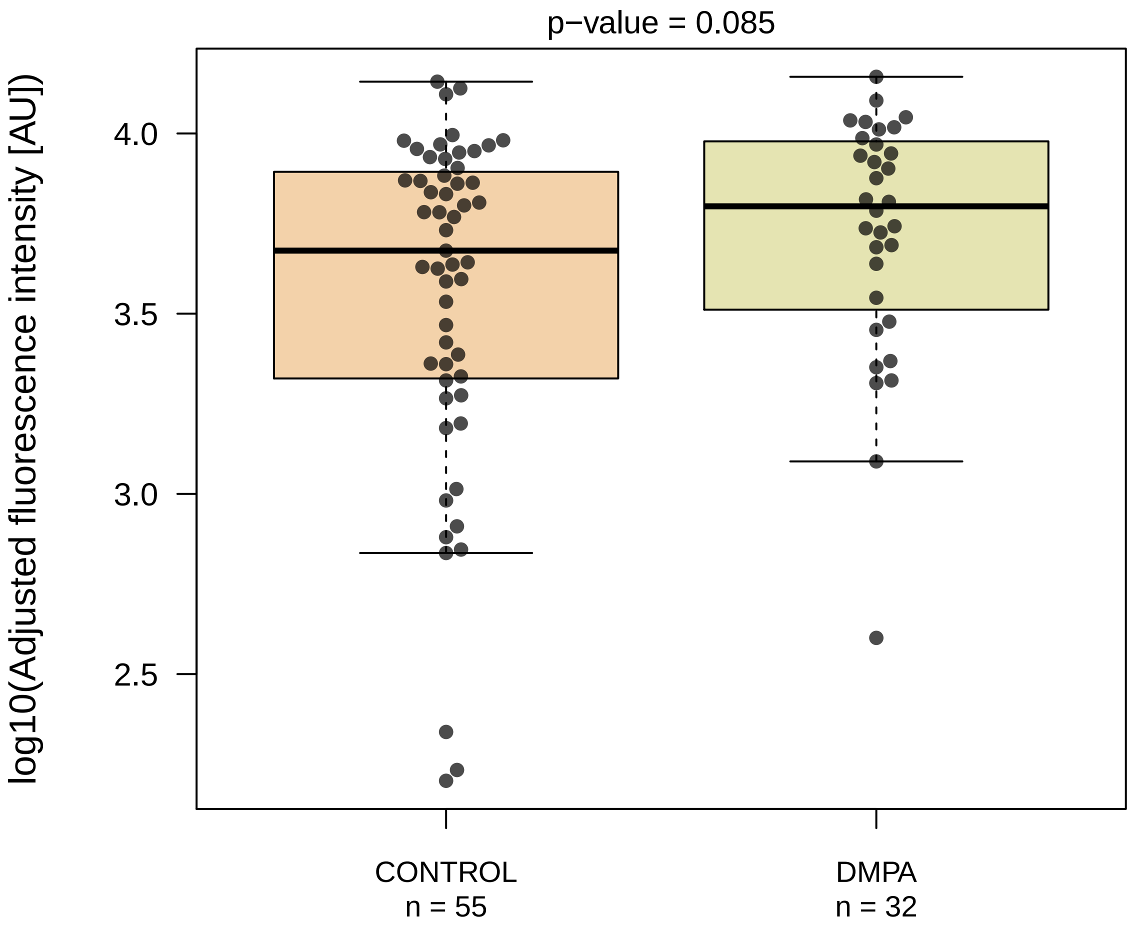

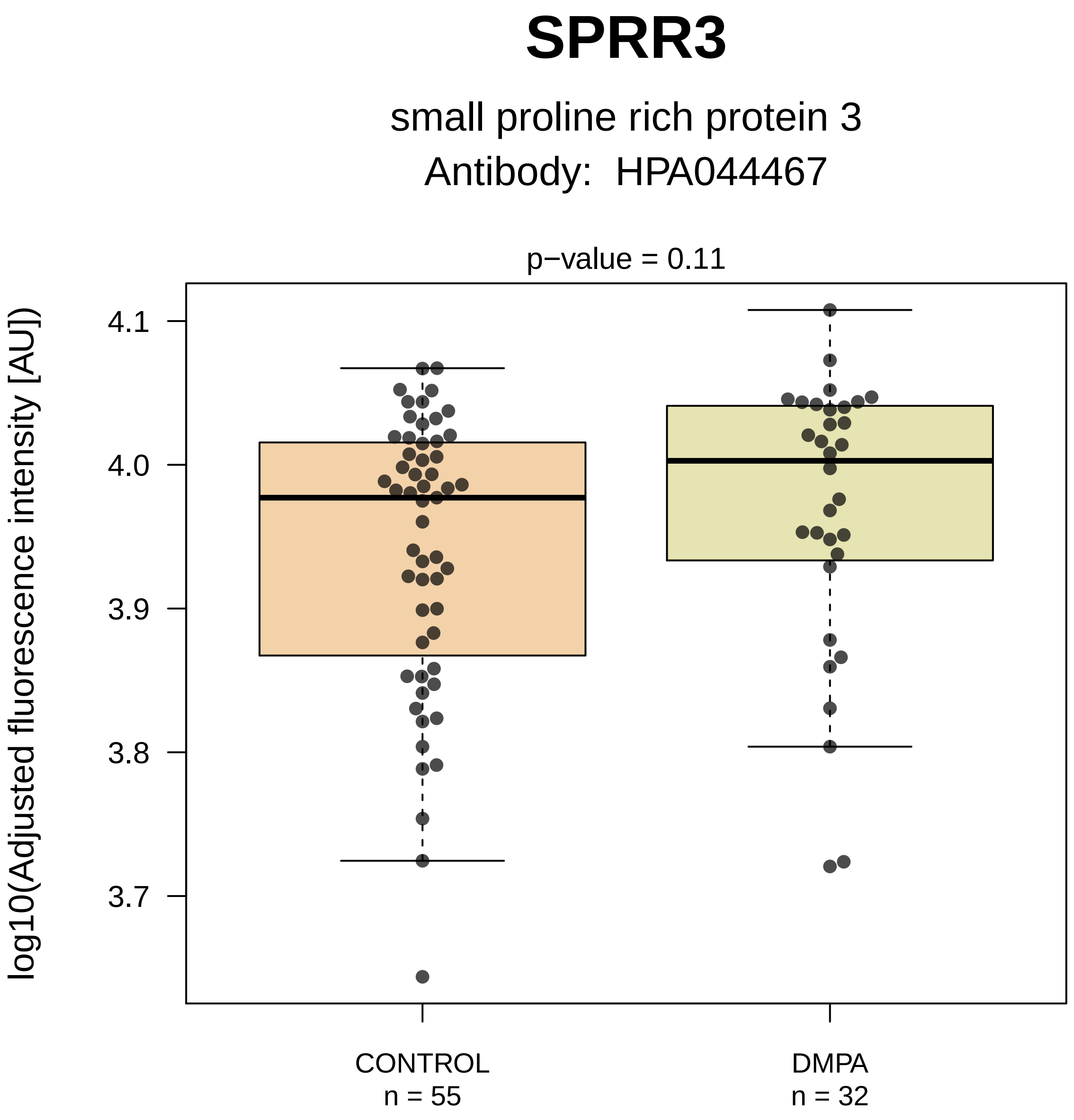
SPRR3
small proline rich protein 3
Antibody: HPA044467
p−value = 0.11
log10(Adjusted fluorescence intensity [AU])
4.1
4.0
3.9
3.8
3.7
CONTROL
n = 55
DMPA
n = 32

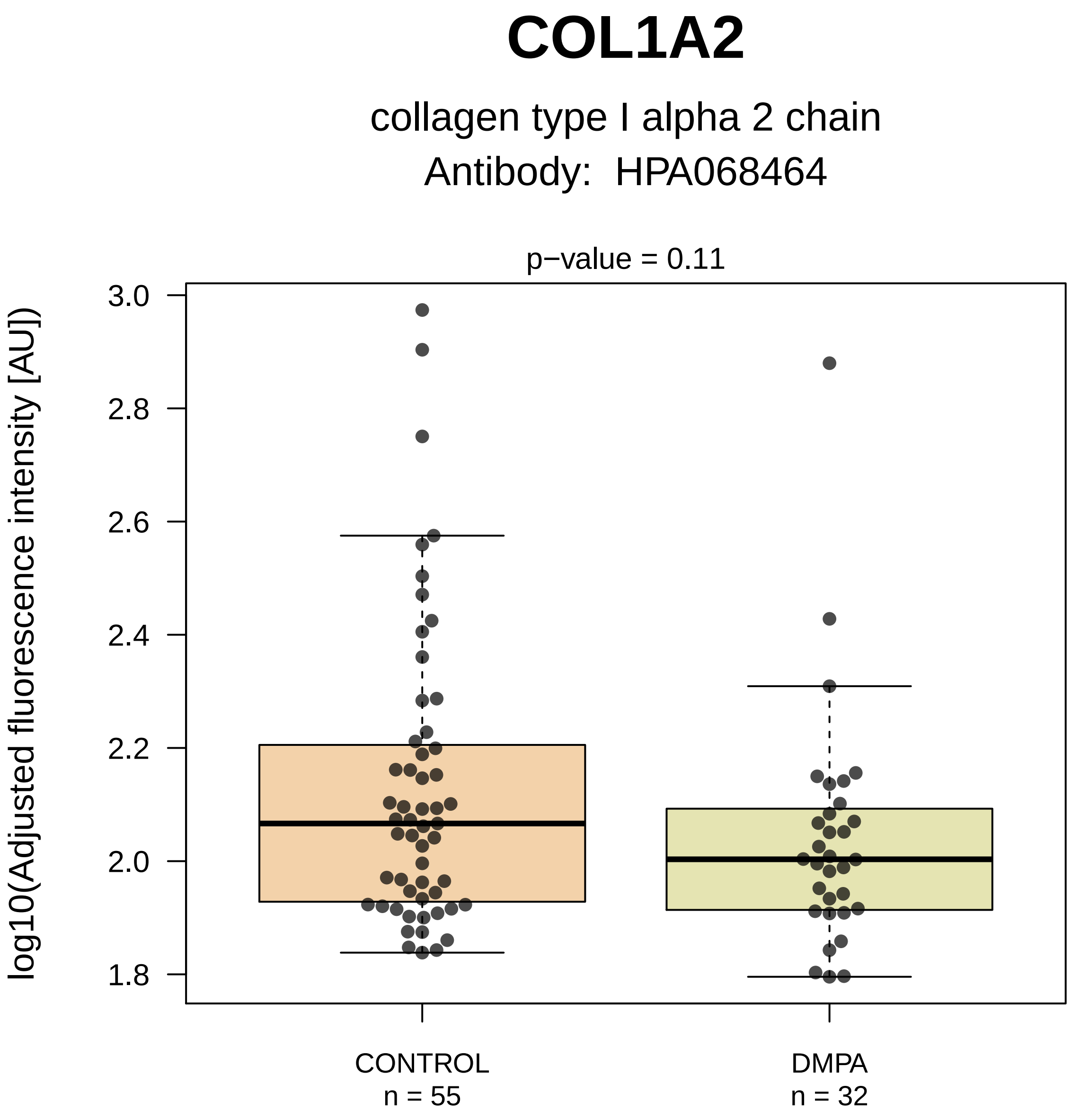
COL1A2
collagen type I alpha 2 chain
Antibody: HPA068464
p−value = 0.11
log10(Adjusted fluorescence intensity [AU])
3.0
2.8
2.6
2.4
2.2
2.0
1.8
CONTROL
n = 55
DMPA
n = 32

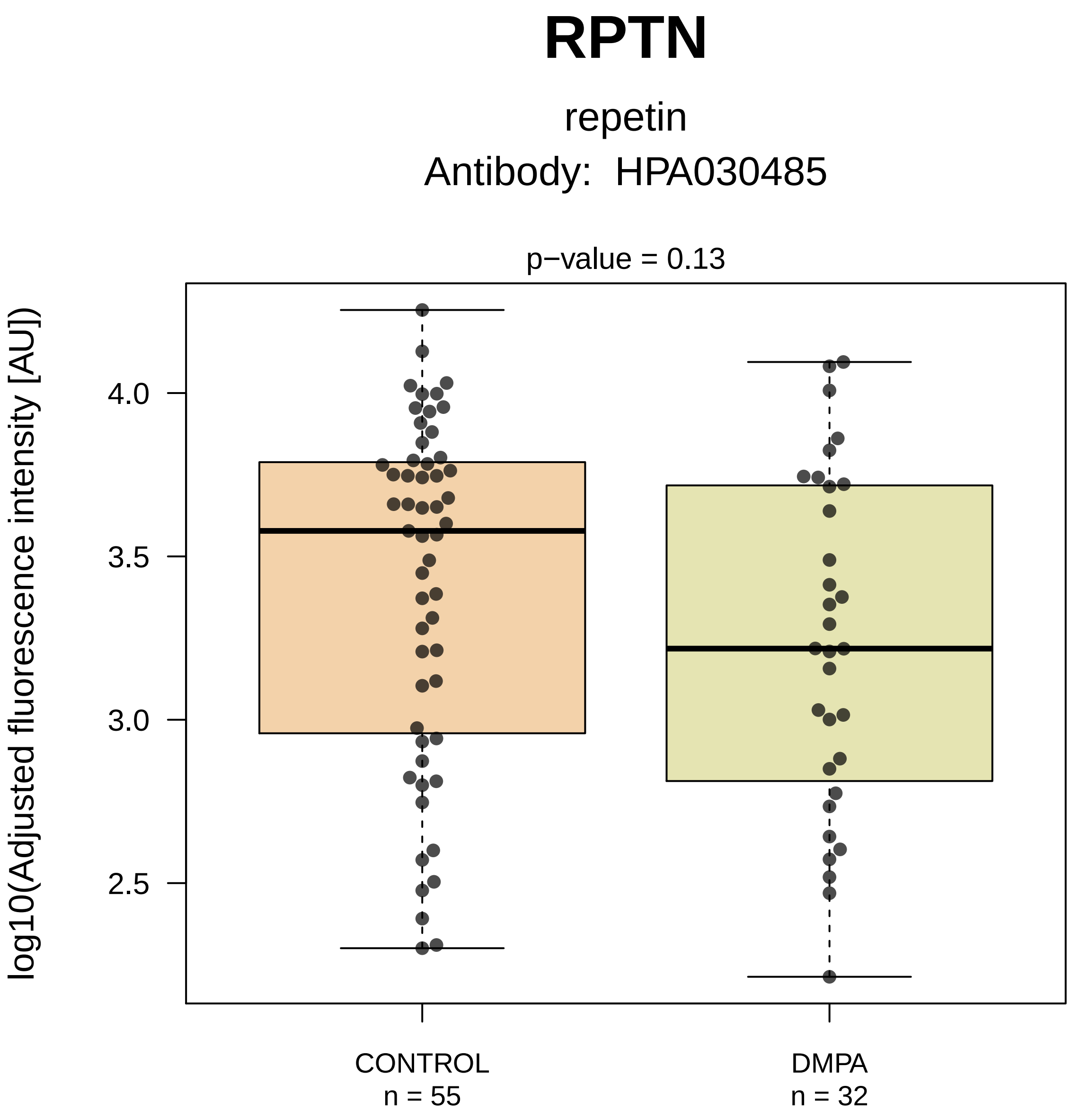

RPTN
repetin
Antibody: HPA030485
p−value = 0.13
log10(Adjusted fluorescence intensity [AU])
4.0
3.5
3.0
2.5
CONTROL
n = 55
DMPA
n = 32

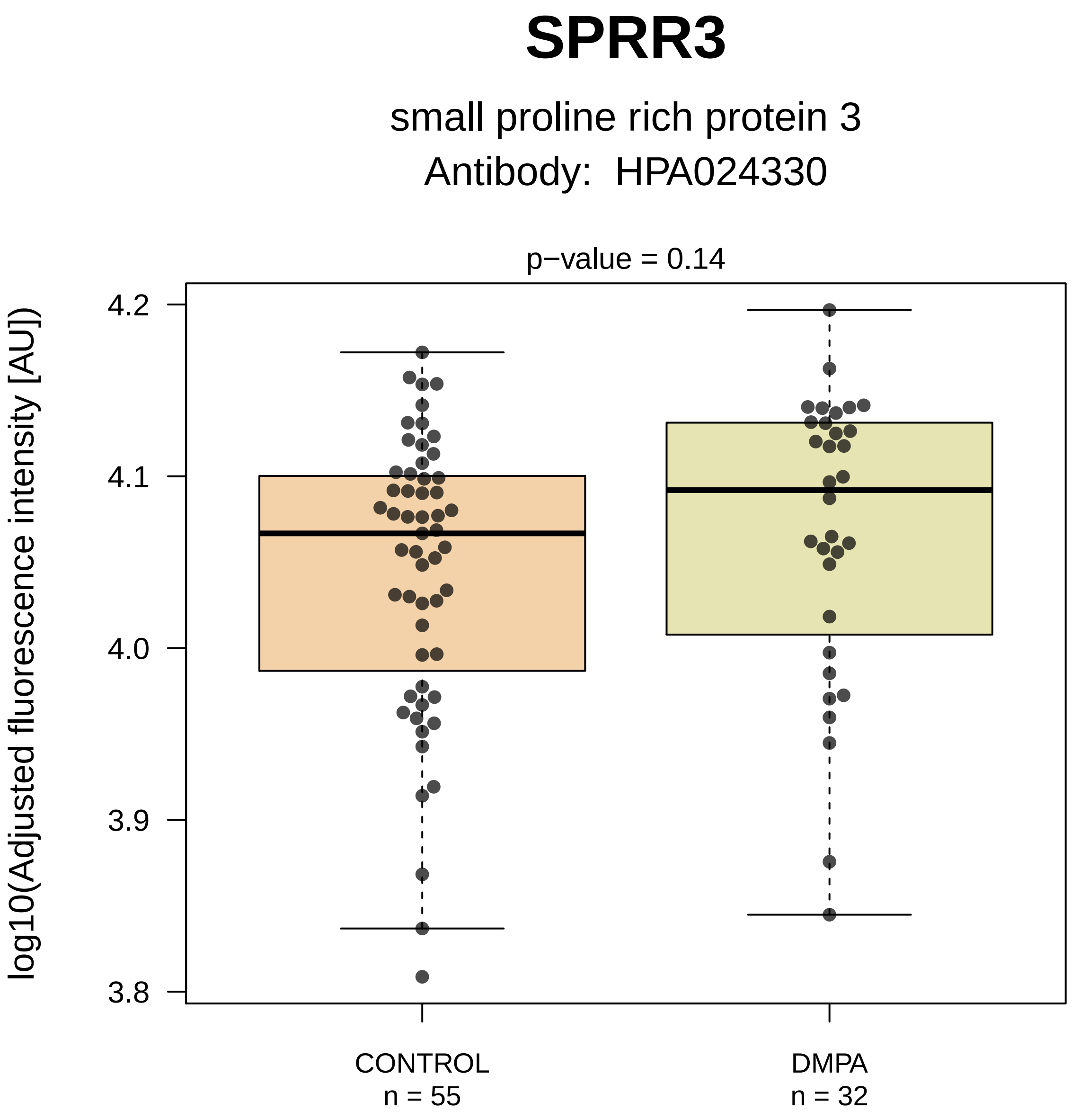
SPRR3
small proline rich protein 3
Antibody: HPA024330
p−value = 0.14
log10(Adjusted fluorescence intensity [AU])
4.2
4.1
4.0
3.9
3.8
CONTROL
n = 55
DMPA
n = 32

MUC16

mucin 16, cell surface associated
Antibody: HPA065600

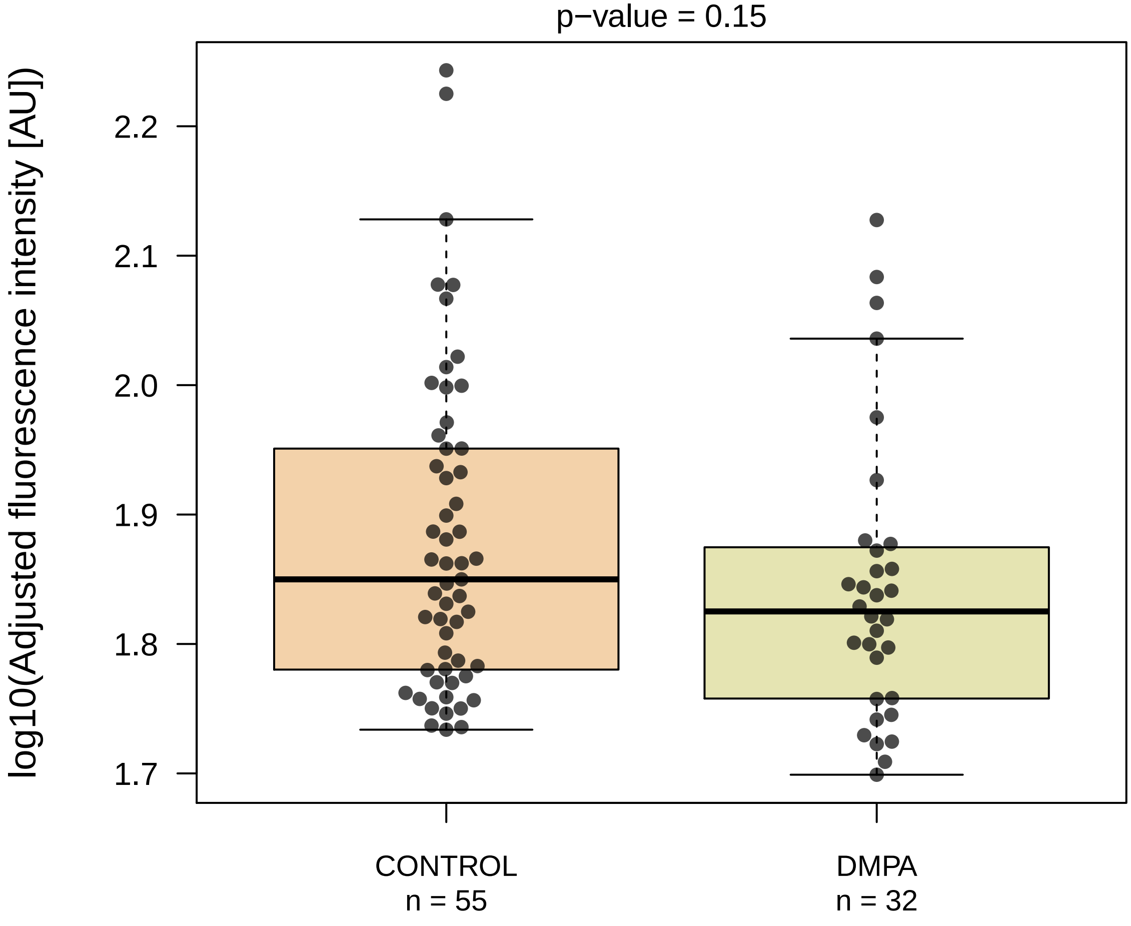

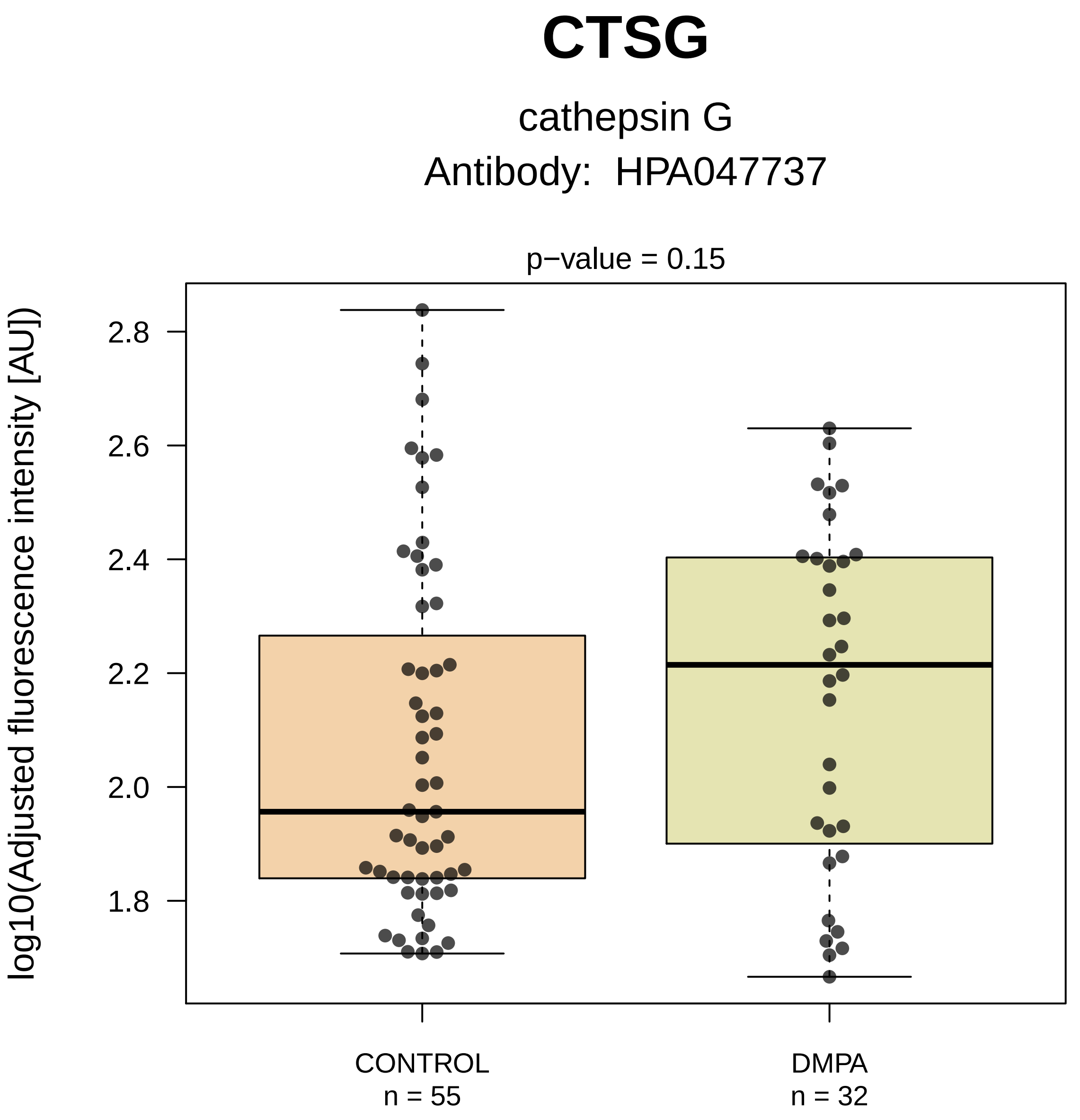

CTSG
cathepsin G
Antibody: HPA047737
p−value = 0.15
log10(Adjusted fluorescence intensity [AU])
2.8
2.6
2.4
2.2
2.0
1.8
CONTROL
n = 55
DMPA
n = 32

TACSTD2

tumor associated calcium signal transducer 2
Antibody: HPA055067

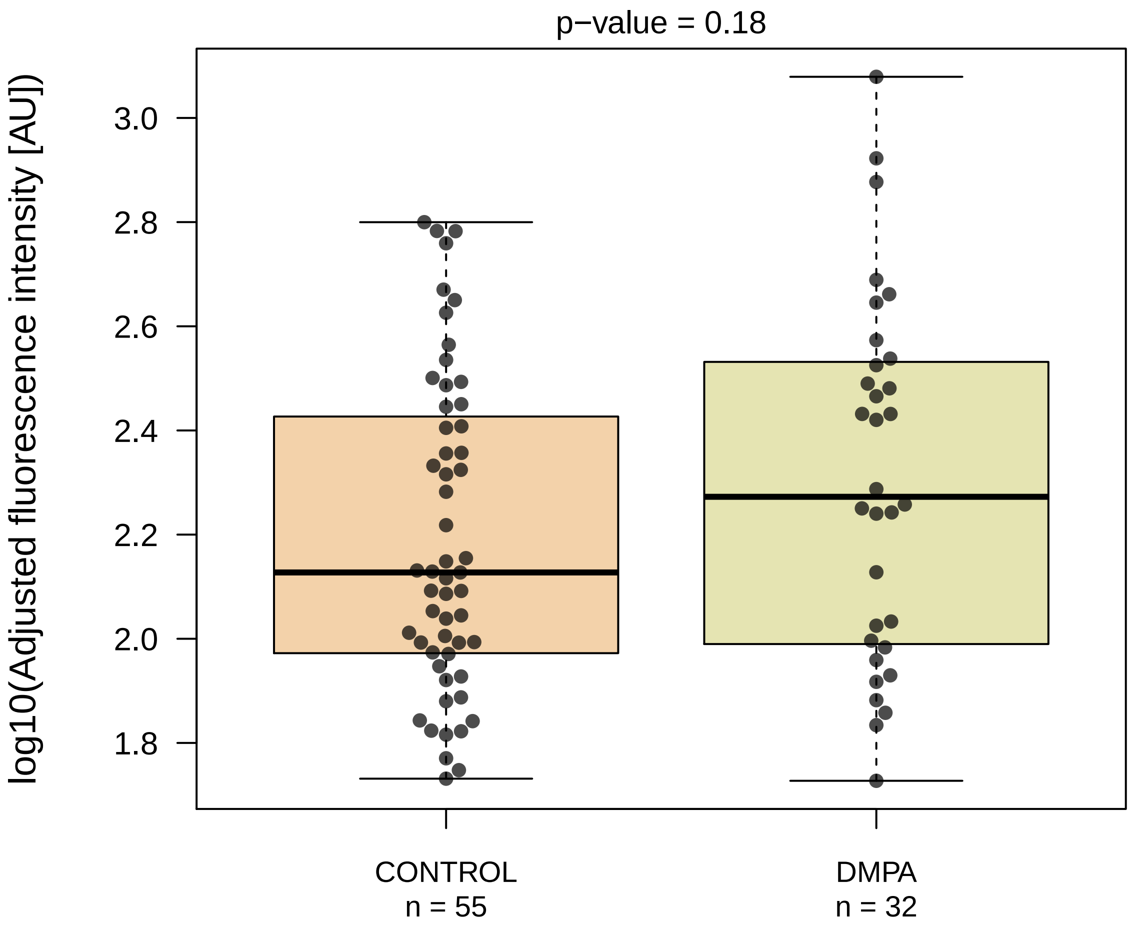

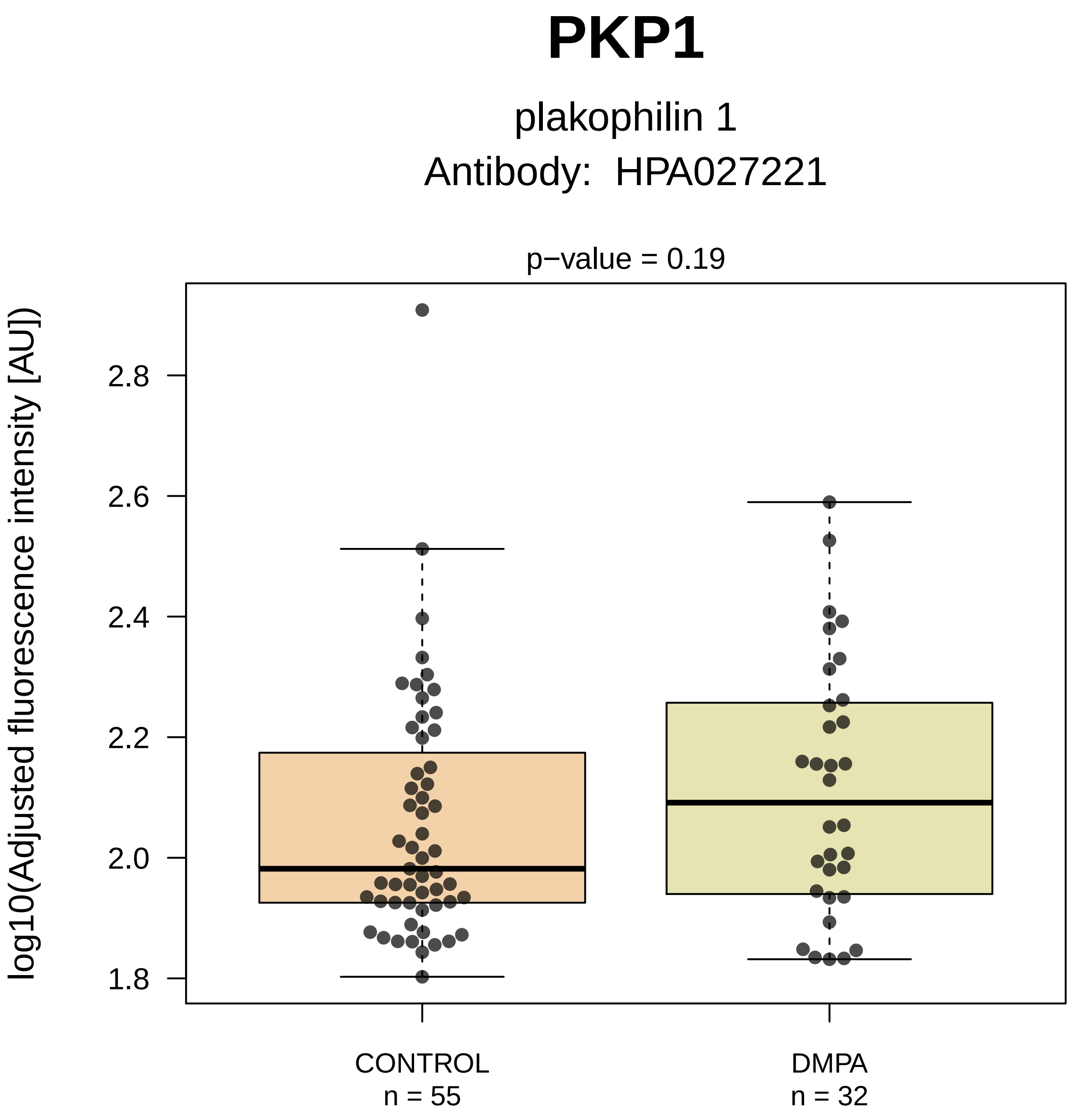
PKP1
plakophilin 1
Antibody: HPA027221
p−value = 0.19
log10(Adjusted fluorescence intensity [AU])
2.8
2.6
2.4
2.2
2.0
1.8
CONTROL
n = 55
DMPA
n = 32

BCAP31

B cell receptor associated protein 31

Antibody: HPA003906

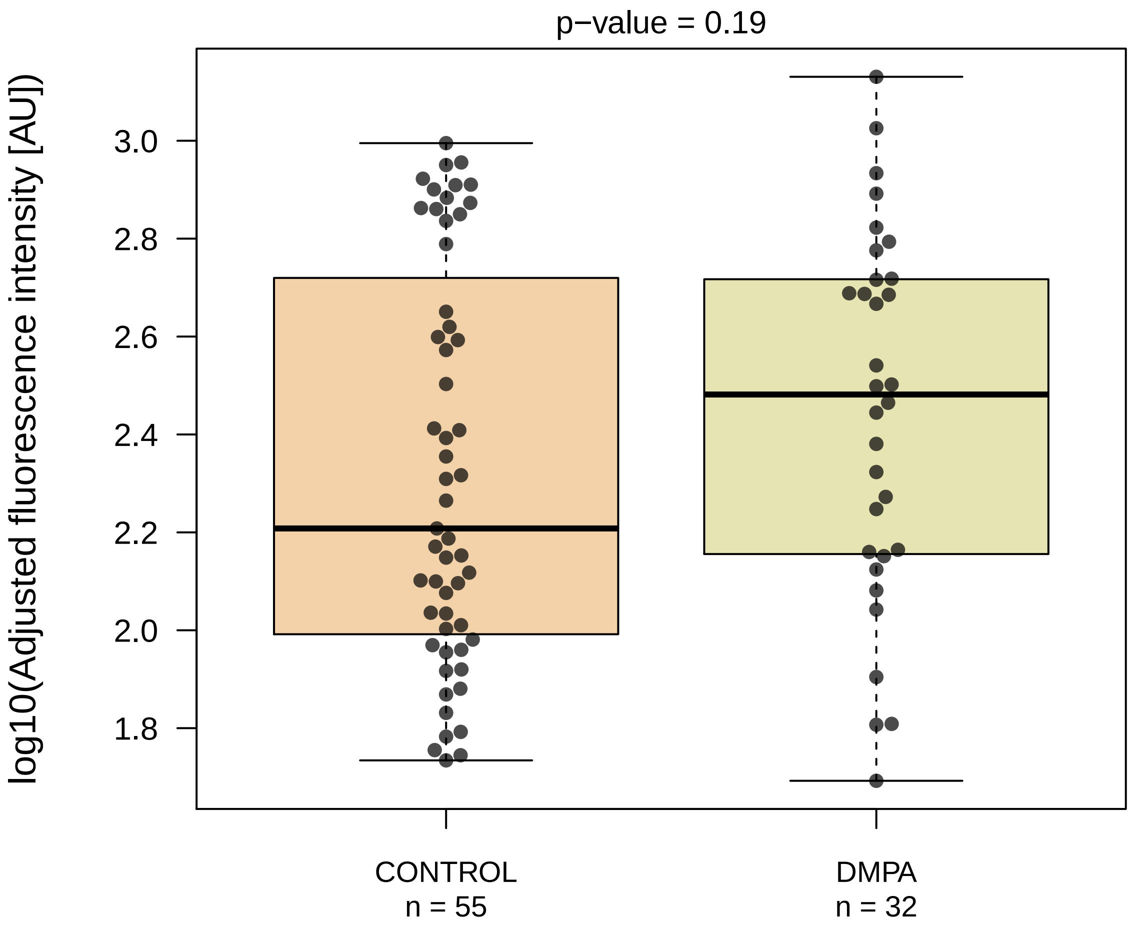

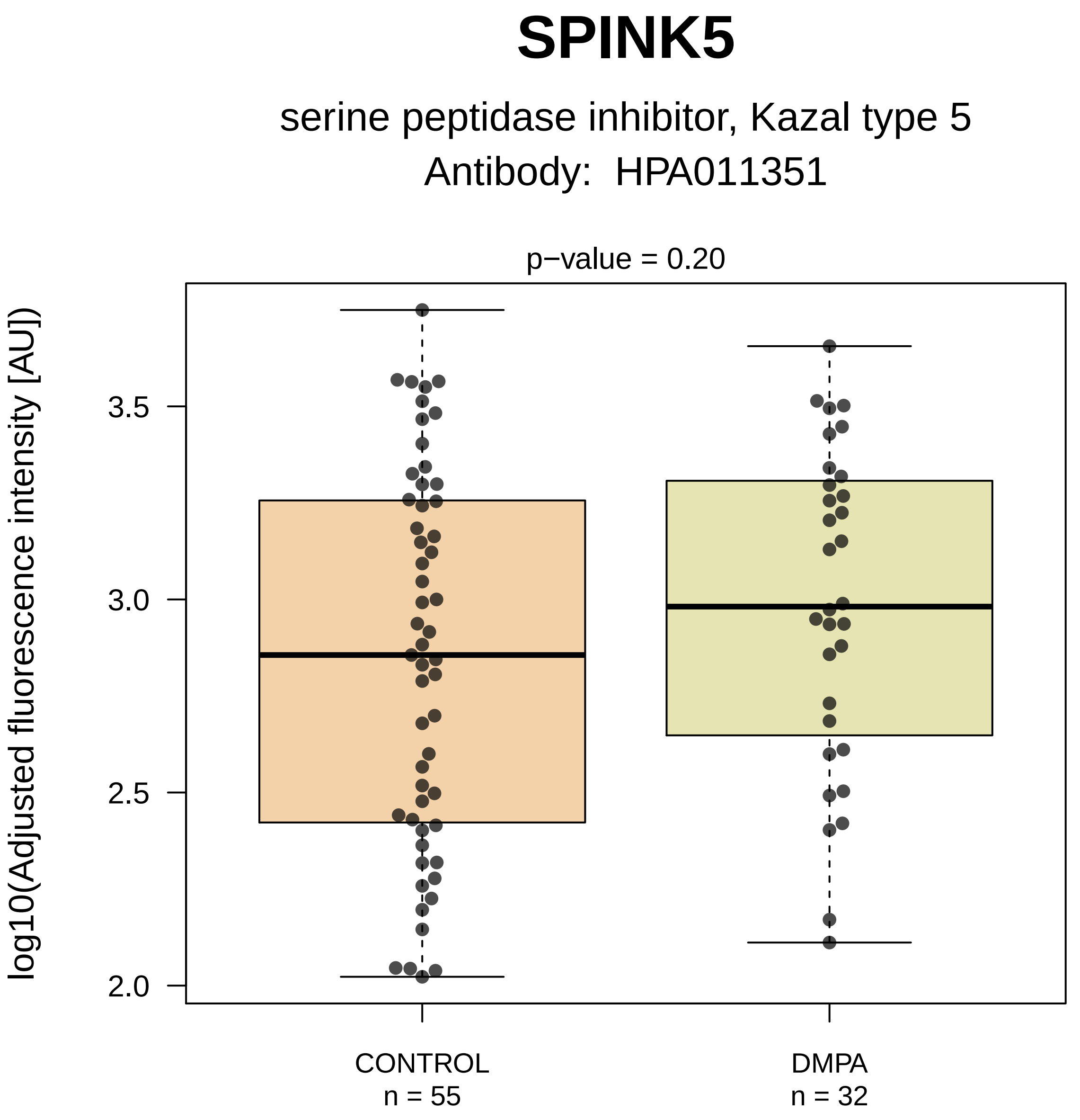
SPINK5
serine peptidase inhibitor, Kazal type 5
Antibody: HPA011351
p−value = 0.20
log10(Adjusted fluorescence intensity [AU])
3.5
3.0
2.5
2.0
CONTROL
n = 55
DMPA
n = 32

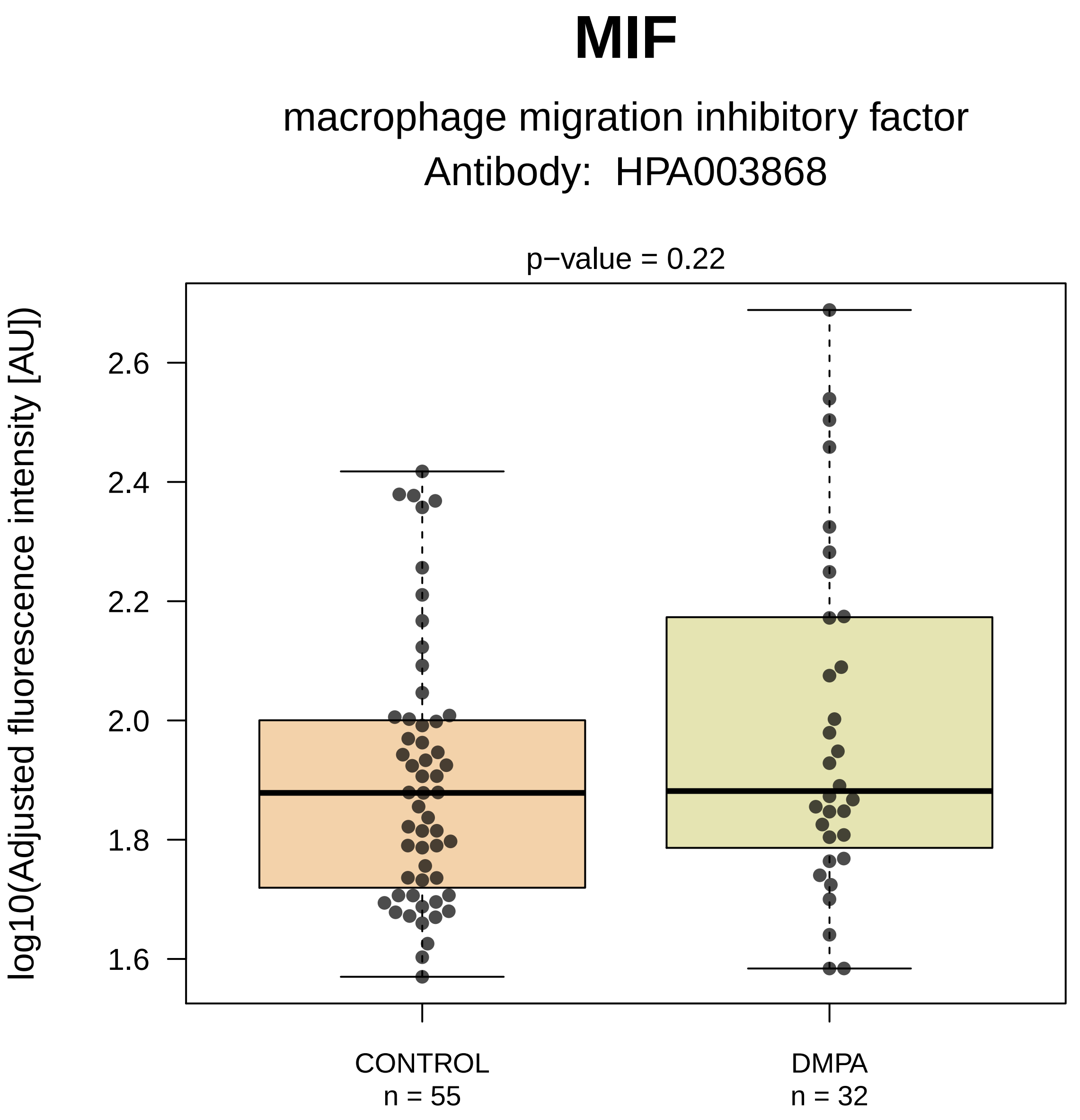
MIF
macrophage migration inhibitory factor
Antibody: HPA003868
p−value = 0.22
log10(Adjusted fluorescence intensity [AU])
2.6
2.4
2.2
2.0
1.8
1.6
CONTROL
n = 55
DMPA
n = 32

FLNA

filamin A

Antibody: HPA002925

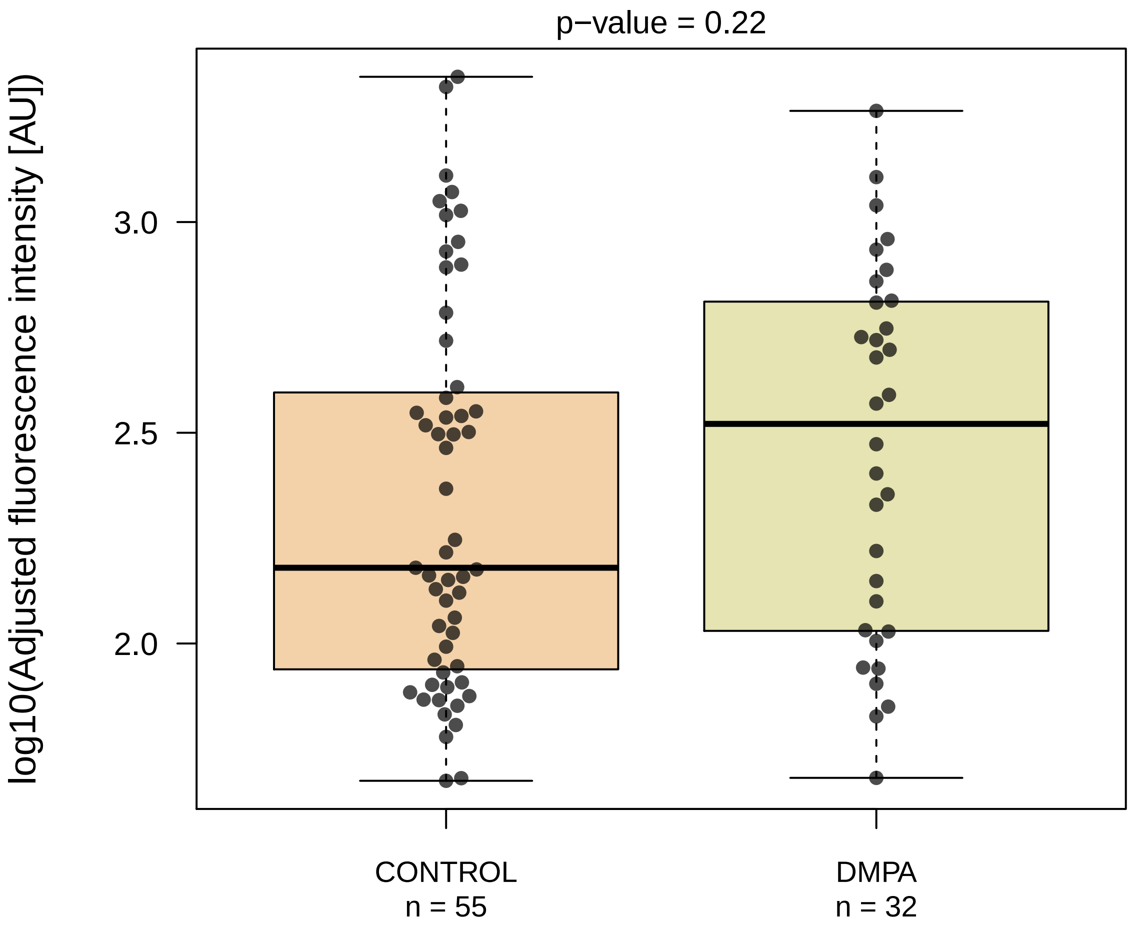

SERPINA1

serpin family A member 1
Antibody: HPA001291

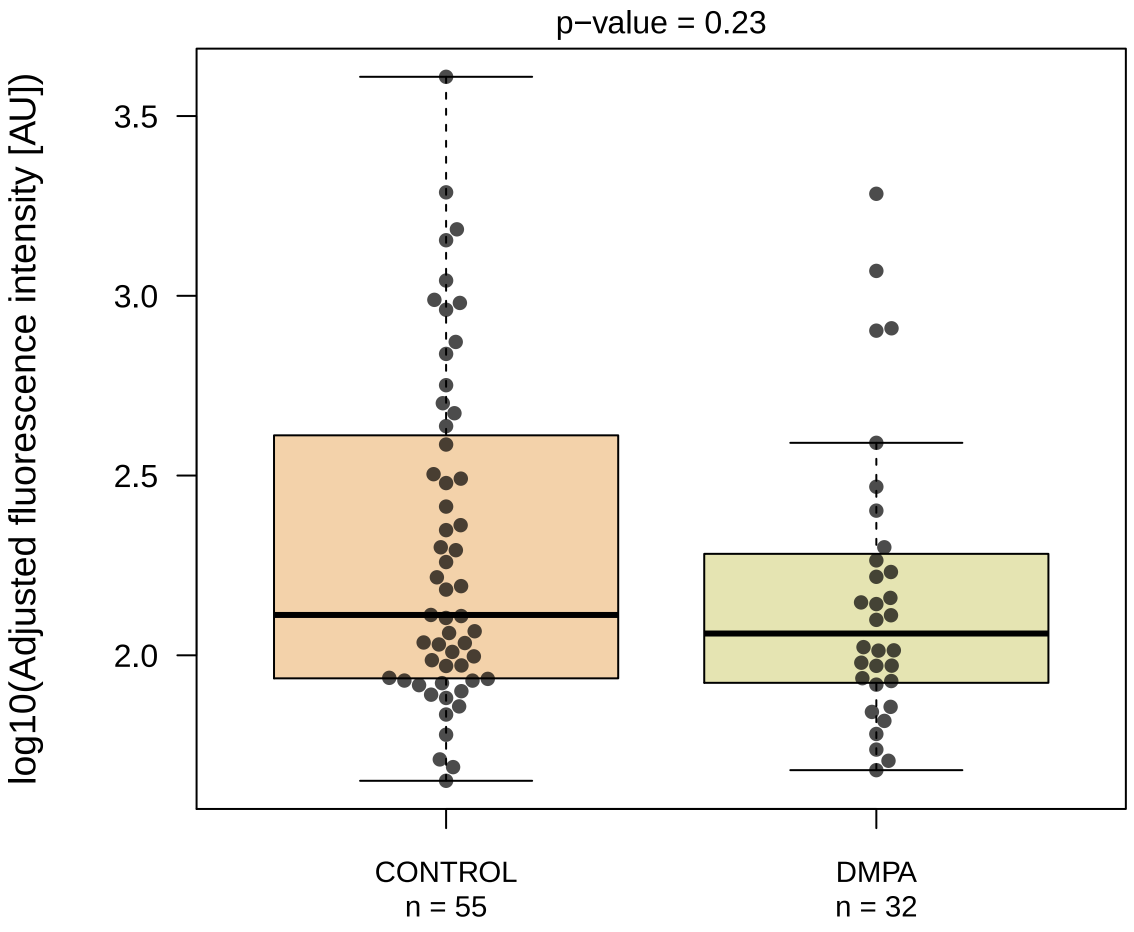

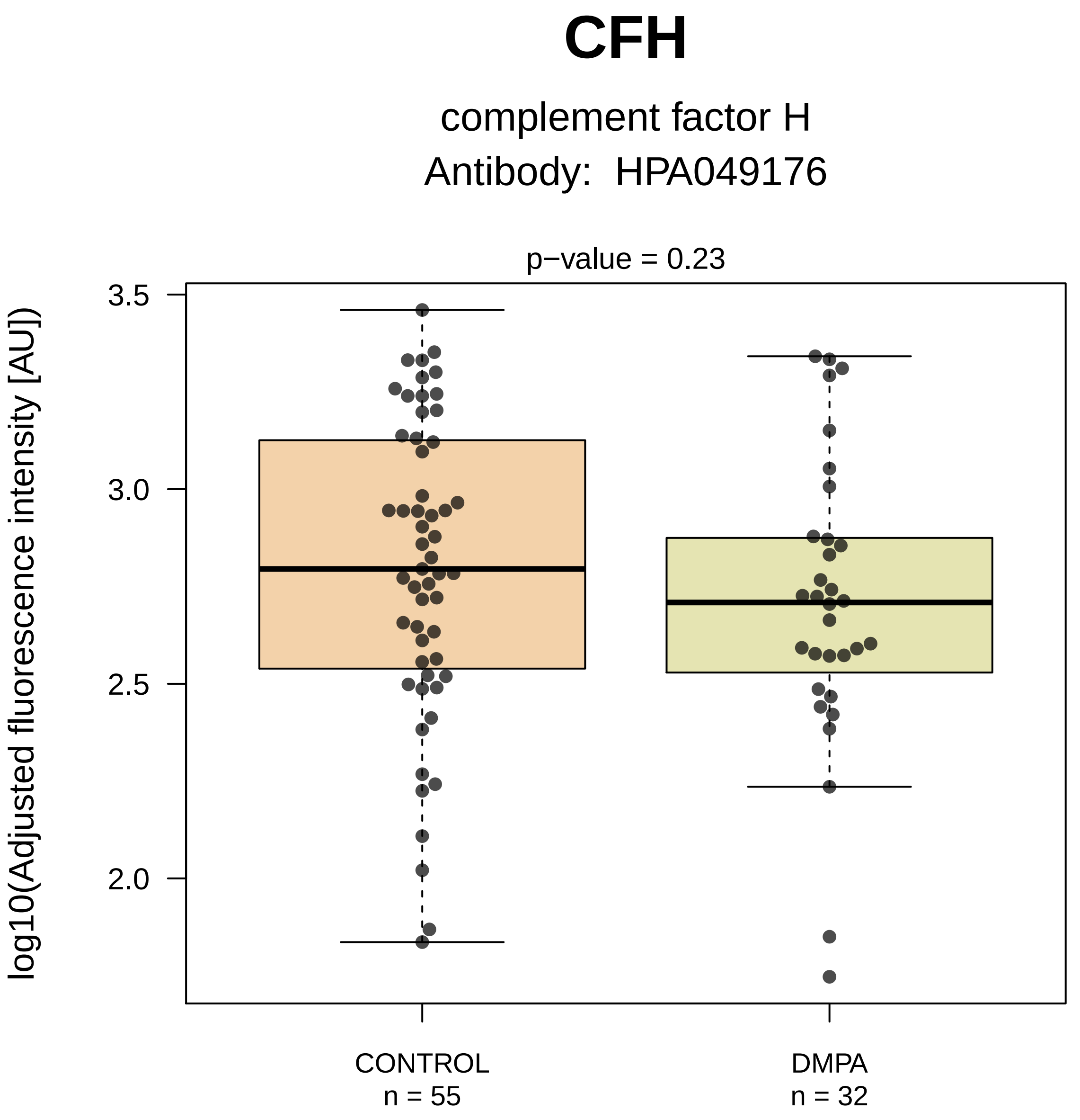
CFH
complement factor H
Antibody: HPA049176
p−value = 0.23
log10(Adjusted fluorescence intensity [AU])
3.5
3.0
2.5
2.0
CONTROL
n = 55
DMPA
n = 32

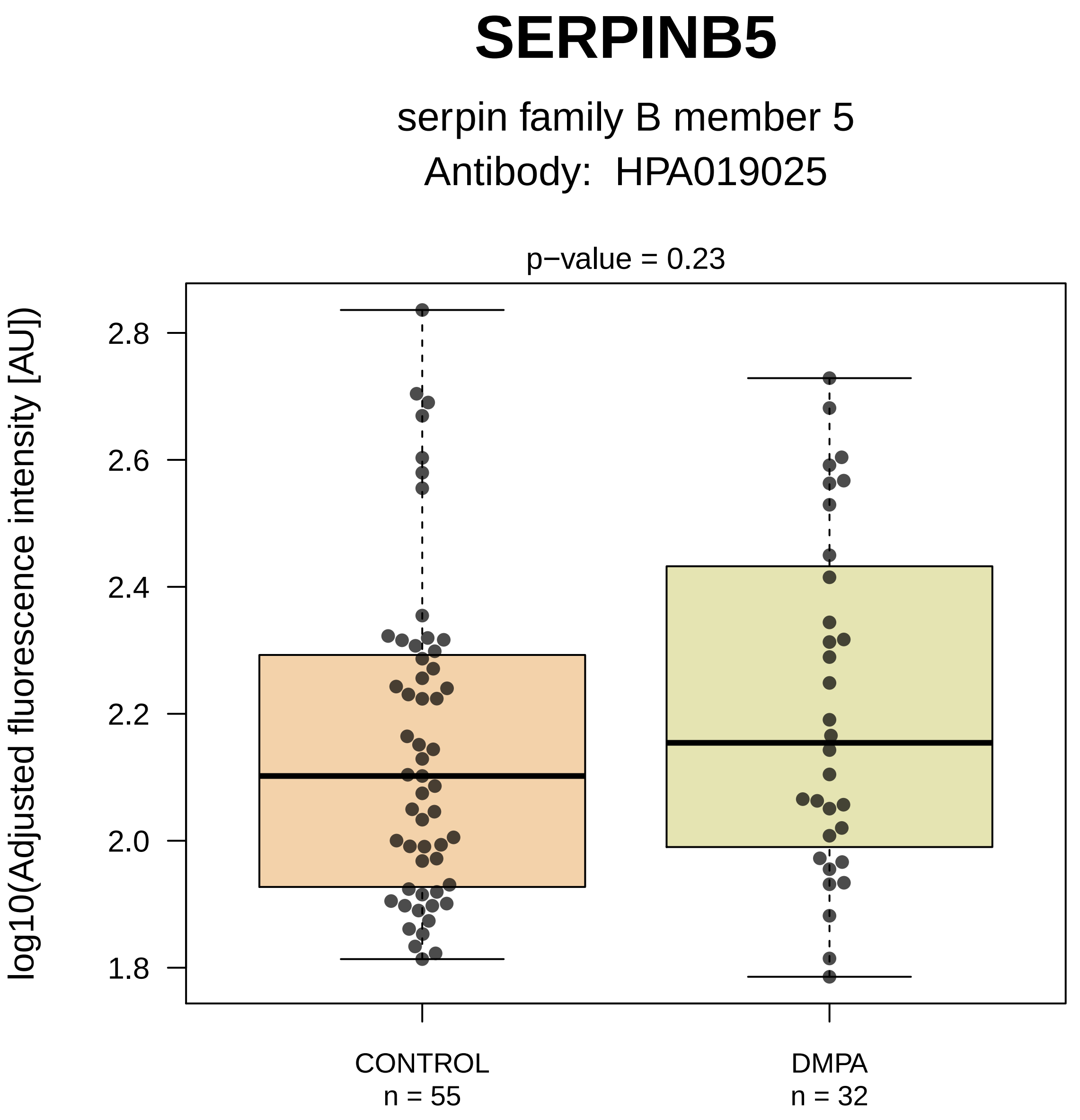
SERPINB5
serpin family B member 5
Antibody: HPA019025
p−value = 0.23
log10(Adjusted fluorescence intensity [AU])
2.8
2.6
2.4
2.2
2.0
1.8
CONTROL
n = 55
DMPA
n = 32

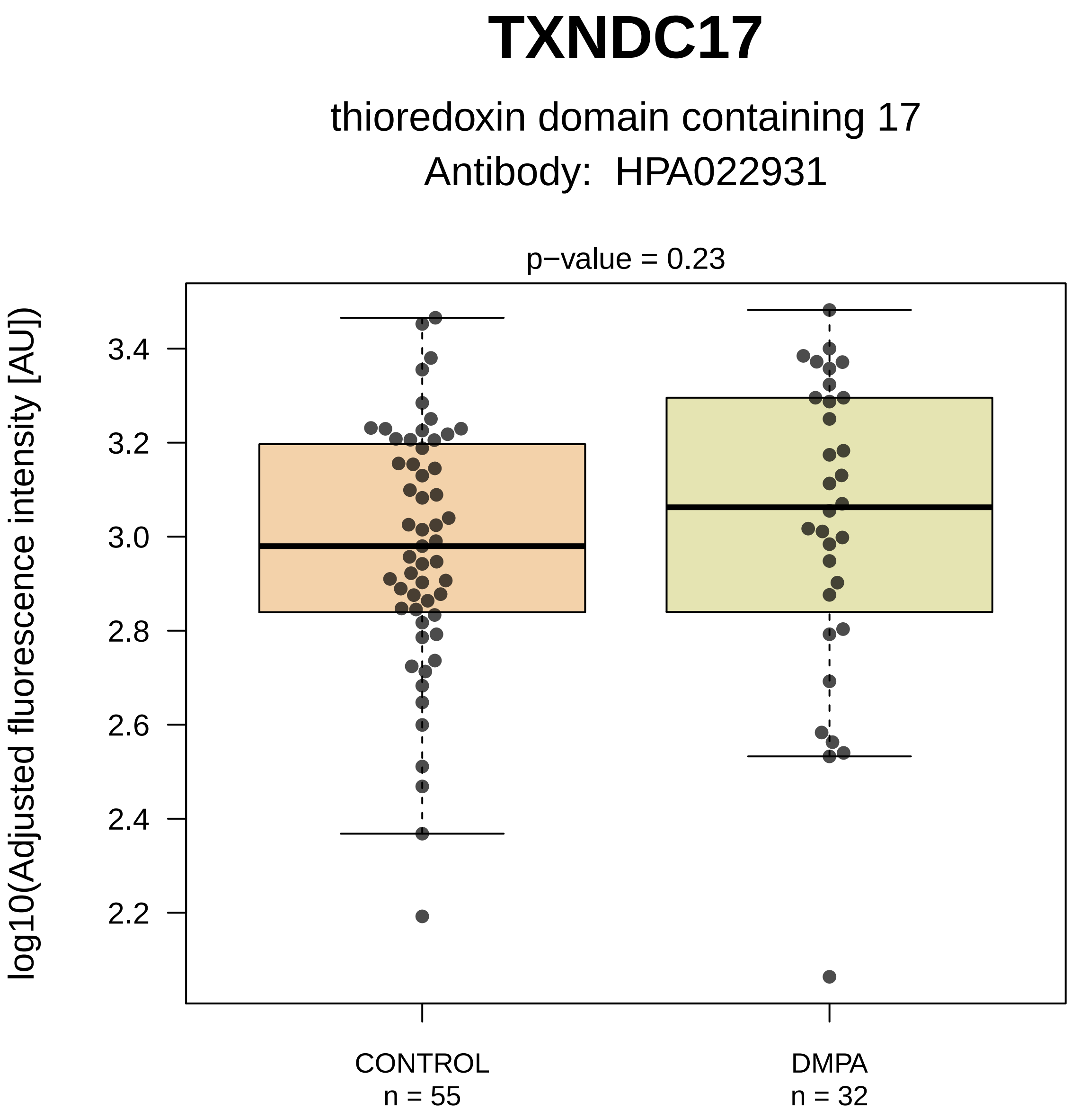

TXNDC17
thioredoxin domain containing 17
Antibody: HPA022931
p−value = 0.23
log10(Adjusted fluorescence intensity [AU])
3.4
3.2
3.0
2.8
2.6
2.4
2.2
CONTROL
n = 55
DMPA
n = 32

MUC5AC

mucin 5AC, oligomeric mucus/gel−forming
Antibody: HPA040615

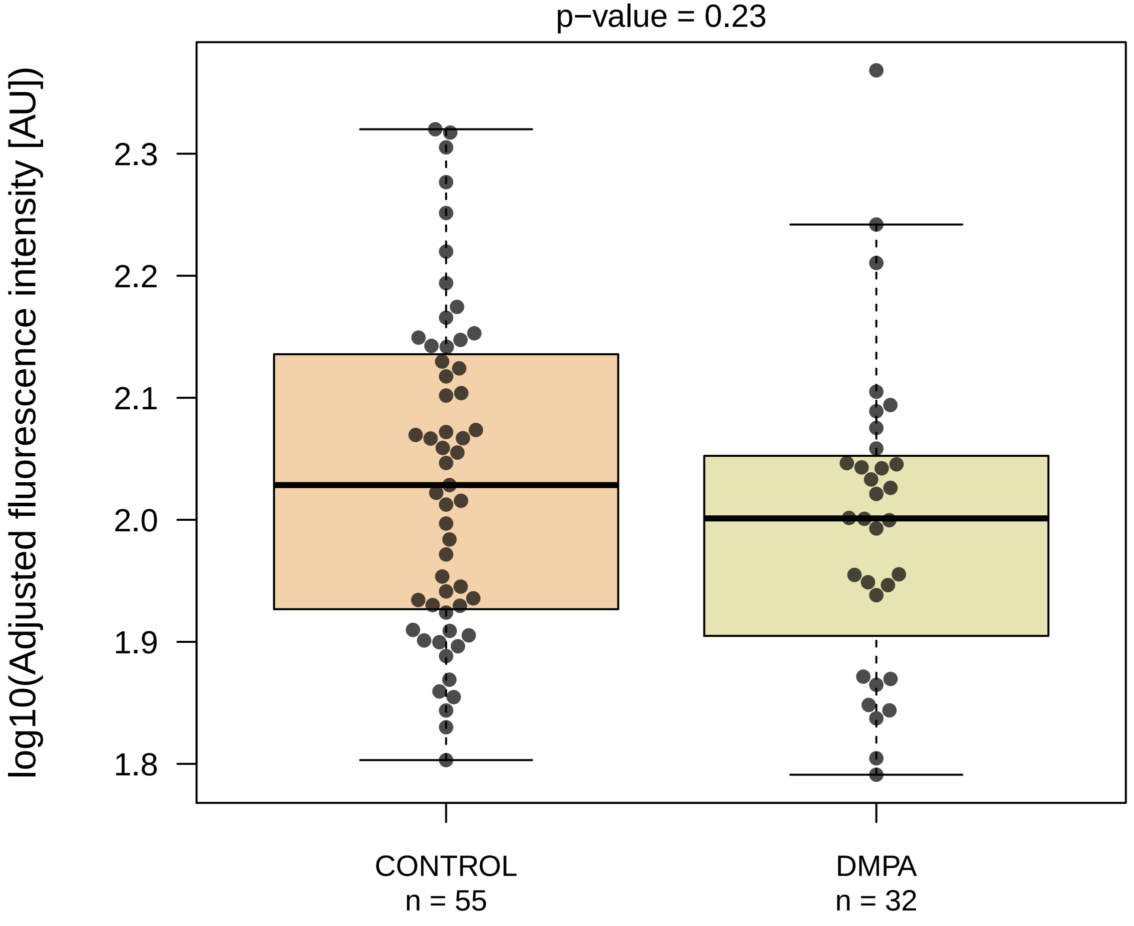

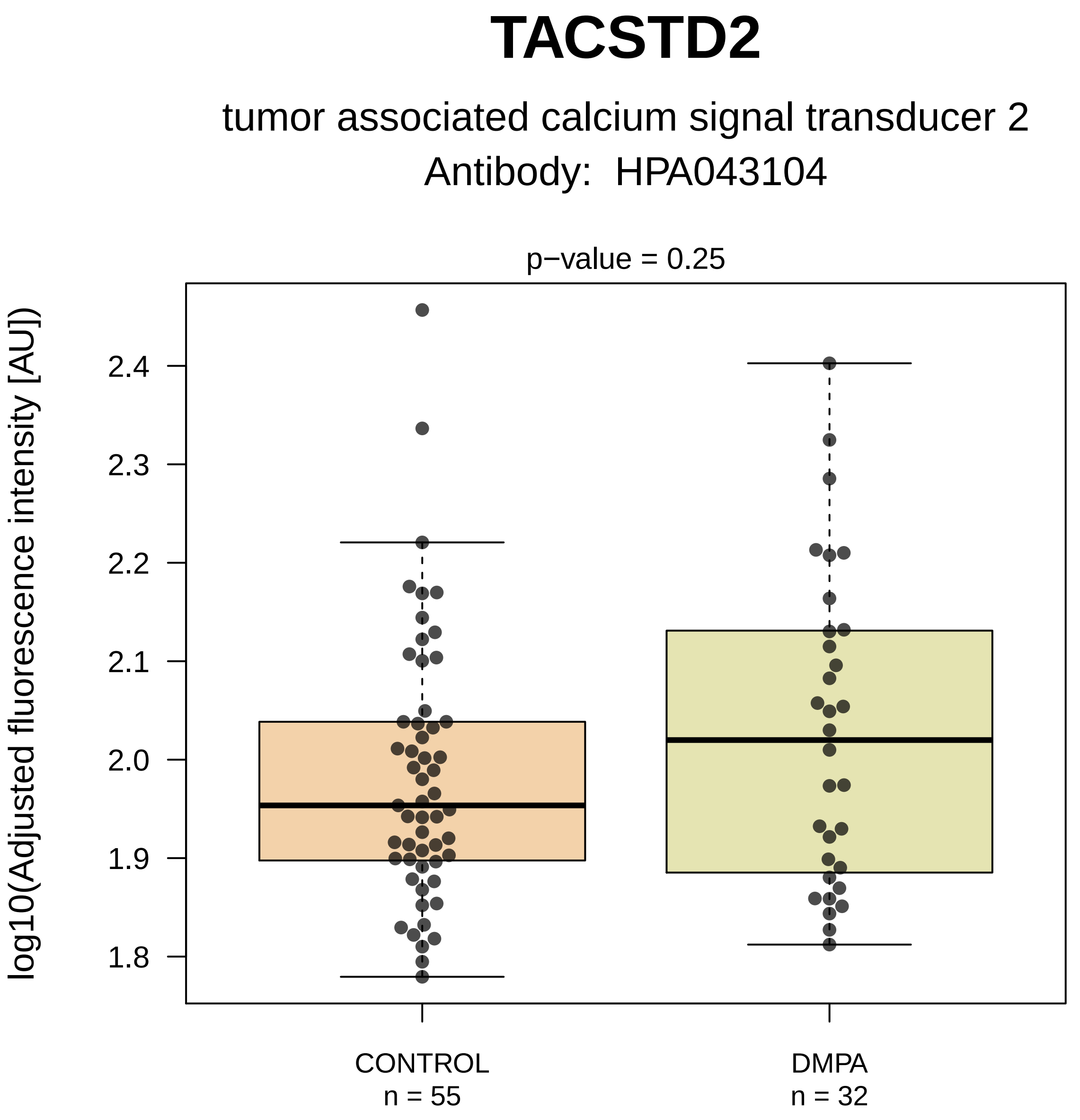
TACSTD2
tumor associated calcium signal transducer 2
Antibody: HPA043104
p−value = 0.25
log10(Adjusted fluorescence intensity [AU])
2.4
2.3
2.2
2.1
2.0
1.9
1.8
CONTROL
n = 55
DMPA
n = 32

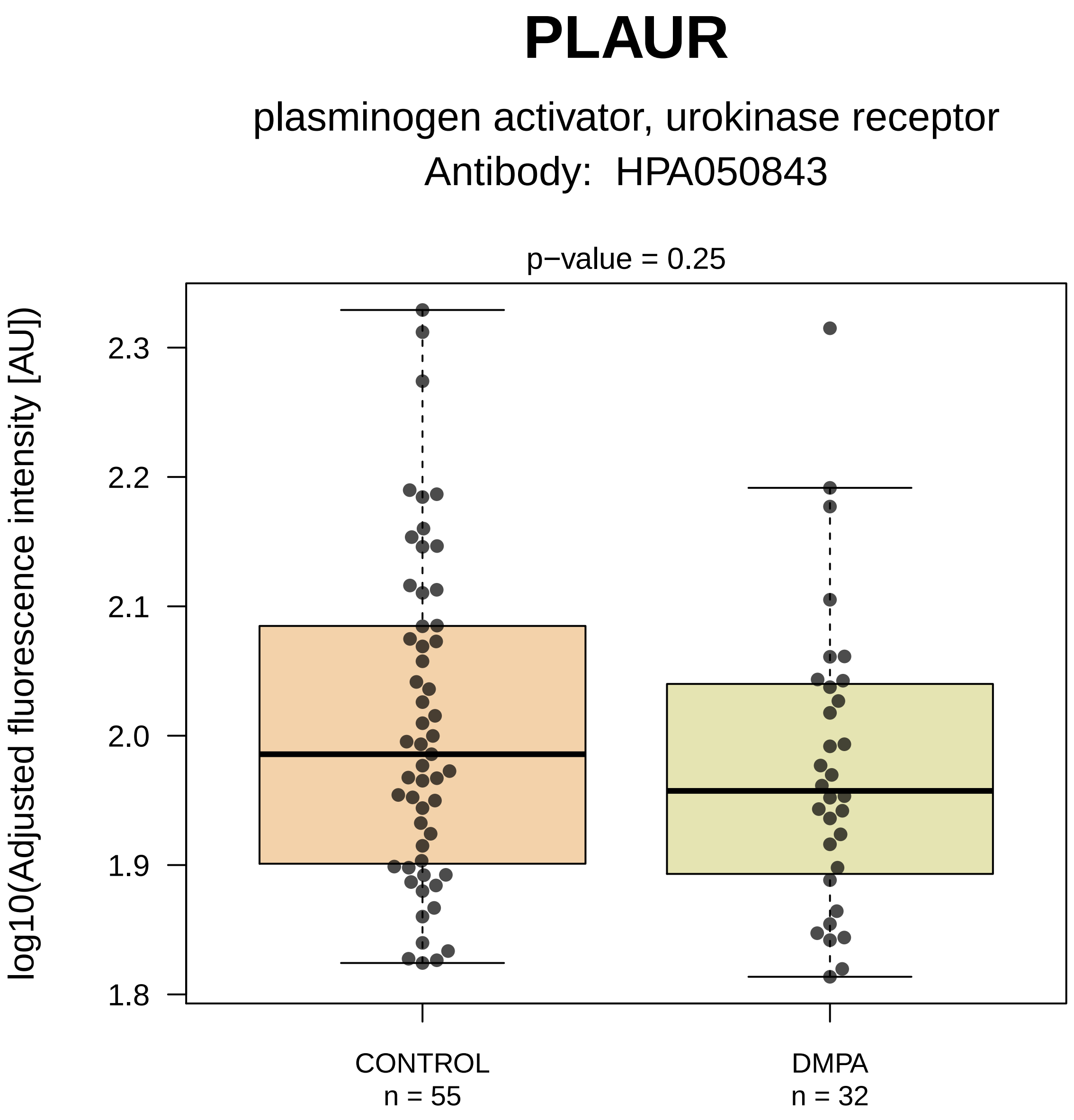
PLAUR
plasminogen activator, urokinase receptor
Antibody: HPA050843
p−value = 0.25
log10(Adjusted fluorescence intensity [AU])
2.3
2.2
2.1
2.0
1.9
1.8
CONTROL
n = 55
DMPA
n = 32

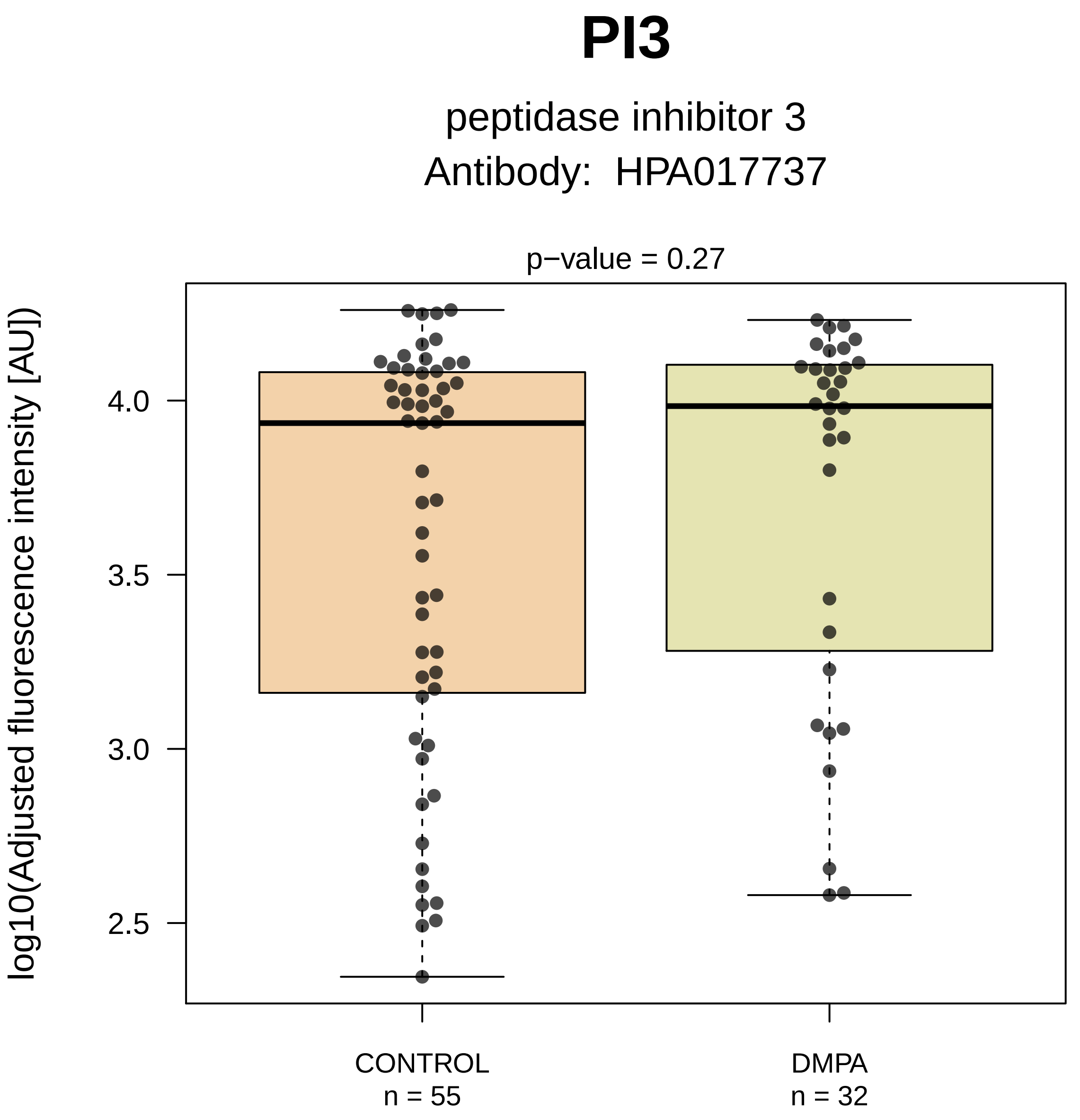
PI3
peptidase inhibitor 3
Antibody: HPA017737
p−value = 0.27
log10(Adjusted fluorescence intensity [AU])
4.0
3.5
3.0
2.5
CONTROL
n = 55
DMPA
n = 32

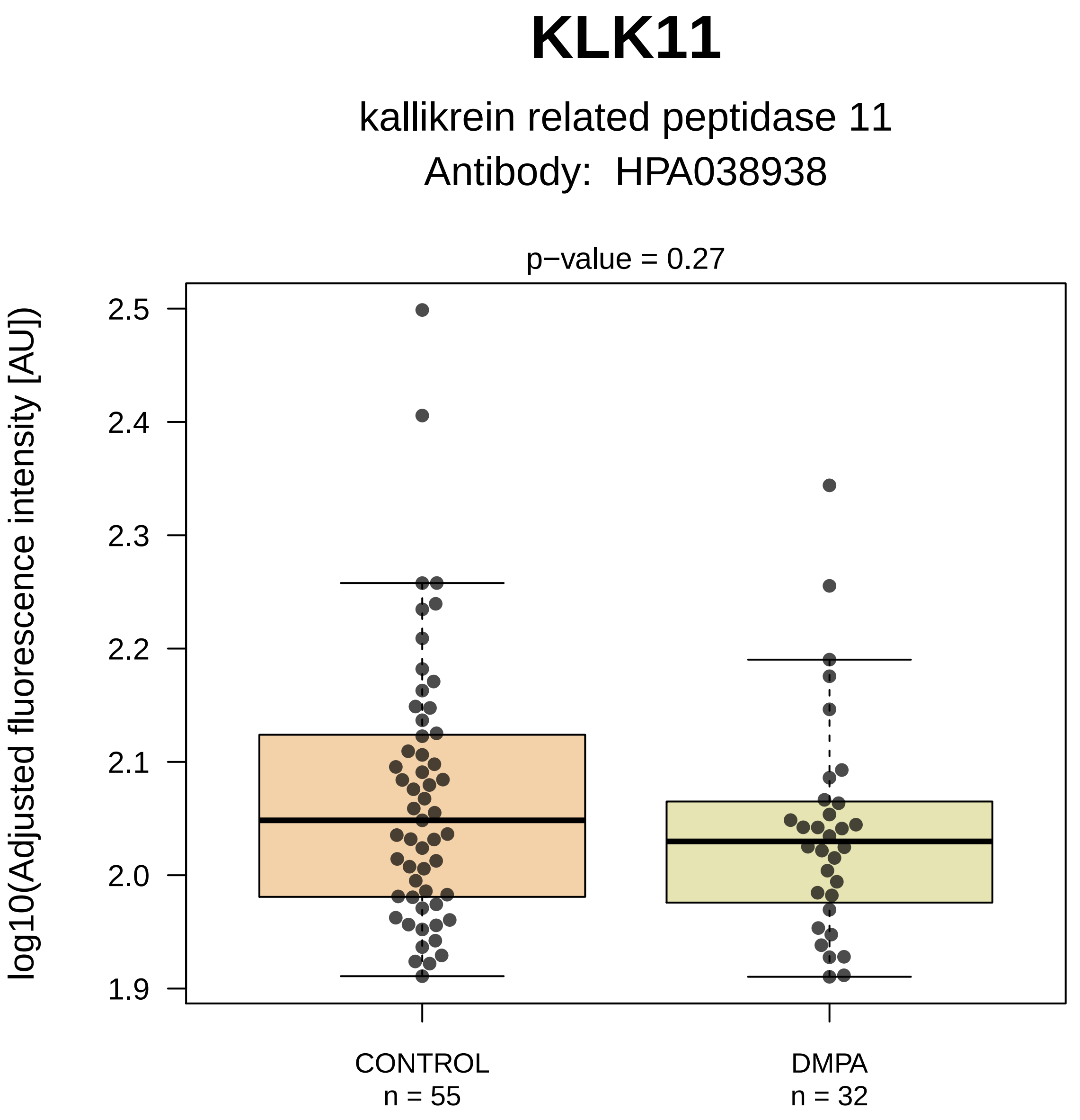
KLK11
kallikrein related peptidase 11
Antibody: HPA038938
p−value = 0.27
log10(Adjusted fluorescence intensity [AU])
2.5
2.4
2.3
2.2
2.1
2.0
1.9
CONTROL
n = 55
DMPA
n = 32

SERPING1

serpin family G member 1

Antibody: HPA048738

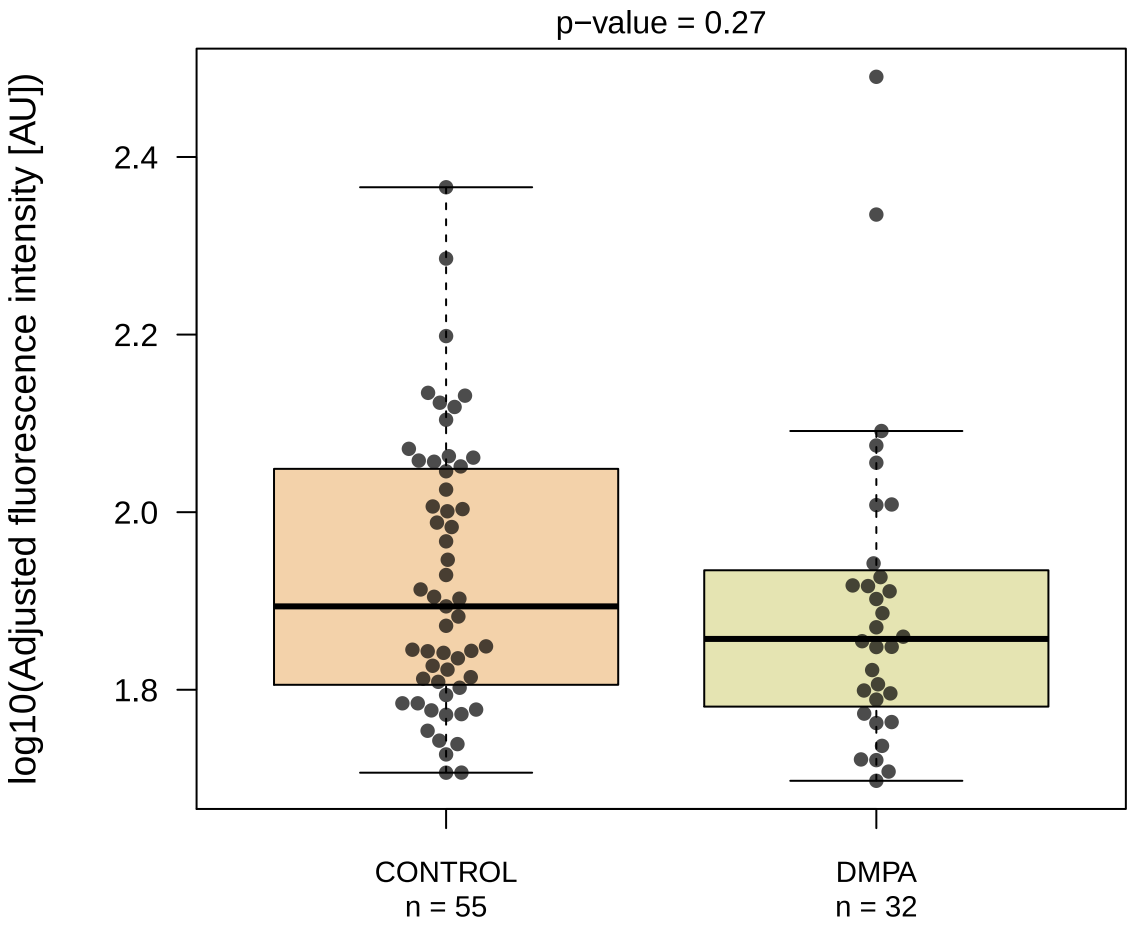

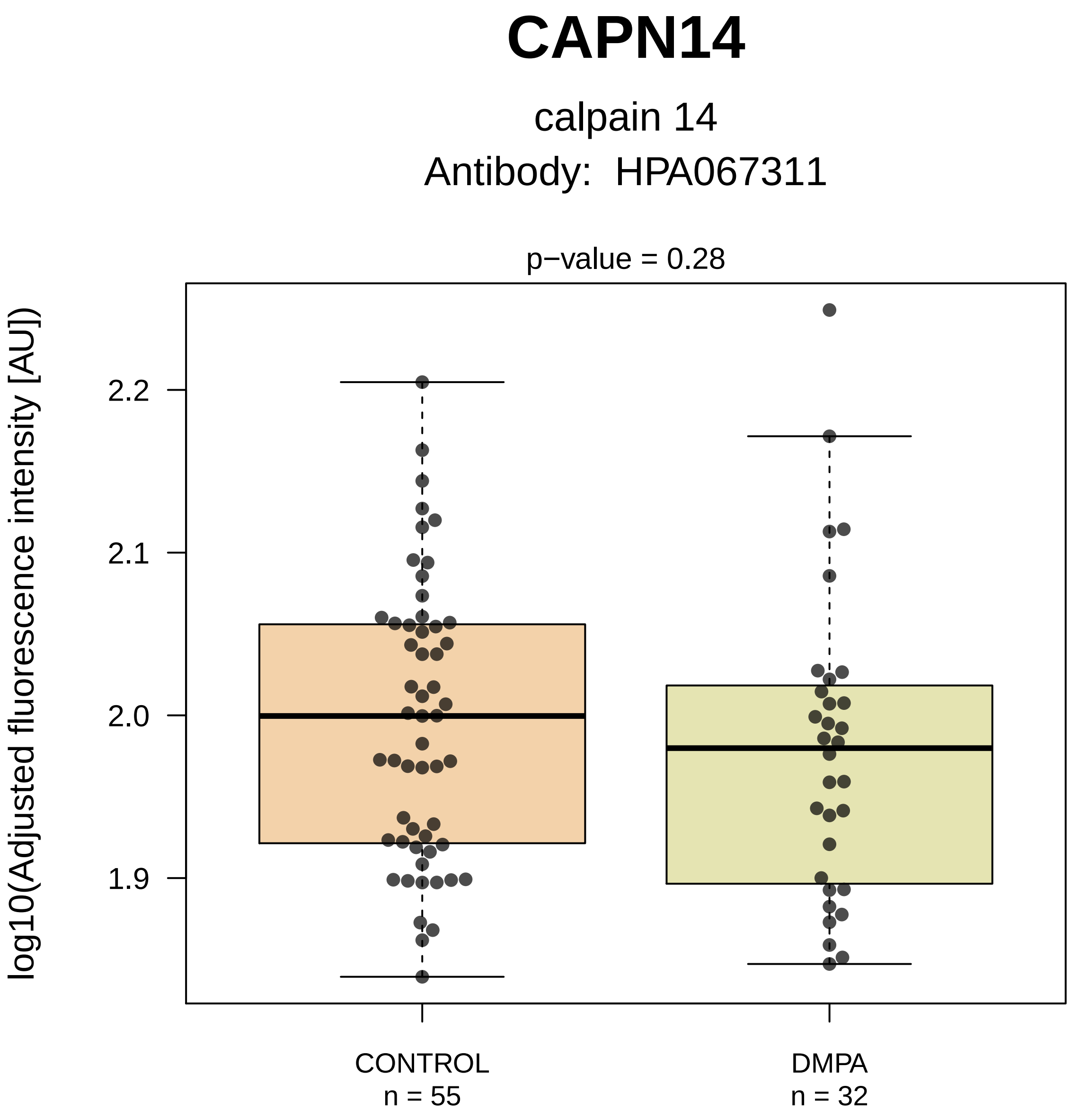

CAPN14
calpain 14
Antibody: HPA067311
p−value = 0.28
log10(Adjusted fluorescence intensity [AU])
2.2
2.1
2.0
1.9
CONTROL
n = 55
DMPA
n = 32

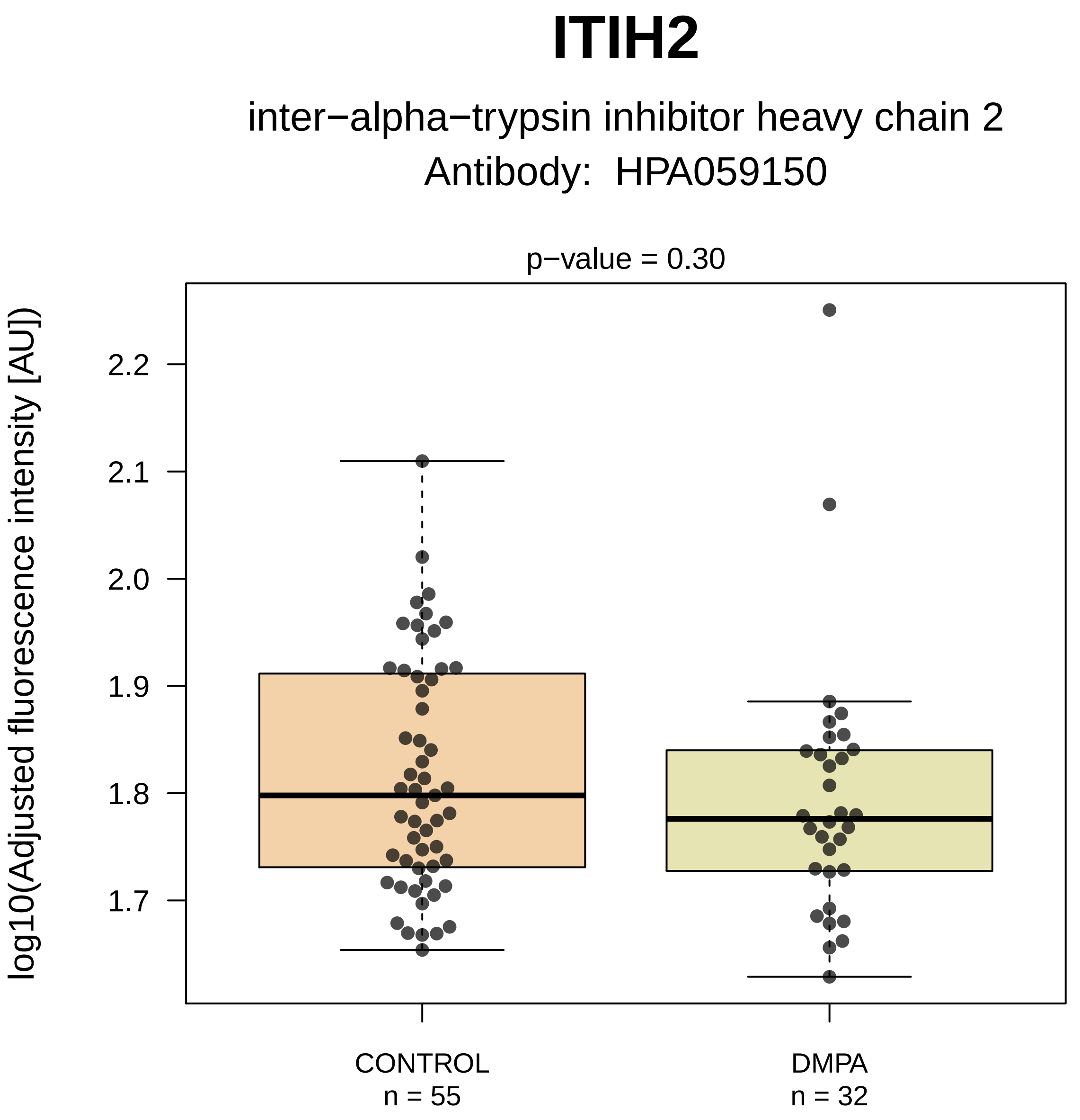
ITIH2
inter−alpha−trypsin inhibitor heavy chain 2
Antibody: HPA059150
p−value = 0.30
log10(Adjusted fluorescence intensity [AU])
2.2
2.1
2.0
1.9
1.8
1.7
CONTROL
n = 55
DMPA
n = 32

DEFA3,DEFA1,DEFA1B

defensin alpha 3,defensin alpha 1,defensin alpha 1B

Antibody: HPA044693

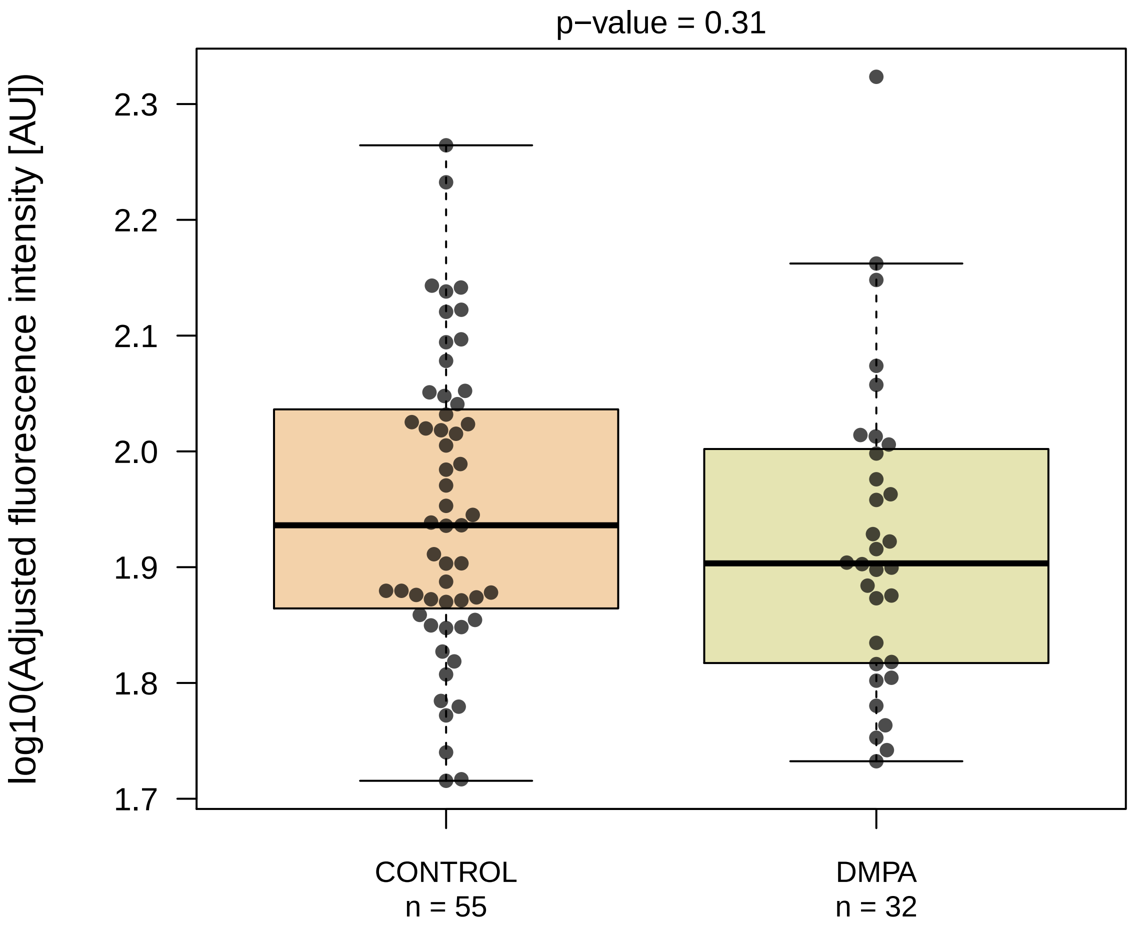

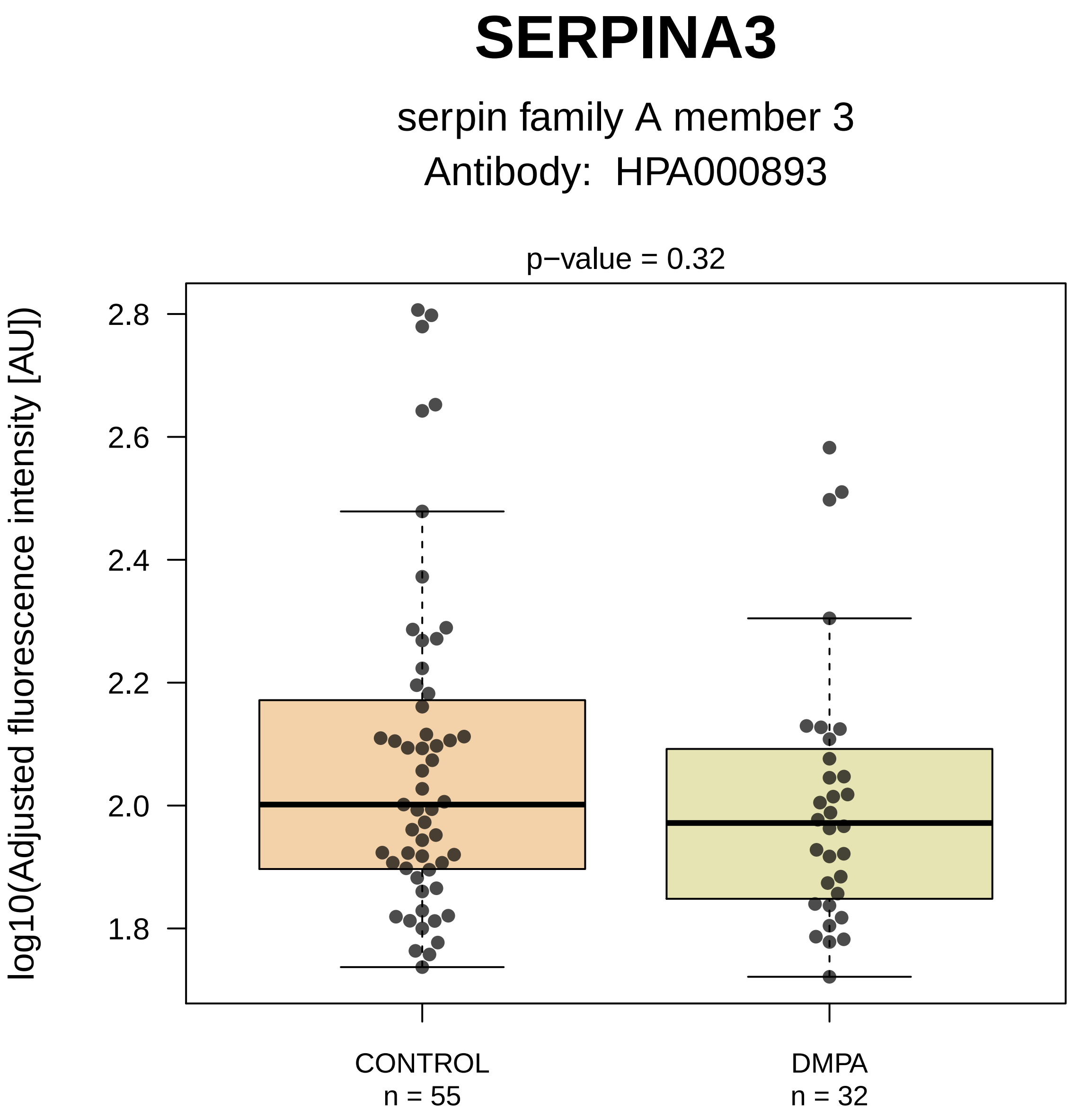
SERPINA3
serpin family A member 3
Antibody: HPA000893
p−value = 0.32
log10(Adjusted fluorescence intensity [AU])
2.8
2.6
2.4
2.2
2.0
1.8
CONTROL
n = 55
DMPA
n = 32

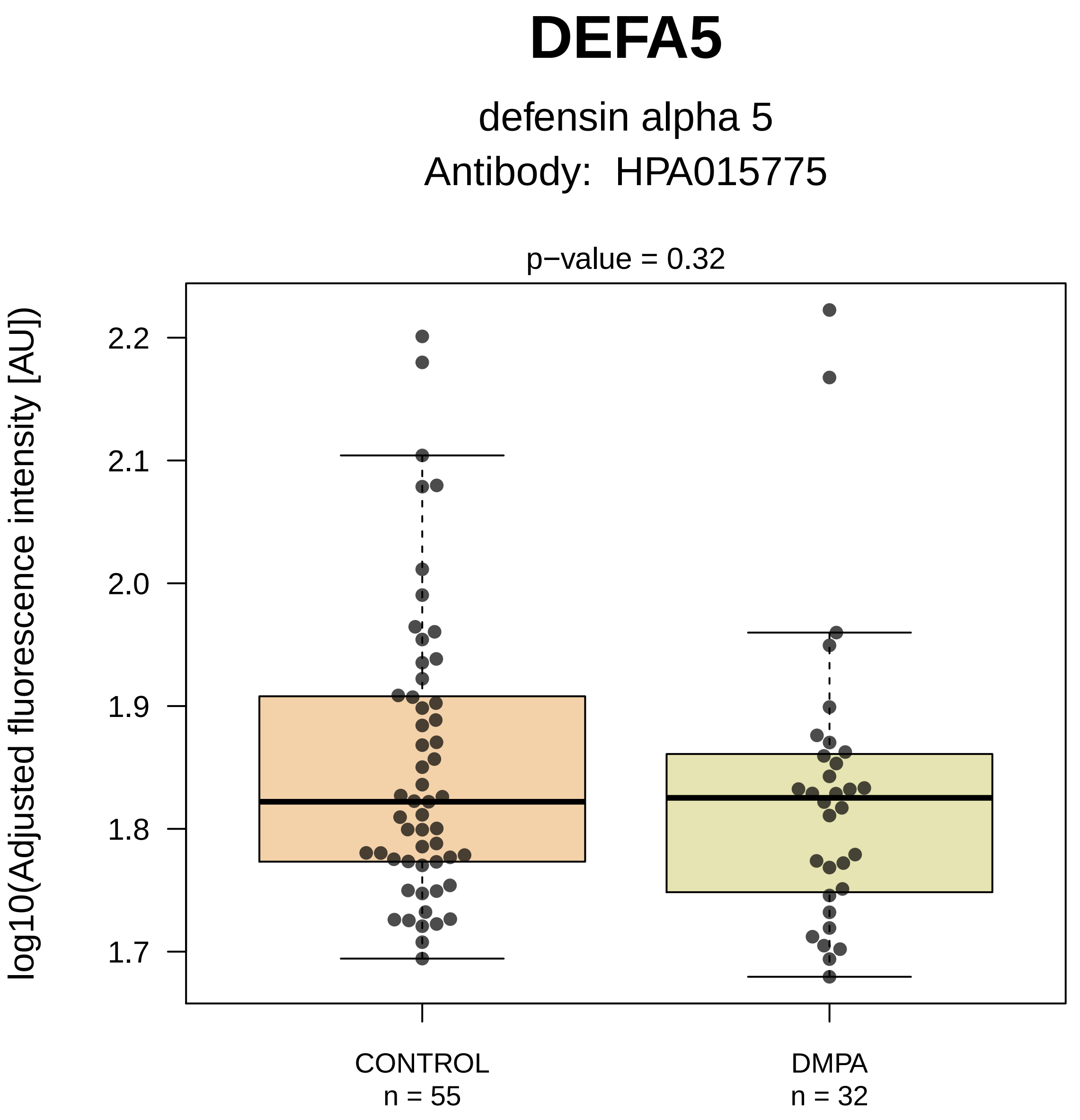
DEFA5
defensin alpha 5
Antibody: HPA015775
p−value = 0.32
log10(Adjusted fluorescence intensity [AU])
2.2
2.1
2.0
1.9
1.8
1.7
CONTROL
n = 55
DMPA
n = 32

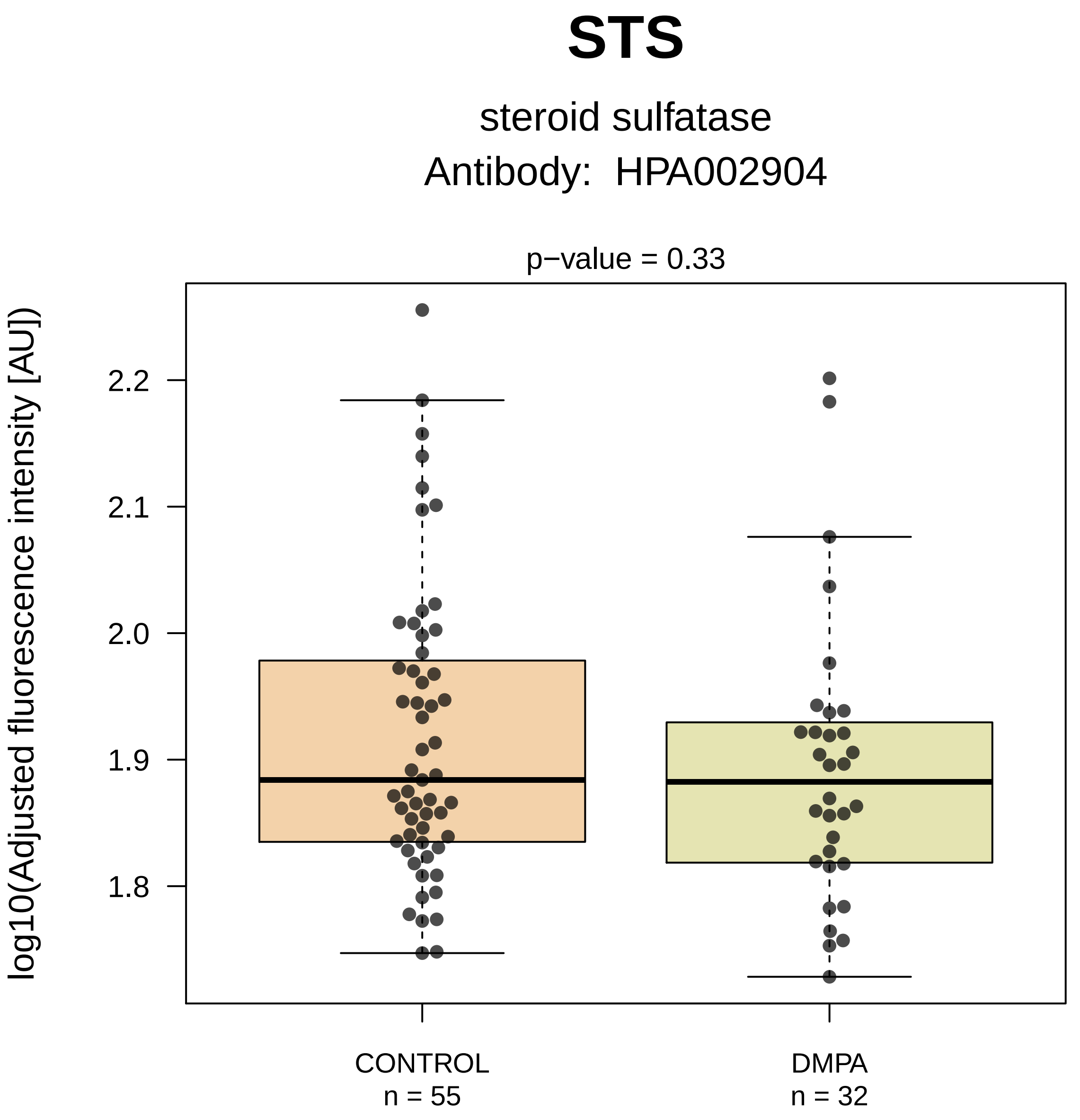
STS
steroid sulfatase
Antibody: HPA002904
p−value = 0.33
log10(Adjusted fluorescence intensity [AU])
2.2
2.1
2.0
1.9
1.8
CONTROL
n = 55
DMPA
n = 32

KRT4

keratin 4

Antibody: HPA034881

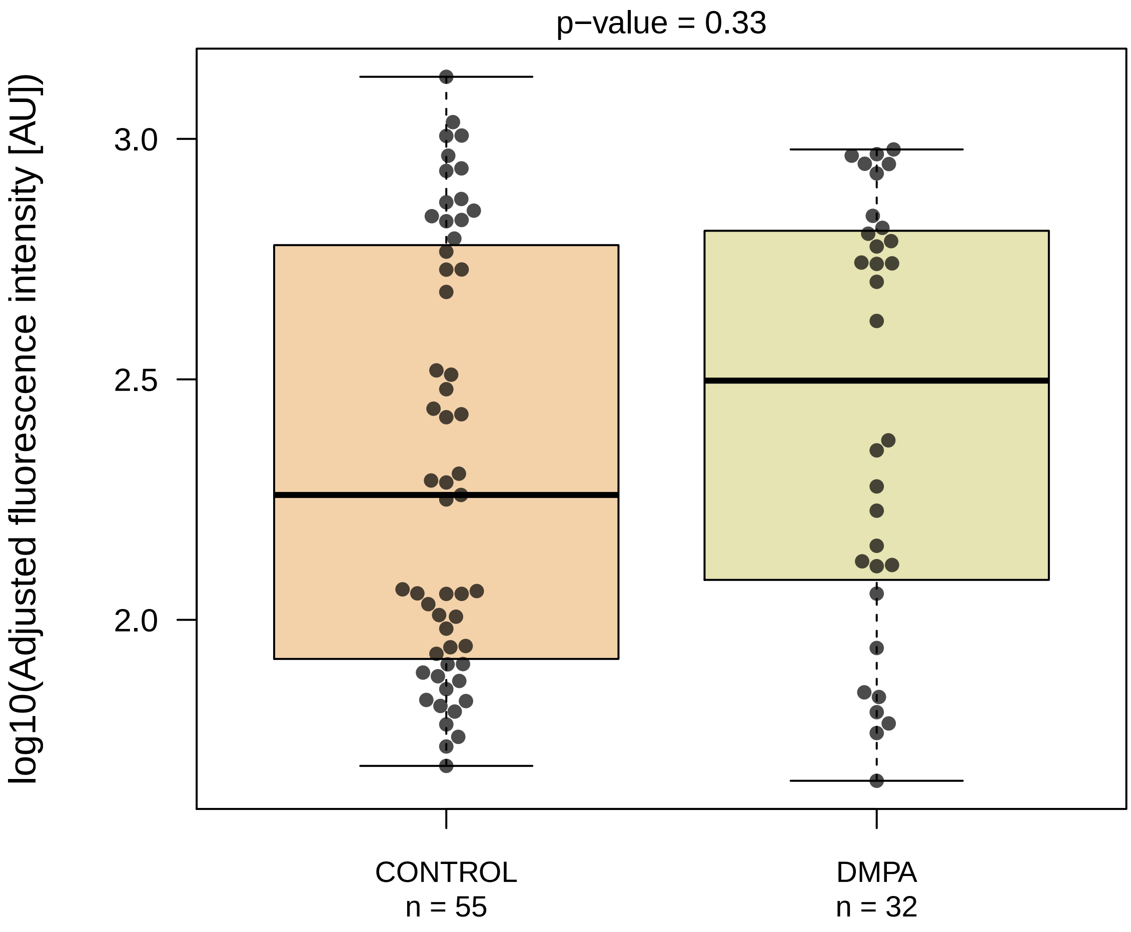

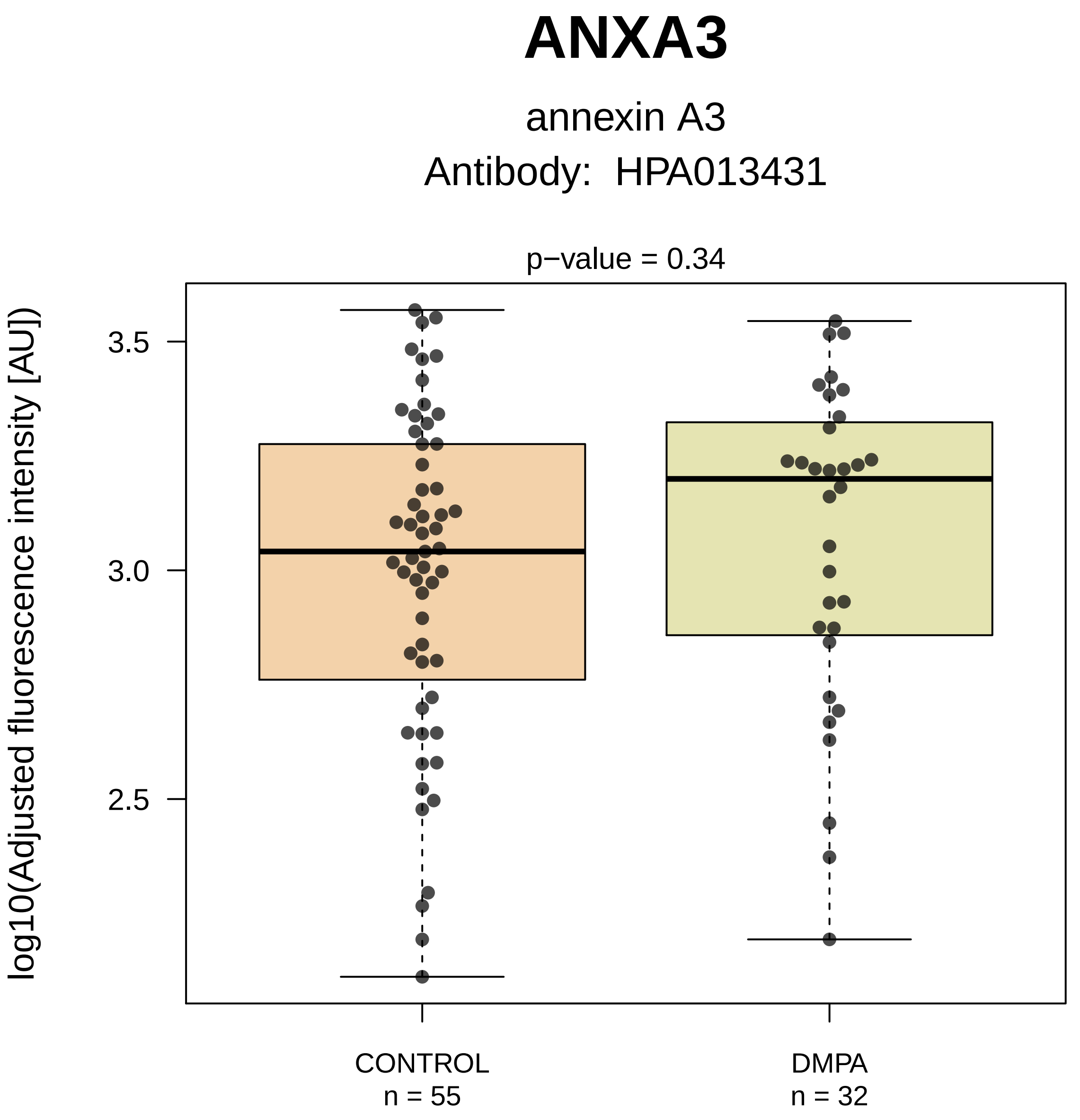
ANXA3
annexin A3
Antibody: HPA013431
p−value = 0.34
log10(Adjusted fluorescence intensity [AU])
3.5
3.0
2.5
CONTROL
n = 55
DMPA
n = 32

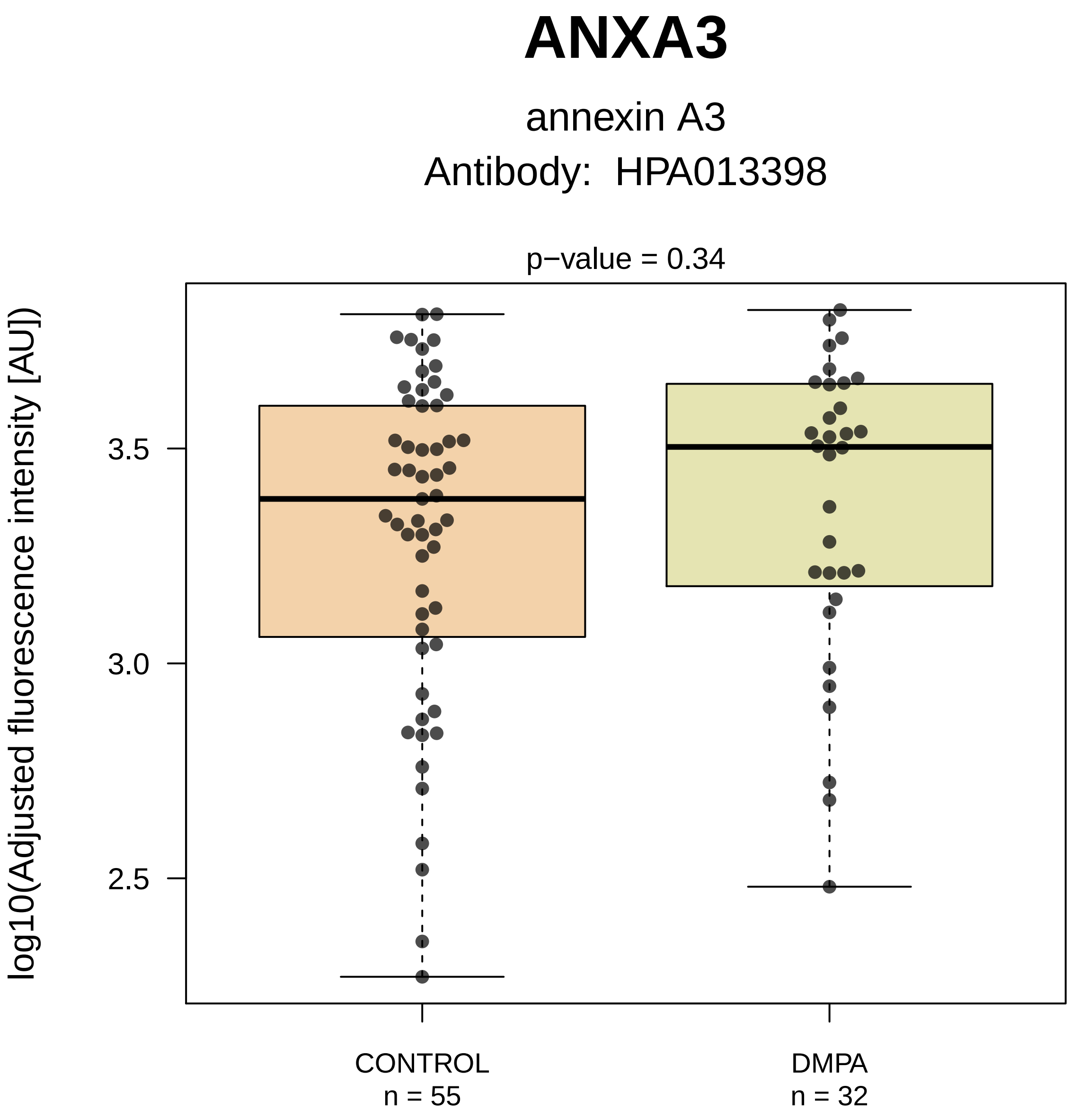
ANXA3
annexin A3
Antibody: HPA013398
p−value = 0.34
log10(Adjusted fluorescence intensity [AU])
3.5
3.0
2.5
CONTROL
n = 55
DMPA
n = 32

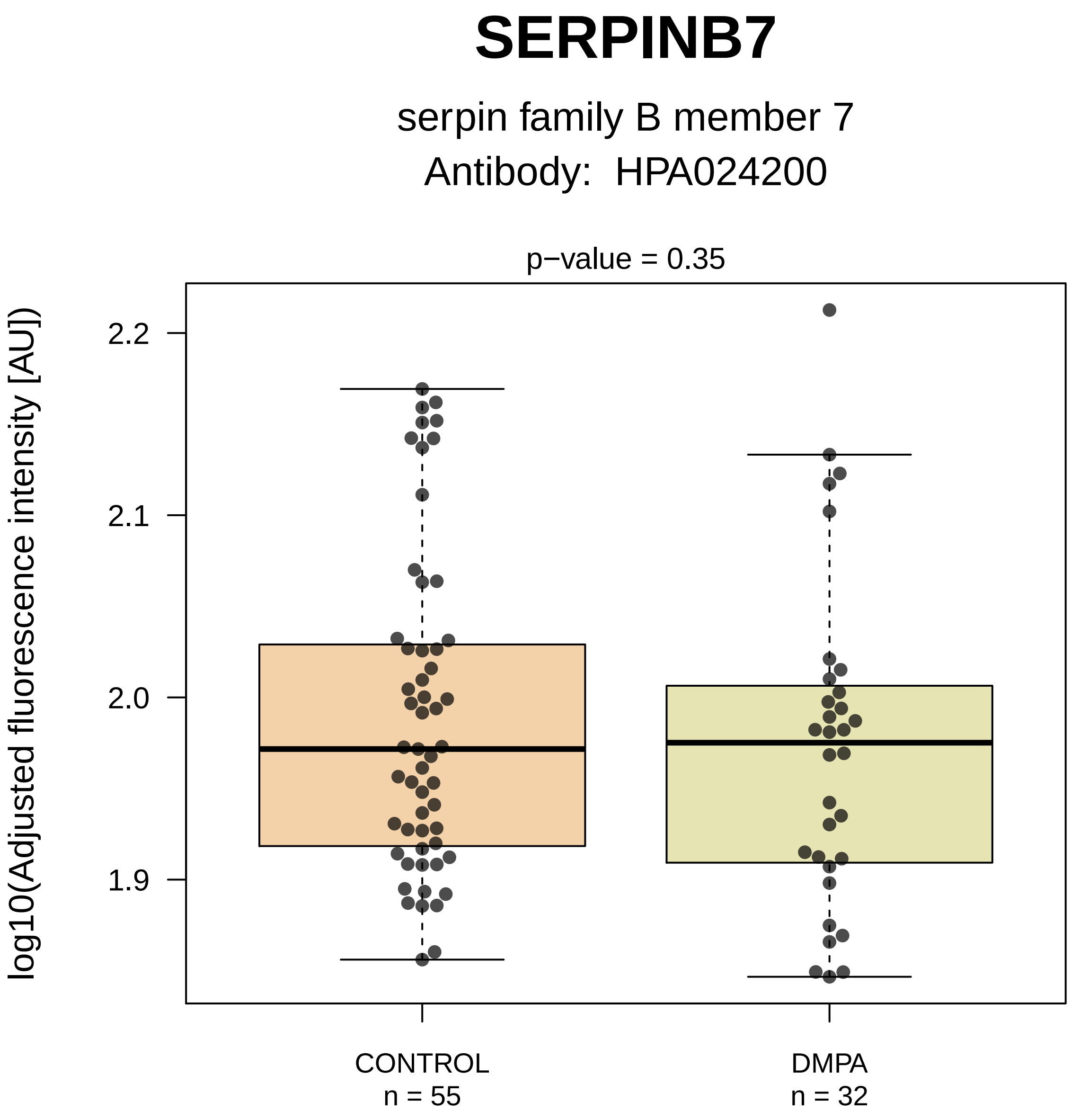
SERPINB7
serpin family B member 7
Antibody: HPA024200
p−value = 0.35
log10(Adjusted fluorescence intensity [AU])
2.2
2.1
2.0
1.9
CONTROL
n = 55
DMPA
n = 32

S100A12

S100 calcium binding protein A12

Antibody: HPA002881

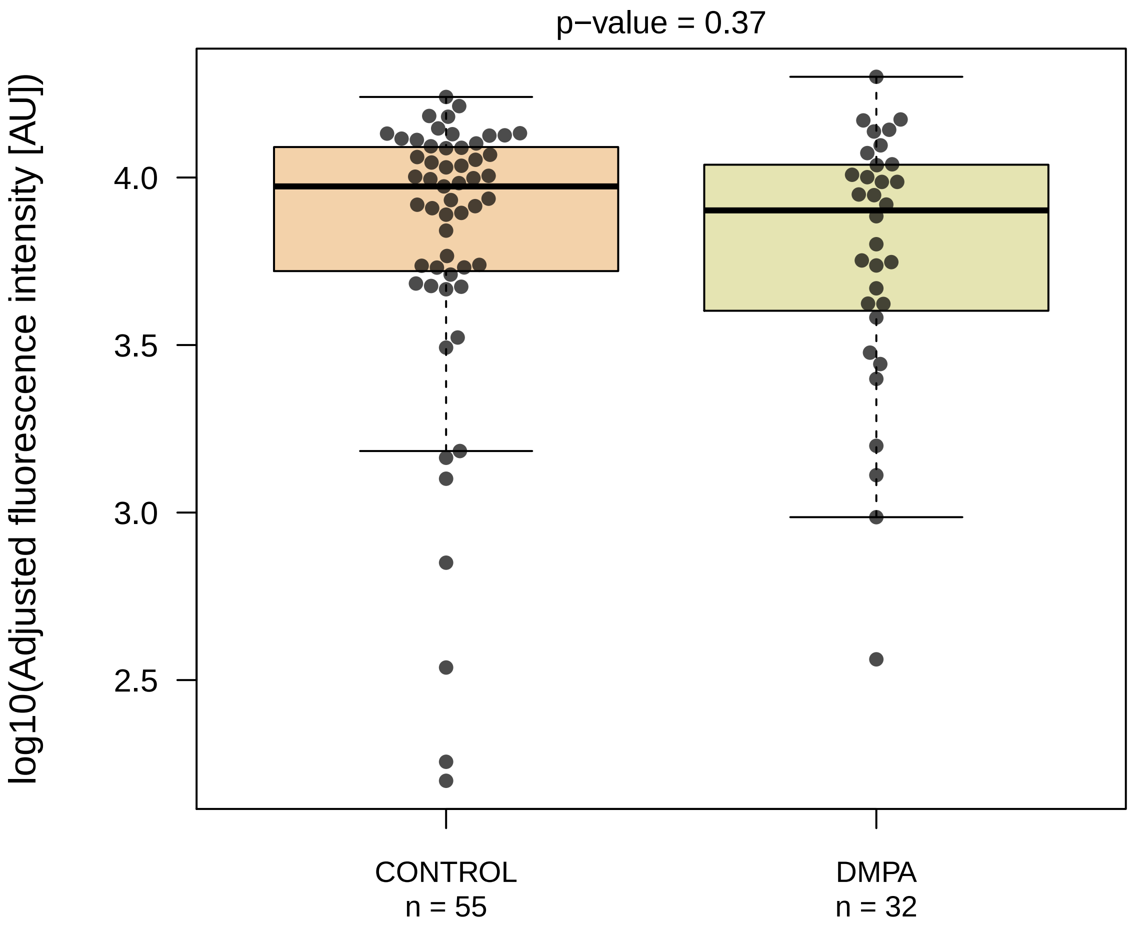

MSN

moesin

Antibody: HPA011135

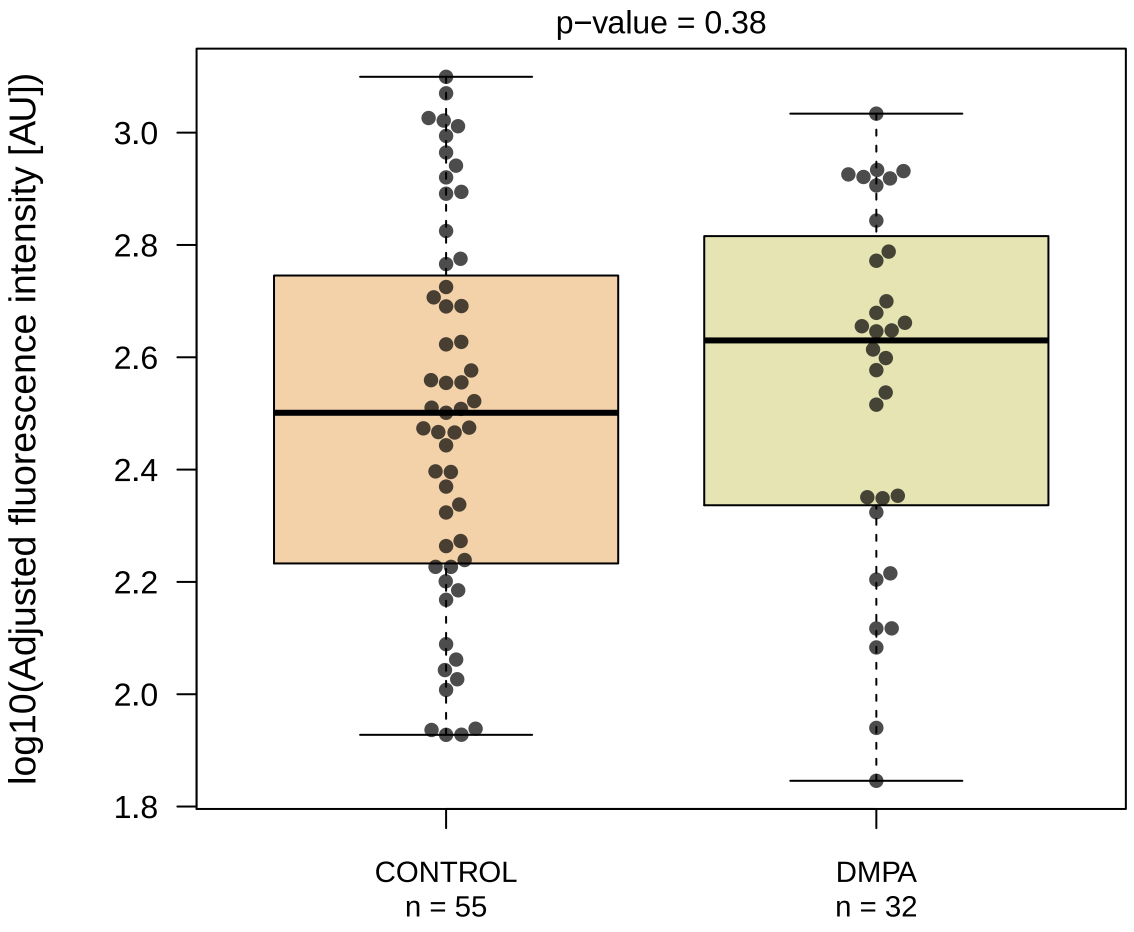

FGA

fibrinogen alpha chain
Antibody: HPA064755

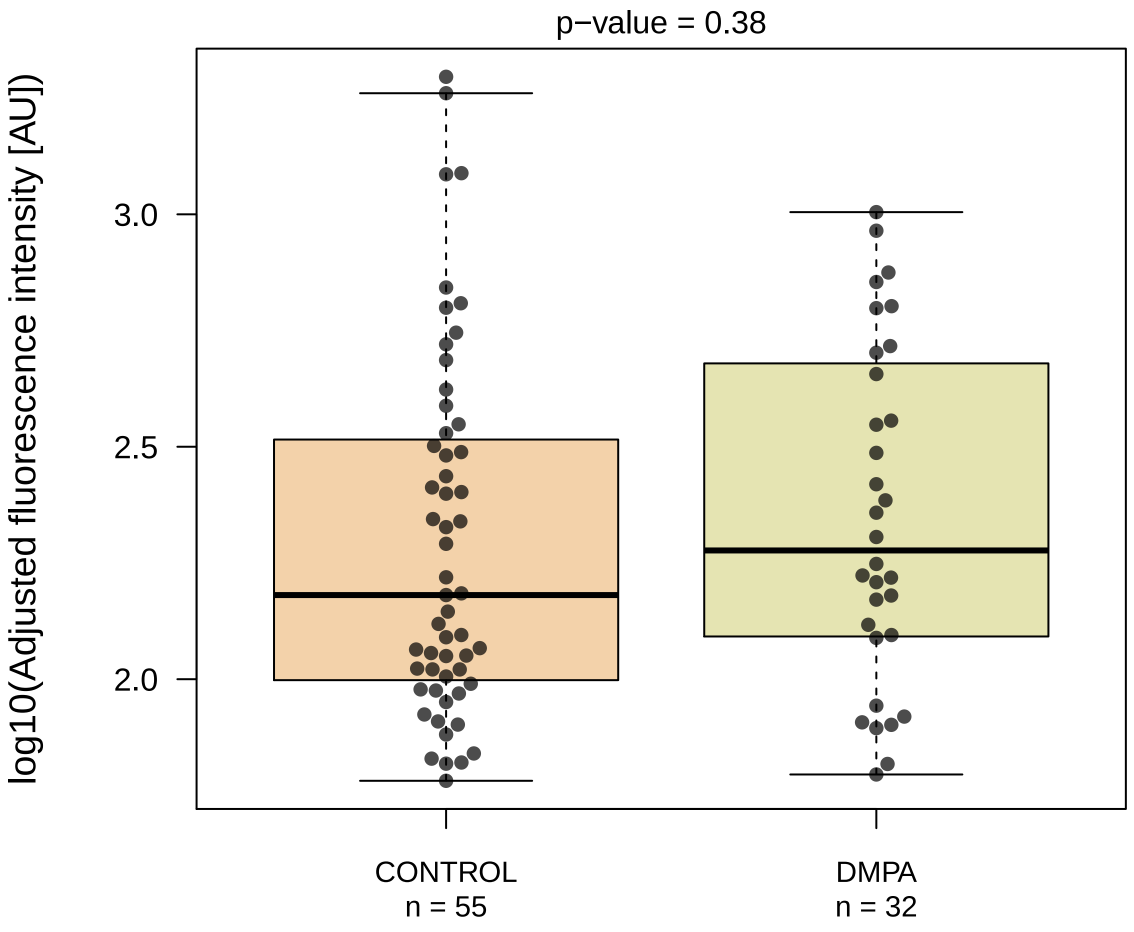

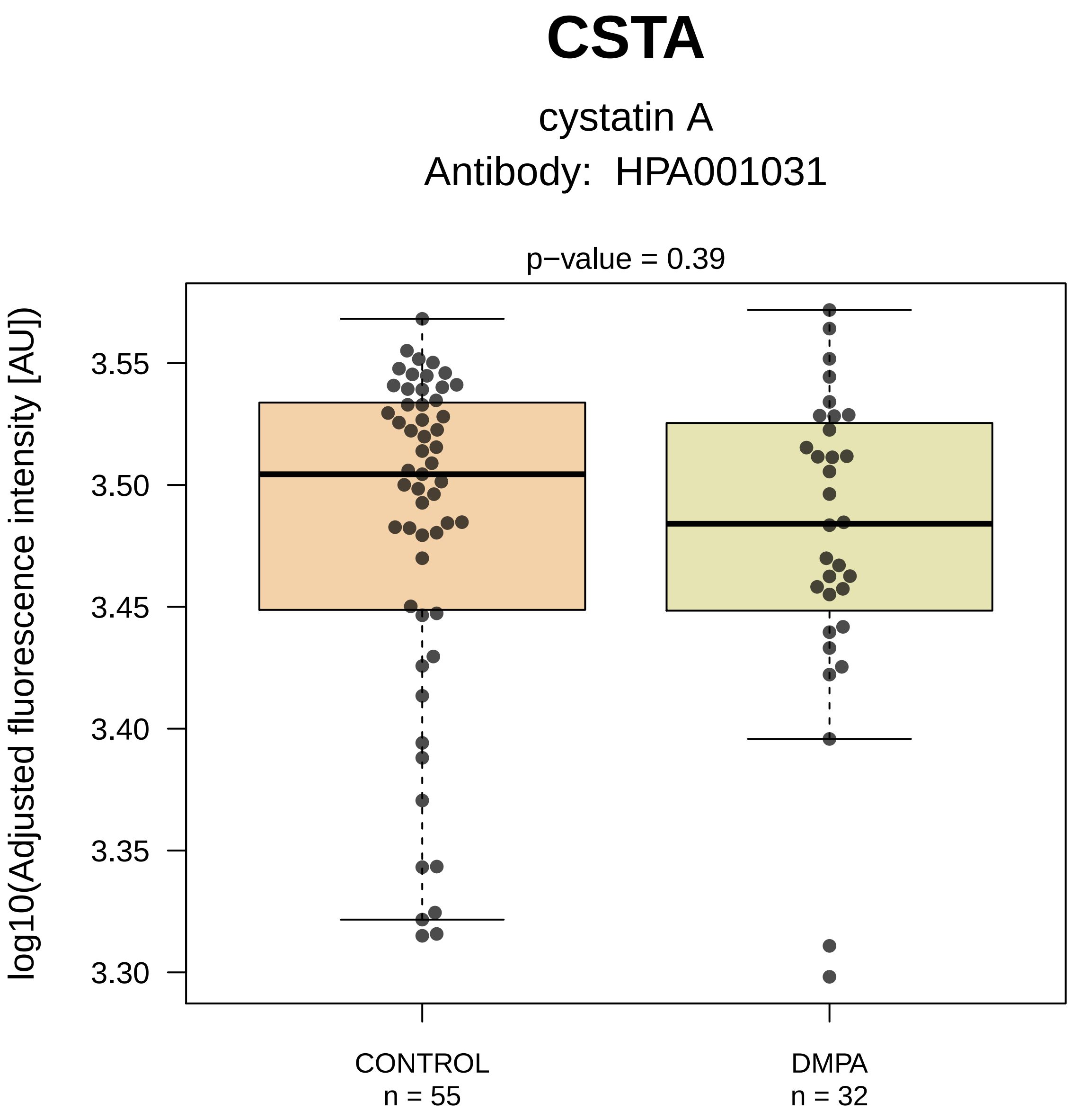
CSTA
cystatin A
Antibody: HPA001031
p−value = 0.39
log10(Adjusted fluorescence intensity [AU])
3.55
3.50
3.45
3.40
3.35
3.30
CONTROL
n = 55
DMPA
n = 32

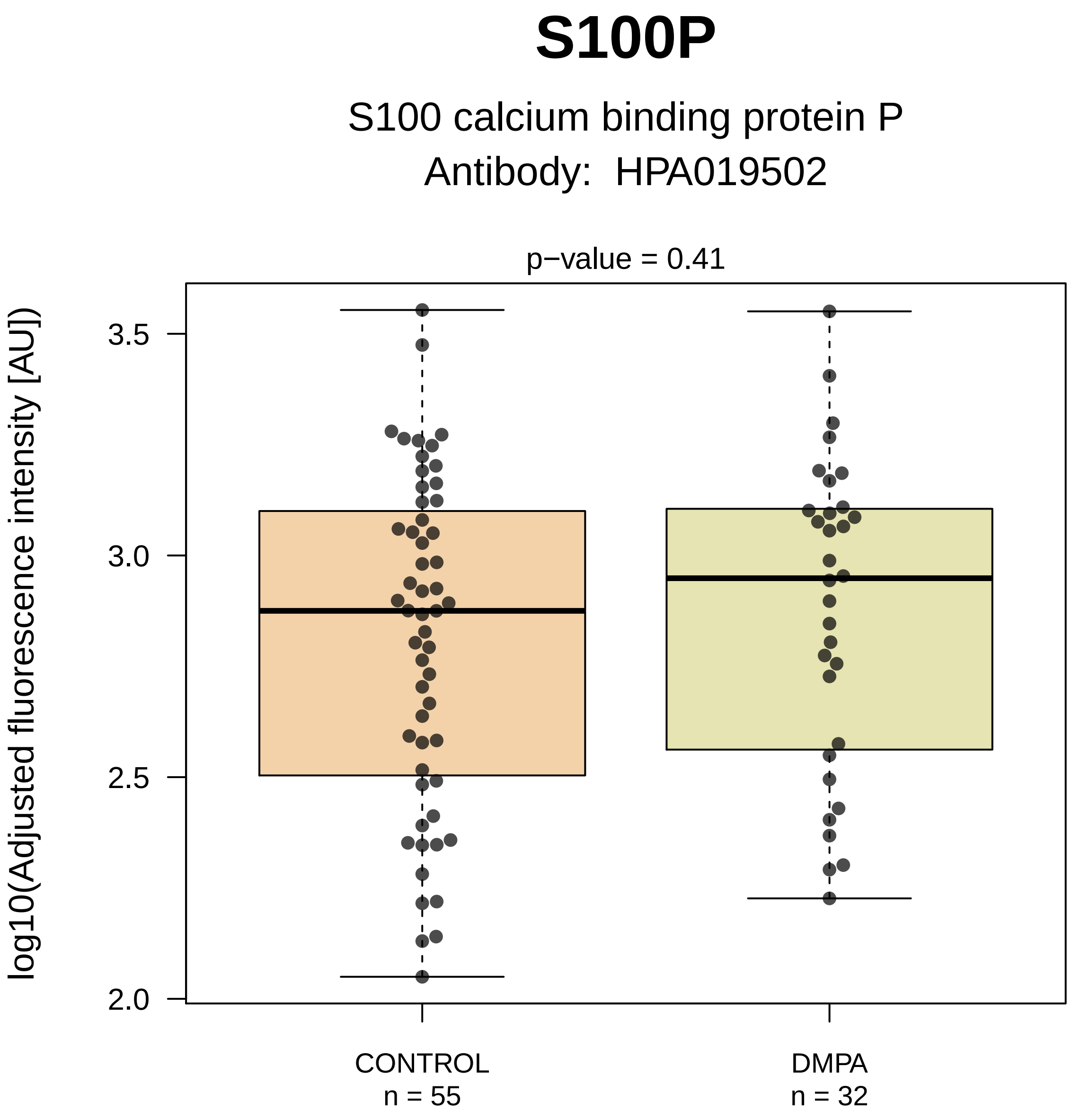

S100P
S100 calcium binding protein P
Antibody: HPA019502
p−value = 0.41
log10(Adjusted fluorescence intensity [AU])
3.5
3.0
2.5
2.0
CONTROL
n = 55
DMPA
n = 32

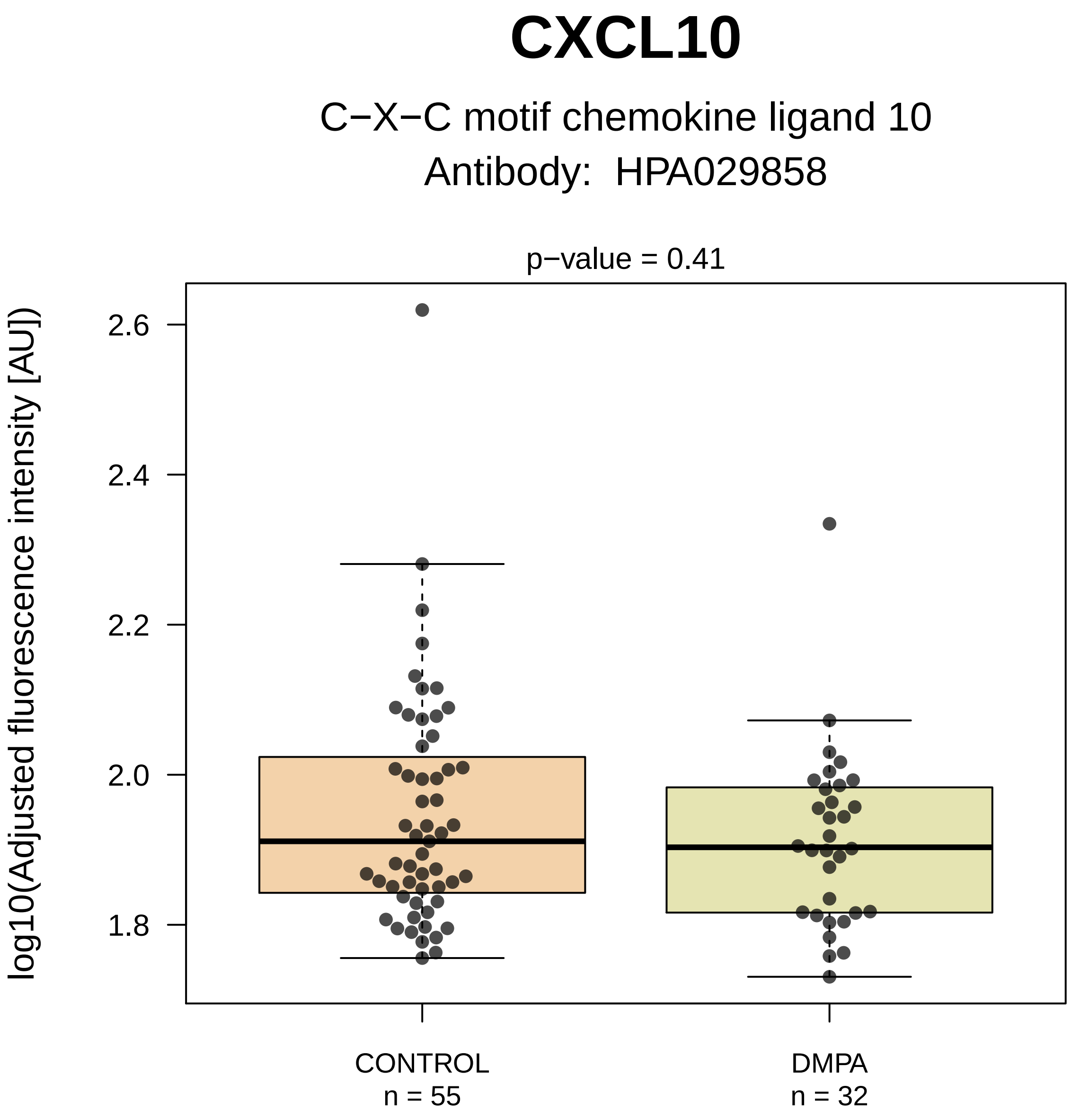
CXCL10
C−X−C motif chemokine ligand 10
Antibody: HPA029858
p−value = 0.41
log10(Adjusted fluorescence intensity [AU])
2.6
2.4
2.2
2.0
1.8
CONTROL
n = 55
DMPA
n = 32

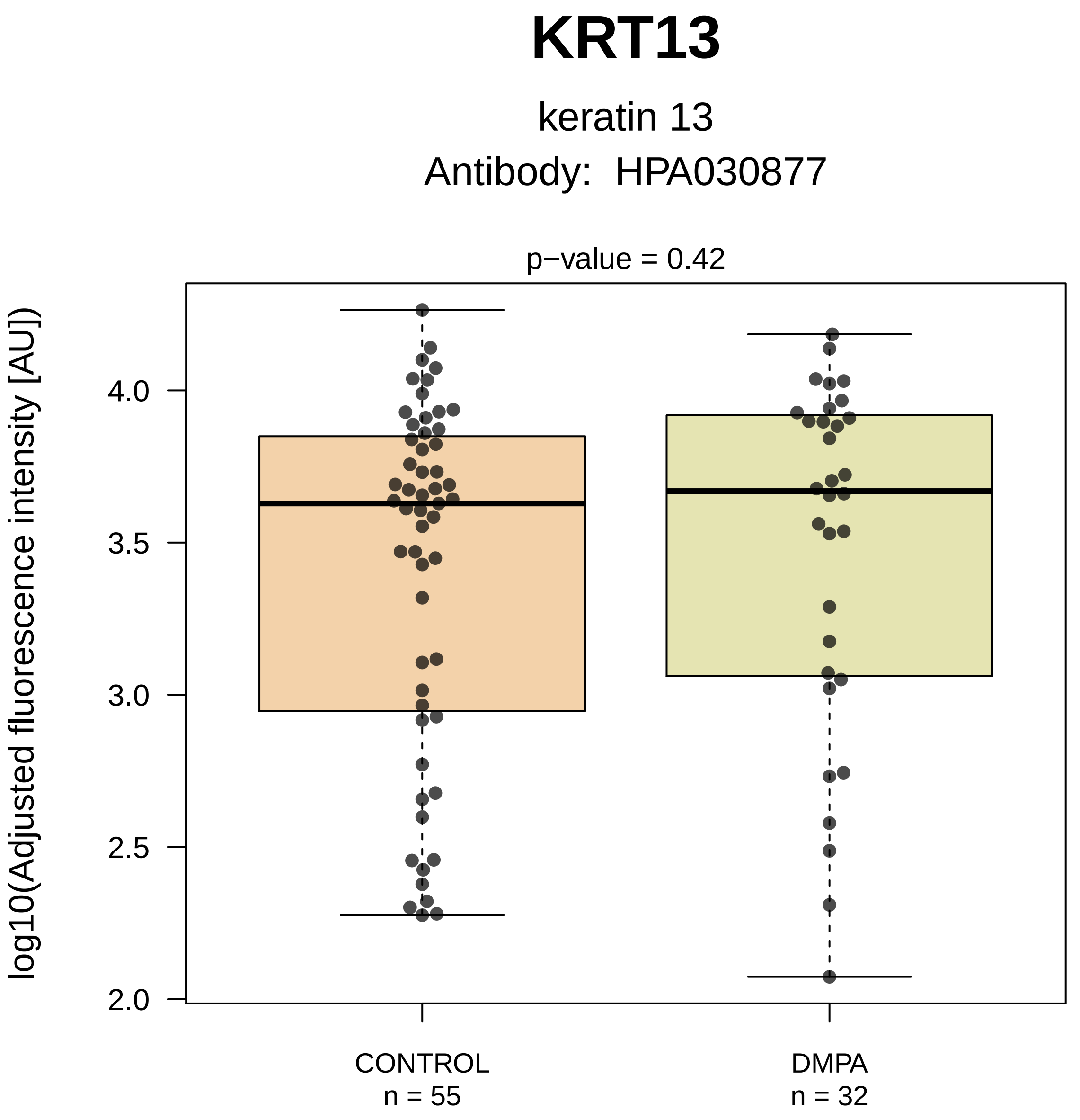

KRT13
keratin 13
Antibody: HPA030877
p−value = 0.42
log10(Adjusted fluorescence intensity [AU])
4.0
3.5
3.0
2.5
2.0
CONTROL
n = 55
DMPA
n = 32

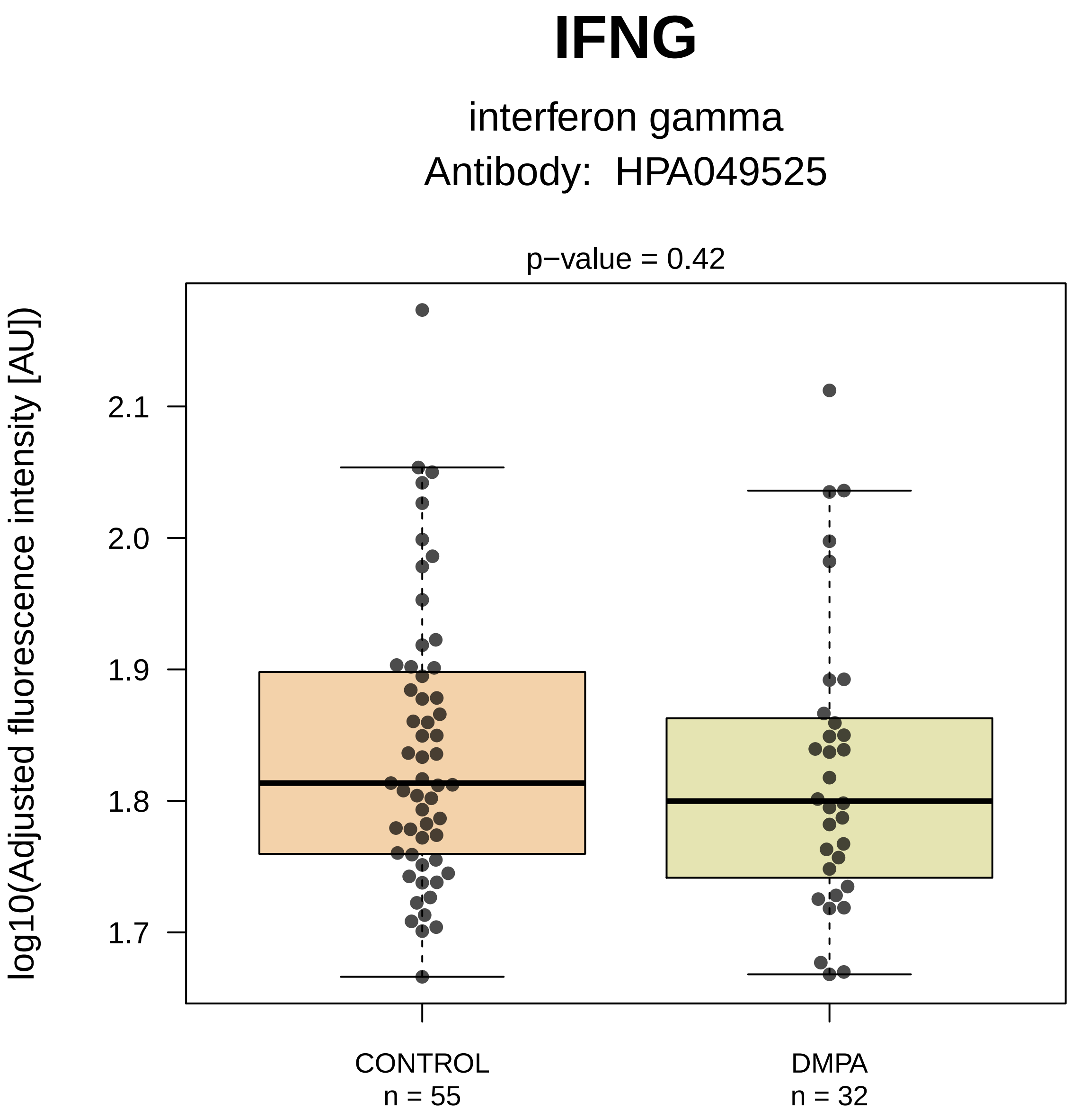
IFNG
interferon gamma
Antibody: HPA049525
p−value = 0.42
log10(Adjusted fluorescence intensity [AU])
2.1
2.0
1.9
1.8
1.7
CONTROL
n = 55
DMPA
n = 32

LRG1

leucine rich alpha−2−glycoprotein 1
Antibody: HPA001888

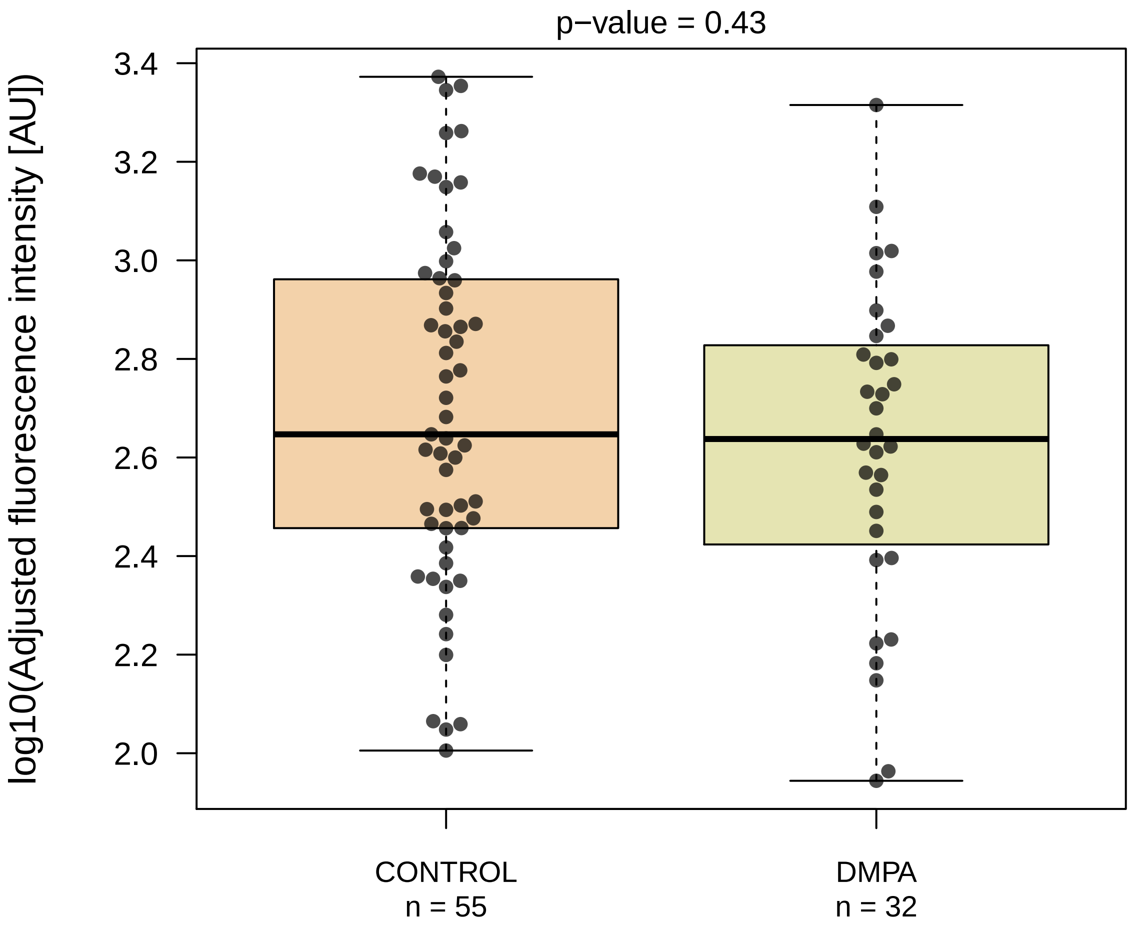

C4BPA

complement component 4 binding protein alpha
Antibody: HPA001845

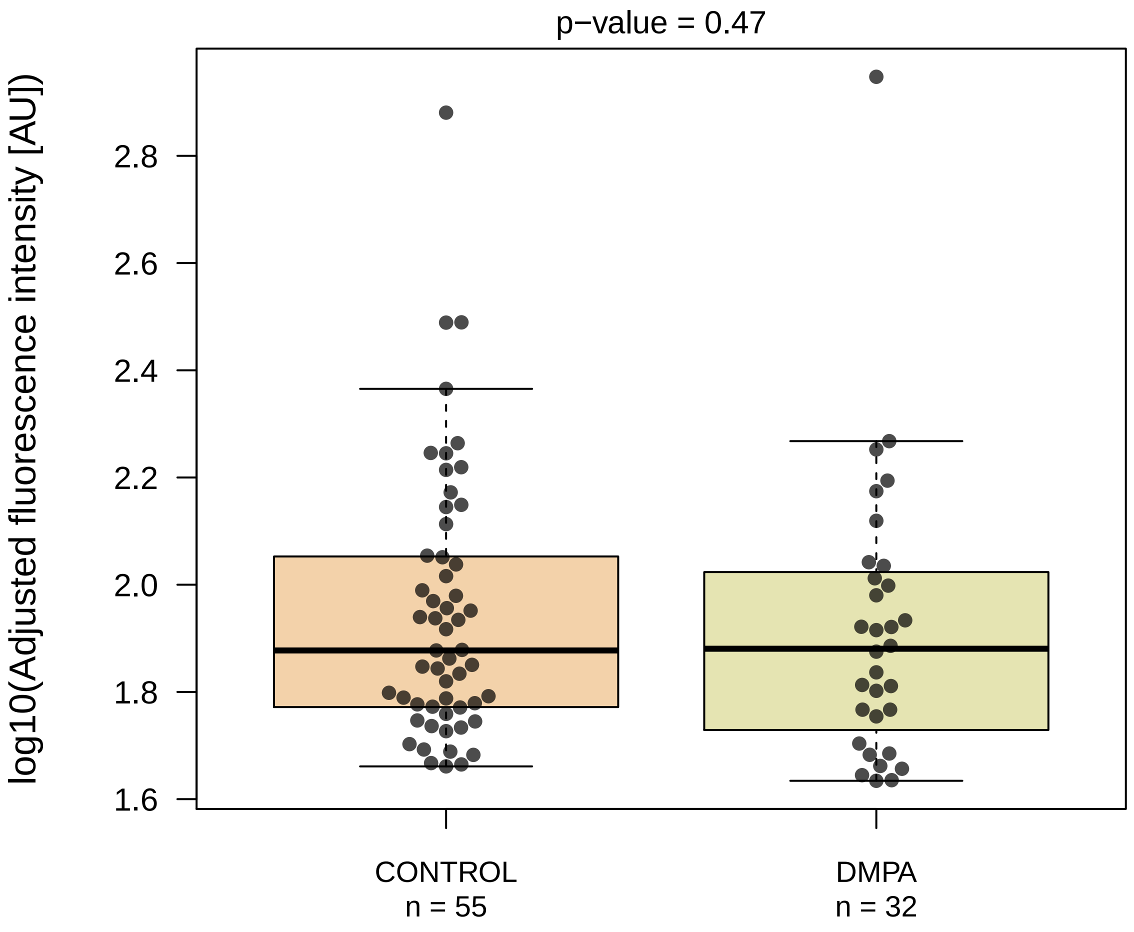

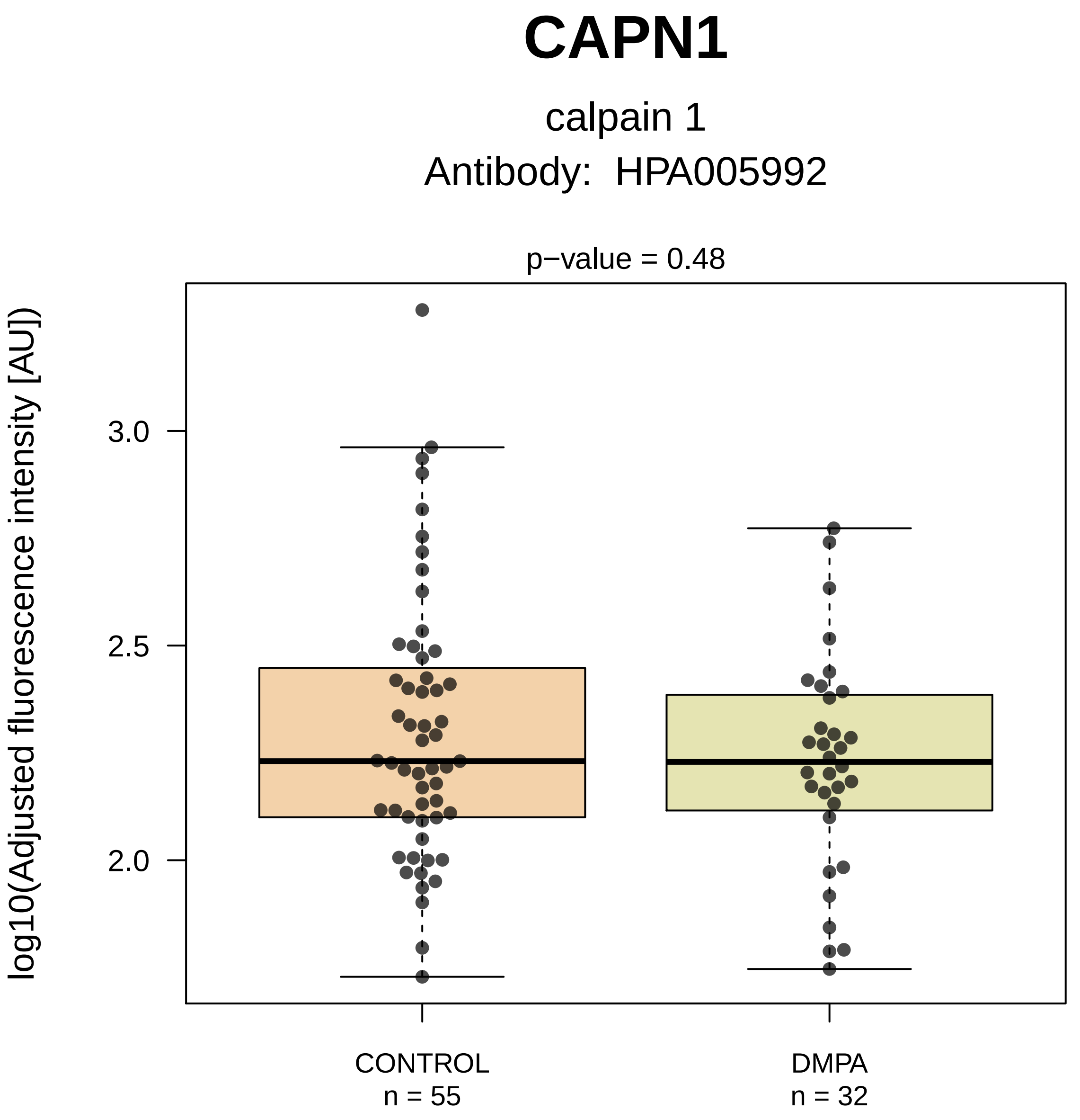
CAPN1
calpain 1
Antibody: HPA005992
p−value = 0.48
log10(Adjusted fluorescence intensity [AU])
3.0
2.5
2.0
CONTROL
n = 55
DMPA
n = 32

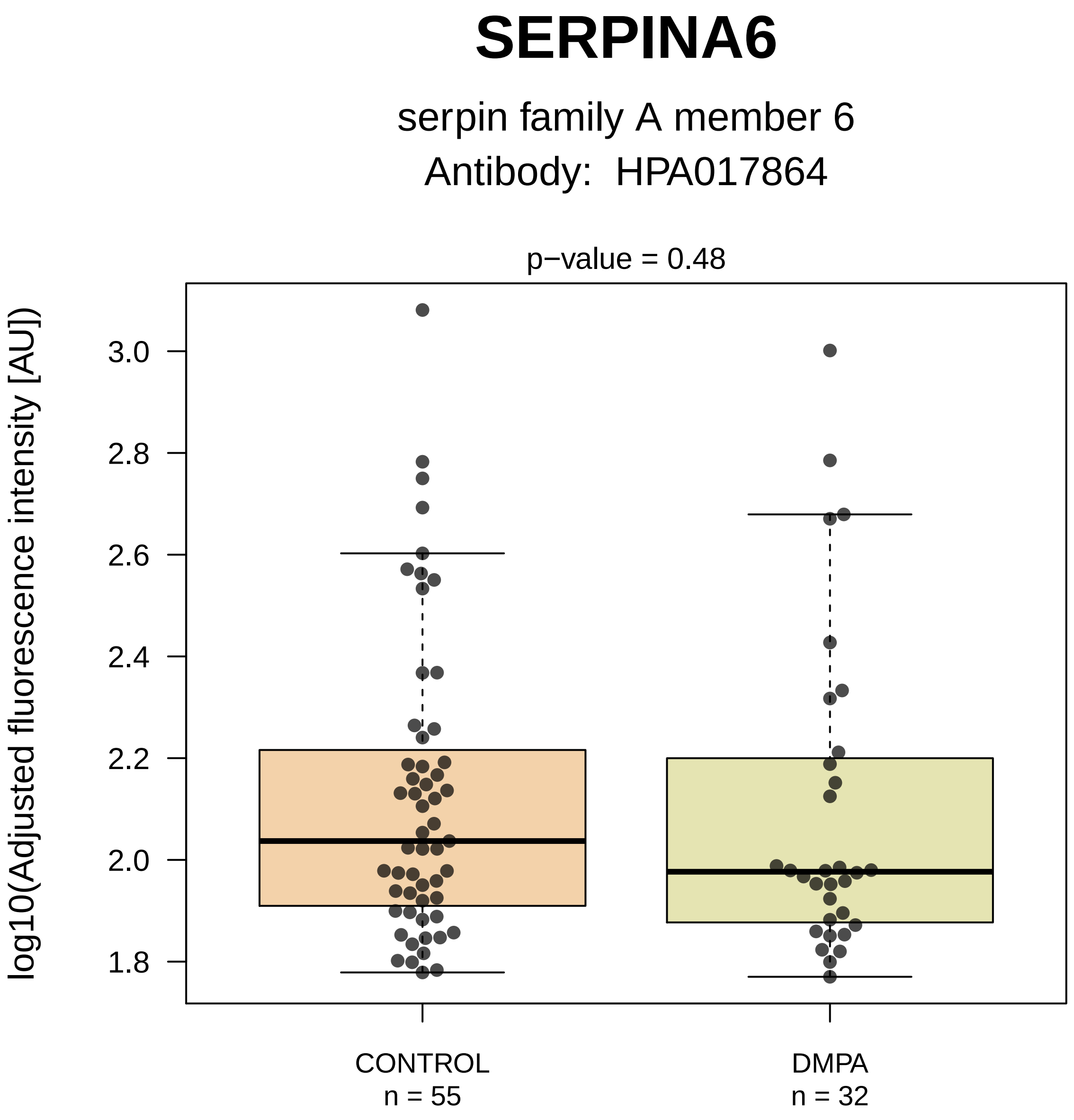
SERPINA6
serpin family A member 6
Antibody: HPA017864
p−value = 0.48
log10(Adjusted fluorescence intensity [AU])
3.0
2.8
2.6
2.4
2.2
2.0
1.8
CONTROL
n = 55
DMPA
n = 32

F11R

F11 receptor
Antibody: HPA061700

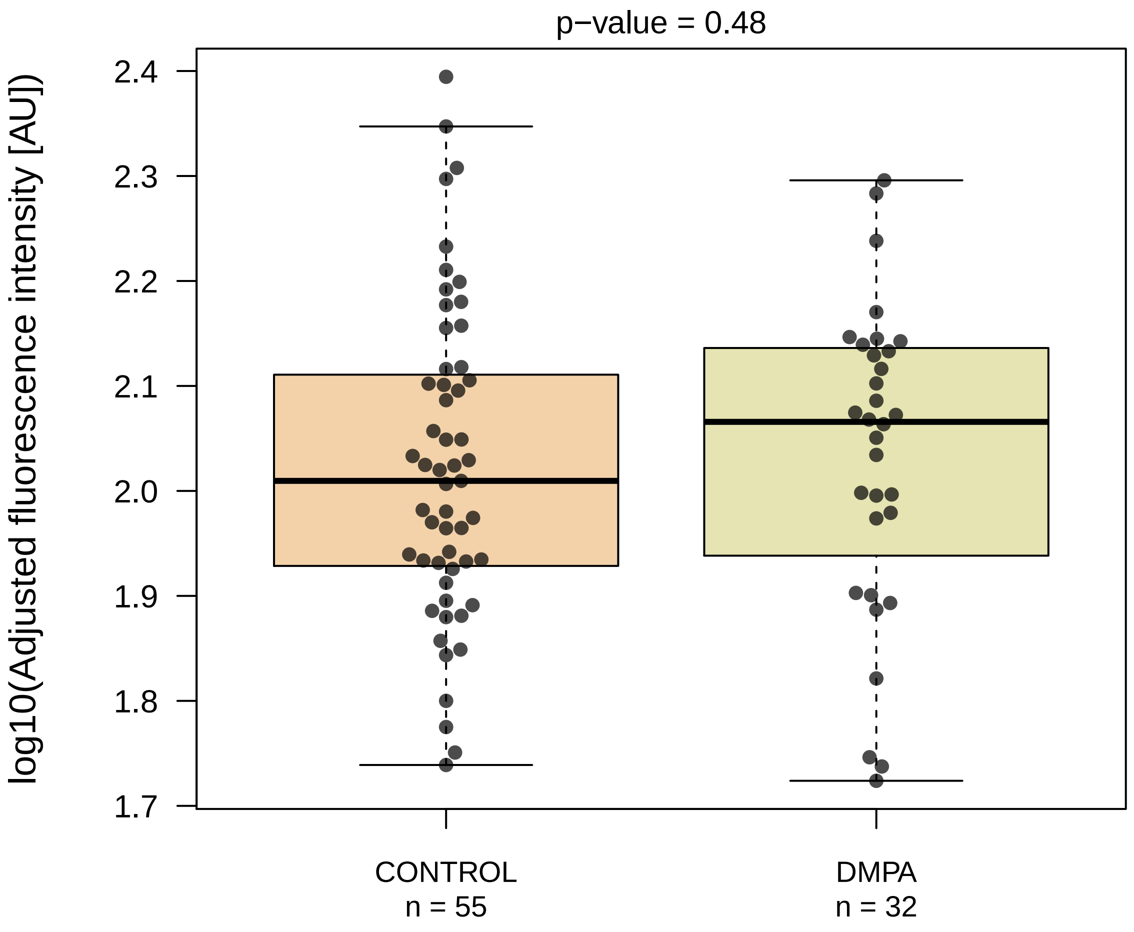

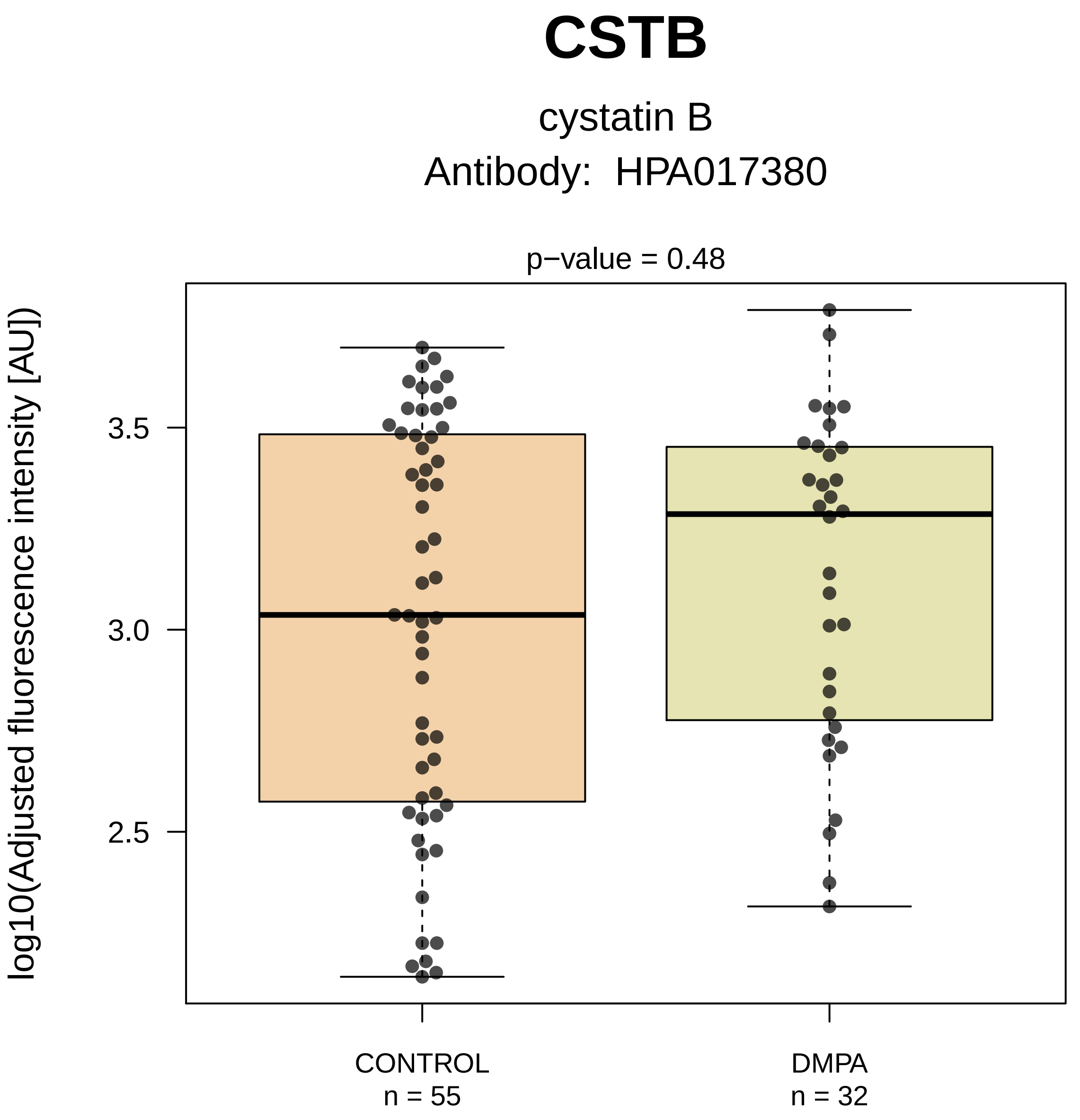
CSTB
cystatin B
Antibody: HPA017380
p−value = 0.48
log10(Adjusted fluorescence intensity [AU])
3.5
3.0
2.5
CONTROL
n = 55
DMPA
n = 32

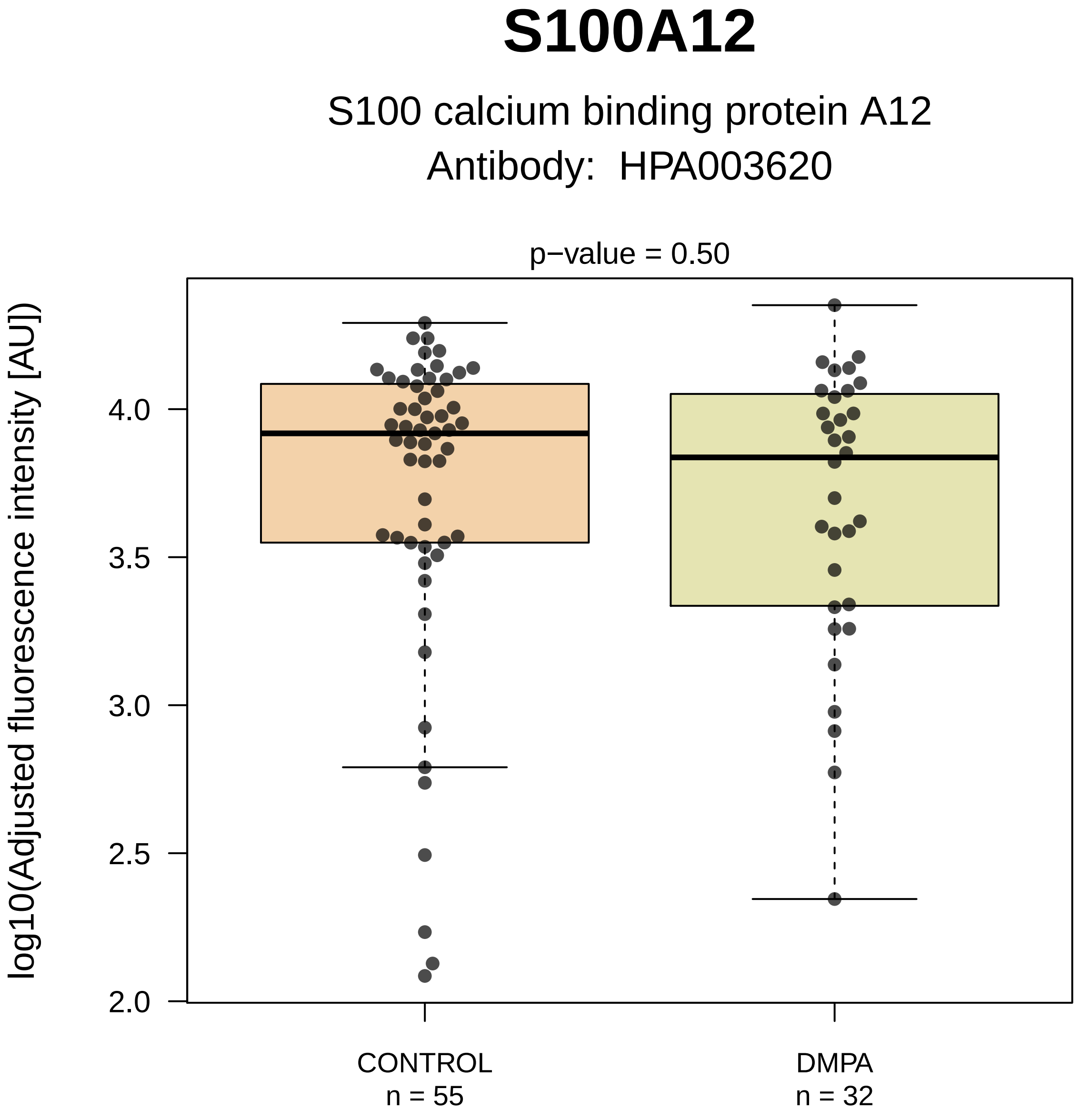
S100A12
S100 calcium binding protein A12
Antibody: HPA003620
p−value = 0.50
log10(Adjusted fluorescence intensity [AU])
4.0
3.5
3.0
2.5
2.0
CONTROL
n = 55
DMPA
n = 32

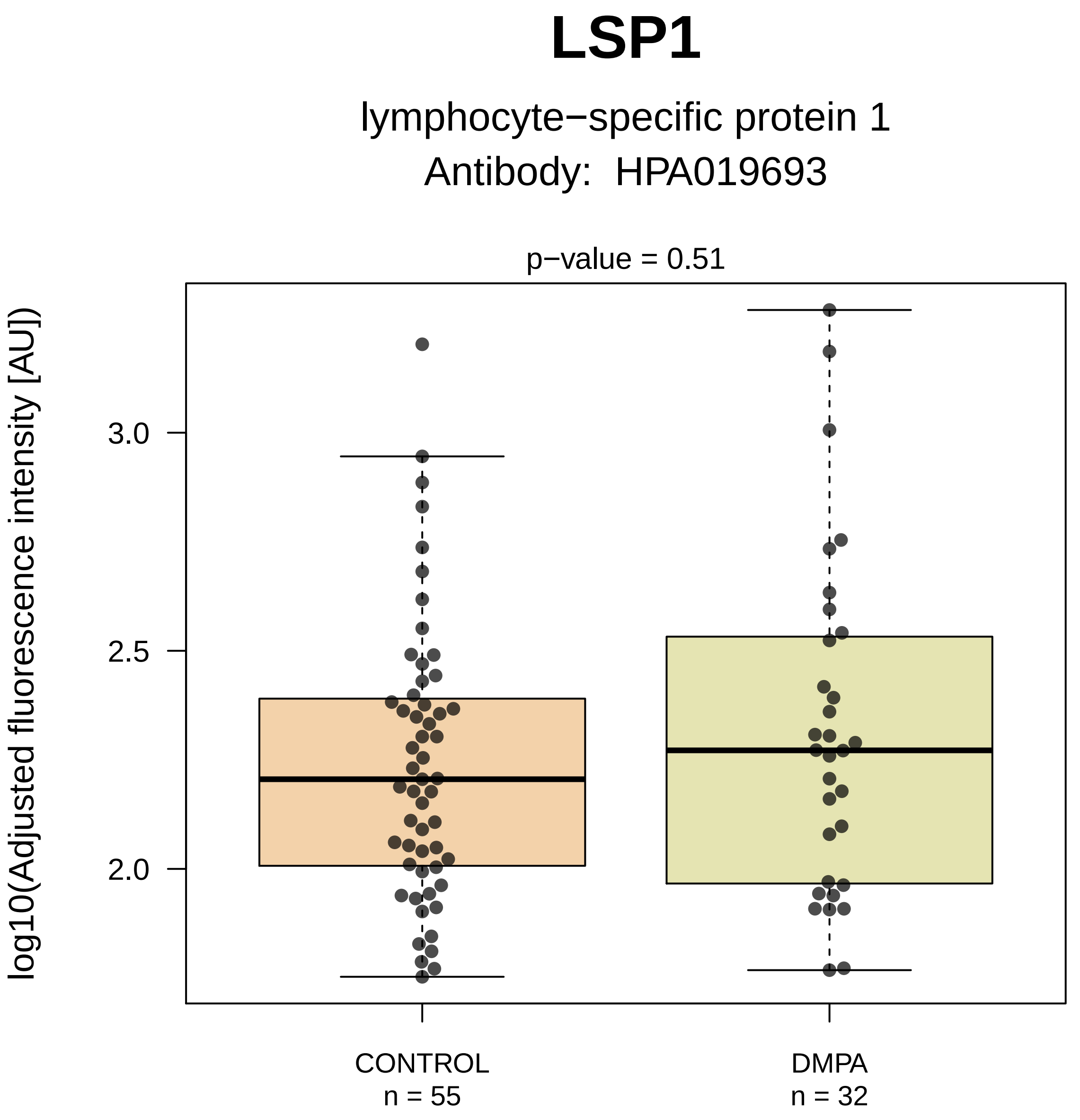
LSP1
lymphocyte−specific protein 1
Antibody: HPA019693
p−value = 0.51
log10(Adjusted fluorescence intensity [AU])
3.0
2.5
2.0
CONTROL
n = 55
DMPA
n = 32

MMP8

matrix metallopeptidase 8
Antibody: HPA022935

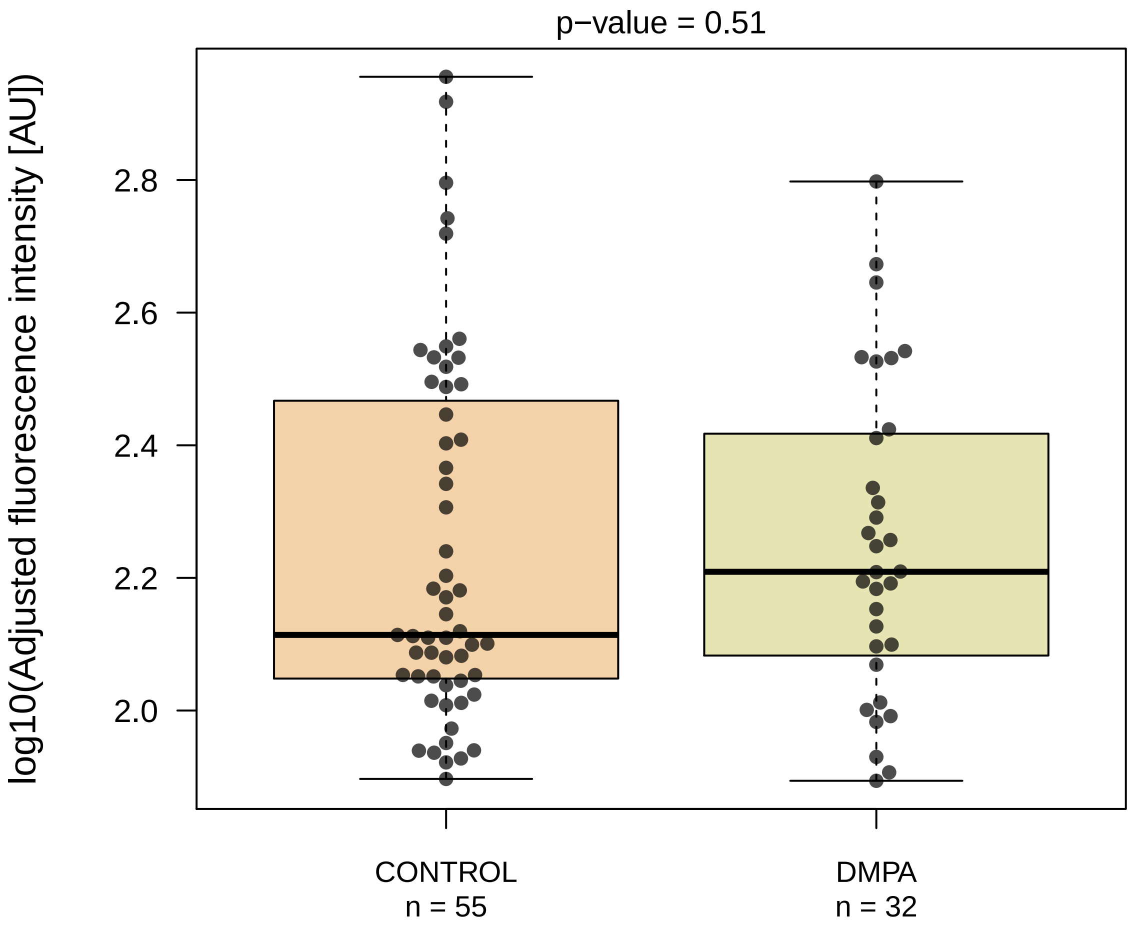

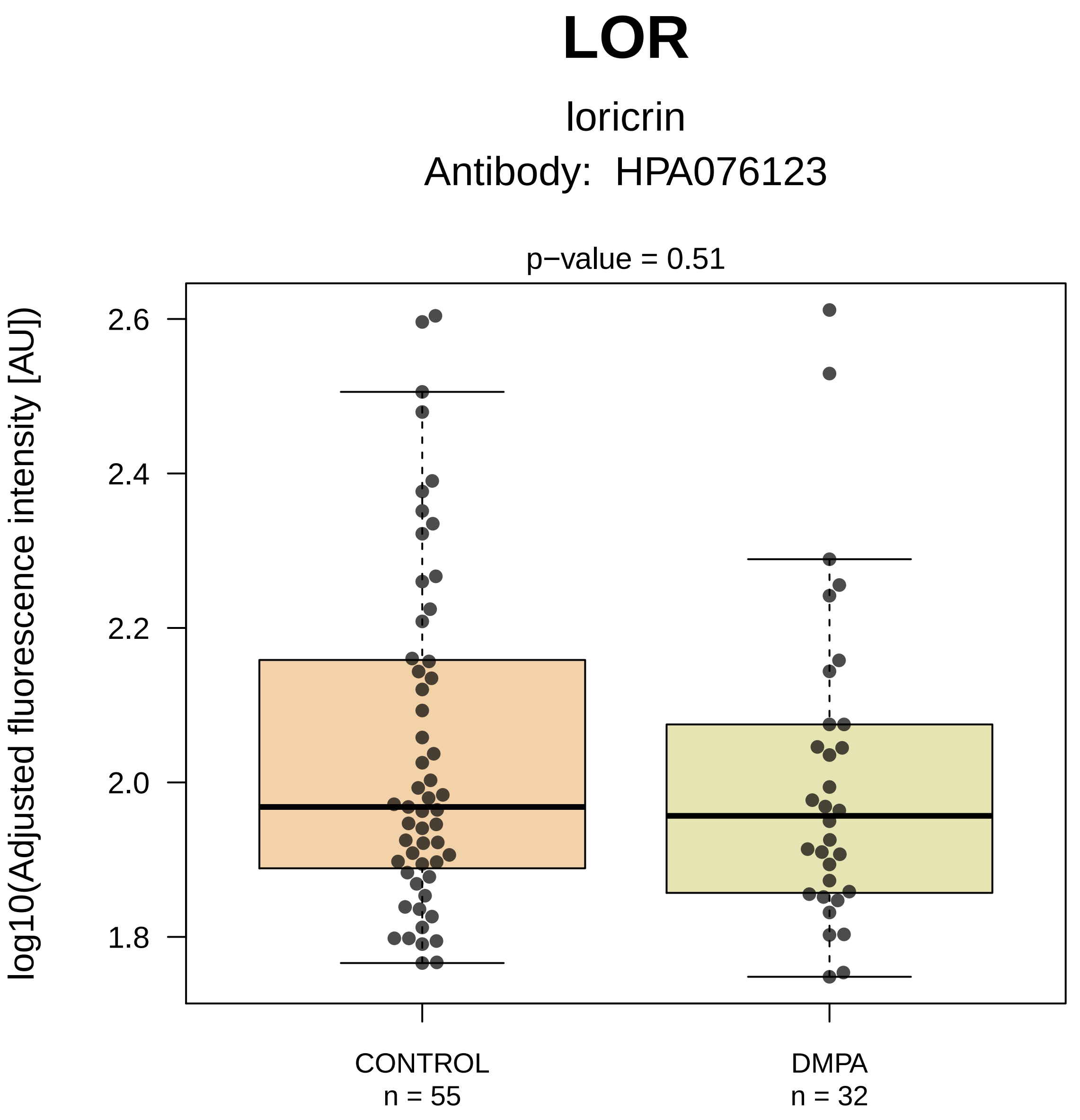

LOR
loricrin
Antibody: HPA076123
p−value = 0.51
log10(Adjusted fluorescence intensity [AU])
2.6
2.4
2.2
2.0
1.8
CONTROL
n = 55
DMPA
n = 32

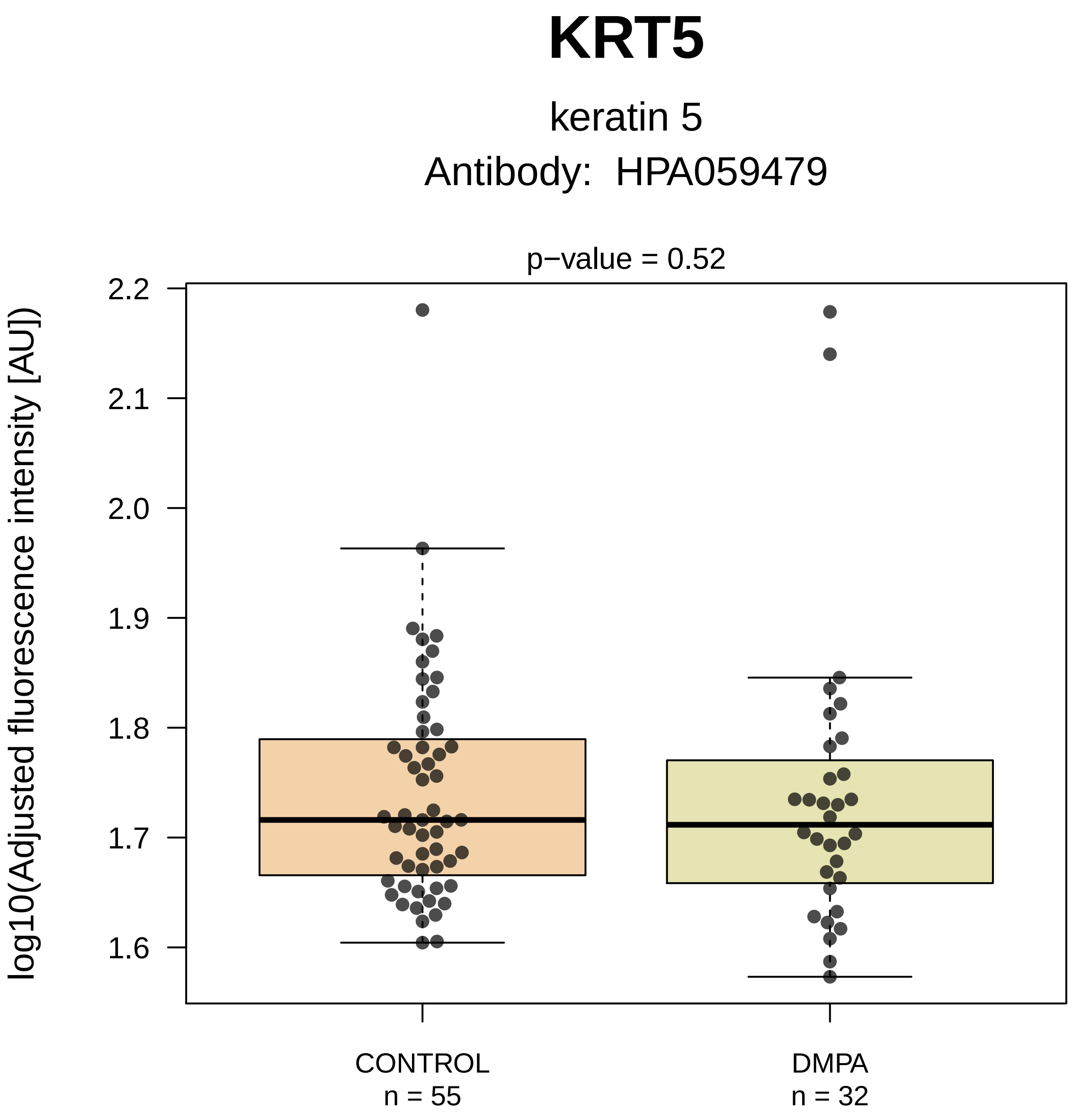
KRT5
keratin 5
Antibody: HPA059479
p−value = 0.52
log10(Adjusted fluorescence intensity [AU])
2.2
2.1
2.0
1.9
1.8
1.7
1.6
CONTROL
n = 55
DMPA
n = 32

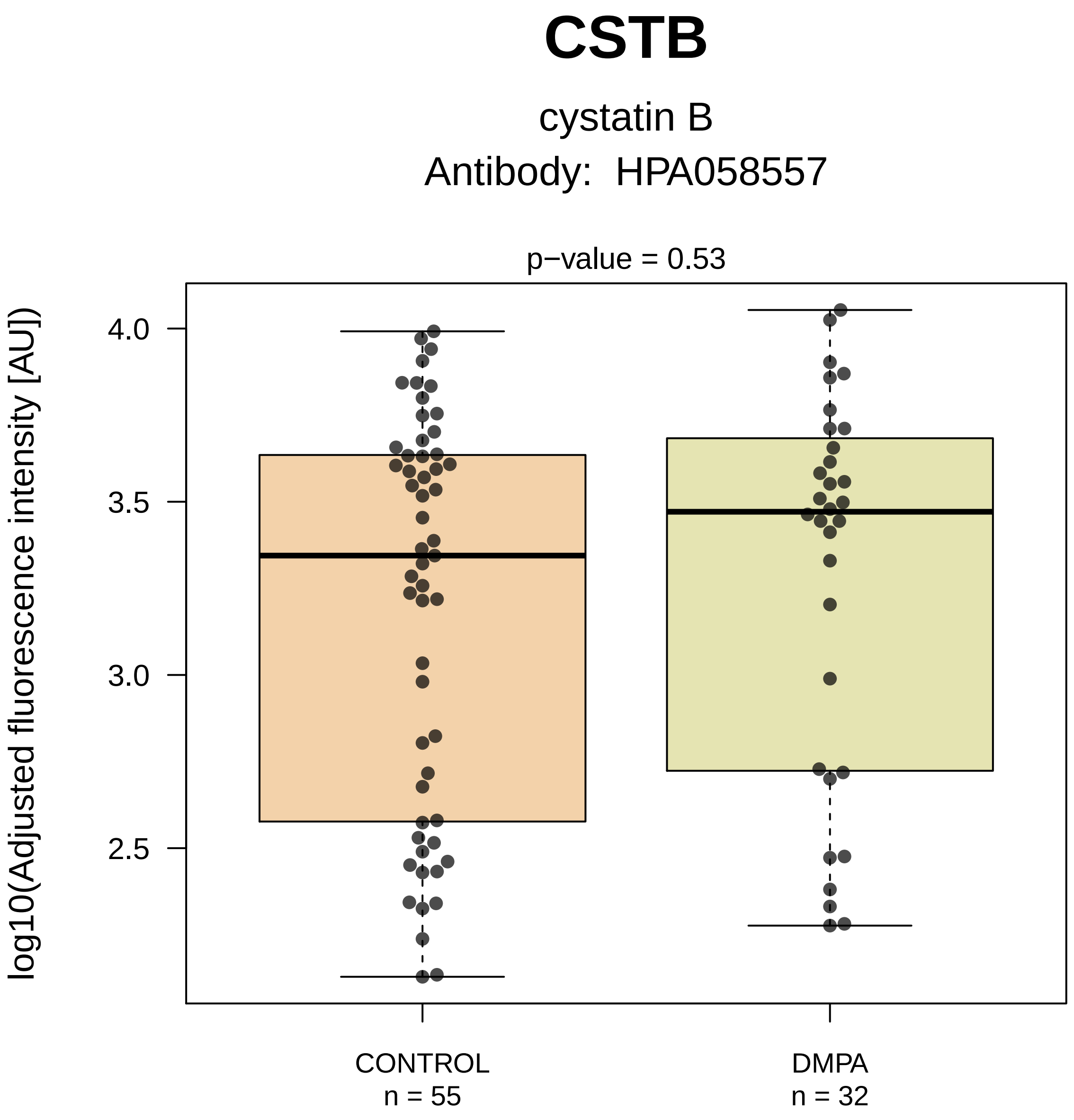
CSTB
cystatin B
Antibody: HPA058557
p−value = 0.53
log10(Adjusted fluorescence intensity [AU])
4.0
3.5
3.0
2.5
CONTROL
n = 55
DMPA
n = 32

CD5L

CD5 molecule like
Antibody: HPA026432

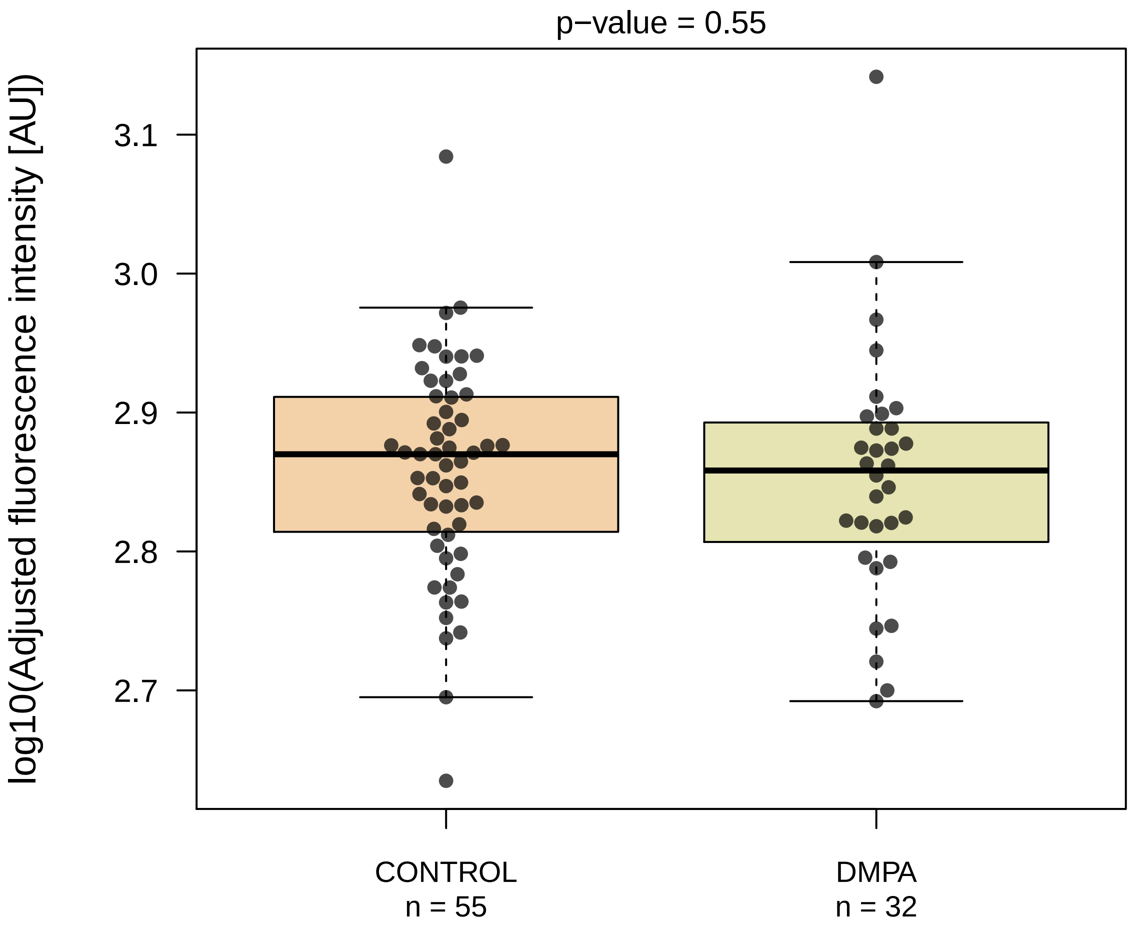

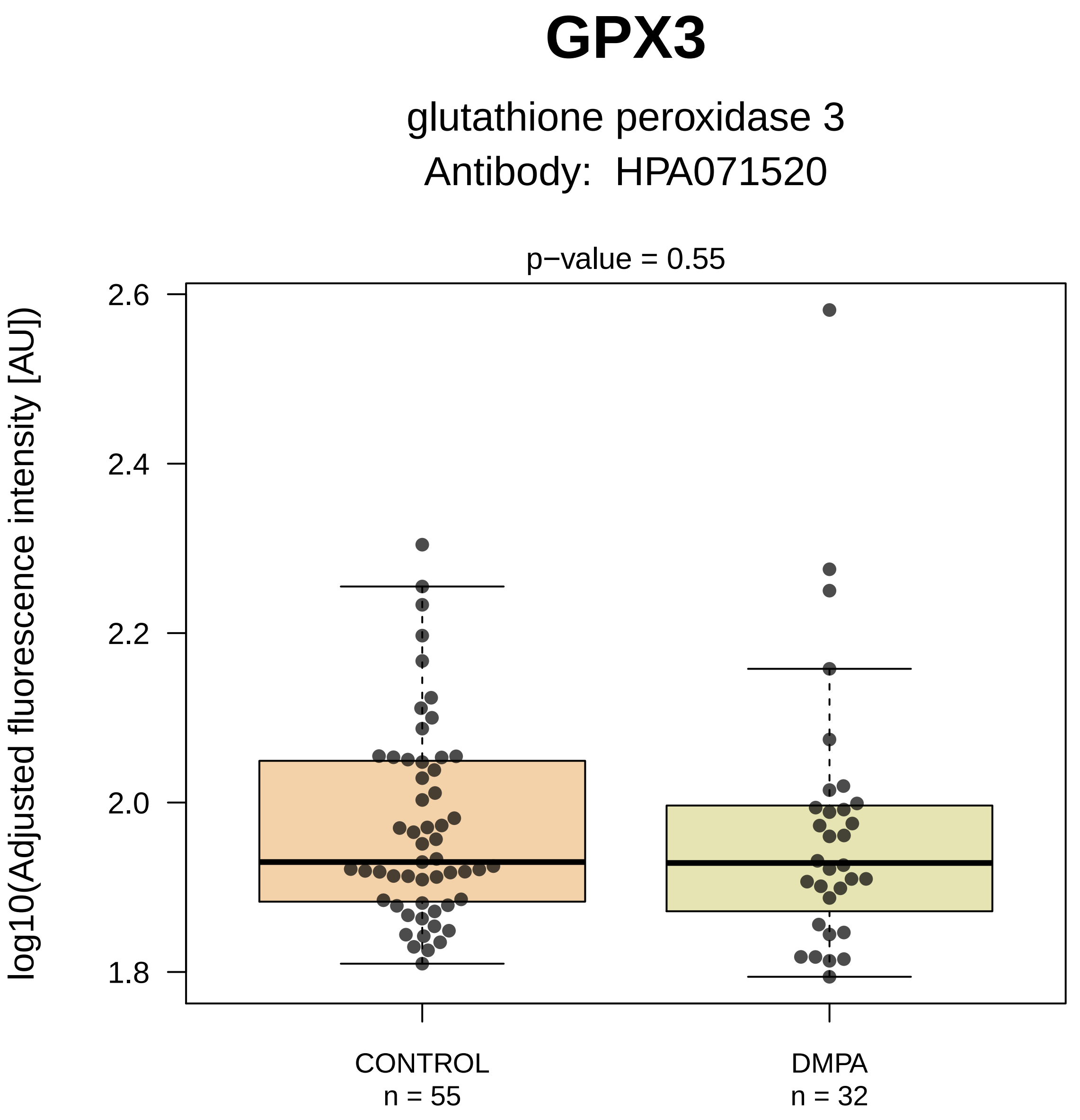
GPX3
glutathione peroxidase 3
Antibody: HPA071520
p−value = 0.55
log10(Adjusted fluorescence intensity [AU])
2.6
2.4
2.2
2.0
1.8
CONTROL
n = 55
DMPA
n = 32

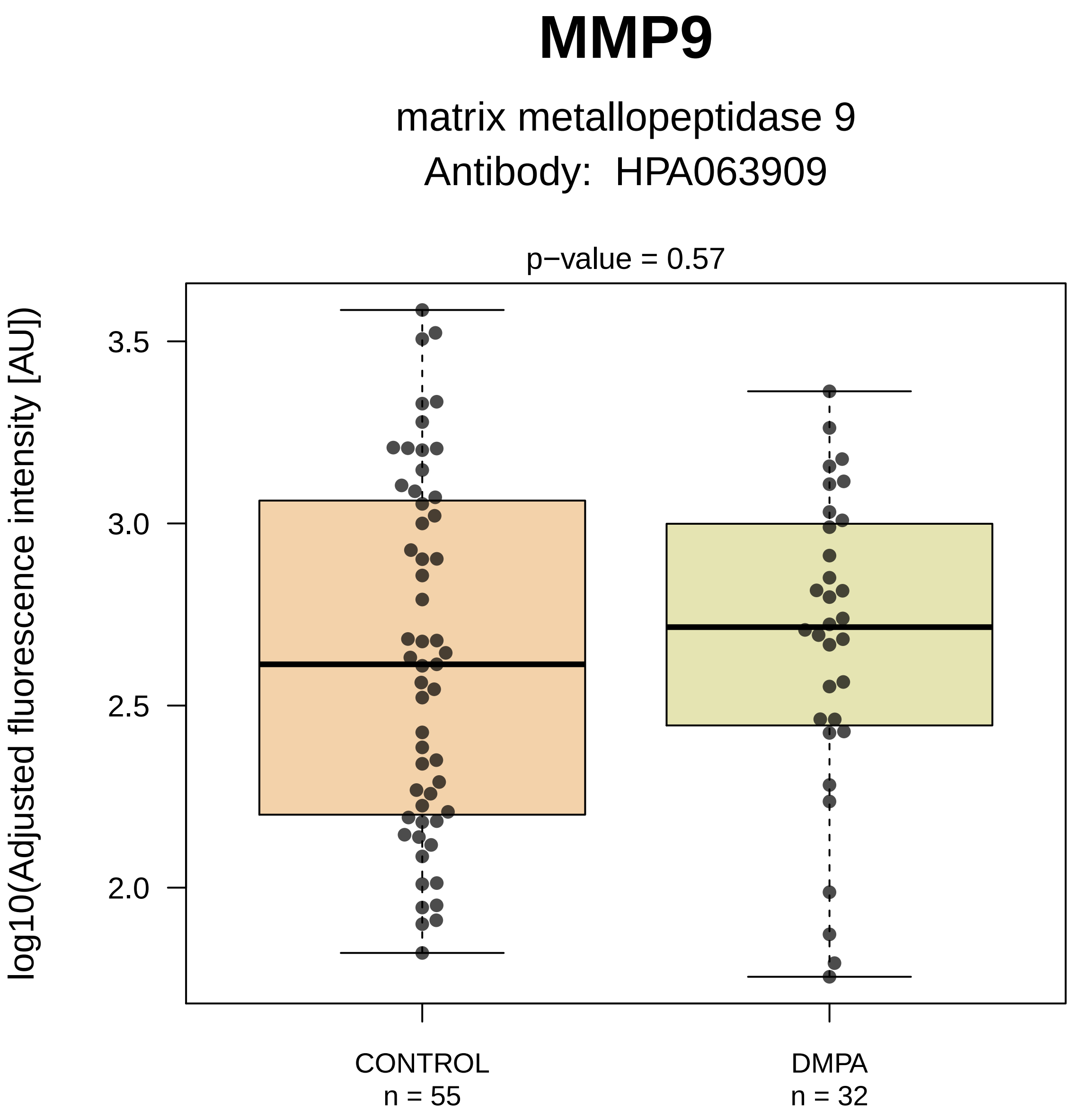
MMP9
matrix metallopeptidase 9
Antibody: HPA063909
p−value = 0.57
log10(Adjusted fluorescence intensity [AU])
3.5
3.0
2.5
2.0
CONTROL
n = 55
DMPA
n = 32

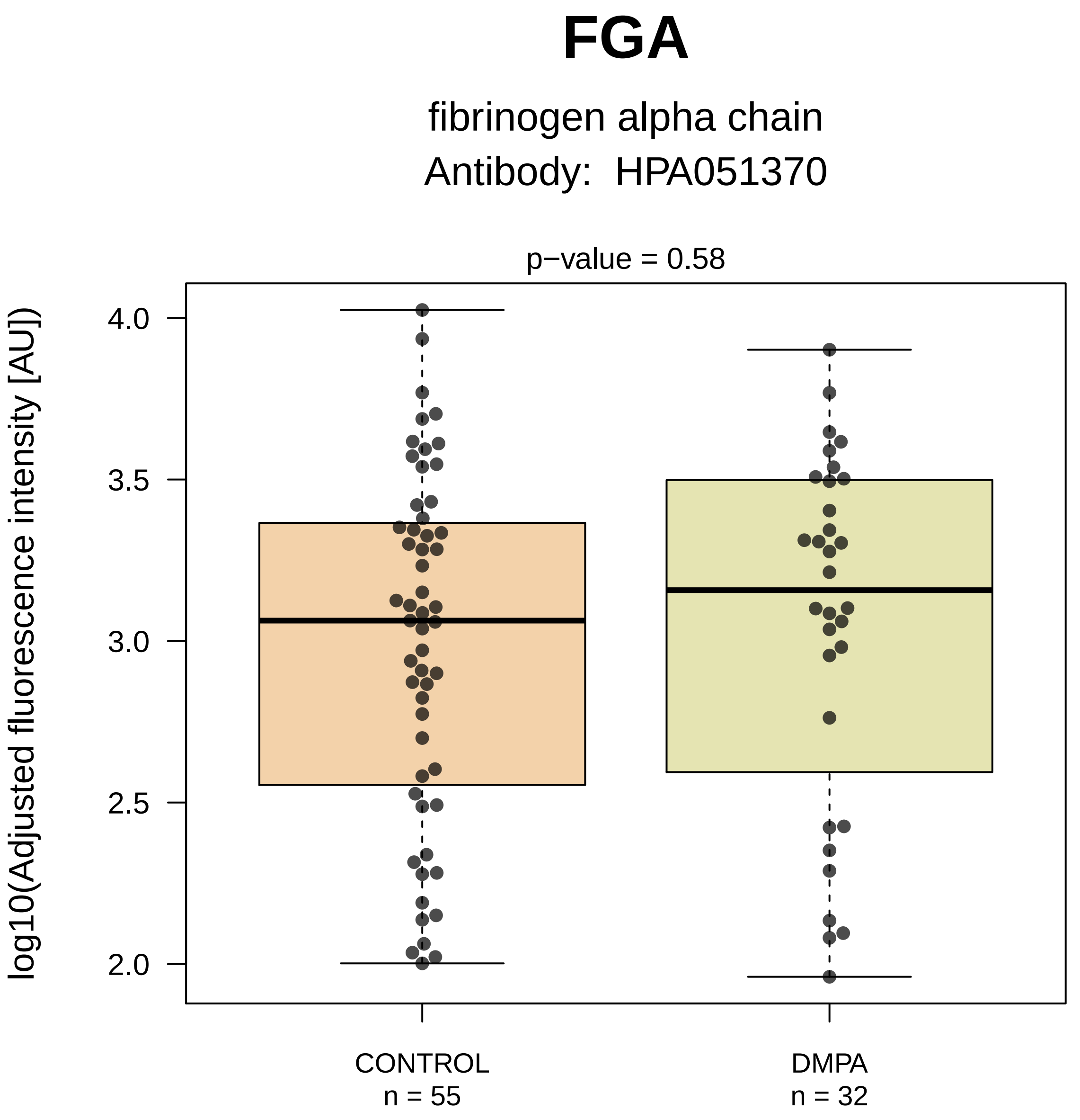
FGA
fibrinogen alpha chain
Antibody: HPA051370
p−value = 0.58
log10(Adjusted fluorescence intensity [AU])
4.0
3.5
3.0
2.5
2.0
CONTROL
n = 55
DMPA
n = 32

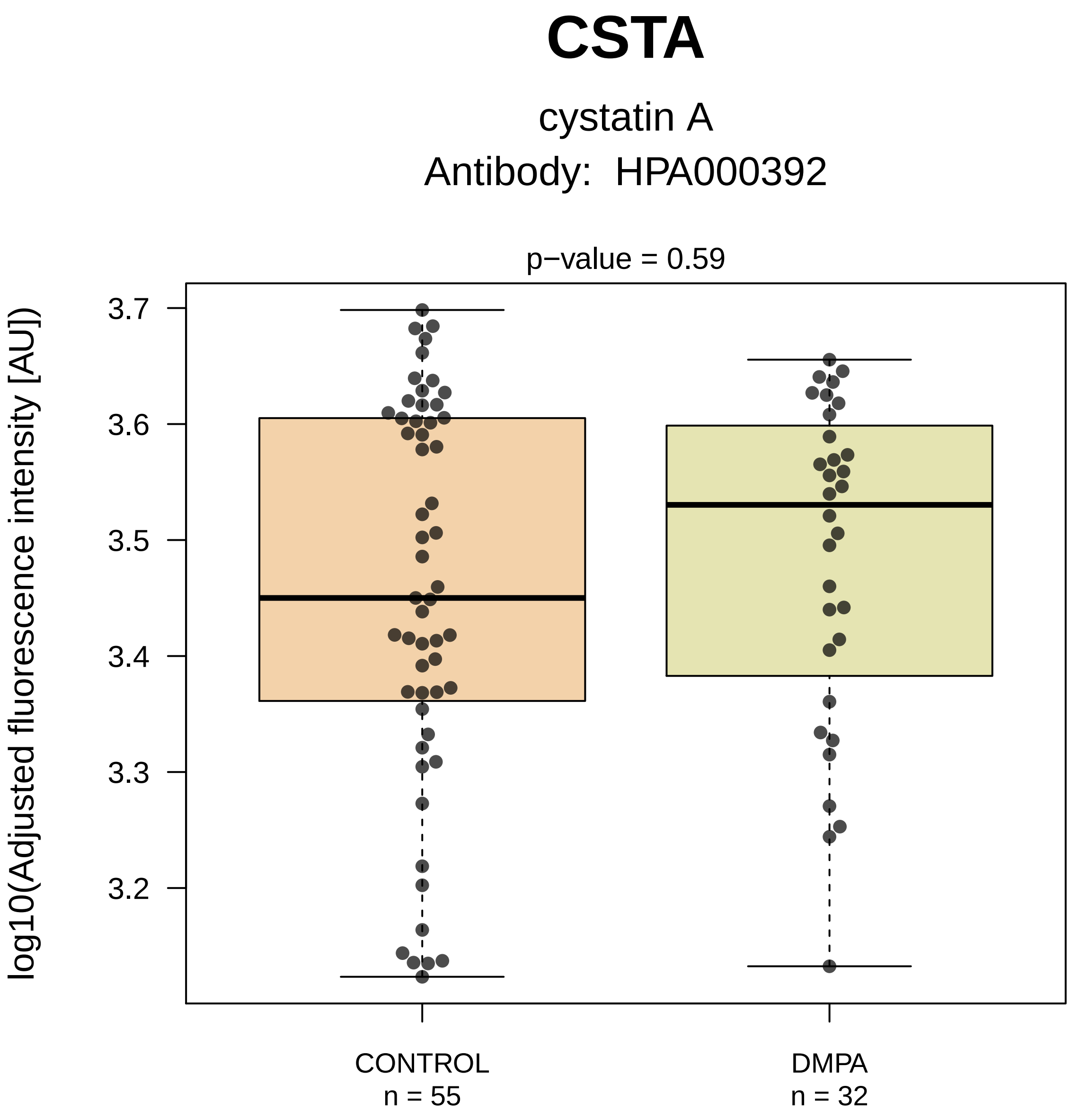
CSTA
cystatin A
Antibody: HPA000392
p−value = 0.59
log10(Adjusted fluorescence intensity [AU])
3.7
3.6
3.5
3.4
3.3
3.2
CONTROL
n = 55
DMPA
n = 32

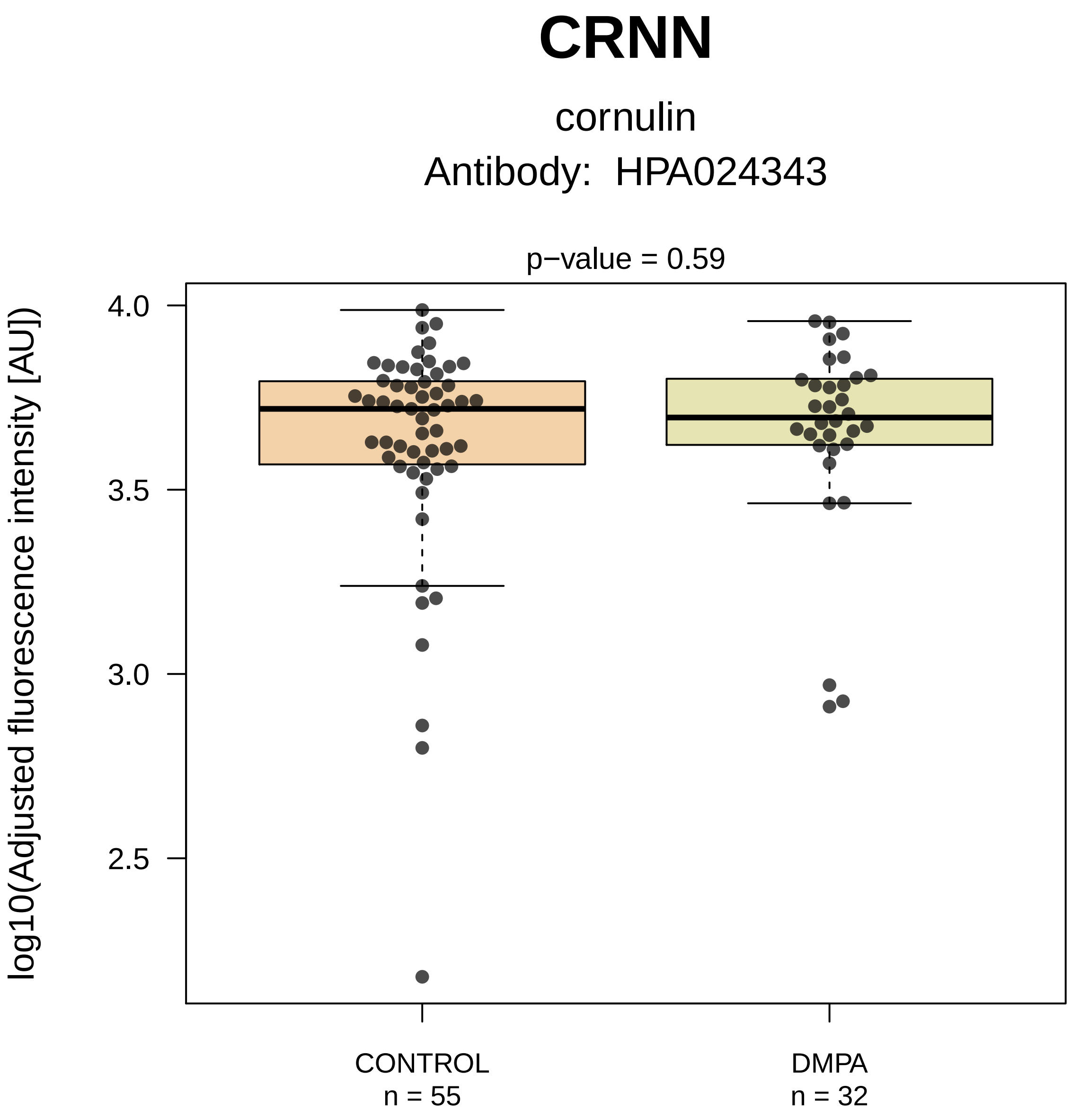
CRNN
cornulin
Antibody: HPA024343
p−value = 0.59
log10(Adjusted fluorescence intensity [AU])
4.0
3.5
3.0
2.5
CONTROL
n = 55
DMPA
n = 32

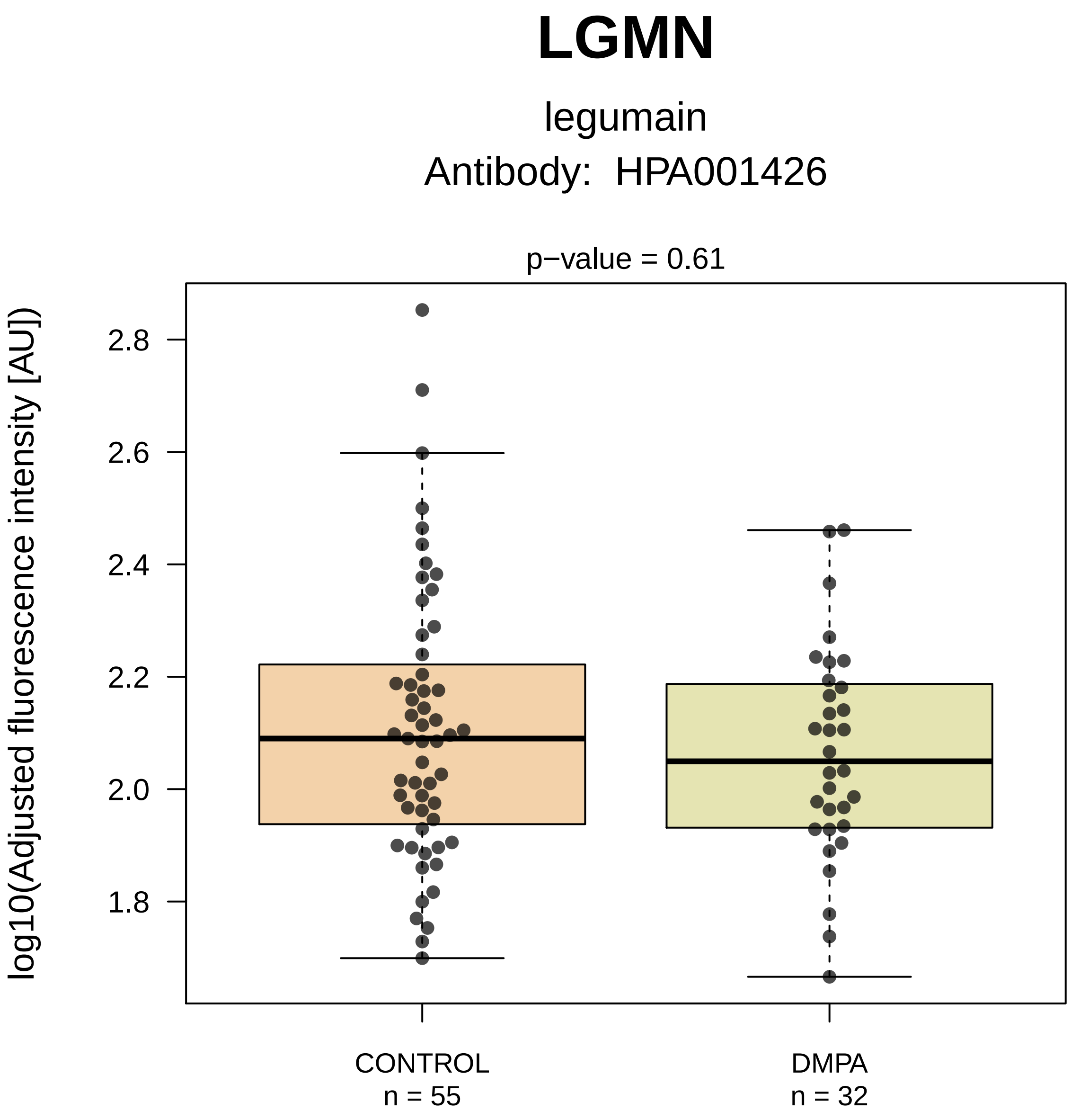
LGMN
legumain
Antibody: HPA001426
p−value = 0.61
log10(Adjusted fluorescence intensity [AU])
2.8
2.6
2.4
2.2
2.0
1.8
CONTROL
n = 55
DMPA
n = 32

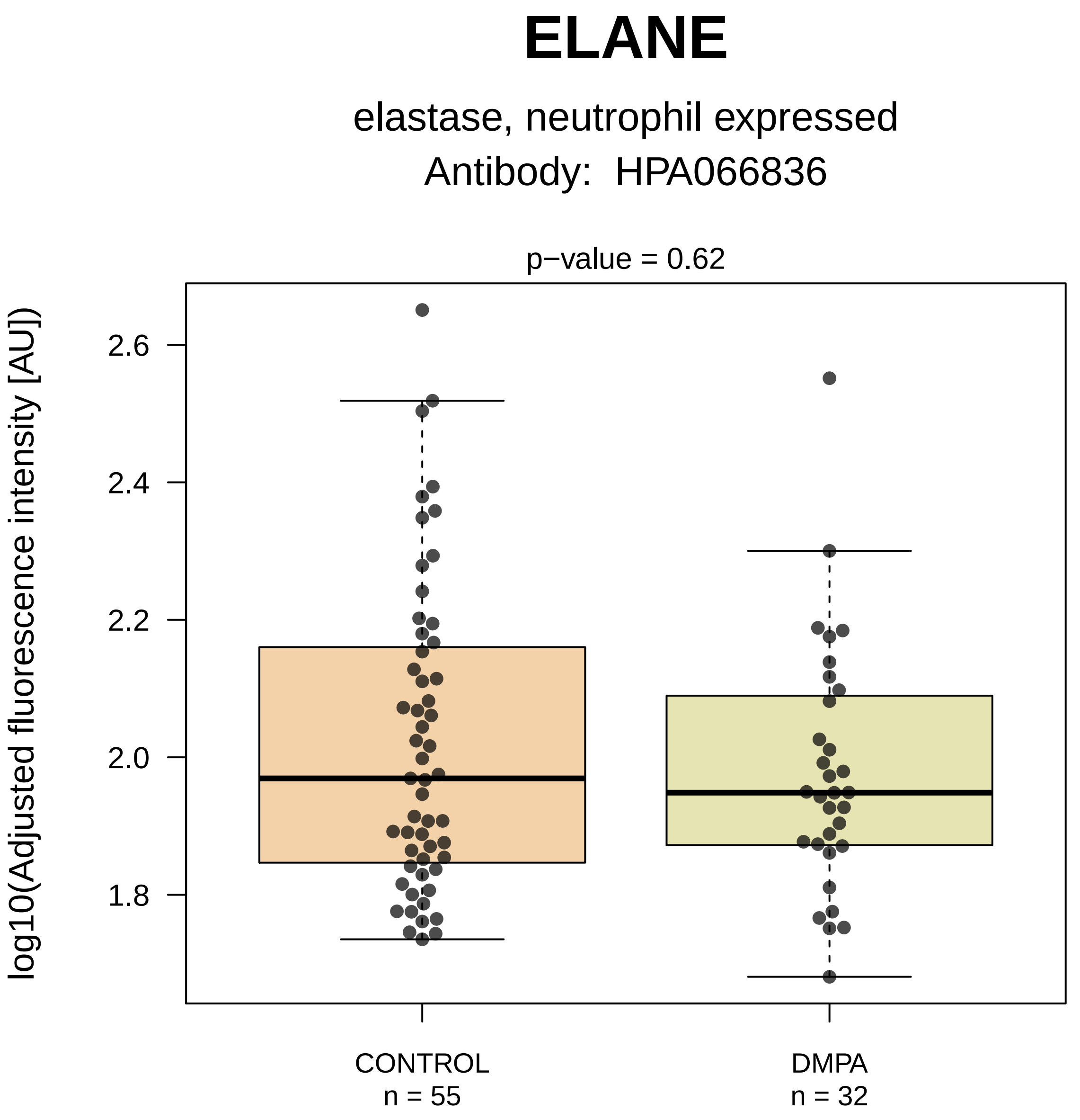
ELANE
elastase, neutrophil expressed
Antibody: HPA066836
p−value = 0.62
log10(Adjusted fluorescence intensity [AU])
2.6
2.4
2.2
2.0
1.8
CONTROL
n = 55
DMPA
n = 32

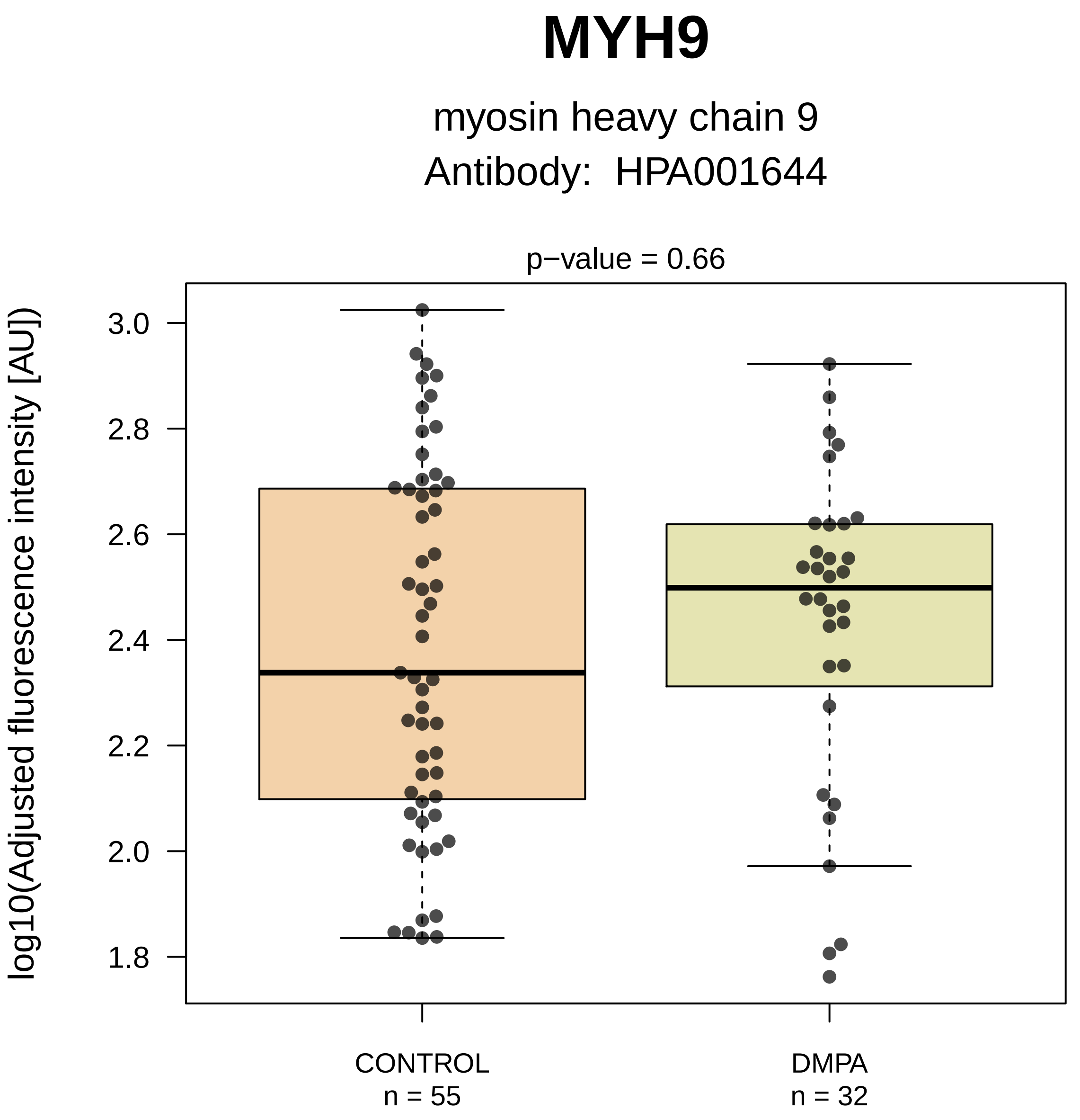
MYH9
myosin heavy chain 9
Antibody: HPA001644
p−value = 0.66
log10(Adjusted fluorescence intensity [AU])
3.0
2.8
2.6
2.4
2.2
2.0
1.8
CONTROL
n = 55
DMPA
n = 32

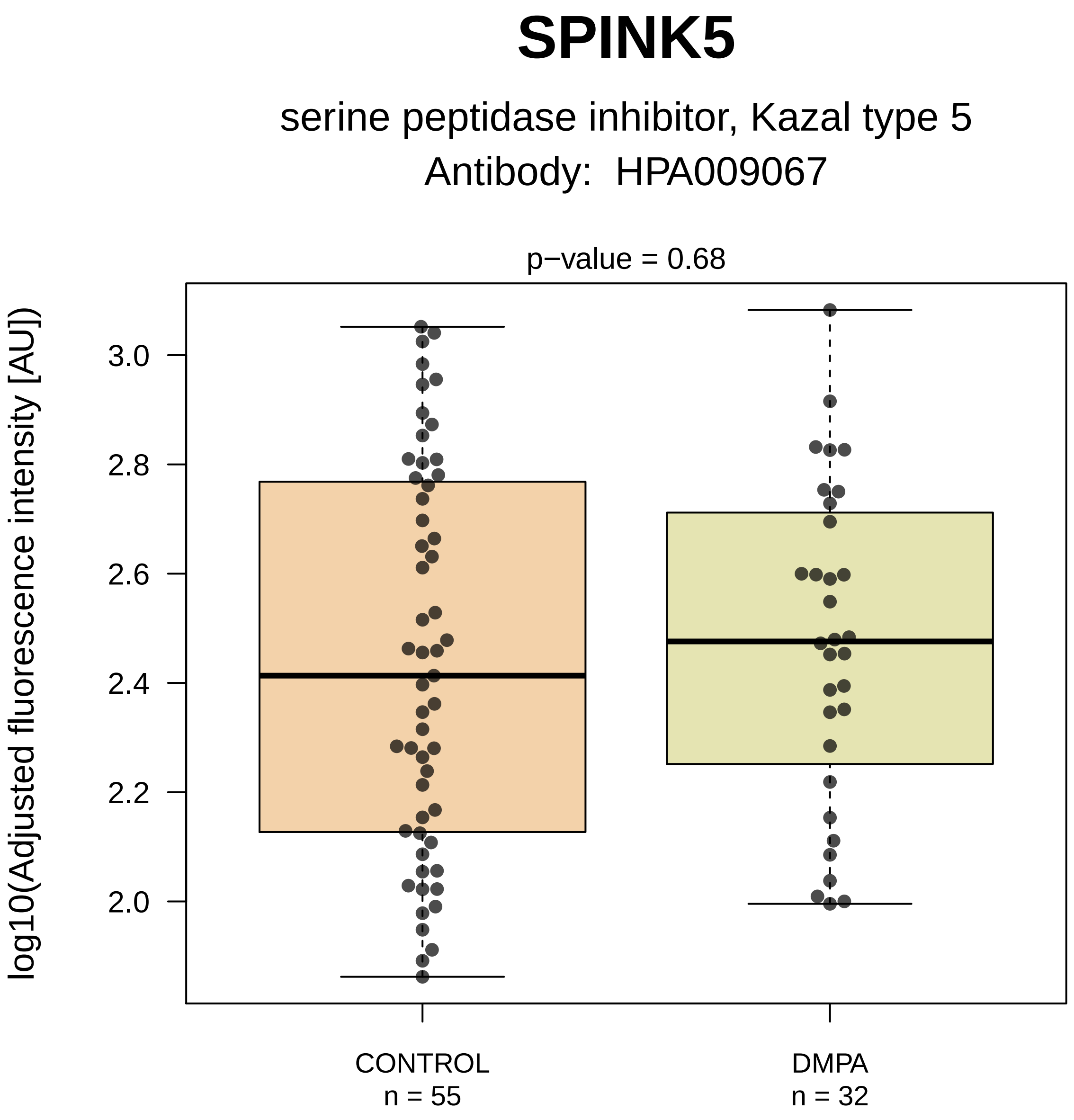

SPINK5
serine peptidase inhibitor, Kazal type 5
Antibody: HPA009067
p−value = 0.68
log10(Adjusted fluorescence intensity [AU])
3.0
2.8
2.6
2.4
2.2
2.0
CONTROL
n = 55
DMPA
n = 32

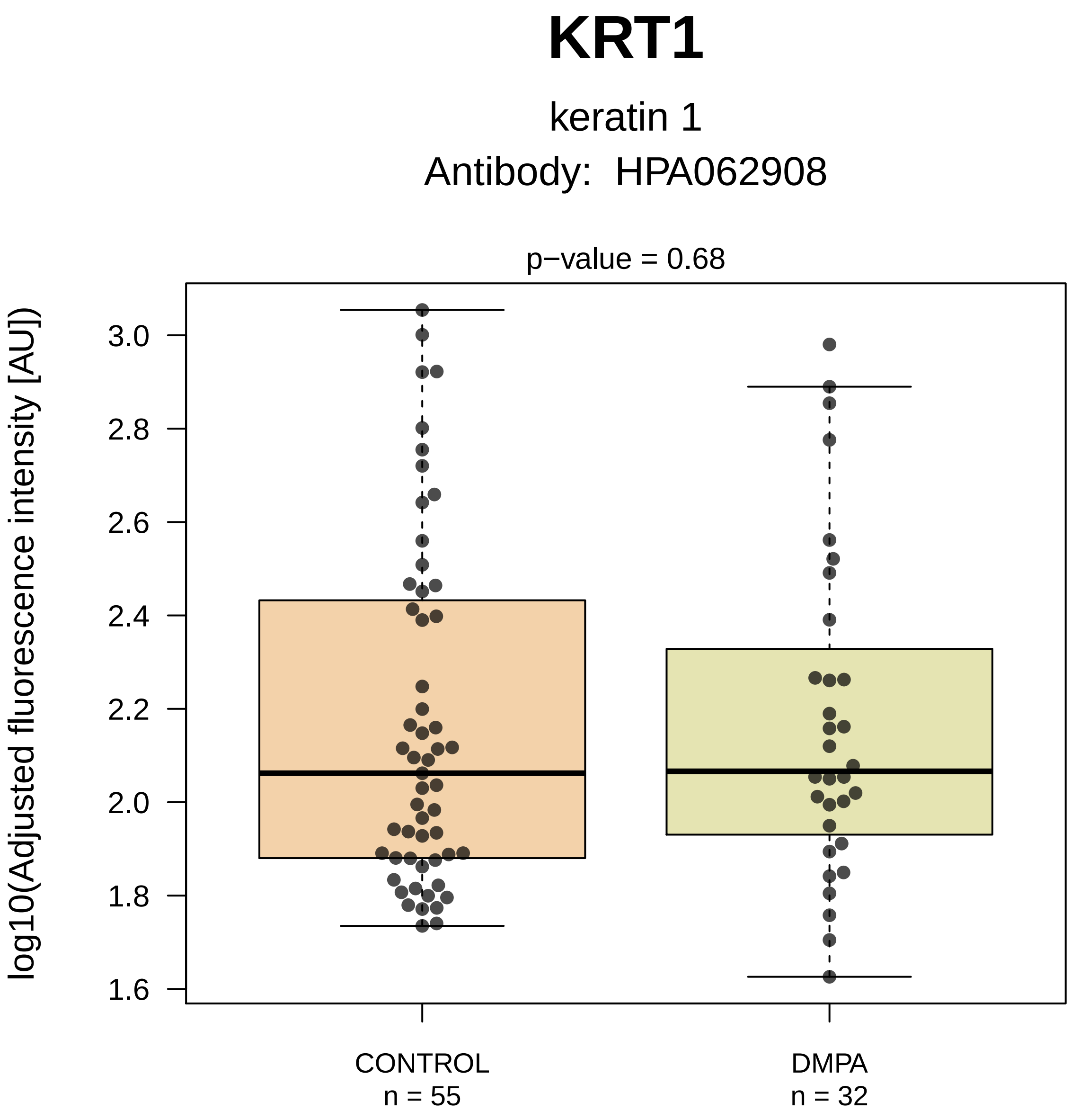
KRT1
keratin 1
Antibody: HPA062908
p−value = 0.68
log10(Adjusted fluorescence intensity [AU])
3.0
2.8
2.6
2.4
2.2
2.0
1.8
1.6
CONTROL
n = 55
DMPA
n = 32

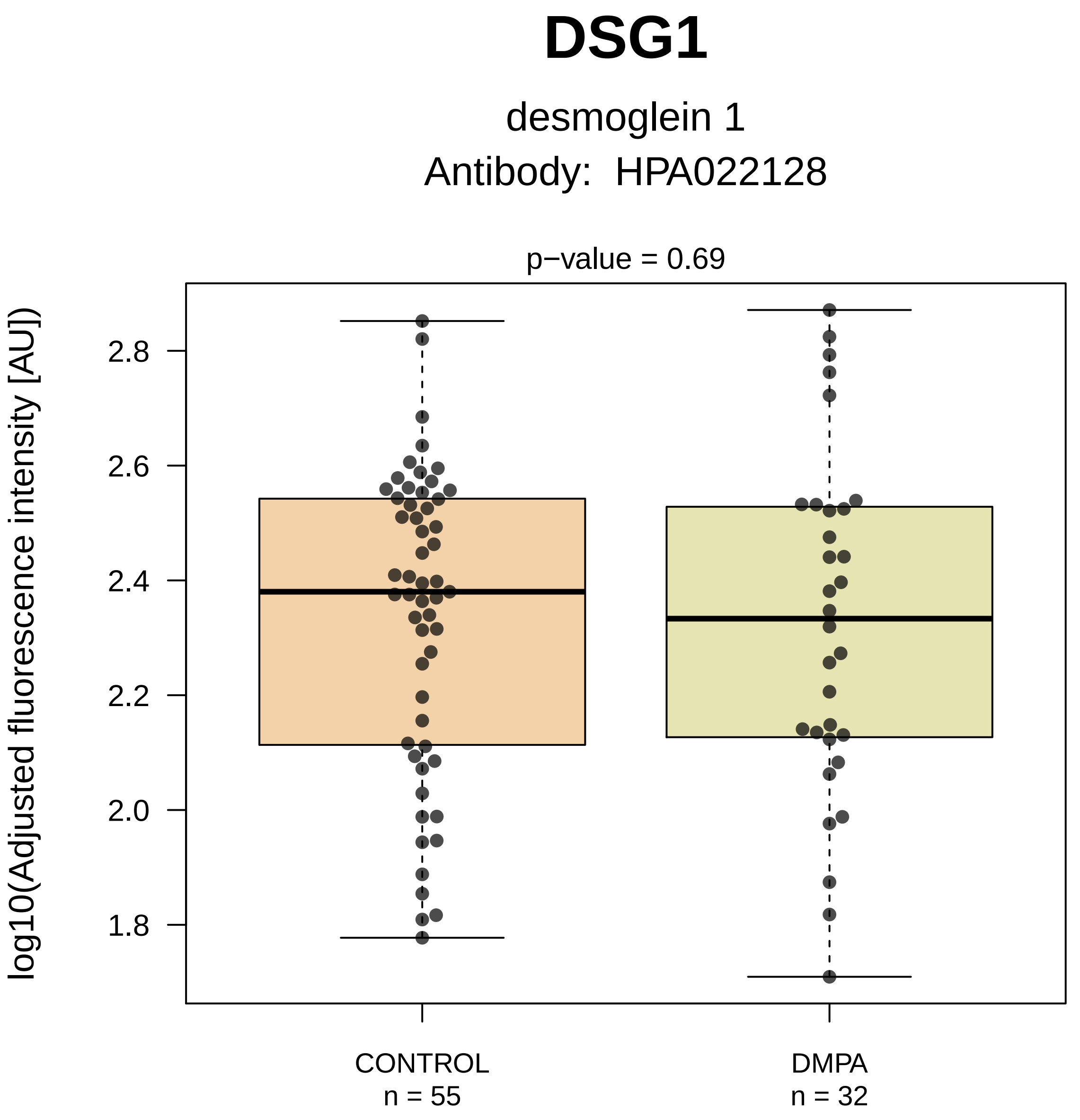
DSG1
desmoglein 1
Antibody: HPA022128
p−value = 0.69
log10(Adjusted fluorescence intensity [AU])
2.8
2.6
2.4
2.2
2.0
1.8
CONTROL
n = 55
DMPA
n = 32

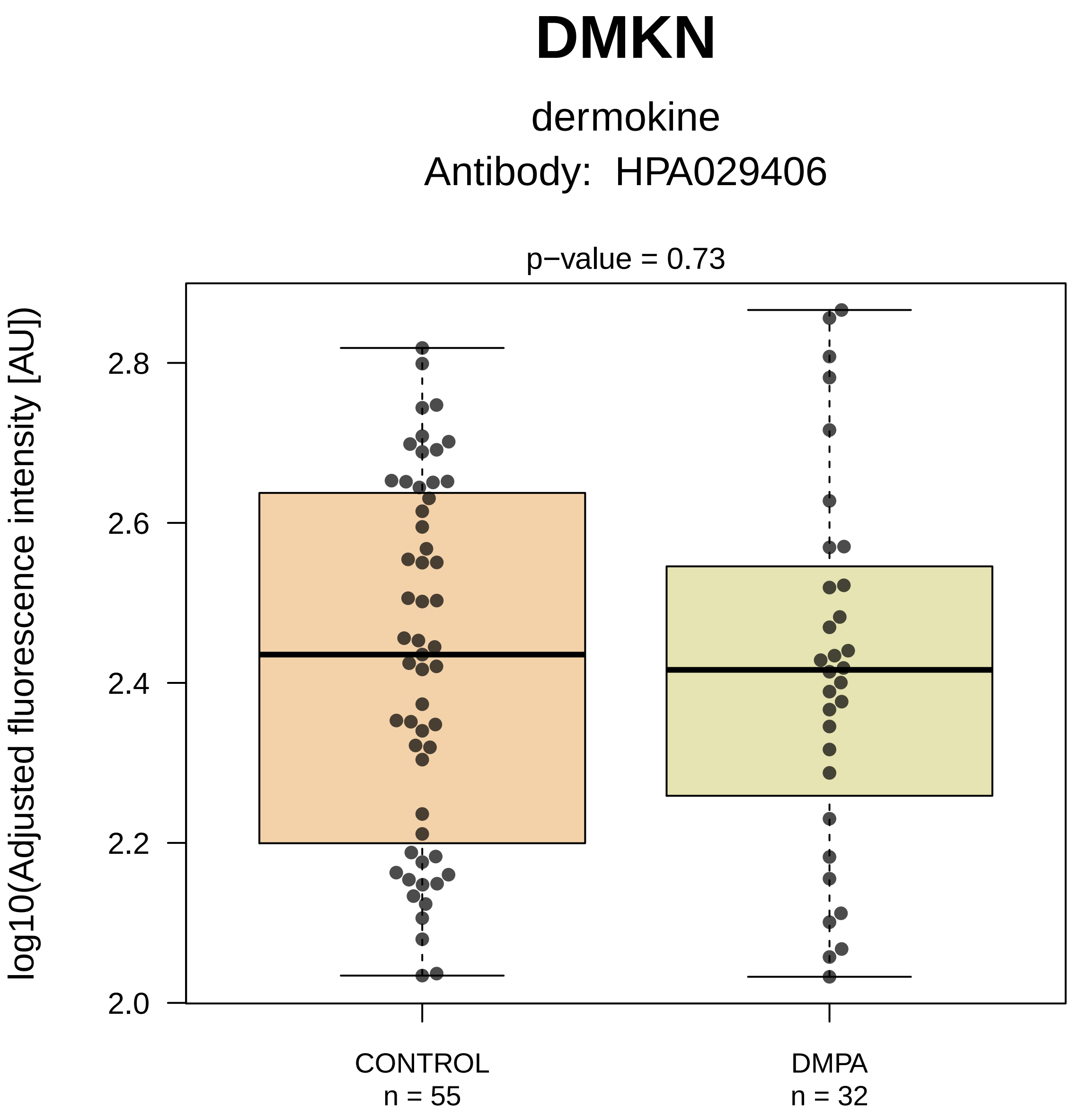

DMKN
dermokine
Antibody: HPA029406
p−value = 0.73
log10(Adjusted fluorescence intensity [AU])
2.0
2.2
2.4
2.6
2.8
CONTROL
n = 55
DMPA
n = 32

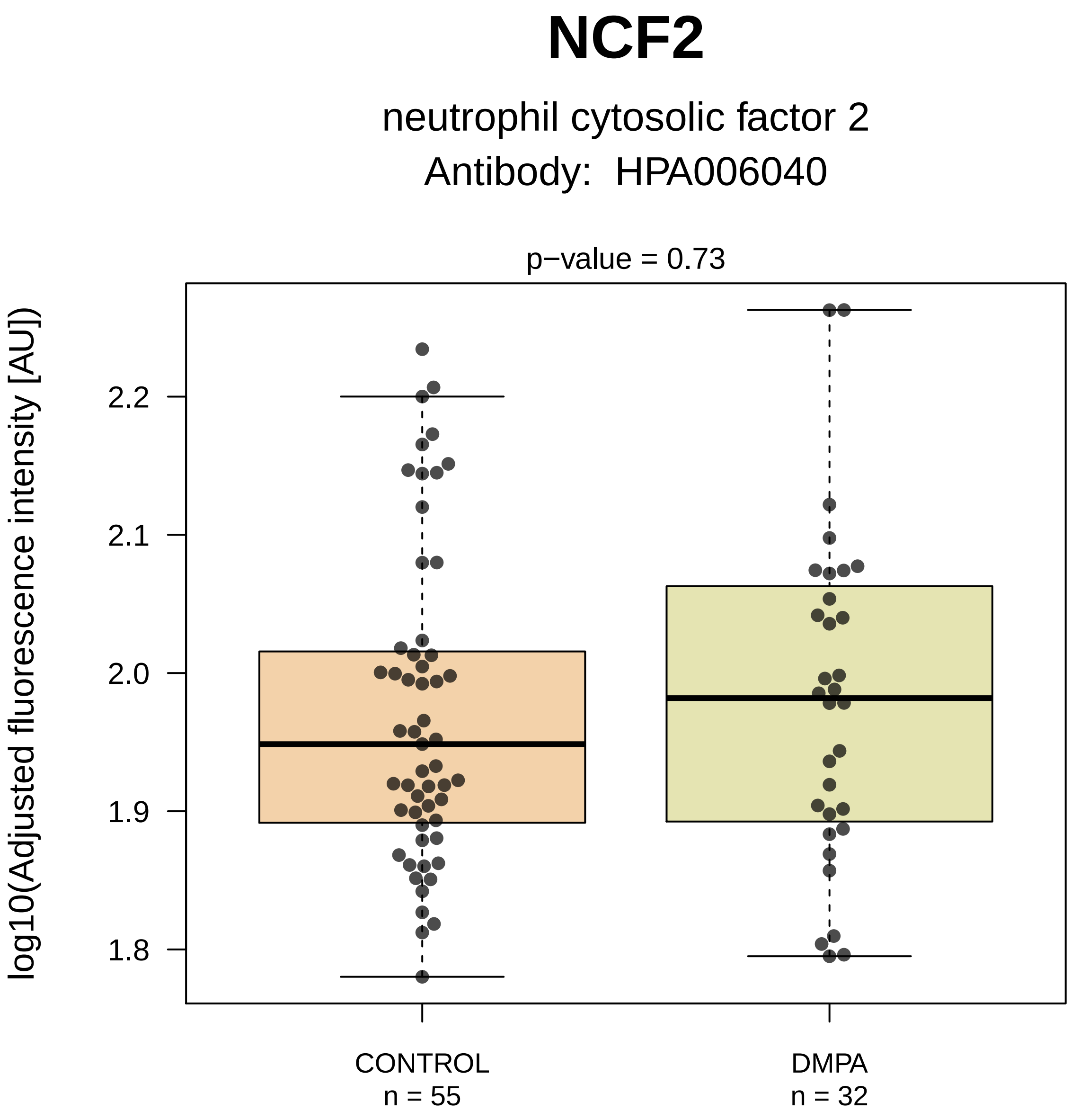
NCF2
neutrophil cytosolic factor 2
Antibody: HPA006040
p−value = 0.73
log10(Adjusted fluorescence intensity [AU])
2.2
2.1
2.0
1.9
1.8
CONTROL
n = 55
DMPA
n = 32

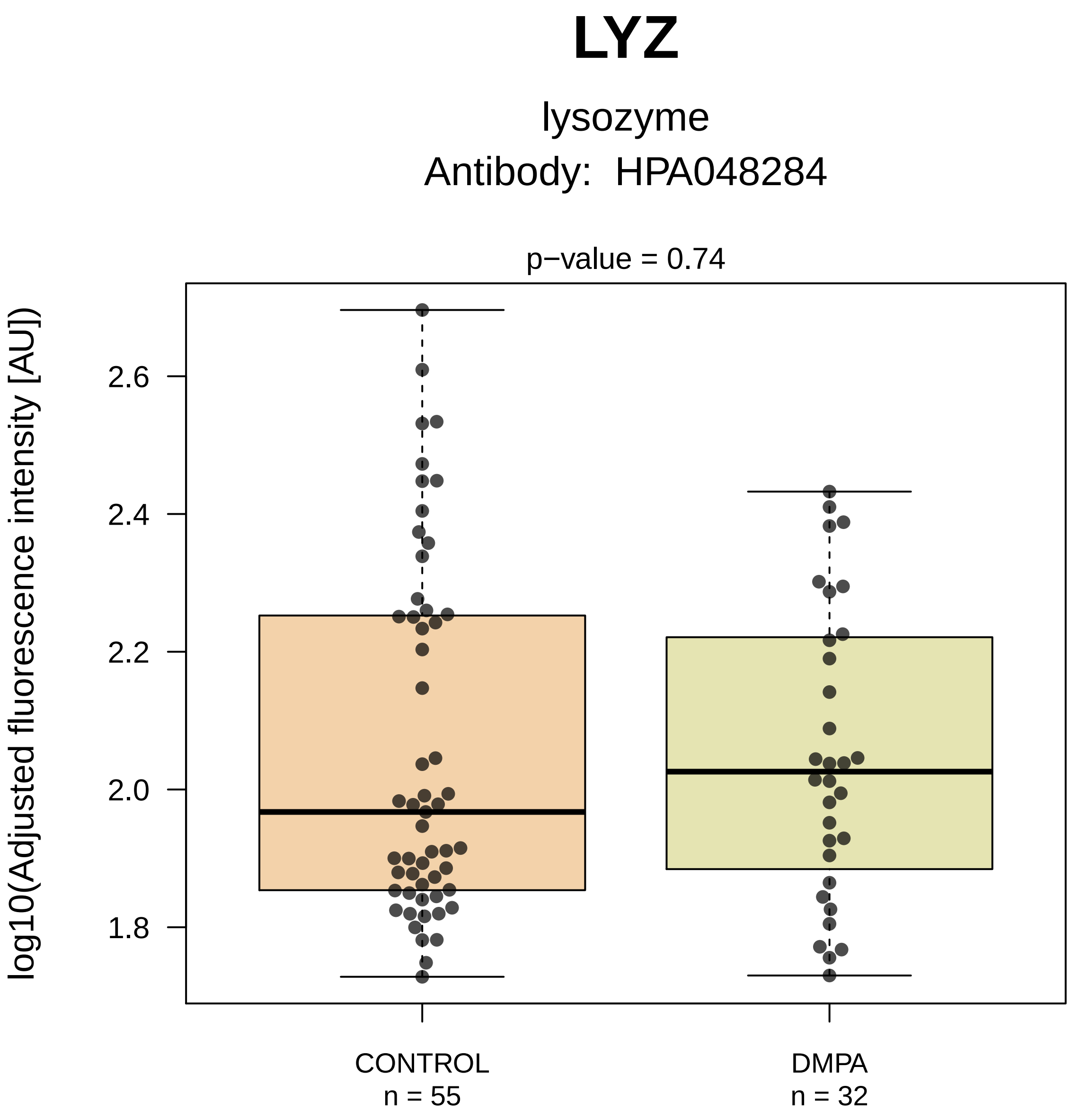
LYZ
lysozyme
Antibody: HPA048284
p−value = 0.74
log10(Adjusted fluorescence intensity [AU])
2.6
2.4
2.2
2.0
1.8
CONTROL
n = 55
DMPA
n = 32

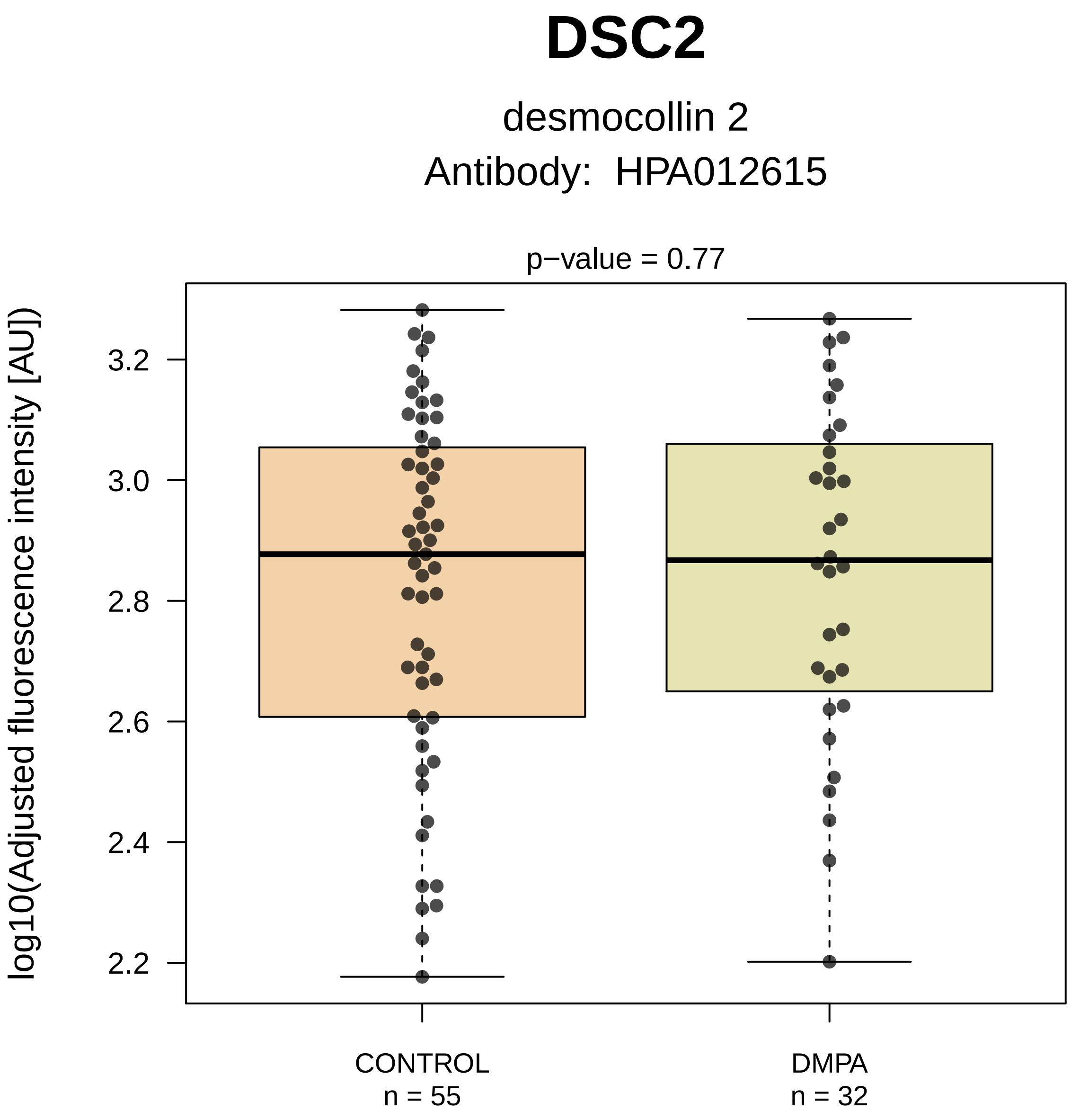
DSC2
desmocollin 2
Antibody: HPA012615
p−value = 0.77
log10(Adjusted fluorescence intensity [AU])
3.2
3.0
2.8
2.6
2.4
2.2
CONTROL
n = 55
DMPA
n = 32

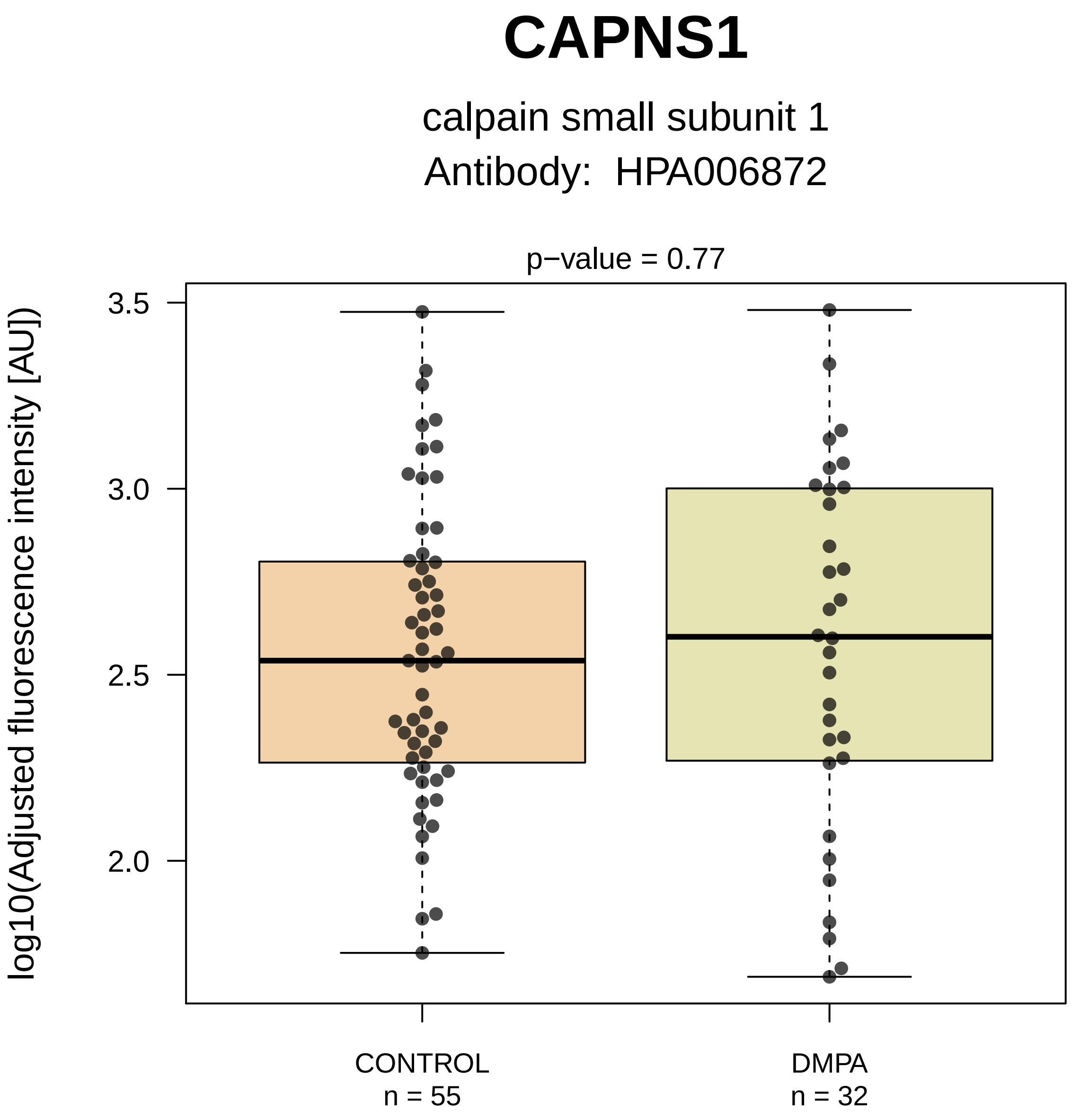
CAPNS1
calpain small subunit 1
Antibody: HPA006872
p−value = 0.77
log10(Adjusted fluorescence intensity [AU])
3.5
3.0
2.5
2.0
CONTROL
n = 55
DMPA
n = 32

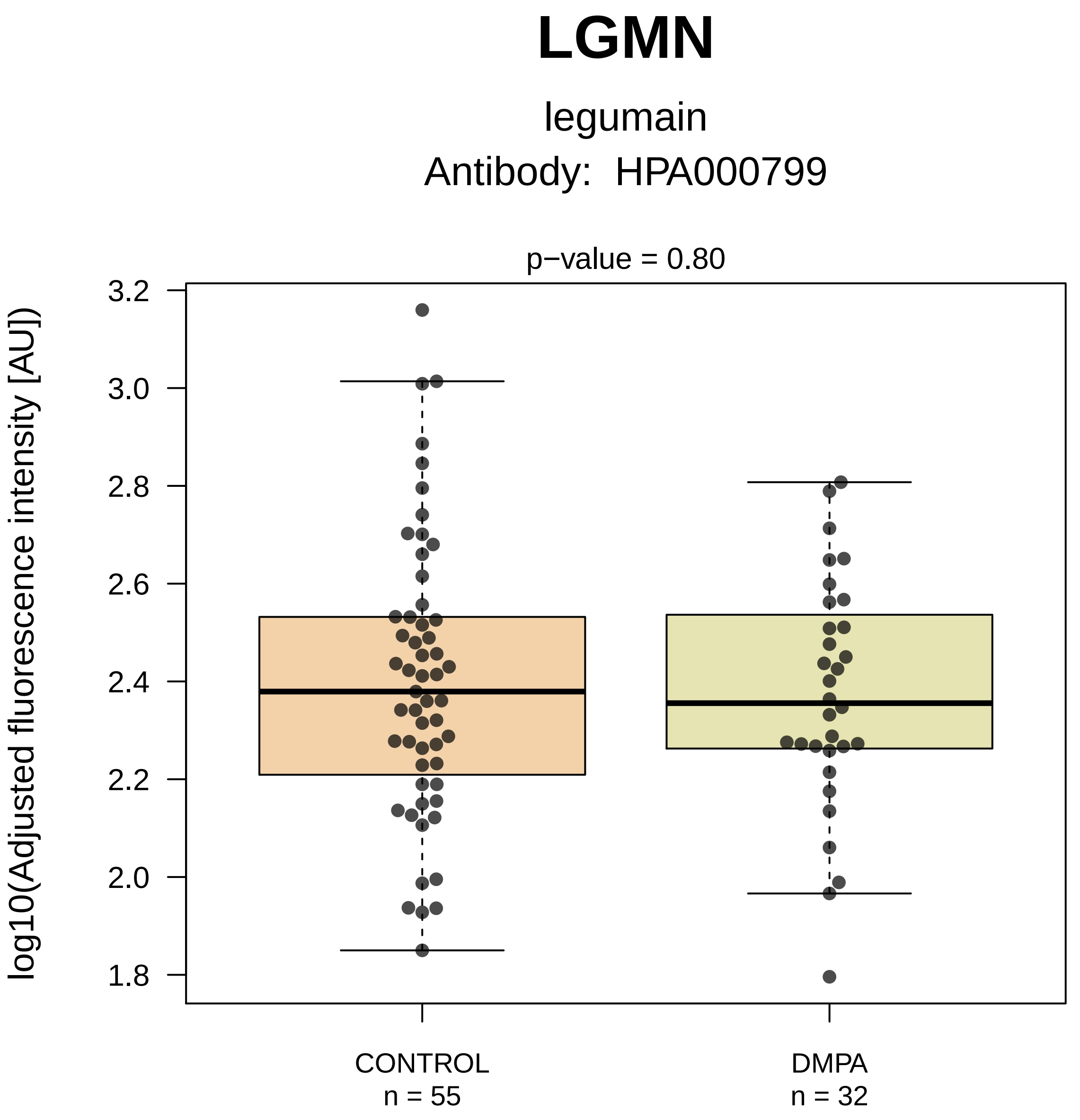
LGMN
legumain
Antibody: HPA000799
p−value = 0.80
log10(Adjusted fluorescence intensity [AU])
3.2
3.0
2.8
2.6
2.4
2.2
2.0
1.8
CONTROL
n = 55
DMPA
n = 32

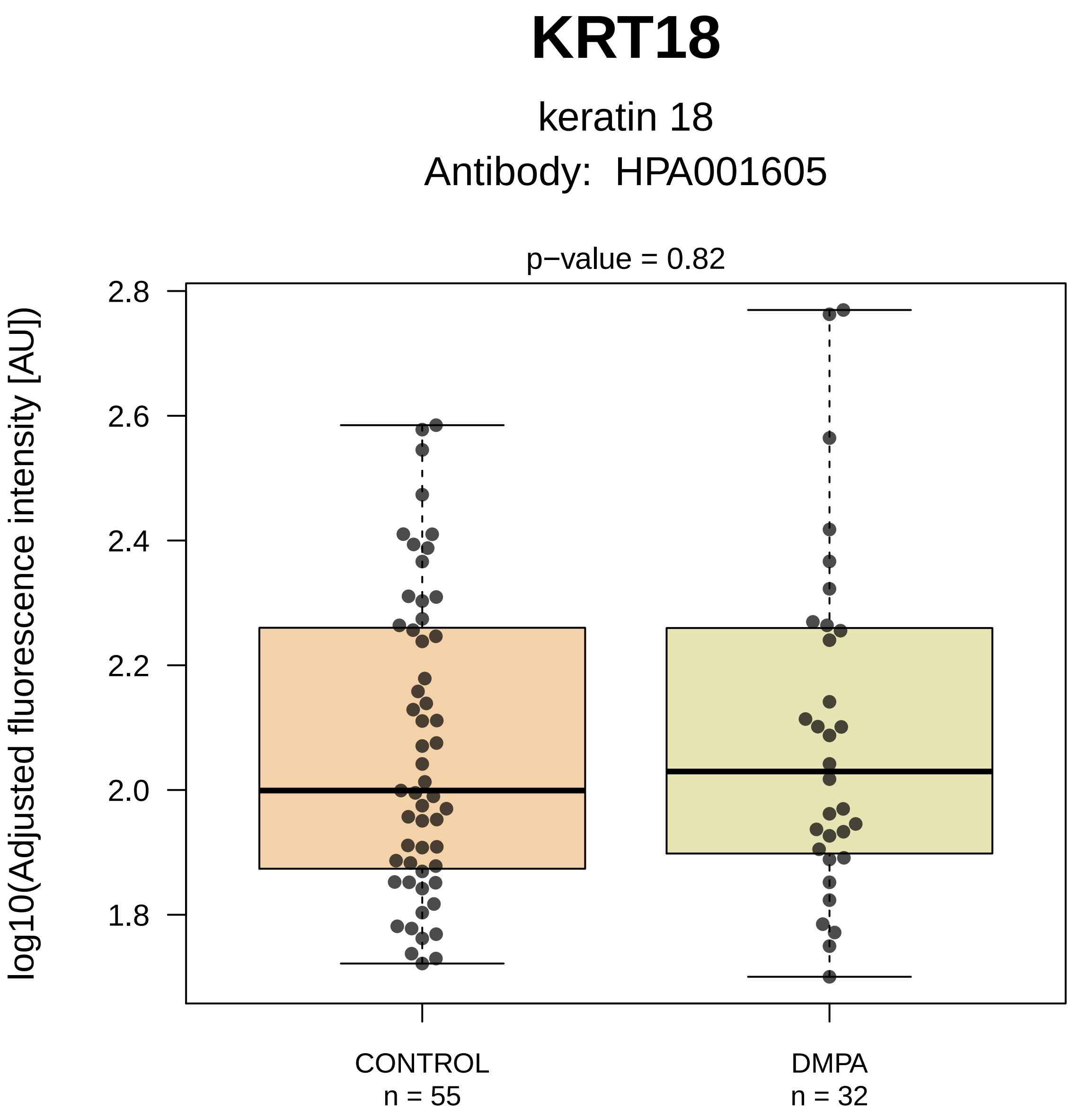
KRT18
keratin 18
Antibody: HPA001605
p−value = 0.82
log10(Adjusted fluorescence intensity [AU])
2.8
2.6
2.4
2.2
2.0
1.8
CONTROL
n = 55
DMPA
n = 32

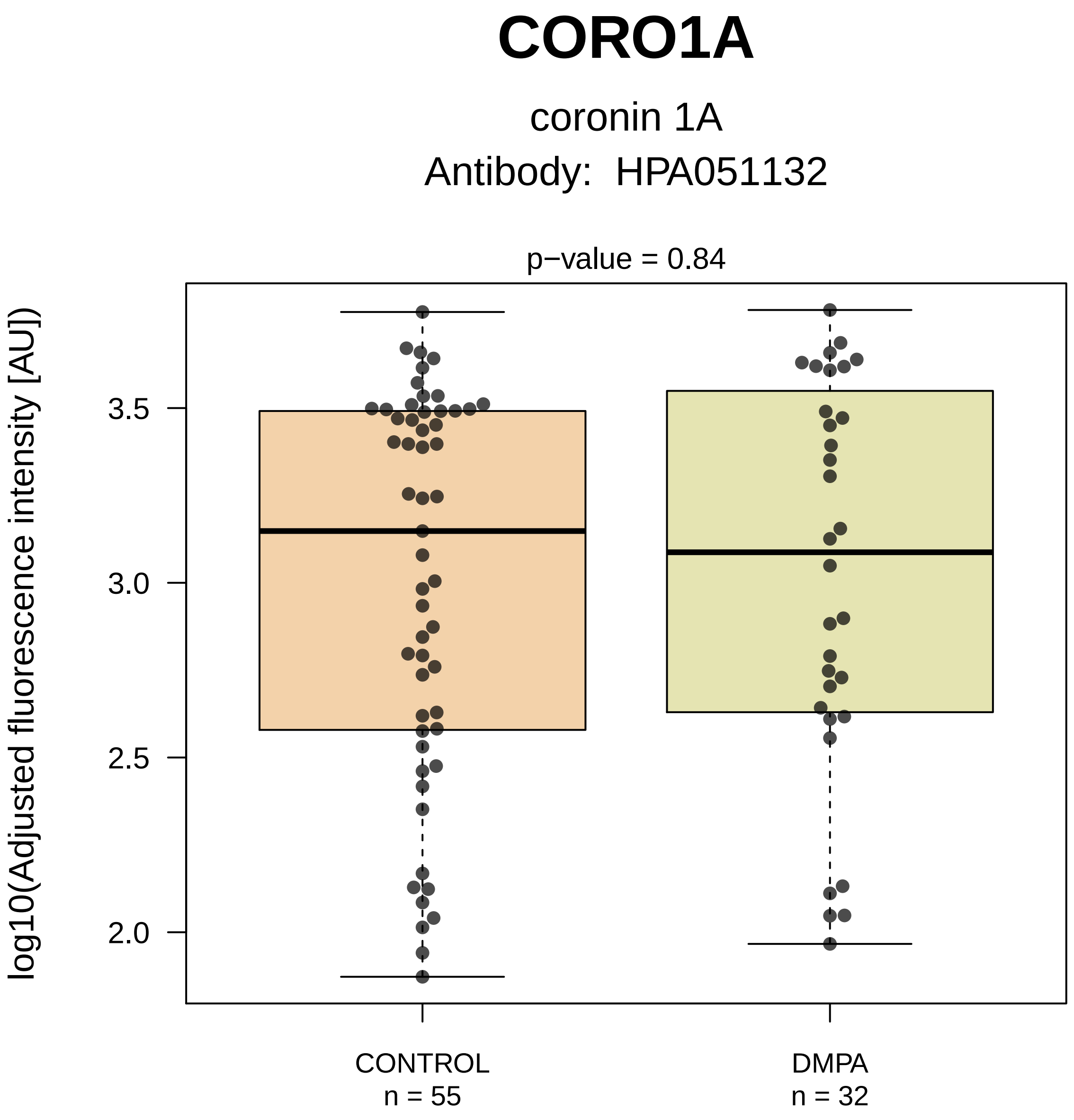

CORO1A
coronin 1A
Antibody: HPA051132
p−value = 0.84
log10(Adjusted fluorescence intensity [AU])
3.5
3.0
2.5
2.0
CONTROL
n = 55
DMPA
n = 32

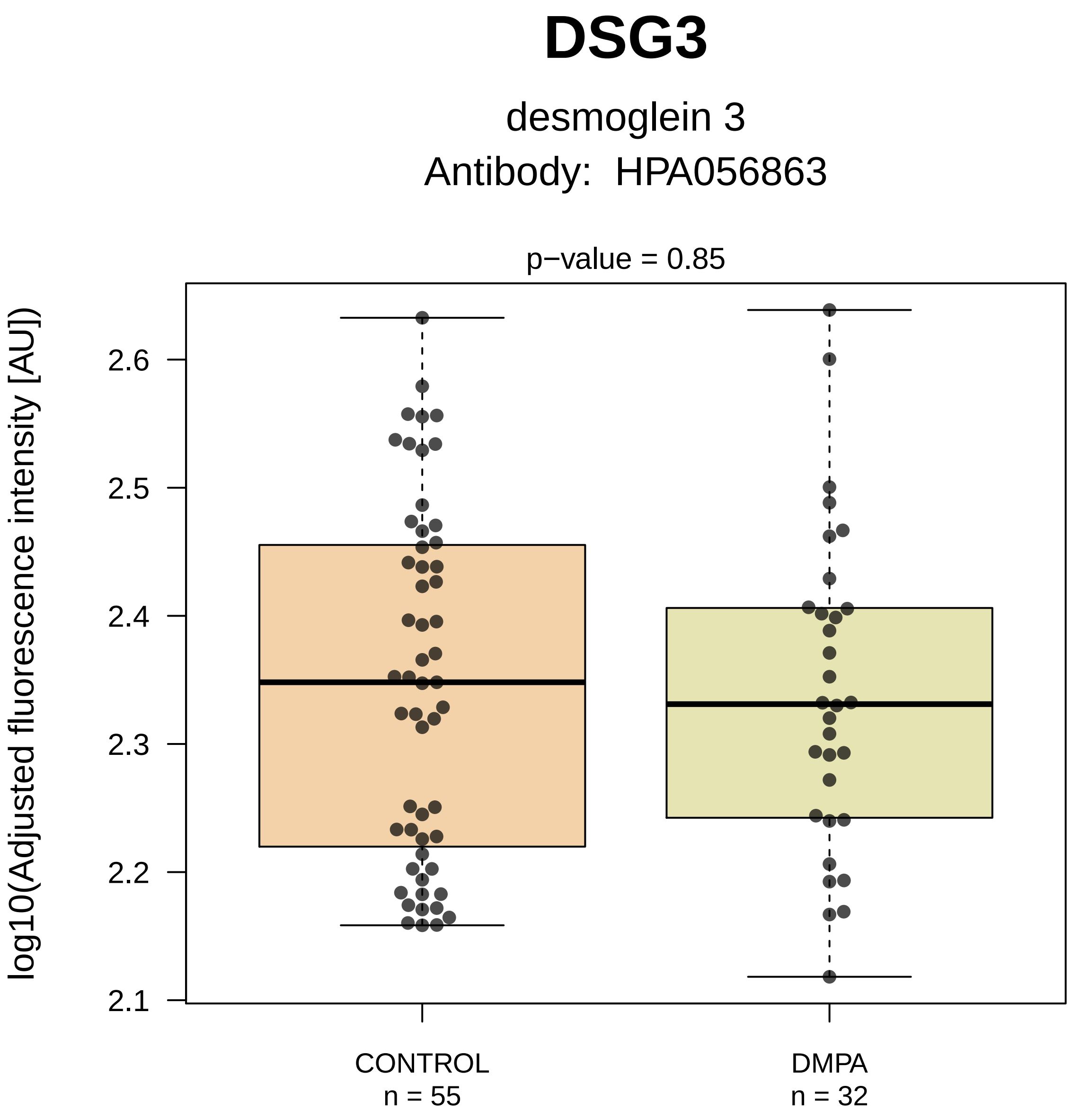
DSG3
desmoglein 3
Antibody: HPA056863
p−value = 0.85
log10(Adjusted fluorescence intensity [AU])
2.1
2.2
2.3
2.4
2.5
2.6
CONTROL
n = 55
DMPA
n = 32

KNG1

kininogen 1
Antibody: HPA001616

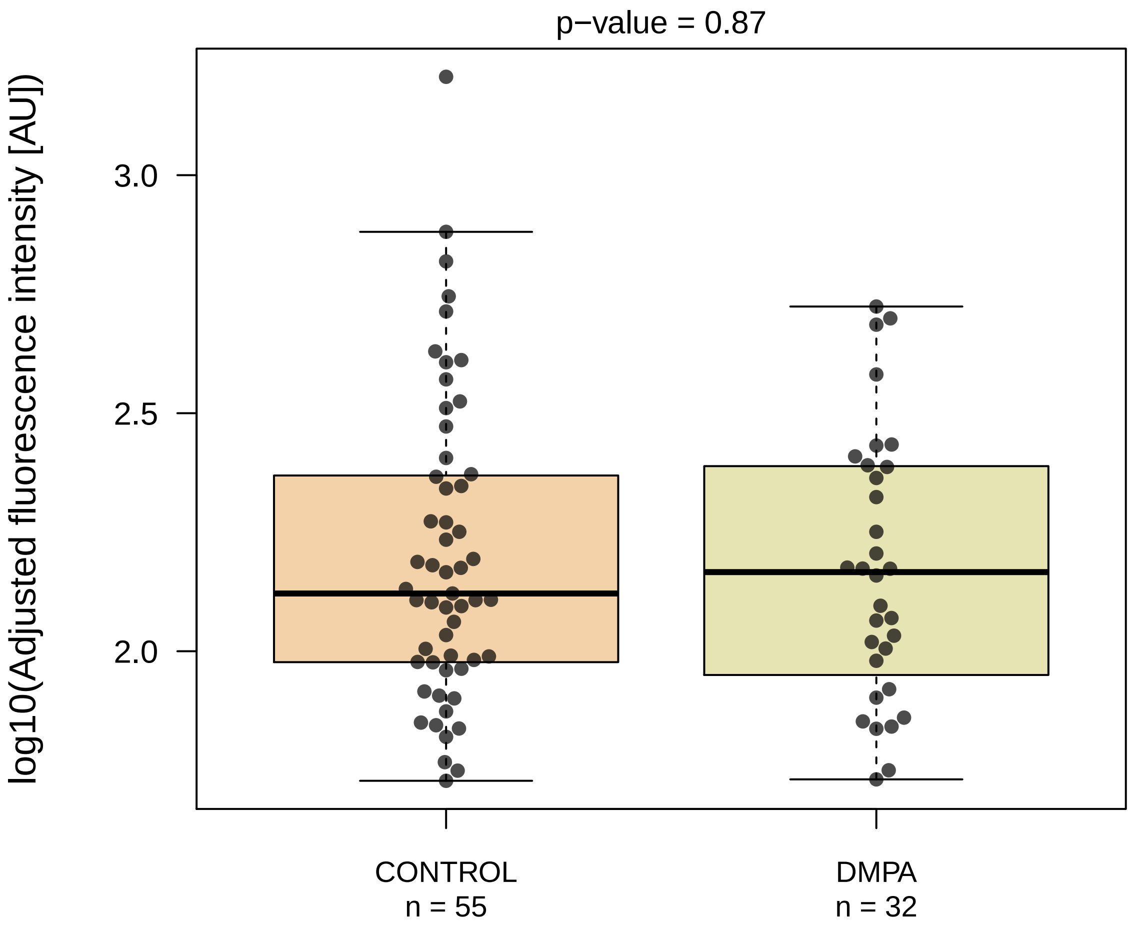

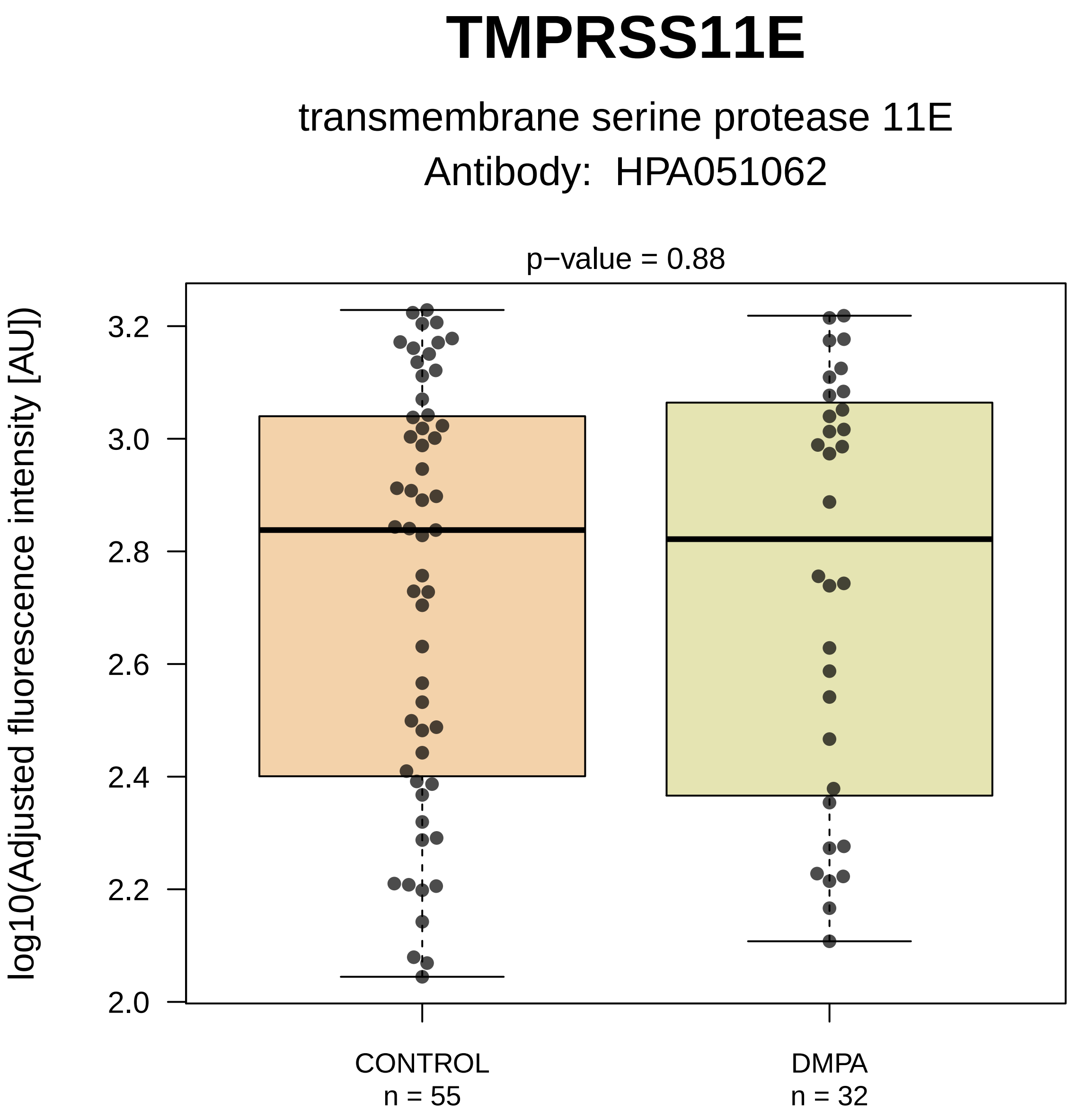
TMPRSS11E
transmembrane serine protease 11E
Antibody: HPA051062
p−value = 0.88
log10(Adjusted fluorescence intensity [AU])
3.2
3.0
2.8
2.6
2.4
2.2
2.0
CONTROL
n = 55
DMPA
n = 32

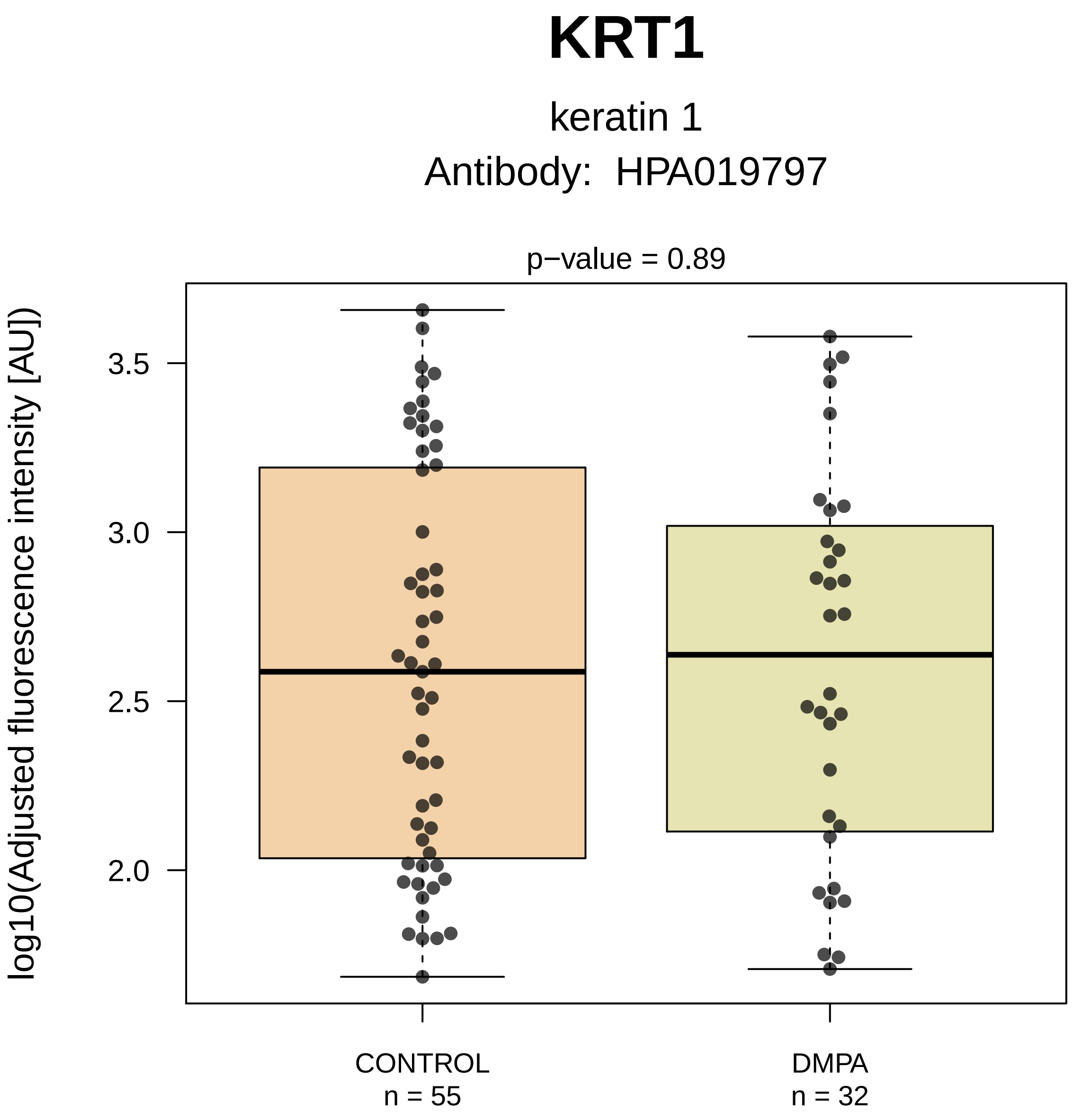
KRT1
keratin 1
Antibody: HPA019797
p−value = 0.89
log10(Adjusted fluorescence intensity [AU])
3.5
3.0
2.5
2.0
CONTROL
n = 55
DMPA
n = 32

AHSG

alpha 2−HS glycoprotein
Antibody: HPA001524

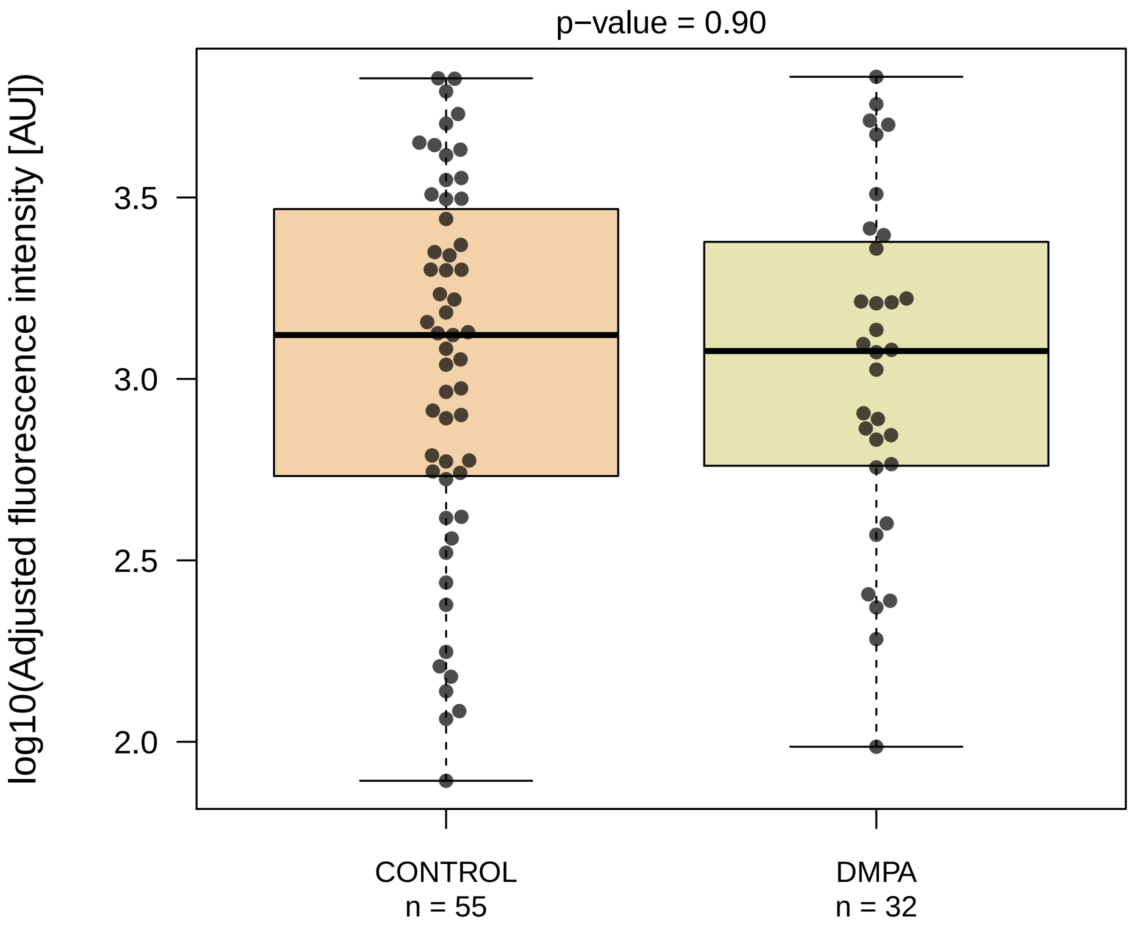

KRT14

keratin 14

Antibody: HPA023040

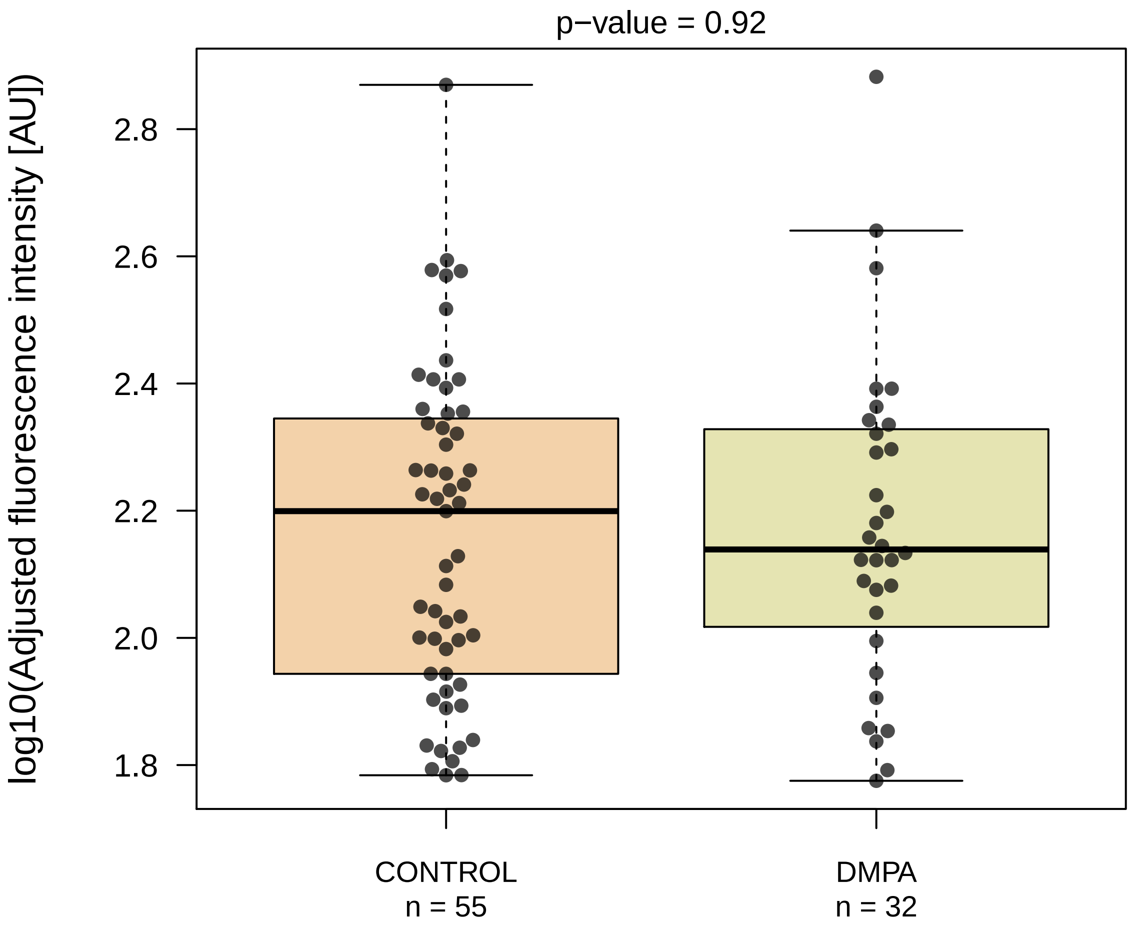

NCF2

neutrophil cytosolic factor 2
Antibody: HPA002327

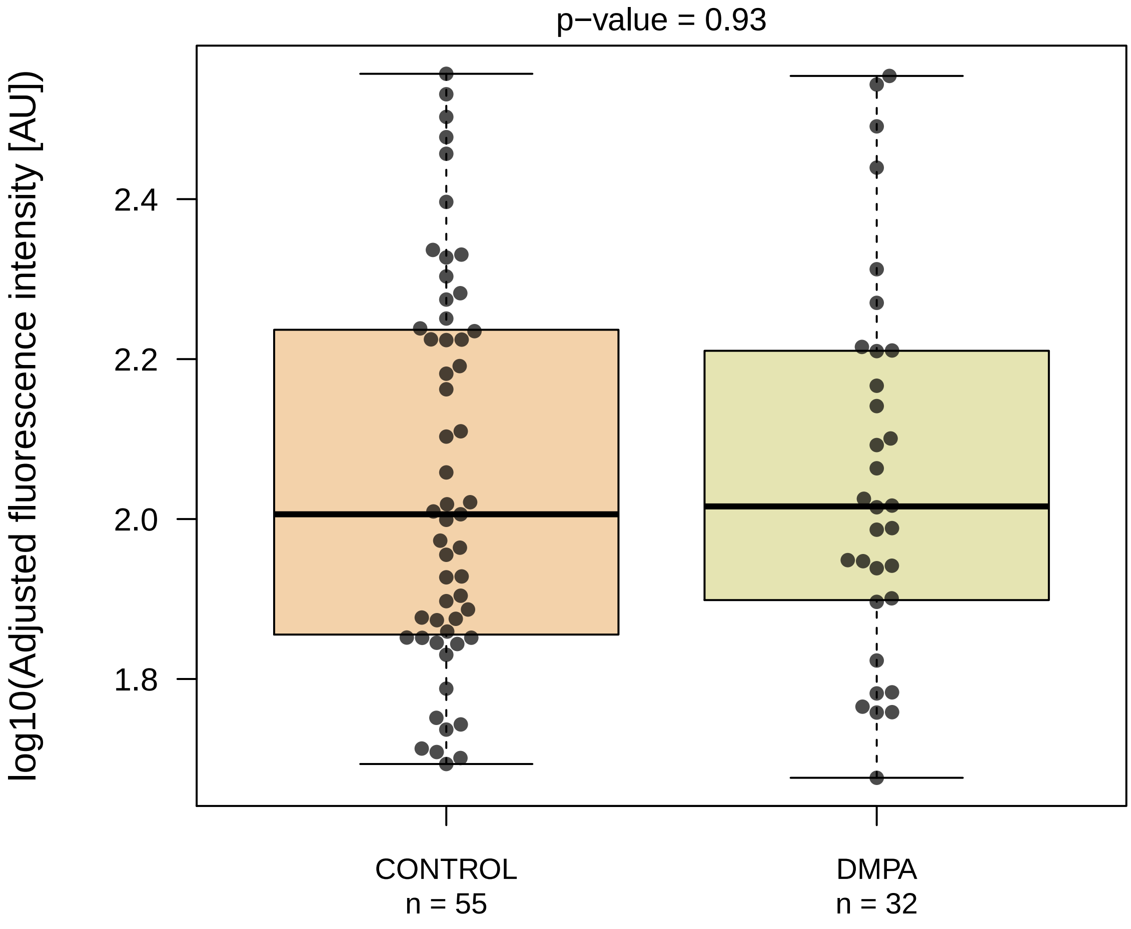

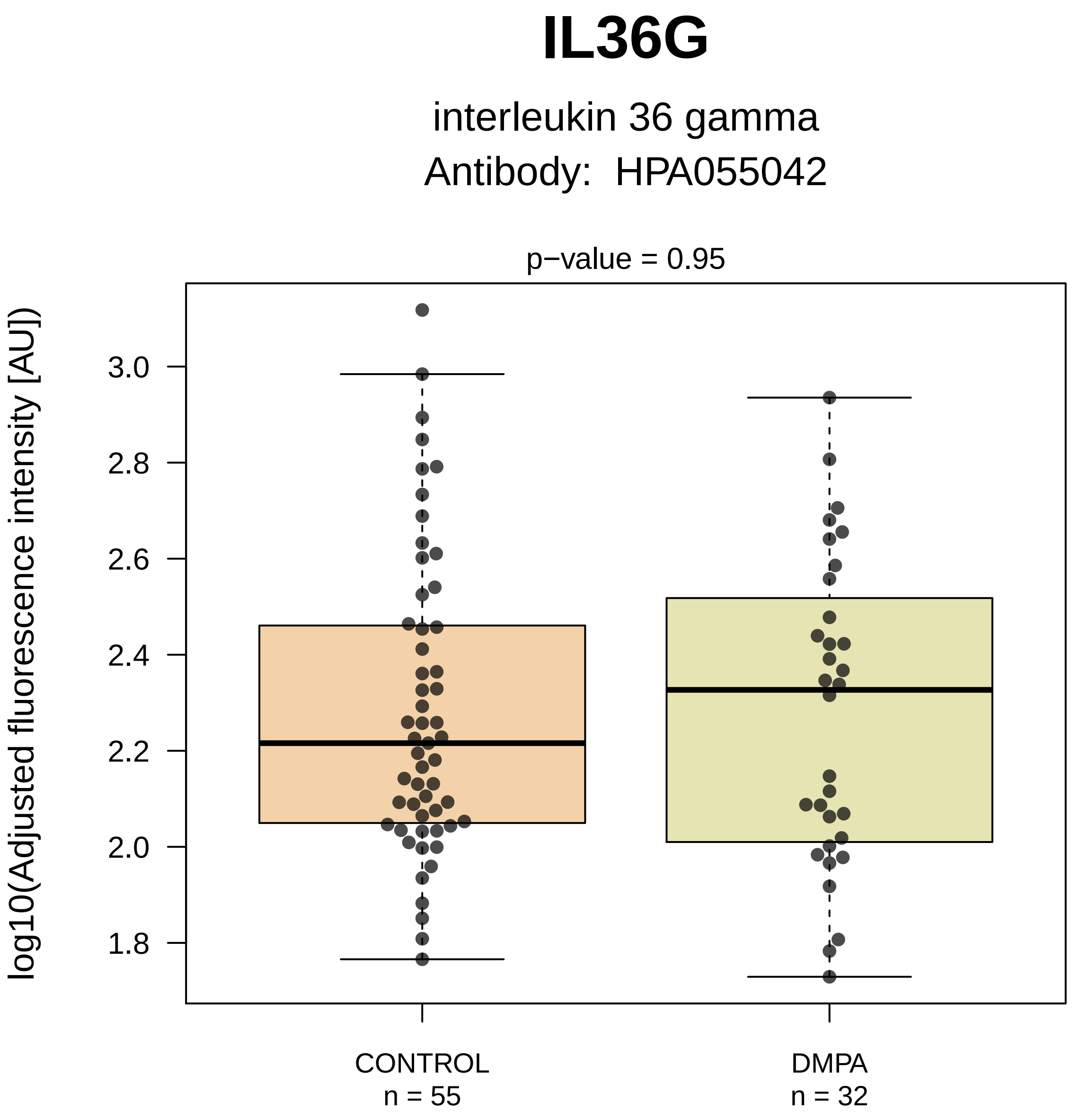
IL36G
interleukin 36 gamma
Antibody: HPA055042
p−value = 0.95
log10(Adjusted fluorescence intensity [AU])
3.0
2.8
2.6
2.4
2.2
2.0
1.8
CONTROL
n = 55
DMPA
n = 32

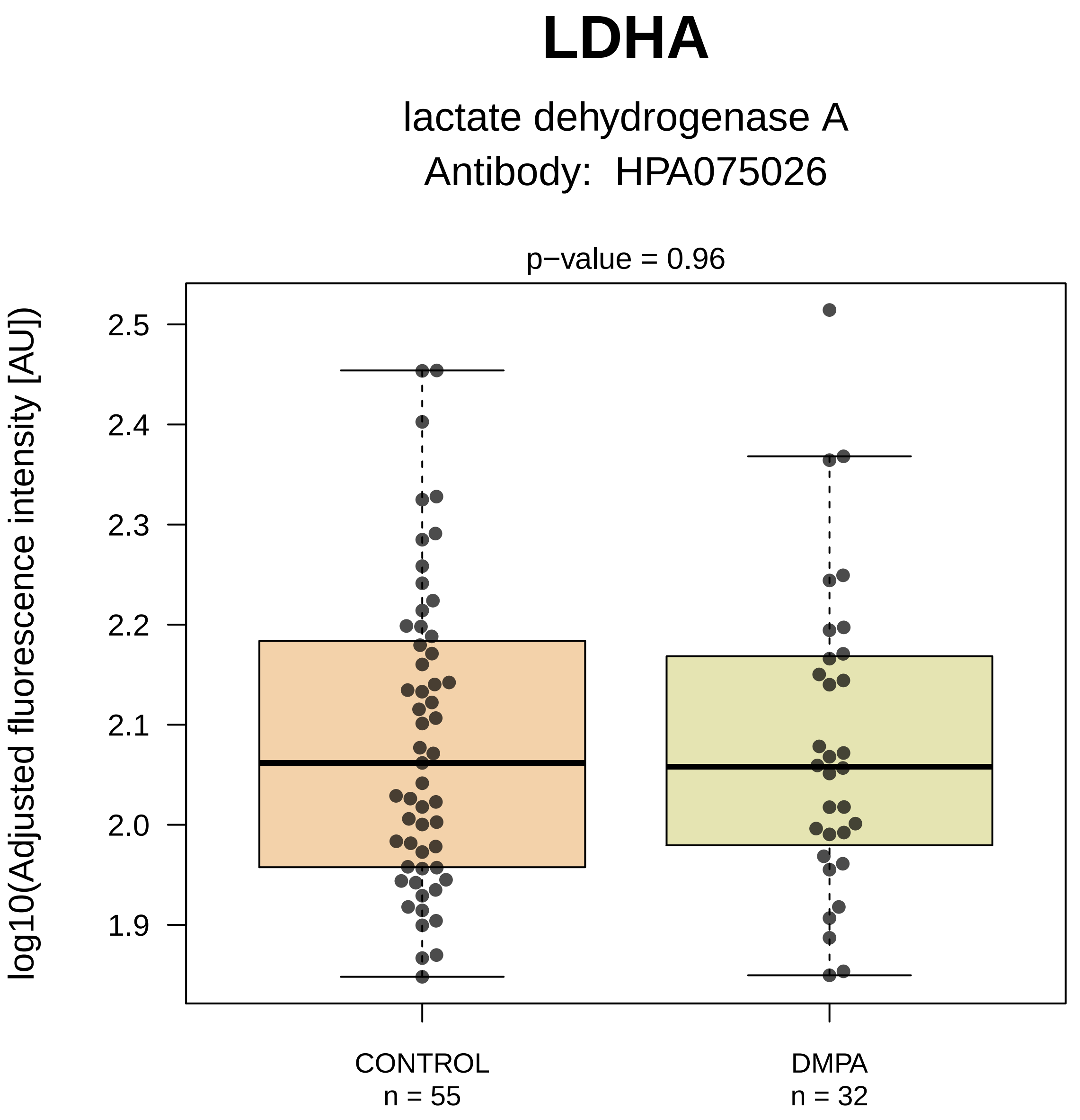
LDHA
lactate dehydrogenase A
Antibody: HPA075026
p−value = 0.96
log10(Adjusted fluorescence intensity [AU])
2.5
2.4
2.3
2.2
2.1
2.0
1.9
CONTROL
n = 55
DMPA
n = 32

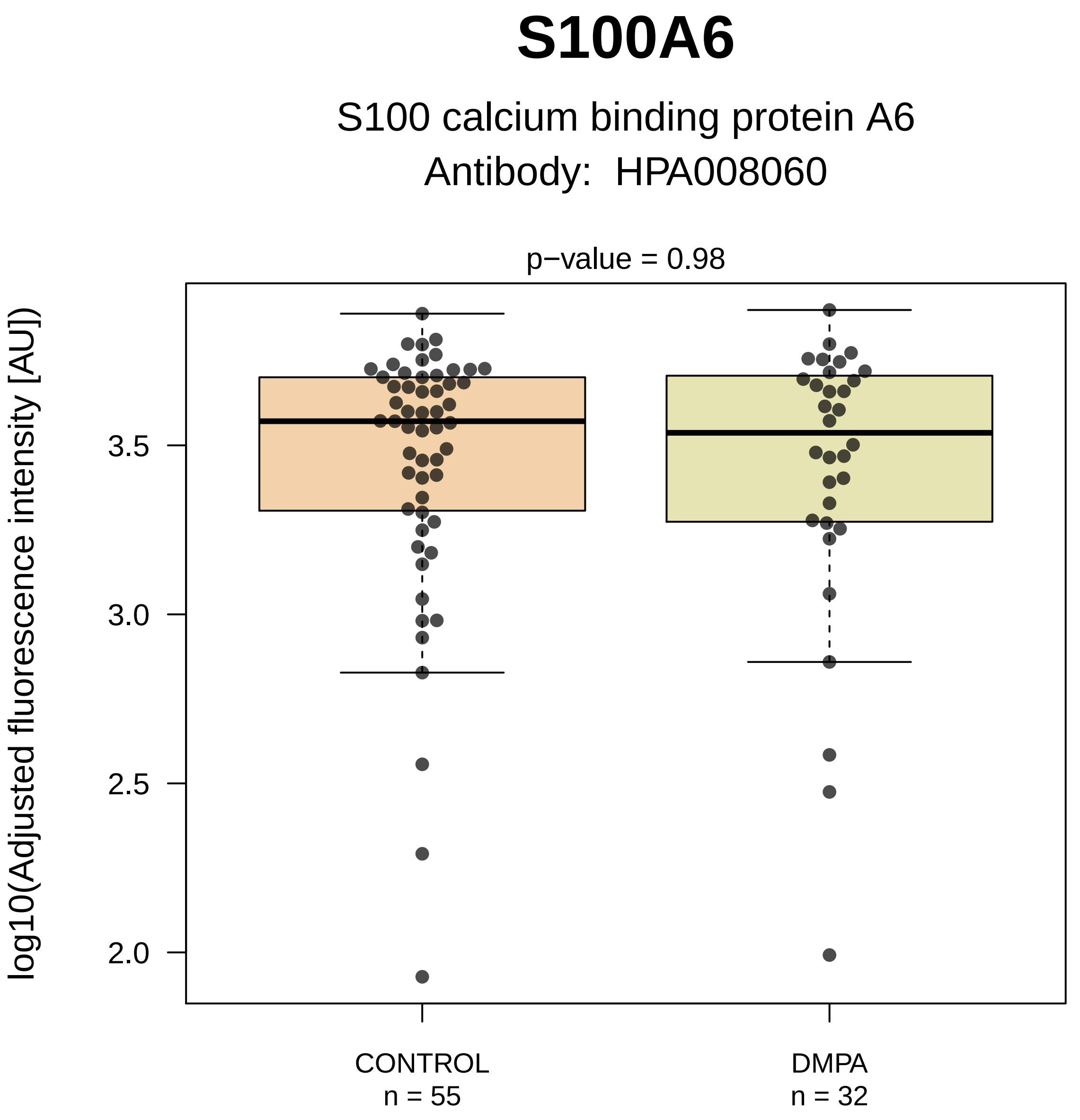
S100A6
S100 calcium binding protein A6
Antibody: HPA008060
p−value = 0.98
log10(Adjusted fluorescence intensity [AU])
3.5
3.0
2.5
2.0
CONTROL
n = 55
DMPA
n = 32

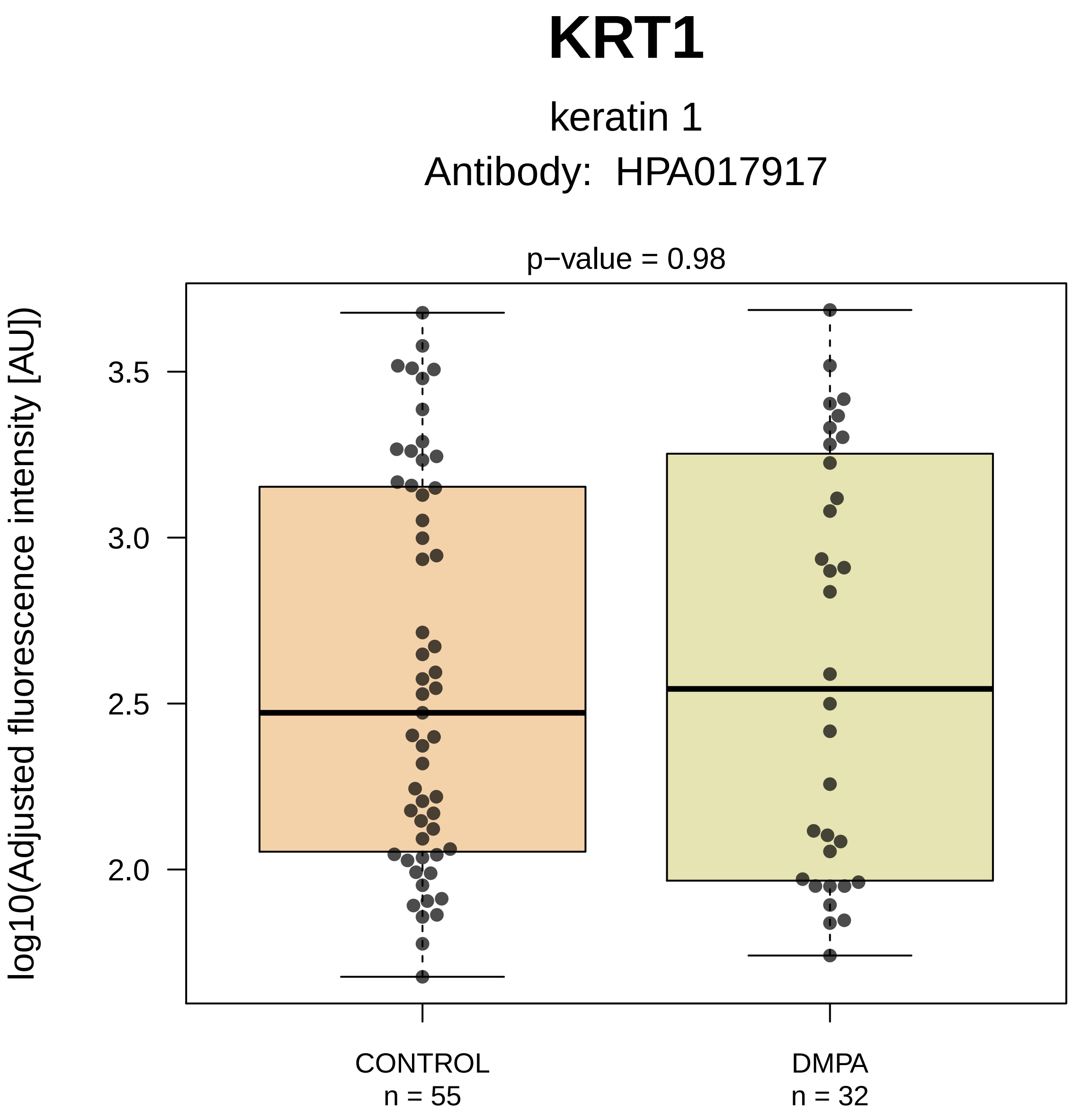
KRT1
keratin 1
Antibody: HPA017917
p−value = 0.98
log10(Adjusted fluorescence intensity [AU])
3.5
3.0
2.5
2.0
CONTROL
n = 55
DMPA
n = 32

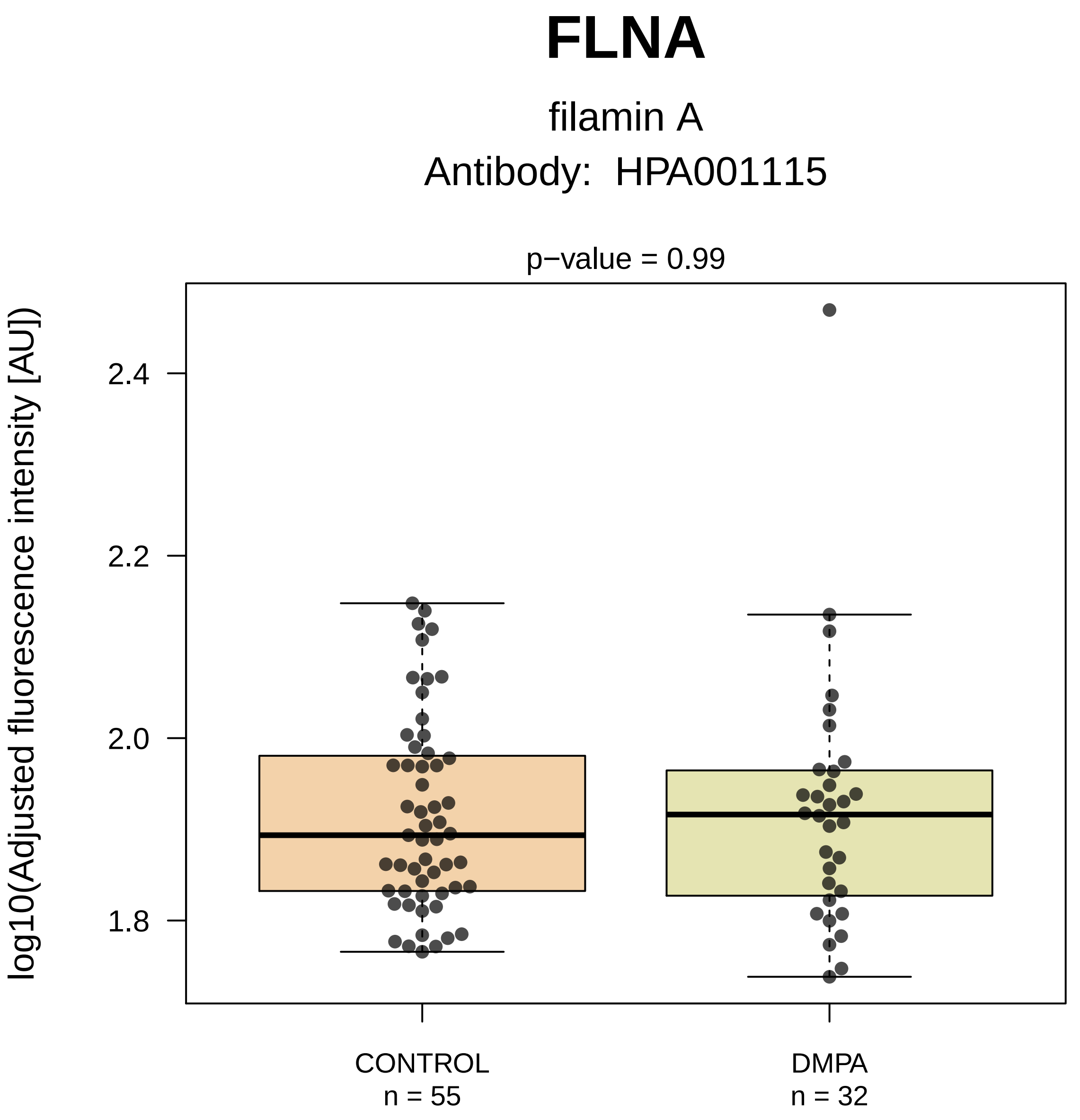
FLNA
filamin A
Antibody: HPA001115
p−value = 0.99
log10(Adjusted fluorescence intensity [AU])
2.4
2.2
2.0
1.8
CONTROL
n = 55
DMPA
n = 32
