# Supplementary material for: Multi-omics analysis of the cervical epithelial integrity of women using depot medroxyprogesterone acetate
Source: PLoS Pathog. 2022 May 9;18(5):e1010494. doi: 10.1371/journal.ppat.1010494 (PMC9119532; doi:10.1371/journal.ppat.1010494)
Supplement: S9 Table — (PDF) [file ppat.1010494.s014.pdf]

S9 Table. *Sociodemographic data and clinical characteristics of study subjects included in the in situ imaging analysis for desmoglein-1 and claudin-1 stainings*

	**DMPA group (n=27)**	**Control group (n=56)**	**P-value**
	Number or median (range or %)	Number or median (range or %)	
**Age (years)**	30 (22, 41)	34 (20, 50)	**0.01**[1]
**Months in sex work**	24 (3, 36)	36 (5, 372)	< **0.001**[1]
- Not available		1 (2%)	
**Self-reported days since onset of last menses***	N/A	9 (3, 44)	
- Not available		4 (7%)	
**Plasma hormone levels**			
Estradiol (pg/ml)	22 (22, 124)	89 (22, 405)	< **0.001**[1]
- Below LLD (22 pg/ml)**	15 (58%)	6 (11%)	< **0.001**[2]
- Not available	1 (4%)	-	
Progesterone (ng/ml)	0.05 (0.05, 0.09)	0.05 (0.05, 19)	< **0.001**[1]
- Below LLD (0.05 ng/ml)**	25 (96%)	28 (50%)	< **0.001**[2]
- Not available	1 (4%)	-	
**Having a regular partner**			0.74[2]
- Yes	11 (41%)	27 (48%)	
- No	15 (56%)	28 (50%)	
- Not available	1 (4%)	1 (2%)	
**Vaginal microbiome composition group**			0.51[3]
- L1 (*L.crispatus/jensenii*)	3 (11%)	6 (11%)	
- L2 (*L. iners*)	10 (37%)	16 (29%)	
- L3 (*Gardnerella*)	5 (19%)	12 (21%)	
- L4 (High diverse)	6 (22%)	20 (36%)	
- L5 (Other)	3 (11%)	2 (4%)	
**Bacterial Vaginosis (BV; based on Nugent Score)**			0.44[2]
- BV	5 (19%)	16 (29%)	
- Intermediate	5 (19%)	15 (27%)	
- Normal	15 (56%)	25 (45%)	
- Not available	2 (7%)	0	

* Only applicable for samples in control group
** Percentage based on total number of samples with values (i.e. excluding samples with not available) in denominator, n= 26 in DMPA group and n= 56 in the control group
LLD: Lower limit of detection. N/A: not applicable.
[1] Mann Whitney U test. [2] Pearson's Chi-squared test. [3]Fischer's exact test. Significant p-values in bold
