# Supplementary material for: Multi-omics analysis of the cervical epithelial integrity of women using depot medroxyprogesterone acetate
Source: PLoS Pathog. 2022 May 9;18(5):e1010494. doi: 10.1371/journal.ppat.1010494 (PMC9119532; doi:10.1371/journal.ppat.1010494)
Supplement: S13 Table — (PDF) [file ppat.1010494.s018.pdf]

S13 Table. *Sociodemographic data and clinical characteristics of study subjects included in the protein profiling assay*

	DMPA group (n=32)	Control group (n=55)	P-value
	Number or median (range or %)	Number or median (range or %)	
**Age (years)**	30 (22, 41)	33 (20, 50)	0.06[1]
**Months in sex work**	24 (3, 36)	36 (4, 372)	**< 0.001**[1]
- Not available		1 (2%)	
**Self-reported days since onset of last menses***	N/A	9 (4, 44)	
- Not available		4 (7%)	
**Plasma hormone levels**			
Estradiol (pg/ml)	22 (22-124)	92 (22-405)	**< 0.001**[1]
- Below LLD (22 pg/ml)**	17 (55%)	6 (11%)	**< 0.001**[2]
- Not available	1 (3%)	1 (2%)	
Progesterone (ng/ml)	0.05 (0.05-0.09)	0.05 (0.05-19)	**< 0.001**[1]
- Below LLD (0.05 ng/ml)**	30 (97%)	30 (56%)	**< 0.001**[2]
- Not available	1 (3%)	1 (2%)	
**Having a regular partner**			0.90[2]
- Yes	19 (59%)	31 (56%)	
- No	12 (38%)	23 (42%)	
- Not available	1 (3%)	1 (2%)	
**Vaginal microbiome composition group**			0.68[3]
- L1 (*L.crispatus/jensenii*)	3 (9%)	6 (11%)	
- L2 (*L. iners*)	11 (34%)	15 (27%)	
- L3 (*Gardnerella*)	6 (19%)	12 (22%)	
- L4 (High diverse)	8 (25%)	19 (35%)	
- L5 (Other)	4 (12%)	3 (5%)	
**Bacterial Vaginosis (BV; based on Nugent Score)**			0.52[2]
- BV	7 (22%)	17 (31%)	
- Intermediate	5 (16%)	13 (24%)	
- Normal	17 (53%)	25 (45%)	
- Not available	3 (9%)	0	

*Only applicable for samples in control group
** Percentage based on total number of samples with values (i.e. excluding samples with not available) in denominator, n= 31 in DMPA group and n= 54 in control group
LLD: Lower limit of detection. N/A: not applicable.
[1] Mann Whitney *U* test. **[2]** Pearson's Chi-squared test. [3]Fischer's exact test. Significant p-values in bold.
