# Supplementary material for: Multi-omics analysis of the cervical epithelial integrity of women using depot medroxyprogesterone acetate
Source: PLoS Pathog. 2022 May 9;18(5):e1010494. doi: 10.1371/journal.ppat.1010494 (PMC9119532; doi:10.1371/journal.ppat.1010494)
Supplement: S14 Table — (PDF) [file ppat.1010494.s019.pdf]

S14 Table. *Adjustment of protein levels for age and time in sex work using generalized linear models.*

HGNC ID	*Control vs DMPA p-value*	*Age p-value*	*Time in sex work p-value*
GPX3	0.125	0.258	**0.025**
ITIH2	**0.004**	0.916	0.344
SERPINB1	**0.031**	0.981	0.289

Difference in protein levels between controls vs DMPA group with adjustment for potential confounders (age and time in sex work). P-values calculated using generalized linear models. $P< 0.05$ considered significant and marked in bold.
