# Supplementary material for: Multi-omics analysis of the cervical epithelial integrity of women using depot medroxyprogesterone acetate
Source: PLoS Pathog. 2022 May 9;18(5):e1010494. doi: 10.1371/journal.ppat.1010494 (PMC9119532; doi:10.1371/journal.ppat.1010494)
Supplement: S1 Text — (PDF) [file ppat.1010494.s021.pdf]

S1 Text

16S ribosomal RNA (rRNA) gene sequencing to determine cervicovaginal microbiome composition

After nucleic acids from the cervicovaginal lavage (CVL) pellet were extracted and the V4 region of the 16S rRNA gene was sequenced as described previously [1], the samples were stratified into five study groups. The following classification was used: L1: More than 90% relative abundance of *L. crispatus* and/or *L. jensenii*; L2: More than 80% *Lactobacillus spp*. (mainly *L. iners);* L3: *Gardnerella spp.* or *Gardnerella/Lactobacillus* mixed*;* L4: Mixed bacterial flora/High Diverse; L5: Bacterial flora not assigned to the other groups (Other).

Preparation of tissues for library preparation and RNA sequencing (RNA-seq) analysis

Frozen ectocervical biopsies stored in RNAlater were thawed, placed in RLT Plus Lysis Buffer (QIAGEN, Hilden, Germany), and homogenized using a TissueLyzer II machine (QIAGEN). RNA was isolated and fpurified using the AllPrep DNA/RNA Mini Kit (QIAGEN) and the QIAcube Connect (QIAGEN). RNA quantities were determined using a NanoDrop ND-1000 spectrophotometer (Thermo Fisher Scientific, Waltham, MA, USA), and RNA integrity number was assessed by the Agilent 2200 TapeStation System (Agilent Technologies, Santa Clara, CA, USA). Poly-A containing mRNA transcripts were purified using oligo (dT)-linked magnetic beads with the TruSeq mRNA-Seq Library Prep Kit (Illumina, San Diego, CA, USA), according to manufacturer instructions. This mRNA was then fragmented adding Fragmentation Mix solution (Illumina) and incubating under elevated temperatures in the presence of divalent cations. Fragmented mRNA was converted into first-strand complementary DNA (cDNA) using reverse transcriptase enzymes and random primers, and the second-strand cDNA was synthesized using DNA Polymerase I. Improved specificity in the synthesis of the second strand was accomplished by replacing dTTP with dUTP. A single adenylate nucleotide was added to the 3´end to prevent strands ligating to each other, and index adapters were ligated to the ends of the double-stranded cDNA fragments, enabling hybridization onto the flow cell. The cDNA fragments (approximately ~300 base pairs) were amplified with PCR and gel purified. Size and quality of the cDNA libraries were determined using a Bioanalyzer 2100 (Agilent Technologies), and the barcoded cDNA libraries were normalized and pooled in equal volumes. These pooled cDNA libraries were then denatured and diluted, and 1.7 pM of each pool was loaded onto a reagent cartridge (Illumina) for subsequent cluster generation and sequencing in a NextSeq 550 (Illumina).

Sequencing cycle parameters included 75 cycles of single-read sequencing, plus eight cycles for index read 1 and index read 2.

Gene set enrichment and transcription factor analysis

In order to identify gene sets associated with each study group, differentially expressed genes (DEGs) were analyzed with gene set analysis using the EnrichR package [2]. Such analysis was performed on all DEGs, taking the up- and downregulated genes separately. Gene Ontology (GO) [3], Kyoto Encyclopedia of Genes and Genomes (KEGG) [4], and WikiPathways [5] databases were used. Pathways containing at least 3 enriched genes and with p-values below 0.01 were considered significant. Transcription factor analysis was performed similarly by searching the Transcriptional Regulatory Relationships Unraveled by Sentence-based Text mining (TRRUST) database [6]. A transcription factor/protein interaction network was constructed and visualized with Igraph [7].

In situ staining for E-cadherin and CD4

Immunofluorescence staining of E-cadherin together with CD4 on 8-µm thick ectocervical tissue sections was performed as previously described [8]. Sections were probed with mouse anti-human E-Cadherin primary antibody (36/E-Cadherin, BD Biosciences, San Jose, CA, USA) followed by an Alexa Fluor 488-conjugated donkey anti-mouse IgG secondary antibody (highly cross absorbed) (A21202, Invitrogen, Thermo Fisher Scientific), as well as rabbit anti-human CD4 antibody (EPR6855, Abcam, Cambridge, UK), followed by an Alexa Fluor 594-conjugated donkey anti-rabbit IgG secondary antibody (highly cross absorbed, including affinity purification against mouse immunoglobulins) (A21207, Invitrogen, Thermo Fisher Scientific). All tissue sections were counterstained with 4′,6-diamidino-2-phenylindole (DAPI; Molecular Probes, Invitrogen, Thermo Fisher Scientific) and mounted with Fluorescent Mounting Medium (Dako, Carpinteria, CA, USA). Negative control staining was included with the primary antibody omitted.

In situ staining for desmoglein-1 and claudin-1

The cryopreserved ectocervical samples were cut into 8-µm thick sections using a cryostat. The tissue sections were then mounted onto SuperFrost® Gold Plus slides (Menzel Gläser, Thermo Fischer Scientific, VWR International AB, Spånga, Sweden), air dried for 1hr, followed by

fixation in 100% acetone (Sigma-Aldrich, St. Louis, MO, US) for 15 min at room temperature. The slides were dried for 10 min to allow evaporation of acetone, and thereafter washed in 1x PBS for 10 min, followed by an additional wash in 1x PBS wash buffer containing 1% HEPES (GE Healthcare, Chicago, IL, US) and 0.1% Saponin (Sigma). *In situ* staining for desmoglein-1 and claudin-1 was done sequentially using the monoclonal mouse anti-desmoglein-1 antibody and the monoclonal rabbit anti-claudin-1 antibody (clone 27B2 and EPRR18871, respectively, from Abcam, Waltham, MA, US) in combination with the highly cross-absorbed secondary antibodies Alexa Fluor 488–conjugated donkey anti-mouse IgG antibody and Alexa Fluor 555–conjugated donkey anti-rabbit IgG antibody, respectively (Invitrogen, Thermo Fisher Scientific). Negative controls were incubated with secondary antibody alone. All tissue sections were counterstained with 4',6-diamidino-2-phenylindole (DAPI) (Invitrogen, Thermo Fischer Scientific, Stockholm, Sweden) and the slides were mounted using fluorescent mounting media (Dako, Carpinteria, CA, USA). The slides were washed with the 1x PBS wash buffer between each incubation step.

Image analysis of E-cadherin and CD4 in the ectocervical epithelium

To set up digital image analysis workflows we used MATLAB v.9.0.0.341360 (R2016a) and CellProfiler v3.1.8 [9] to assess the *in situ* staining of E-cadherin and CD4. The expression of the E-cadherin was used to define four epithelial layers, as previously described [8]. Shortly, the superficial layer, which lacks expression of E-cadherin, was manually outlined by drawing a line separating the area devoid of E-cadherin staining from the underlying positively stained area, the intermediate (IM) layer. The IM layer was further separated into two parts, the upper IM layer, defined by a broken net structure, and the lower IM layer, defined by an intact net structure. Lastly, the parabasal layer was defined based on its high nucleus density. Three metrics were used to investigate epithelial barrier and integrity: 1) Total epithelial thickness, as well as the thickness of each of the four individual epithelial layers, was measured as an average of an apical-to-basal and basal-to-apical measurement, to account for the irregular shape of the ectocervical epithelium. 2) The percentage of E-cadherin area coverage was calculated by assessing the E-cadherin net area relative to the total epithelial area in the upper and lower IM layers and in the parabasal layer, as well as these three layers combined. 3) The mean fluorescence intensity (MFI) of E-cadherin staining in the entire identified net structure, as well as in the upper and lower IM layers and the parabasal layer, was determined.

A second digital image analysis workflow was used, as previously described (Edfeldt et al, JID, 2020) to assess the frequency and spatial localization of $CD4^+$ cells in the ectocervical epithelium. Shortly, the CD4 staining was segmented out using a white top-hat noise-reduction filter, together with an image-dependent intensity threshold (1.7 x the upper quartile intensity in each image). Autofluorescence background in the apical layer of the ectocervical epithelium was manually removed using EditObjectsManually module in CellProfiler. $CD4^+$ cells in the ectocervical epithelium have an irregular morphology, and consequently, it is difficult to assign stained areas to a specific cell nucleus. Therefore, the percentage of positively stained area per total tissue area was used as proxy for the percentage of positive cells, and the following three measurements were assessed: 1) frequency of $CD4^+$cells per total tissue area in the total epithelium, as well as in each of the four individual layers; 2) proportion of $CD4^+$cells in each of the four individual layers; and 3) average distance from $CD4^+$cells to the apical border.

Image analysis of desmogelin-1 and claudin-1 expression in the ectocervical epithelium

The epithelial compartment was outlined by manual annotations, i.e., regions of interest (ROI) in CaseViewer (version 2.4, 3DHistech Ltd., Budapest, Hungary). Between two to six ROIs per tissue sample were selected and each ROI was exported as independent greyscale image for each channel (FITC, Cy3 and DAPI) and were analyzed independently. Image analysis was performed using Fiji (v1.53c) [10] and MATLAB (vR2020b, MathWorks, Natick, MA, US). The apical border and the basal membrane were manually outlined using Fiji, to enable analysis of only the epithelium, i.e., excluding the cervical submucosa and lumen from analysis. The apical and basal lines were connected by straight lines creating a polygon, masking the epithelium in the ROI, which functioned as a basis for all further image analysis. The net-like structures of the desmoglein-1 and claudin-1 staining were detected using a contrast-independent approach, which highlights curvilinear structures and is less sensitive to intensity variations. This method was proposed by Obara et. al [11] and a MATLAB implementation of the method was used to obtain the enhanced nets of desmoglein-1 and claudin-1. Fiji was used for all the following steps to assess the desmoglein-1 and claudin-1 staining. First, the net structure was segmented using the Otsu threshold [12], which allows for maximum intra-class variation with respect to the image histogram. Next, the obtained threshold value was multiplied by a correction factor of 1.6 in order to optimize the segmented nets. Based on the segmented nets, a Euclidian distance transform (EDT) map was created [13]. The EDT expands all segmented objects to connect the segmented nets enabling the analysis of fragmented net structures. A distance threshold of 15 was used to create the final binary mask of the

desmoglein-1 and claudin-1 nets. Artifacts and autofluorescence, present in the apical layer of the epithelium with area smaller than 6 200 um^2 (50 000 pixels) were removed from the desmoglein-1 and claudin-1 net. Thereafter, EDT maps from the nets to the manually outlined apical line was used to segment the upper desmoglein-1 or claudin-1 negative layers (called upper layer), and from the basal line to the segment the lower desmoglein-1 and claudin-1 negative layers (called lower layer), respectively. Morphological operators were applied in order to enhance the segmented results [14]. These steps collectively segmented the epithelium into three layers: upper layer, desmoglein-1^+ or claudin-1^+ layer and lower layer.

An average epithelial thickness was calculated to get the most accurate representation of the epithelial height as previously described [8]. Briefly, the distance from each point on the apical line to the nearest point on the basal line was calculated by creating an EDT map from the apical line to the corresponding basal line, and vice versa. The average thickness of the three segmented layers was calculated using the same method.

Thus, by utilizing the masked epithelium, the total area and height of the whole epithelium, the height of the three separate layers as well as the relative height of the three layers were calculated. Moreover, within the desmoglein-1^+ and claudin-1^+ layers, the MFI of the staining was calculated. In images where no desmoglein-1 or claudin-1 could be detected, height measurements of upper or lower layer could not be performed and were hence removed from analysis of these parameters.

Protein profiling using suspension bead arrays

Antibodies from Human Protein Atlas (www.proteinatlas.org) targeting a panel of pre-selected proteins (n=74) were immobilized onto color-coded and carboxylated magnetic beads using EDC-NHS chemistry, as described previously [15]. All bead identities were pooled together after coupling of antibodies, to form the suspension bead array. The full protein content of the CVL samples were biotinylated, in the same manner as previously described for cervicovaginal secretions [16]. Each sample was diluted 1/2 in PBS supplemented with bovine serum albumin (Sigma-Aldrich, Stockholm, Sweden) and rabbit IgG (Bethyl Laboratories, Montgomery, TX, US) and directly labelled using a 10x molar excess of biotin over total protein amount. A second dilution of 1/50 was performed in a buffer composed of 0.5% w/v polyvinylalcohol, 0.8% w/v polyvinylpyrrolidone and 0.1% w/v casein (all SigmaAldrich) before the samples were heat-treated at 56°C for 30 min. The

labelled and heat-treated samples were combined with the suspension bead array and incubated overnight at room temperature. The unbound proteins were removed by washing and the captures proteins were crosslinked to the immobilized antibodies using 0.4% paraformaldehyde (Alfa Aesar, Thermo Scientific, Stockholm, Sweden). Detection was enabled using a streptavidin-conjugated fluorophore (R-phycoerythrin, Invitrogen Thermo Scientific, Stockholm, Sweden) and read out was performed in a Flexmap 3D instrument (Luminex corporation, Austin, TX, US) where binding events were reported as fluorescence intensity in arbitrary units. The crosslinking, washing and detection was performed as described previously [15].

The open source software R was used for data processing and visualizations [17]. The data was log10 transformed before normalization. The normalization procedure consisted of two steps, first a robust linear regression (rlm from package MASS) was used to diminish the effects of delay time during read out. The median signal intensity per protein was added to the obtained residuals. In addition, differences between 96-well plates were reduced [18]. Mann-Whitney *U* test (wilcox.test from package stats) were used to evaluate differences of protein levels between groups where a p-value below 0.05 was regarded significant. Generalized linear models (glm from package stats) were used to investigate the effect of potential confounders.

References

1. Anahtar MN, Bowman BA, Kwon DS. Efficient Nucleic Acid Extraction and 16S rRNA Gene Sequencing for Bacterial Community Characterization. J Vis Exp. 2016;(110). doi: 10.1016/j.immuni.2015.04.019.

2. Kuleshov MV, Jones MR, Rouillard AD, Fernandez NF, Duan Q, Wang Z, et al. Enrichr: a comprehensive gene set enrichment analysis web server 2016 update. Nucleic Acids Res. 2016;44(W1):W90-7. doi: 10.1093/nar/gkw377.

3. Gene Ontology Consortium. The Gene Ontology resource: enriching a GOld mine. Nucleic Acids Res. 2021;49(D1):D325-d34. doi: 10.1093/nar/gkaa1113.

4. Kanehisa M, Furumichi M, Sato Y, Ishiguro-Watanabe M, Tanabe M. KEGG: integrating viruses and cellular organisms. Nucleic Acids Res. 2021;49(D1):D545-d51. doi: 10.1093/nar/gkaa970.

5. Martens M, Ammar A, Riutta A, Waagmeester A, Slenter DN, Hanspers K, et al. WikiPathways: connecting communities. Nucleic Acids Res. 2021;49(D1):D613-d21. doi: 10.1093/nar/gkaa1024.

6. Han H, Cho JW, Lee S, Yun A, Kim H, Bae D, et al. TRRUST v2: an expanded reference database of human and mouse transcriptional regulatory interactions. Nucleic Acids Res. 2018;46(D1):D380-d6. doi: 10.1093/nar/gkx1013.

7. Csárdi G, Nepusz T. The igraph software package for complex network research. InterJournal Complex Systems. 2006.

8. Edfeldt G, Lajoie J, Röhl M, Oyugi J, Åhlberg A, Khalilzadeh-Binicy B, et al. Regular use of depot medroxyprogesterone acetate causes thinning of the superficial lining and apical distribution of HIV target cells in the human ectocervix. J Infect Dis. 2020. doi: 10.1093/infdis/jiaa514.

9. McQuin C, Goodman A, Chernyshev V, Kamentsky L, Cimini BA, Karhohs KW, et al. CellProfiler 3.0: Next-generation image processing for biology. PLoS Biol. 2018. doi: 10.1371/journal.pbio.2005970.

10. Schindelin J, Arganda-Carreras I, Frise E, Kaynig V, Longair M, Pietzsch T, et al. Fiji: an open-source platform for biological-image analysis. Nat Methods. 2012;9(7):676-82. doi: 10.1038/nmeth.2019

11. Obara B, Fricker M, Gavaghan D, Grau V. Contrast-independent curvilinear structure detection in biomedical images. IEEE Trans Image Process. 2012;21(5):2572-81. doi: 10.1109/tip.2012.2185938.

12. Otsu N. A Threshold Selection Method from Gray-Level Histograms. IEEE Transactions on Systems, Man, and Cybernetics. 1979;9(1):62 - 6. doi: 10.1109/TSMC.1979.4310076.

13. Leymarie F, Levine MD. Fast raster scan distance propagation on the discrete rectangular lattice. CVGIP: Image Understanding. 1992:84-94. doi: 10.1016/1049-9660(92)90008-Q.

14. Legland D, Arganda-Carreras I, Andrey P. MorphoLibJ: integrated library and plugins for mathematical morphology with ImageJ. Bioinformatics. 2016;32(22):3532-4. doi: 10.1093/bioinformatics/btw413.

15. Pin E, Sjöberg R, Andersson E, Hellström C, Olofsson J, Jernbom Falk A, et al. Array-Based Profiling of Proteins and Autoantibody Repertoires in CSF. Methods Mol Biol. 2019;2044:303-18. doi: 10.1007/978-1-4939-9706-0_19.

16. Manberg A, Bradley F, Qundos U, Guthrie BL, Birse K, Noel-Romas L, et al. A High-throughput Bead-based Affinity Assay Enables Analysis of Genital Protein Signatures in Women At Risk of HIV Infection. Mol Cell Proteomics. 2019;18(3):461-76. doi: 10.1074/mcp.RA118.000757.

17. R Core Team. R: A language and environment for statistical computing. 2020. [cited 10 Aug 2021]. Available from: www.r-project.org.

18. Hong MG, Lee W, Nilsson P, Pawitan Y, Schwenk JM. Multidimensional Normalization to Minimize Plate Effects of Suspension Bead Array Data. J Proteome Res. 2016;15(10):3473-80. doi: 10.1021/acs.jproteome.5b01131.
